# The host tropism of current zoonotic H7N9 viruses depends mainly on an acid-labile hemagglutinin with a single amino acid mutation in the stalk region

**DOI:** 10.1371/journal.ppat.1012427

**Published:** 2024-10-22

**Authors:** Tomo Daidoji, Hiroki Sadakane, Kotaro Garan, Norihito Kawashita, Yasuha Arai, Yohei Watanabe, Takaaki Nakaya

**Affiliations:** 1 Department of Pathobiology, School of Veterinary Medicine, Rakuno Gakuen University, Hokkaido, Japan; 2 Department of Infectious Diseases, Graduate School of Medical Science, Kyoto Prefectural University of Medicine, Kyoto, Japan; 3 Faculty of Science and Engineering, Kindai University, Osaka, Japan; Universite Laval Faculte de medecine, CANADA

## Abstract

The incidence of human infection by zoonotic avian influenza viruses, especially H5N1 and H7N9 viruses, has increased. Current zoonotic H7N9 avian influenza viruses (identified since 2013) emerged during reassortment of viruses belonging to different subtypes. Despite analyses of their genetic background, we do not know why current H7N9 viruses are zoonotic. Therefore, there is a need to identify the factor(s) responsible for the extended host tropism that enables these viruses to infect humans as well as birds. To identify H7N9-specific amino acids that confer zoonotic properties on H7N9 viruses, we performed multiple alignment of the hemagglutinin (HA) amino acid sequences of A/Shanghai/1/2013 (H7N9) and A/duck/Zhejiang/12/2011(H7N3) (a putative, non- or less zoonotic HA donor to the zoonotic H7N9 virus). We also analyze the function of an H7N9 HA-specific amino acid with respect to HA acid stability, and evaluated the effect of acid stability on viral infectivity and virulence in a mouse model. HA2-116D, preserved in current zoonotic H7N9 viruses, was crucial for loss of HA acid stability. The acid-labile HA protein in H7 viruses played an important role in infection of human airway epithelial cells; HA2-116D contributed to infection and replication of H7 viruses. Finally, HA2-116D served as a H7 virulence factor in mice. These results suggest that acid-labile HA harboring HA2-116D confers zoonotic characteristics on H7N9 virus and that future novel zoonotic avian viruses could emerge from non-zoonotic H7 viruses via acquisition of mutations that remove HA acid stability.

## Author summary

Despite detailed analyses of the genetic background, we do not know why H7N9 avian influenza viruses circulating since 2013 behave as zoonotic viruses. Here, we (i) identified a specific amino acid, HA2-116D, in the HA protein that confers zoonotic properties on the H7N9 virus; (ii) show that HA2-116D plays an important role in viral infectivity and replication in human airway epithelial cells through its ability to increase pH sensitivity, which readily induces viral-cell membrane fusion in host cells and subsequent infection; (iii) show that HA2-116D is not usually present in the HA protein of H7 viruses with low zoonotic potential; and (iv) show that HA2-116D increases the virulence of the H7 virus in mice. These results suggest that acid-labile HA is an important factor underlying efficient infection of human airway epithelial cells, as well as a virulence factor for mammalian hosts, and that novel zoonotic avian viruses could emerge from non-zoonotic H7 viruses that acquire mutations resulting in loss of HA acid stability.

## Introduction

Recently, several subtypes of avian influenza virus (AIV) such as H5N1, H5N6, H5N8, H6N1, H7N2, H7N3, H7N4, H7N7, H7N9, H9N2, H10N3, H10N7, and H10N8 have been identified as zoonotic [1–19]. Of these, infection of humans by H5N1 and H7N9 viruses has increased (between 2003–2023, 878 cases of human infection with H5N1 virus was reported by the WHO (https://www.who.int/publications/m/item/cumulative-number-of-confirmed-human-cases-for-avian-influenza-a(h5n1)-reported-to-who—2003-2023-14-july-2023). The first case of human infection by H7N9 avian influenza virus (AIV) was reported in Shanghai, China, in February 2013 [20,21]. Since February 2013, H7N9 virus has spread country-wide. Indeed, 1568 human cases and 616 deaths were reported as of August 2023 (https://apps.who.int/iris/handle/10665/365675). Although H7N9 has not yet achieved human-to-human transmission, the virus can be transmitted directly from birds to humans [20–25] and is highly pathogenic, including severe disease with high mortality.

Current zoonotic H7N9 viruses identified since 2013 emerged due to reassortment of viruses belonging to different subtypes. This reassortment resulted in hemagglutinin (HA) and neuraminidase (NA) proteins that are genetically related to duck-origin H7N3 viruses and duck- or wild bird-origin N9 viruses, respectively; other 6-segmented genomes are closely related to H9N2 viruses circulating in poultry in China during a similar time period (2010–2012) [20,21,26–28]. Although detailed genetic analyses have been conducted, it remains unclear why current H7N9 viruses behave as zoonotic viruses that infect both avian and human hosts. Therefore, it is important to identify the factor(s) responsible for the broad host tropism of current zoonotic H7N9 viruses and to identify the mechanism of infection in human hosts.

The surface HA protein of influenza virus plays a major role in infection of host cells. The HA protein has two different functions: receptor binding and membrane fusion. After receptor binding and viral internalization via endocytosis, influenza viruses induce fusion between the viral and endosomal membranes through a low pH-dependent conformational change in the HA protein [29–34]. Previously, we showed a positive correlation between the pH threshold for HA activation to induce membrane fusion and the ability of AIVs to infect human airway epithelial cells [35,36]. In these experiments, current zoonotic H5N1 viruses exhibited efficient infection of and replication in human tracheal and/or bronchiolar epithelial cells via the HA protein, which showed an elevated pH threshold for activation. By contrast, non-zoonotic AIVs belonging to H5N3 and H5N9 subtypes were not activated, and did not become infectious until their HA proteins were exposed to lower pH [35,36], suggesting that elevated pH values for HA activation are associated with the broad host tropism of current zoonotic H5N1 viruses. Because current zoonotic H7N9, as well as H5N1, viruses behave as zoonotic viruses with high mortality rates in humans, we hypothesized that current zoonotic H7N9 viruses also exhibit efficient infection and replication in human airway epithelial cells with elevated pH values for HA activation, and that these properties make H7N9 viruses zoonotic. By contrast, because no putative donor virus for the H7N9 HA gene [although a reliable candidate is A/duck/Zhejiang/12/2011(H7N3), referred to as Dk/ZJ (H7N3) [20,21,26–28]], or related avian viruses, have been shown to infect human hosts, we also hypothesize that zoonotic H7N9 viruses that appeared in 2013 became zoonotic after acquisition of amino acid mutations via the HA gene of the donor virus, i.e., Dk/ZJ (H7N3), resulting in increased pH sensitivity.

Here, to verify our hypothesis regarding the infectious mechanism of current zoonotic H7N9 viruses, we compared the HA acid stability of A/Shanghai/1/2013 (H7N9) [Shanghai (H7N9)], which is a prototype of the current zoonotic H7N9 virus, with that of Dk/ZJ (H7N3), a non-zoonotic virus that is a strong candidate as the HA donor for current zoonotic H7N9 viruses. We also tried to identify the amino acid responsible for the HA acid stability of H7N9 viruses, and evaluated the effect of amino acid mutations on the ability of the virus to infect human bronchiolar epithelial cells. In addition, we evaluated the effect of a H7HA protein with increased pH sensitivity on pathogenicity in an animal model.

## Results

### Alignment of HA amino acid between H7N3 and H7N9 viruses

**Fig 1:**
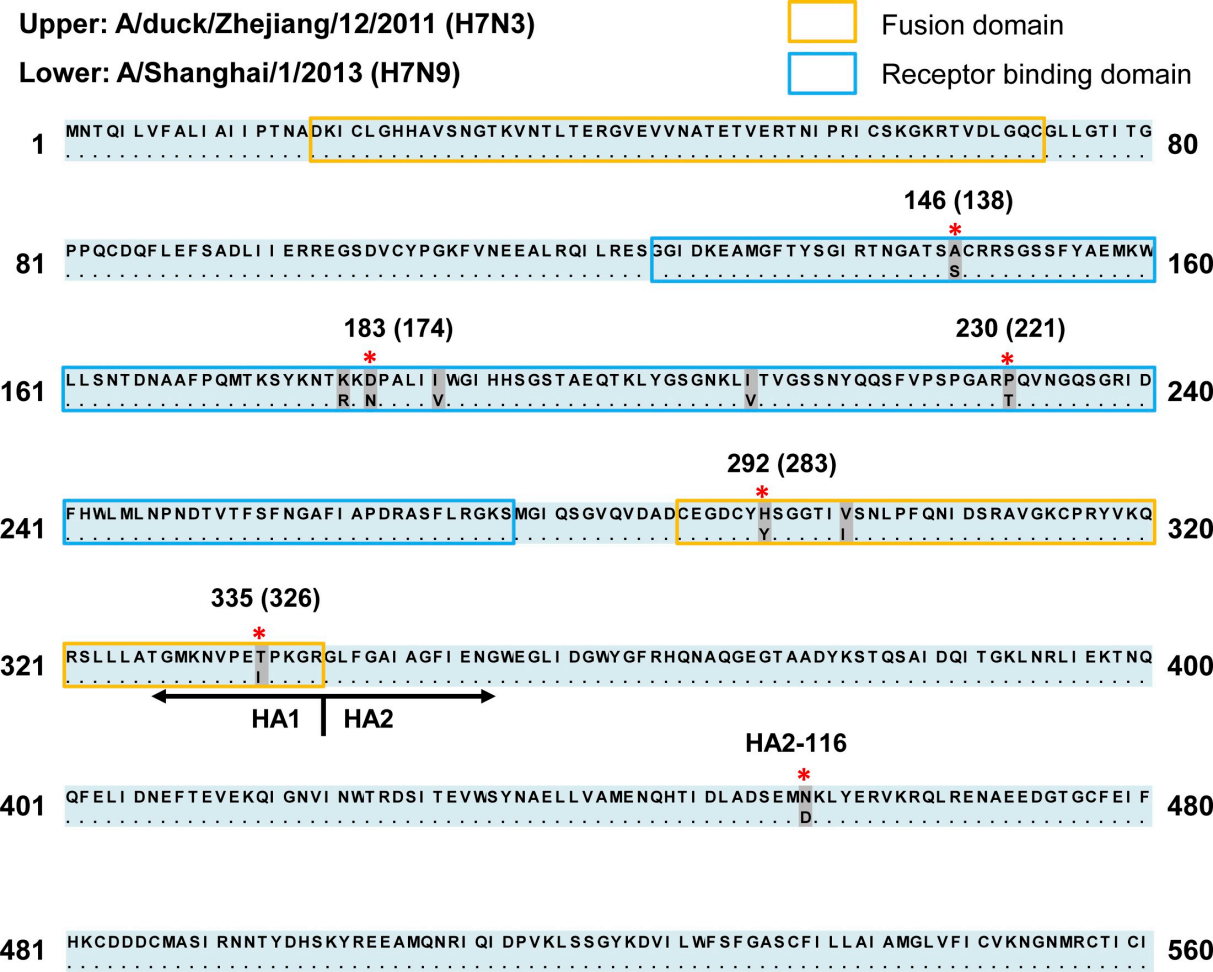
Comparison of the HA protein of current zoonotic H7N9 viruses with that of a non-zoonotic H7N3 strain. The amino acid sequence of the HA protein of Shanghai (H7N9), a prototype strain of the current zoonotic H7N9, was compared with that of Dk/ZJ (H7N3), a putative donor of the HA gene during virus reassortment. Amino acid differences are shown in gray. The six amino acids that differ in terms of physicochemical properties are depicted by both asterisks and numbers (H3 numbering is shown in parentheses).

First, we tested the hypothesis that zoonotic H7N9, which emerged in 2013, acquired crucial amino acids, which affect HA acid stability and allow efficient infection of human cells, from the HA protein supplied by the ancestor virus belonging to the H7N3 subtype. Because Dk/ZJ (H7N3) is thought to be a putative donor of the HA genome to current zoonotic H7N9 viruses [20,21,26–28], we aligned the HA amino acid sequence of Dk/ZJ (H7N3) with that of Shanghai (H7N9), which is a prototype strain for current zoonotic H7N9 viruses [20,21,26–28]. The alignment revealed ten amino acid differences between Dk/ZJ (H7N3) and Shanghai (H7N9) (Fig 1). We focused on six amino acids with distinct physicochemical properties. Of these, three were located in the receptor binding domain, two were located in the fusion domain, and one was located in the stalk region (Fig 1). Further alignment of HA amino acids revealed that 326I and HA2-116D (based on H3 numbering) were highly conserved in Shanghai (H7N9) and other strains of current zoonotic H7N9 viruses identified since 2013 (S1 Fig).

### Identification of the crucial amino acid that affects the HA acid stability of current zoonotic H7N9 viruses

**Fig 2:**
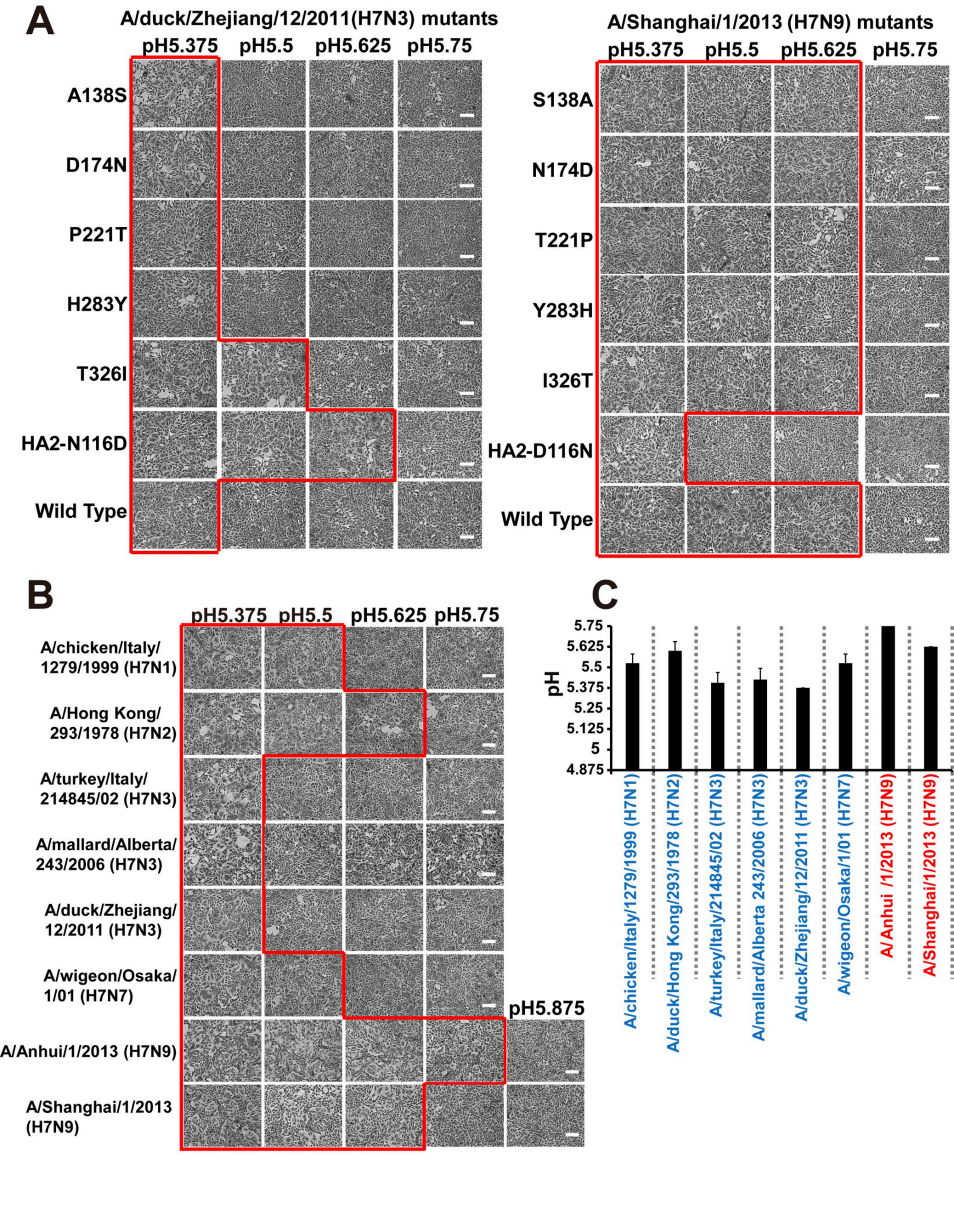
Virus-cell membrane fusion at low pH in MDCK cells expressing the HA protein of H7 viruses. (A, B) MDCK cells were transfected with the influenza virus HA gene from Dk/ZJ (H7N3) or Shanghai (H7N9), or with HA from their respective HA mutants (A), or with HA from several zoonotic and non-zoonotic H7 viruses [Anhui (H7N9), Shanghai (H7N9), Ck/Ita (H7N1), Dk/Hk (H7N2), Tk/Ita/214845 (H7N3), Mal/Alb(H7N3), Dk/ZJ (H7N3), or Wg/Os (H7N7)] (B). Fusion induction over a pH range of 5.375–5.875 (increasing in increments of 0.125) was conducted at 24 h post-transfection. Representative fields of cells transfected with each of the indicated viruses and exposed to low pH are shown. Red squares show polykaryon formation. Micrographs lacking a red square represent a pH above the fusion threshold (A, B). The pH threshold was determined as described in “Materials and Methods.” Scale bars, 200 μm. (C) Summary of the pH threshold for membrane fusion for all viral subtypes in transfected cells. Data are expressed as the mean ± S.D. of four [Anhui (H7N9) and Tk/Ita/214845 (H7N3)], five [Ck/Ita (H7N1), Dk/Hk (H7N2), Mal/Alb (H7N3), Dk/ZJ (H7N3), or Wg/Os (H7N7)], or six [Shanghai (H7N9)] independent results.

Next, we used a membrane fusion assay [36,37] to examine the role of the six HA amino acids that differ between Dk/ZJ (H7N3) and Shanghai (H7N9). Cells were transfected with HA expression plasmids harboring wild-type Dk/ZJ (H7N3), or with Shanghai (H7N9), or with their respective mutant HA clones, prior to the assay. The highest pH at which the HA protein showed fusogenic activity was then measured, and the pH threshold for HA activation was determined. The membrane fusion assay showed that the pH threshold for wild-type Dk/ZJ (H7N3), and HA mutants A138S, D174N, P221T, and H283Y [based on H3 numbering], was 5.375, whereas that for mutants T326I and HA2-N116D was 5.5 and 5.625, respectively (Fig 2A). The pH threshold for wild-type Shanghai (H7N9) and HA mutants S138A, N174D, T221P, Y283H, and I326T [based on H3 numbering] was 5.625, whereas that for HA2-D116N was 5.375 (Fig 2A). These results suggest that amino acid HA2-116 is crucial for HA acid stability, and that the mutation HA2-116D results in loss of HA stability. In addition, we used the same assay to examine the HA acid stability of other non-zoonotic H7 viruses lacking HA2-116D. The pH threshold for non-zoonotic viruses was lower (5.375–5.5) than that for Shanghai (H7N9); the exception was A/duck/Hong Kong/293/1978 (H7N2), with a pH threshold of 5.625 (Fig 2B and 2C).

### Structural analysis based on a HA model of non-zoonotic H7N3 and current zoonotic H7N9 viruses

**Fig 3:**
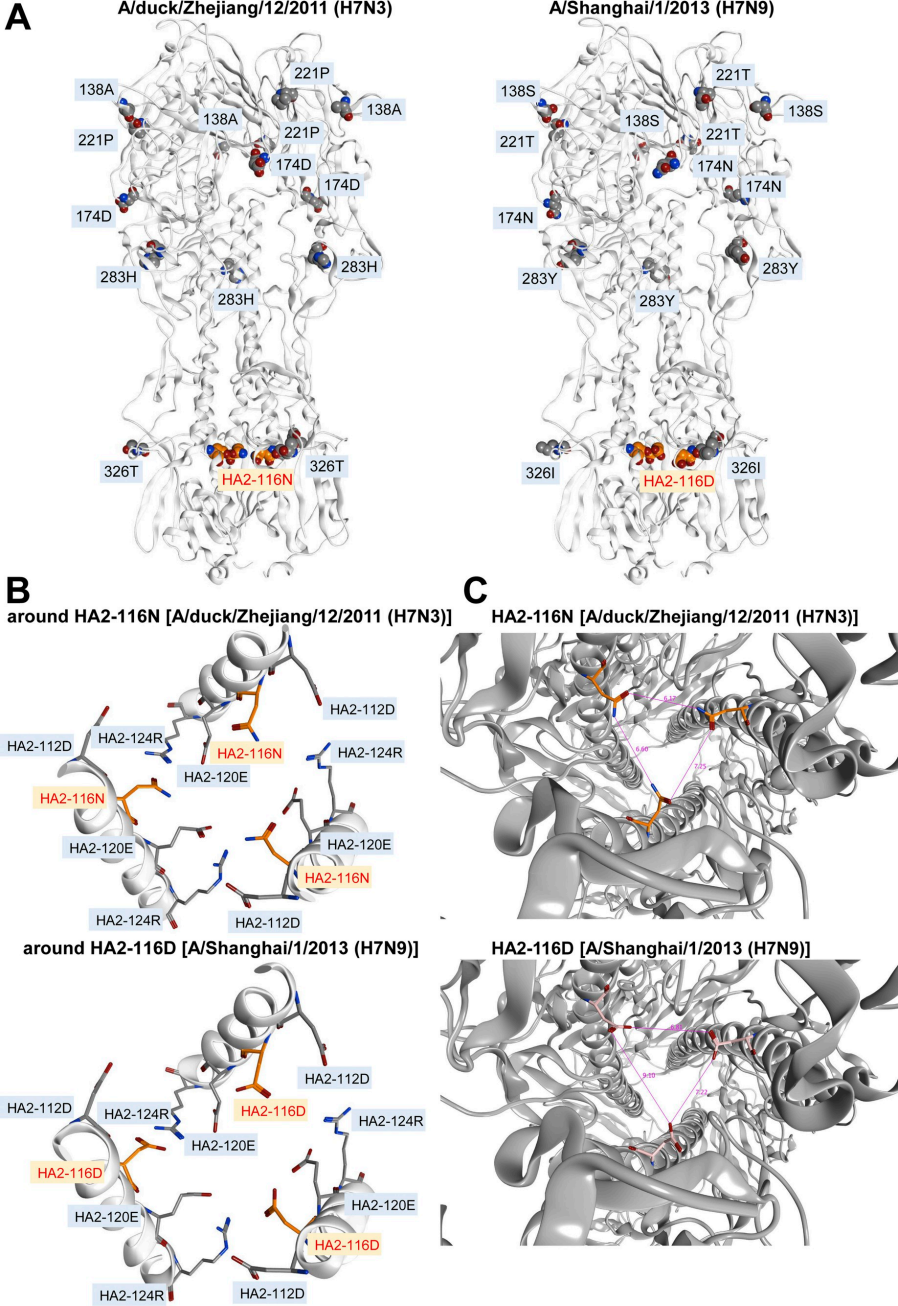
Structural model of the HA proteins of Dk/ZJ (H7N3) and Shanghai (H7N9). (A) Ribbon model of the HA trimer from Dk/ZJ (H7N3) and Shanghai (H7N9). Key amino acid residues are shown in the space-filling model. (B) Close-up view of the area around the HA2-116N [Dk/ZJ (H7N3)] or HA2-116D [Shanghai (H7N9)] residue in the HA stalk region. Key amino acid residues are labeled. (C) The distance between individual HA stalks in Dk/ZJ (H7N3) and Shanghai (H7N9) was compared. The values indicate the distance between individual HA stalks.

The importance of HA2-116D in current zoonotic H7N9 viruses led us to conduct a structural analysis of HA trimetric models to examine its function. First, we generated models of the HA trimetric structure based on the recently obtained X-ray crystal structure of the HA protein of influenza A virus (Protein Data Bank ID code 4LN3). Our models showed that HA2-116N and HA2-116D, which belong to Dk/ZJ (H7N3) and Shanghai (H7N9), respectively, are located in the stalk region of the HA trimer (Fig 3A). HA modeling based on the structure of HA2-116N and HA2-116D yielded different results: the calculated interaction energies between these amino acids and the other residues was -0.39 (average value) kcal/mol for HA2-116N, and 4.91 (average value) kcal/mol for HA2-116D (Fig 3B); and the distance between each HA monomer within the HA trimer of Shanghai (H7N9) was greater than that of Dk/ZJ (H7N3) (Fig 3C).

### The importance of HA acid stability for viral infectivity of human airway epithelial cells

**Fig 4:**
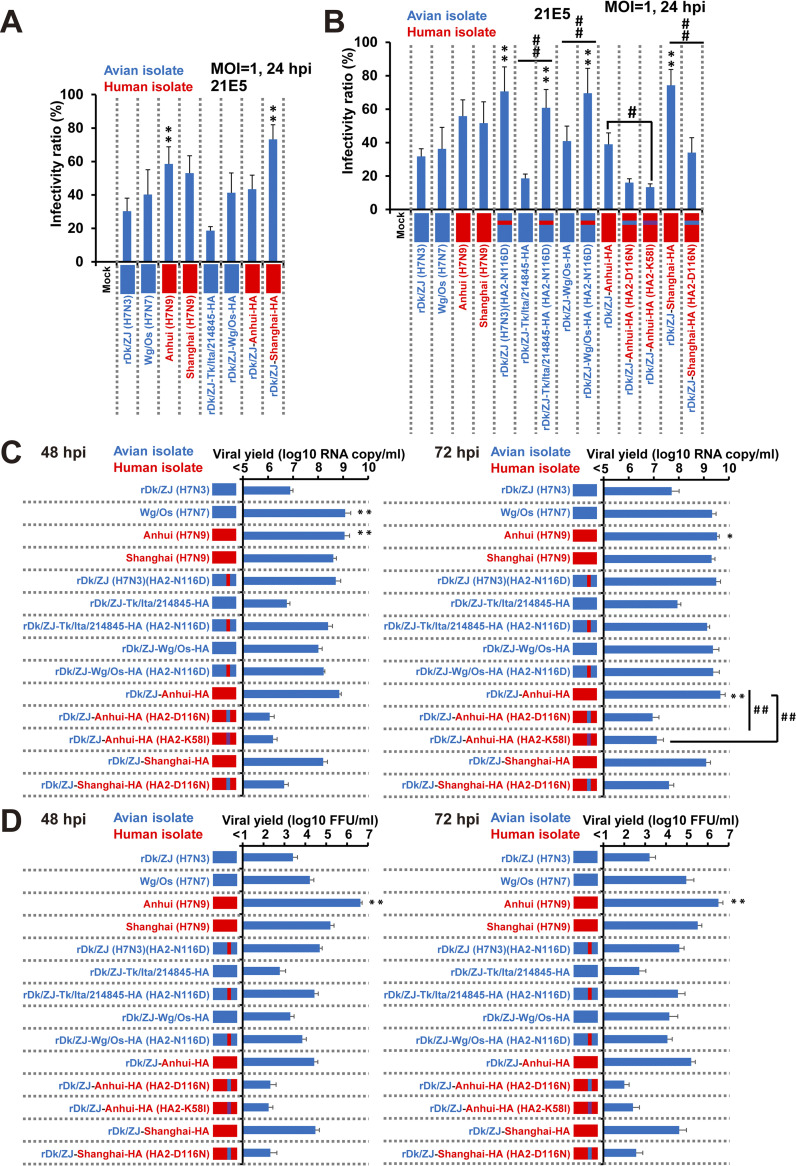
The infectivity and growth kinetics of current H7N9 and other H7 viruses in immortalized human bronchiolar epithelial cells. (A, B) 21E5 cells, derived from primary human bronchiolar epithelial cells [please see “Materials and Methods”] were infected with rDk/ZJ (H7N3), Wg/Os (H7N7), Anhui (H7N9), or Shanghai (H7N9), or with recombinant Dk/ZJ (H7N3) [rDk/ZJ-Tk/Ita/214845-HA, rDk/ZJ-Wg/Os-HA, rDk/ZJ-Anhui-HA, or rDk/ZJ-Shanghai-HA] (A), or with recombinant Dk/ZJ (H7N3), or the HA mutants [rDk/ZJ (H7N3) (HA2-N116D), rDk/ZJ-Tk/Ita/214845-HA (HA2-N116D), rDk/ZJ-Wg/Os-HA (HA2-N116D), rDk/ZJ-Anhui-HA (HA2-D116N), rDk/ZJ-Anhui-HA (HA2-K58I), and rDk/ZJ-Shanghai-HA (HA2-D116N)] (B). All cells were infected at an m.o.i. of 1. Viral infectivity was determined by calculating the percentage of antigen-positive cells after immunostaining at 24 h post-infection. Data are expressed as the mean ± S.D. of four independent results. (C) 21E5 cells were infected with rDk/ZJ (H7N3), Wg/Os (H7N7), Shanghai (H7N9), or Anhui (H7N9), or with recombinant Dk/ZJ (H7N3) [rDk/ZJ-Tk/Ita/214845-HA, rDk/ZJ-Wg/Os-HA, rDk/ZJ-Anhui-HA, or rDk/ZJ-Shanghai-HA], and the respective HA mutants [rDk/ZJ (H7N3) (HA2-N116D), rDk/ZJ-Tk/Ita/214845-HA (HA2-N116D), rDk/ZJ-Wg/Os-HA (HA2-N116D), rDk/ZJ-Anhui-HA (HA2-D116N), rDk/ZJ-Anhui-HA (HA2-K58I), and rDk/ZJ-Shanghai-HA (HA2-D116N)]. All cells were infected at an m.o.i. of 0.1. The amount of progeny vRNA within the culture supernatants at 48 and 72 h post-infection was determined by measuring virus titers in quantitative real-time PCR assays (the growth curves of released virions at 12, 24, 48, 72, 96, and 120 h post-infection are shown in S4A Fig). (D) 21E5 cells were infected with the same virus used in (C) at an m.o.i. of 0.1. The infectious titer of the released virions within the culture supernatants at 48 and 72 h post-infection was determined in a focus-forming assay (the growth curves based on the infectious virus titer of released virions at 12, 24, 48, 72, 96, and 120 h post-infection are shown in S4B Fig). Data are expressed as the mean ± S.D. of three (C) or four (D) independent results. Asterisks indicate that the values for each virus were significantly different from that of rDk/ZJ (H7N3) within the same graph (A–D); sharps indicate that the values for each mutated virus (HA2-N116D, HA2-D116N, or HA2-K58I) were significantly different from that of each virus without the mutation within the same graph (B–D). A p-value < 0.05 (single asterisk or sharp) or < 0.01 (double asterisk or double sharp) was considered significant (one-way ANOVA followed by Tukey’s multiple comparisons post-hoc test; A–D). The mutated amino acids (HA2-N116D or HA2-D116N) are shown in red (HA2-N116D) and blue (HA2-D116N), respectively. The mutated amino acid HA2-K58I is shown in purple.

To evaluate the effect of HA acid stability on the ability of viruses to infect human airway epithelial cells, we generated recombinant Dk/ZJ (H7N3) possessing the HA gene of zoonotic H7N9 viruses [A/Anhui/1/2013(H7N9)[Anhui (H7N9)] and Shanghai (H7N9), denoted here as rDk/ZJ-Anhui-HA and rDk/ZJ-Shanghai-HA, respectively)]; and non-zoonotic H7 viruses [A/Turkey/Italy/214845/02 (H7N3)[Tk/Ita/214845 (H7N3)] and A/wigeon/Osaka/1/01 (H7N7)[Wg/Os (H7N7)], denoted here as rDk/ZJ-Tk/Ita/214845-HA and rDk/ZJ-Wg/Os-HA, respectively)]. The HA protein of these viruses showed a different pH threshold for HA activation to induce membrane fusion (Fig 2B and 2C). Next, we infected airway epithelial cell clone 21E5 [36] with these recombinant viruses. As shown in Fig 4A, Anhui (H7N9), Shanghai (H7N9), and recombinant Dk/ZJ (H7N3) harboring the H7N9 HA gene with an elevated pH threshold (pH 5.625–5.75) showed higher infectivity than the parent virus rDk/ZJ (H7N3) (pH 5.375), although the infectivity of rDk/ZJ-Anhui-HA was not as high as that of rDk/ZJ-Shanghai-HA. Recombinant Dk/ZJ (H7N3) harboring the HA gene of Tk/Ita/214845 (H7N3), which has a lower pH threshold for HA protein activation (pH 5.375), showed similar infectivity to that of the parent virus rDk/ZJ (H7N3). Wild-type Wg/Os (H7N7) and recombinant Dk/ZJ (H7N3) harboring the HA gene of Wg/Os (H7N7), with a modest pH threshold for HA protein activation (pH 5.5), also showed higher infectivity than the parent virus rDk/ZJ (H7N3) (Fig 4A).

### Role of the HA2-116 mutation in viral infectivity of human airway epithelial cells

To examine the infectious mechanism underlying HA-mediated membrane fusion, we infected 21E5 cells with recombinant Dk/ZJ (H7N3) possessing the HA amino acid mutation at HA2-116. As shown in Fig 4B, recombinant Dk/ZJ (H7N3) [denoted here as rDk/ZJ (H7N3)(HA2-N116D)] showed significantly higher infectivity than the parent virus rDk/ZJ (H7N3), which has the amino acid “N” at HA2-116. In addition, rDk/ZJ-Tk/Ita/214845-HA and rDk/ZJ-Wg/Os-HA harboring the mutation HA2-N116D [denoted here as rDk/ZJ-Tk/Ita/214845-HA (HA2-N116D) and rDk/ZJ-Wg/Os-HA (HA2-N116D), respectively] showed significantly higher infectivity than the respective parental viruses (rDk/ZJ-Tk/Ita/214845-HA and rDk/ZJ-Wg/Os-HA, respectively) harboring HA2-116N. However, rDk/ZJ-Shanghai-HA harboring the mutation HA2-D116N [denoted here as rDk/ZJ-Shanghai-HA (HA2-D116N)] showed significantly lower infectivity than the parent virus (rDk/ZJ-Shanghai-HA harboring HA2-116D). The rDk/ZJ-Anhui-HA virus harboring HA2-D116N [denoted here as rDk/ZJ-Anhui-HA (HA2-D116N)] showed traits similar to those of rDk/ZJ-Shanghai-HA (HA2-D116N). The rDk/ZJ-Anhui-HA virus harboring HA2-K58I, which stabilizes HA [38,39] [denoted here as rDk/ZJ-Anhui-HA (HA2-K58I)] also showed significantly lower infectivity than the parent virus rDk/ZJ-Anhui-HA harboring HA2-116D. The pH threshold (range) for HA activation of viruses with or without amino acid 116D was ΔpH +0.25–0.375 (HA2-N116D) or -0.25 (HA2-D116N) (S2A and S2B Fig). The loss of HA acid stability, mediated by HA2-116D, was also reflected in the HA thermostability observed in the HA assay (S3 Fig). These results suggest that the amino acid at HA2-116 plays a key role in HA stability, and that HA2-116D is crucial for efficient viral infectivity of human airway epithelial cells.

### The role of HA acid stability and the HA2-116 mutation in viral growth kinetics in human airway epithelial cells

Because the acid stability of the HA protein contributes to viral infectivity of human airway epithelial cells, we also evaluated the effect of HA acid stability on viral growth in 21E5 cells. The growth kinetics of Anhui (H7N9), Shanghai (H7N9), Wg/Os (H7N7), and recombinant Dk/ZJ (H7N3) possessing H7N9 HA genes (rDk/ZJ-Anhui-HA and rDk/ZJ-Shanghai-HA) were significantly higher than those of the parent virus rDk/ZJ (H7N3) (Figs 4C and S4A). The growth kinetics of rDk/ZJ-Tk/Ita/214845-HA were similar to those of the parent virus rDk/ZJ (H7N3) (Figs 4C and S4A). In addition, the growth kinetics of recombinant viruses harboring HA2-N116D [i.e., rDk/ZJ (H7N3) (HA2-N116D), rDk/ZJ-Tk/Ita/214845-HA (HA2-N116D), and rDk/ZJ-Wg/Os-HA (HA2-N116D)] were also higher than those of their parent viruses [rDk/ZJ (H7N3), rDk/ZJ-Tk/Ita/214845-HA, and rDk/ZJ-Wg/Os-HA, respectively], despite the fact that there is no significant difference between the recombinant virus with the HA mutation and the parent virus (Figs 4C and S4A). However, the growth kinetics of recombinant viruses with HA mutation HA2-D116N [rDk/ZJ-Anhui-HA (HA2-D116N)] or the HA mutation HA2-K58I [rDk/ZJ-Anhui-HA (HA2-K58I)] were significantly lower than those of the parental virus [rDk/ZJ-Anhui-HA] harboring 116D (Fig 4C). The growth kinetics of rDk/ZJ-Shanghai-HA (HA2-D116N) were also lower than that of the parent virus rDk/ZJ-Shanghai-HA, despite the fact that there is no significant difference between them (Fig 4C). We confirmed a similar trend between the copy number and infectious titer of virions released from 21E5 cells (Figs 4C, 4D, S4A and S4B). Analysis of the kinetic parameters of viral replication revealed a positive correlation between the viral copy number of released virions and the pH threshold of the HA protein for membrane fusion (S5 Fig).

Whereas we analyzed the effect of HA acid stability on infectivity and replication of the H7N9 prototype [Shanghai (H7N9) or Anhui (H7N9) strains], as well as other H7 viruses that harbor no multiple basic amino acids within the HA cleavage site (low pathotypes in birds), H7N9 strains harboring multiple basic amino acids (high pathotypes in birds) have appeared and propagated since 2016 (the fifth wave) [40]. In addition, several avian strains that harbor multiple basic amino acids in the HA protein were already known to be zoonotic viruses before H7N9 viruses appeared in 2013 [2]. Therefore, to evaluate the effect of HA acid stability on replication of H7 strains harboring multiple basic amino acids (highly pathogenic type), we also generated recombinant virus Dk/ZJ (H7N3) harboring the HA gene of A/Canada/rv504/2004 (H7N3)[Canada (H7N3)], A/Mexico/InDRE7218/2012 (H7N3)[Mexico (H7N3)], or A/Netherlands/219/2003 (H7N7)[Netherland (H7N7)] (denoted here as rDk/ZJ-Canada-HA, and rDk/ZJ-Mexico-HA, and rDk/ZJ-Netherland-HA, respectively), which harbor multiple basic amino acids within the HA cleavage site. We then infected 21E5 cells with these recombinant viruses. As shown in S6A Fig, rDk/ZJ-Mexico-HA and rDk/ZJ-Netherland-HA showed significantly higher infectivity than the parent virus rDk/ZJ (H7N3), whereas the infectivity of rDk/ZJ-Canada-HA was slightly higher than that of the parent virus rDk/ZJ (H7N3) (S6A Fig). The pH threshold for A/Canada/rv504/2004 (H7N3), A/Mexico/InDRE7218/2012 (H7N3), and A/Netherlands/219/2003 (H7N7) was 5.5 (S6B Fig), which is higher than that of parent virus Dk/ZJ (H7N3)(5.375) (Fig 2B and 2C). In addition, we performed structural analysis of HA trimetric models to examine the HA function of three highly pathogenic strains. This structural analysis revealed that the interaction energies between the helix (HA2-75–129) on the stalk and other HA molecules within each highly pathogenic strain were higher than those of parent virus Dk/ZJ (H7N3) (S6C Fig and S1 and S2 Tables). The interaction energies of the inter-helix (HA2-75–129) within each highly pathogenic strain were also higher than those of parent virus Dk/ZJ (H7N3) (S6C Fig and S1 and S2 Tables).

### Prevalence of the HA mutation (HA2-116D) in H7 AIV

The HA amino acid residue (HA2-116D) in zoonotic H7N9 viruses that emerged in 2013 plays an important role in infectivity of human cells; therefore, we investigated the conservation of HA2-116D in H7N9 viruses circulating before 2013 (isolated in 2001–2012) and from 2013 and after (isolated in 2013–2020), along with H7 subtypes other than H7N9. Analysis of HA amino acid sequences obtained from a database revealed that in H7N9 viruses isolated from 2013–2020, HA2-116D is conserved in human strains (100%) and avian strains (98.1%) (Table 1). The data also revealed that HA2-116N in H7N9 viruses isolated from 2001–2012 is conserved in avian strains (100%). However, HA2-116D was rarely found in avian H7N2 strains isolated from 2001–2012 (0.7%) or from 2013–2020 (11.1%), in avian H7N3 strains isolated from 2001–2012 (2.7%), or in avian H7N6 strains isolated from 2001–2012 (2.3%) or from 2013–2020 (40.0%); the mutation (HA2-116D) was not detected at all in the H7N1, H7N4, H7N5, H7N7, and H7N8 subtypes (Table 1).

**Table 1:** Prevalence of the HA2-116 amino acid mutation in the database.

		% of strains harboring the mutation (no. of strains)	
Subtype	Mutation	Human viruses	Avian viruses
H7N1 (2001–2012)	HA2 116N	N.D.	100.0 (26)
	HA2 116D	N.D.	0 (0)
H7N1 (2013–2020)	HA2 116N	N.D.	100.0 (4)
	HA2 116D	N.D.	0 (0)
H7N2 (2001–2012)	HA2 116N	0 (0)	10.1 (14)
	HA2 116S	100.0 (1)	89.2 (124)
	HA2 116D	0 (0)	0.7 (1)
H7N2 (2013–2020)	HA2 116N	N.D.	77.8 (7)
	HA2 116A	N.D.	11.1 (1)
	HA2 116D	N.D.	11.1 (1)
H7N3 (2001–2012)	HA2 116N	100.0 (2)	93.1 (312)
	HA2 116T	0 (0)	4.2 (14)
	HA2 116D	0 (0)	2.7 (9)
H7N3 (2013–2020)	HA2 116N	N.D.	100 (66)
	HA2 116D	N.D.	0 (0)
H7N4 (2001–2012)	HA2 116N	N.D.	100.0 (18)
	HA2 116D	N.D.	0 (0)
H7N4 (2013–2020)	HA2 116N	N.D.	N.D.
	HA2 116D	N.D.	N.D.
H7N5 (2001–2012)	HA2 116N	N.D.	100.0 (1)
	HA2 116D	N.D.	0 (0)
H7N5 (2013–2020)	HA2 116N	N.D.	N.D.
	HA2 116D	N.D.	N.D.
H7N6 (2001–2012)	HA2 116N	N.D.	97.7 (43)
	HA2 116D	N.D.	2.3 (1)
H7N6 (2013–2020)	HA2 116N	N.D.	60.0 (3)
	HA2 116D	N.D.	40.0 (2)
H7N7 (2001–2012)	HA2 116N	100.0 (2)	100.0 (154)
	HA2 116D	0 (0)	0 (0)
H7N7 (2013–2020)	HA2 116N	100 (1)	100 (76)
	HA2 116D	0 (0)	0 (0)
H7N8 (2001–2012)	HA2 116N	N.D.	100.0 (11)
	HA2 116D	N.D.	0 (0)
H7N8 (2013–2020)	HA2 116N	N.D.	N.D.
	HA2 116D	N.D.	N.D.
H7N9 (2001–2012)	HA2 116N	N.D.	100.0 (45)
	HA2 116D	N.D.	0 (0)
H7N9 (2013–2020)	HA2 116N	0 (0)	1.9 (8)
	HA2 116D	100.0 (82)	98.1 (420)

**Fig 5:**
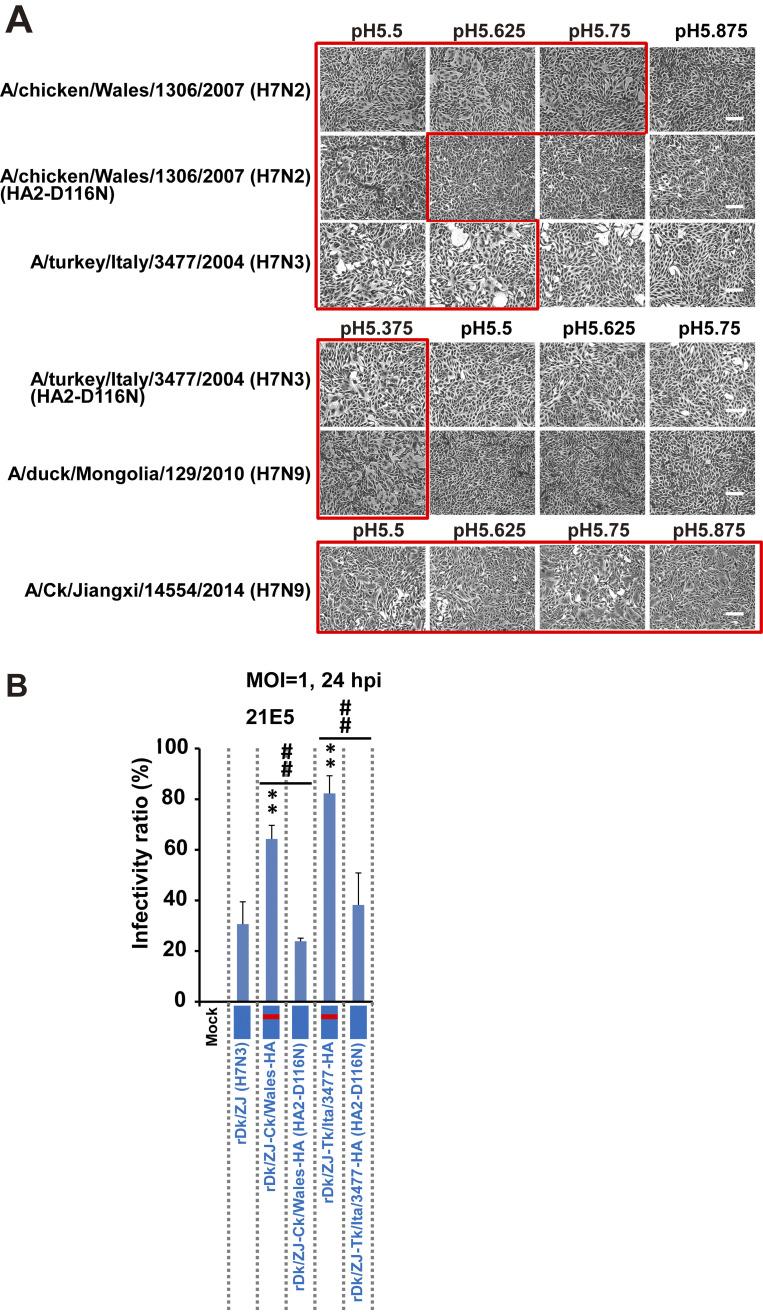
Membrane fusion activity and infectivity of H7 avian isolates harboring HA2-116D. (A) MDCK cells were transfected with the influenza virus HA gene from Ck/Wal (H7N2) and Tk/Ita/3477 (H7N3), or with their respective HA mutants (HA2 D116N), Dk/Mon (H7N9), and Ck/JX (H7N9). Fusion induction over a pH range of 5.375–5.875 was conducted as described in Fig 2. Red squares show polykaryon formation. Micrographs lacking a red square represent a pH above the fusion threshold. The pH threshold was determined as described in “Materials and Methods.” Scale bars, 200 μm. (B) 21E5 cells derived from primary human bronchiolar epithelial cells [see “Materials and Methods”] were infected with rDk/ZJ (H7N3), rDk/ZJ-Ck/Wales-HA, or rDk/ZJ-Tk/Ita/3477-HA, or with their respective HA mutants [rDk/ZJ-Ck/Wales-HA (HA2-D116N) and rDk/ZJ-Tk/Ita/3477-HA (HA2-D116N)]. All cells were infected at an m.o.i. of 1. Viral infectivity was determined by calculating the percentage of antigen-positive cells after immunostaining at 24 h post-infection. Data are expressed as the mean ± S.D. of four independent results. Asterisks indicate that the values for each virus were significantly different from that of rDk/ZJ (H7N3) within the same graph; sharps indicate that the values for each virus harboring a mutation (HA2-D116N) were significantly different from those of each virus without the mutation within the same graph. A p-value < 0.05 (single asterisk or sharps) or < 0.01 (double asterisk or double sharp) was considered significant (one-way ANOVA followed by Tukey’s multiple comparisons post-hoc test). The position of HA2-116D (native residue) in both rDk/ZJ-Ck/Wales-HA and rDk/ZJ-Tk/Ita/3477-HA is shown in red. The mutated amino acid (HA2-D116N) is shown in blue (same as the background color).

To confirm the function of HA2-116D in H7 subtypes other than H7N9, we conducted cell-to-cell fusion assays using MDCK cells expressing the HA genes of A/chicken/Wales/1306/2007(H7N2)(Ck/Wal (H7N2)), A/turkey/Italy/3477/2004 (H7N3)(Tk/Ita/3477 (H7N3)) (both harboring HA2-116D), and their respective back mutants (both harboring HA2-D116N); we also examined the HA gene of H7N9 viruses isolated before 2013 [A/duck/Mongolia/129/2010 (H7N9) [HA2-116N]], and in 2013 and after [A/Ck/Jiangxi/14554/2014 (H7N9)(Ck/JX (H7N9)) [HA2-116D]]. The fusion assay showed that the pH threshold for HA activation of wild-type Ck/Wal (H7N2) and Tk/Ita/3477 (H7N3) was 5.75 and 5.625, respectively, whereas that for their back mutants were 5.5 and 5.375, respectively (Fig 5A). The pH threshold for A/duck/Mongolia/129/2010(H7N9)(Dk/Mon (H7N9)) and Ck/JX (H7N9) was 5.375 and ≥5.875, respectively (Fig 5A). To evaluate the effect of HA2-116D on infectivity of other H7 subtypes, we generated recombinant virus Dk/ZJ (H7N3) harboring the HA gene of Ck/Wal (H7N2) and Tk/Ita/3477 (H7N3) (both harboring HA2-116D, and denoted here as rDk/ZJ-Ck/Wales-HA and rDk/ZJ-Tk/Ita/3477-HA, respectively), as well as their back mutants [denoted here as rDk/ZJ-Ck/Wales-HA (HA2-D116N) and rDk/ZJ-Tk/Ita/3477-HA (HA2-D116N), respectively]. We then infected 21E5 cells with these recombinant viruses. As shown in Fig 5B, rDk/ZJ-Ck/Wales-HA and rDk/ZJ-Tk/Ita/3477-HA showed significantly higher infectivity than the parent virus rDk/ZJ (H7N3). In addition, rDk/ZJ-Ck/Wales-HA and rDk/ZJ-Tk/Ita/3477-HA showed higher infectivity than rDk/ZJ-Ck/Wales-HA (HA2-D116N) or rDk/ZJ-Tk/Ita/3477-HA (HA2-D116N) (Fig 5B). These results suggest that amino acid residue HA2-116D found in H7N2 or H7N3 subtypes also plays a critical role in infection of human airway epithelial cells, similar to that observed for current zoonotic H7N9 viruses harboring HA2-116D.

### The role of amino acid residue HA2-116D in viral infection and replication in primary human airway epithelial cells

**Fig 6:**
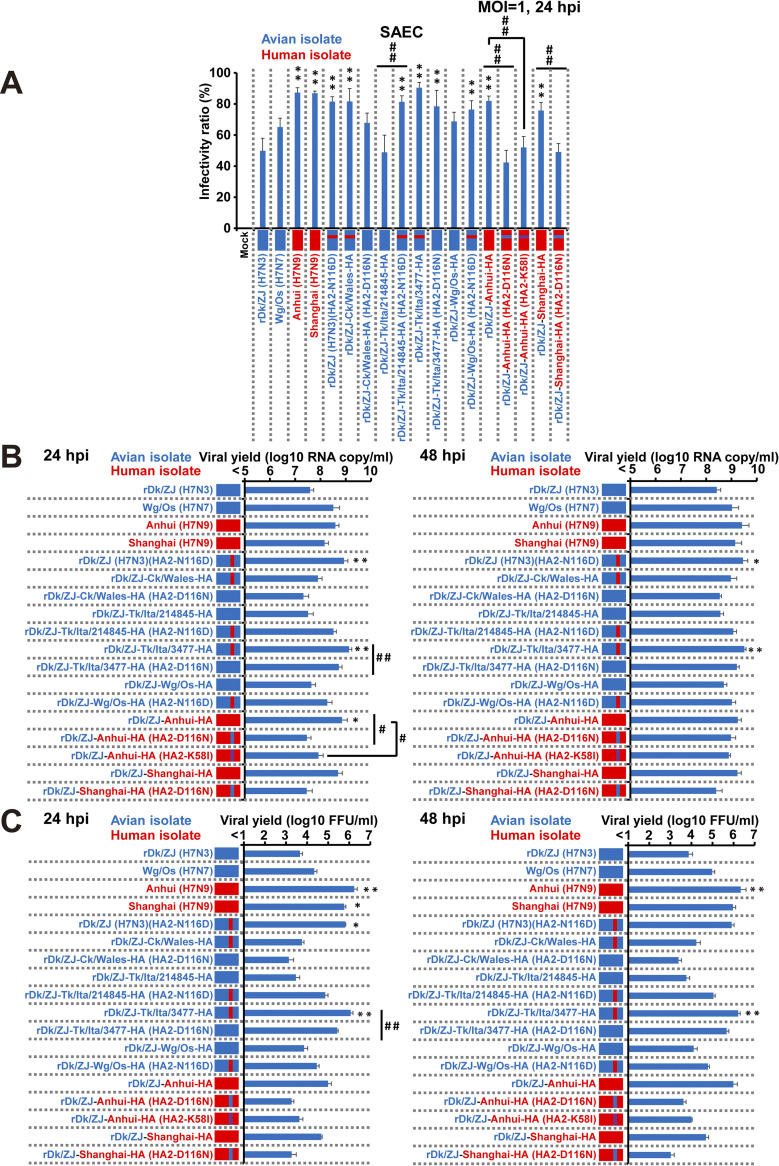
The infectivity and growth kinetics of current H7N9 and other H7 viruses in primary human bronchiolar epithelial cells. (A) Primary human bronchiolar epithelial cells [SAECs, see “Materials and Methods”] were infected with rDk/ZJ (H7N3), Wg/Os (H7N7), Anhui (H7N9), or Shanghai (H7N9), or with recombinant Dk/ZJ (H7N3) [rDk/ZJ-Ck/Wales-HA, rDk/ZJ-Tk/Ita/214845-HA, rDk/ZJ-Tk/Ita/3477-HA, rDk/ZJ-Wg/Os-HA, rDk/ZJ-Anhui-HA, and rDk/ZJ-Shanghai-HA], or with recombinant Dk/ZJ (H7N3) harboring an HA mutation [rDk/ZJ (H7N3) (HA2-N116D), rDk/ZJ-Ck/Wales-HA (HA2-D116N), rDk/ZJ-Tk/Ita/214845-HA (HA2-N116D), rDk/ZJ-Tk/Ita/3477-HA (HA2-D116N), rDk/ZJ-Wg/Os-HA (HA2-N116D), rDk/ZJ-Anhui-HA (HA2-D116N), rDk/ZJ-Anhui-HA (HA2-K58I), and rDk/ZJ-Shanghai-HA (HA2-D116N)]. All cells were infected at an m.o.i. of 1. Viral infectivity was determined by calculating the percentage of antigen-positive cells after immunostaining at 24 h post-infection. Data are expressed as the mean ± S.D. of three independent results. (B) SAECs were infected with rDk/ZJ (H7N3), Wg/Os (H7N7), Anhui (H7N9), or Shanghai (H7N9); with recombinant Dk/ZJ (H7N3) [rDk/ZJ-Ck/Wales-HA, rDk/ZJ-Tk/Ita/214845-HA, rDk/ZJ-Tk/Ita/3477-HA, rDk/ZJ-Wg/Os-HA, rDk/ZJ-Anhui-HA, or rDk/ZJ-Shanghai-HA]; or with recombinant Dk/ZJ (H7N3) harboring an HA mutation [rDk/ZJ (H7N3)(HA2-N116D), rDk/ZJ-Ck/Wales-HA (HA2-D116N), rDk/ZJ-Tk/Ita/214845-HA (HA2-N116D), rDk/ZJ-Tk/Ita/3477-HA (HA2-D116N), rDk/ZJ-Wg/Os-HA (HA2-N116D), Dk/ZJ-Anhui-HA (HA2-D116N), rDk/ZJ-Anhui-HA (HA2-K58I), and rDk/ZJ-Shanghai-HA (HA2-D116N)]. All cells were infected at an m.o.i. of 0.1. The amount of progeny vRNA within the culture supernatants at 24 and 48 h post-infection was determined by measuring virus titers in quantitative real-time PCR assays (the growth curves of released virions at 12, 24, 48, 72, 96, and 120 h post-infection are shown in S8A Fig). (C) SAECs were infected with the same virus used in (B) at an m.o.i. of 0.1. The infectious titer of the released virions within the culture supernatants at 24 and 48 h post-infection was determined in a focus-forming assay (the growth curves based on the infectious virus titer of released virions at 12, 24, 48, 72, 96, and 120 h post-infection are shown in S8B Fig). Data are expressed as the mean ± S.D. of three independent results. Asterisks indicate that the values for each virus were significantly different from that of rDk/ZJ (H7N3) within the same graph (A–C); sharps indicate that the values for each virus with a mutation (HA2-N116D, HA2-D116N, or HA2-K58I) was significantly different from that of each virus without a mutation within the same graph (A–C). A p-value < 0.05 (single asterisk or sharp) or < 0.01 (double asterisk or double sharp) was considered significant (one-way ANOVA followed by Tukey’s multiple comparisons post-hoc test; A–C). The mutated amino acids (HA2-N116D and HA2-D116N) are shown in red (HA2-N116D) and blue (HA2-D116N), respectively. The mutated amino acid HA2-K58I is shown in purple. The position of HA2-116D (native residue) in both rDk/ZJ-Ck/Wales-HA and rDk/ZJ-Tk/Ita/3477-HA is also shown in red. Please note that the mutated amino acid (HA2-116) in rDk/ZJ-Ck/Wales-HA (HA2-D116N) and rDk/ZJ-Tk/Ita/3477-HA (HA2-D116N) is shown in the same color as the background (blue).

Next, we confirmed that the effects of HA2-116D, which destabilizes the HA protein, are also observed in SAECs, which are cultured primary cells. First, we infected SAECs with recombinant Dk/ZJ (H7N3) viruses possessing the H7N9 HA gene [rDk/ZJ-Anhui-HA and rDk/ZJ-Shanghai-HA] or the H7N2/N3/N7 HA gene [rDk/ZJ-Ck/Wales-HA, rDk/ZJ-Tk/Ita/214845-HA, rDk/ZJ-Tk/Ita/3477-HA, and rDk/ZJ-Wg/Os-HA], as well as their recombinant counterparts harboring HA mutations HA2-D116N, HA2-N116D, or HA2-K58I [rDk/ZJ (H7N3)(HA2-N116D), rDk/ZJ-Anhui-HA (HA2-D116N), rDk/ZJ-Anhui-HA (HA2-K58I), rDk/ZJ-Shanghai-HA (HA2-D116N), rDk/ZJ-Ck/Wales-HA (HA2-D116N), rDk/ZJ-Tk/Ita/214845-HA (HA2-N116D), rDk/ZJ-Tk/Ita/3477-HA (HA2-D116N), and rDk/ZJ-Wg/Os-HA (HA2-N116D)]. We also infected cells with the wild-type viruses. As shown in Fig 6A, rDk/ZJ-Ck/Wales-HA, rDk/ZJ-Tk/Ita/3477-HA, rDk/ZJ-Anhui-HA, and rDk/ZJ-Shanghai-HA, which harbor the native HA2-116D, showed significantly higher infectivity than the parental virus rDk/ZJ (H7N3). By contrast, rDk/ZJ-Tk/Ita/214845-HA, which lacks HA2-116D, showed similar infectivity to rDk/ZJ (H7N3) (Fig 6A). In addition, rDk/ZJ(H7N3) (HA2-N116D) and rDk/ZJ-Tk/Ita/214845-HA (HA2-N116D) showed significantly higher infectivity than the parent viruses [rDk/ZJ (H7N3) and rDk/ZJ-Tk/Ita/214845-HA, respectively] harboring HA2-116N (Fig 6A). However, rDk/ZJ-Anhui-HA (HA2-D116N) and rDk/ZJ-Shanghai-HA (HA2-D116N) showed significantly lower infectivity than their parent viruses [rDk/ZJ-Anhui-HA and rDk/ZJ-Shanghai-HA, respectively] harboring HA2-116D. rDk/ZJ-Anhui-HA (HA2-K58I) also showed significantly lower infectivity than the parent virus rDk/ZJ-Anhui-HA harboring HA2-116D. rDk/ZJ-Ck/Wales-HA (HA2-D116N) and rDk/ZJ-Tk/Ita/3477-HA (HA2-D116N) showed lower infectivity than the parent viruses rDk/ZJ-Ck/Wales-HA and rDk/ZJ-Tk/Ita/3477-HA, respectively, harboring HA2-116D (Fig 6A). The enhanced or reduced infectivity mediated by the HA2-116 mutation was observed even in primary airway epithelial cells from different donors, although susceptibility to infection varied between individuals (Figs 6A and S7).

In addition, we used primary SAECs to evaluate the effect of HA acid stability on virus growth. The replication kinetics of Wg/Os (H7N7), Anhui (H7N9), and Shanghai (H7N9) were higher than those of rDk/ZJ (H7N3) (Figs 6B and S8A). The growth curves for Dk/ZJ-Tk/Ita/3477-HA, rDk/ZJ-Anhui-HA, and rDk/ZJ-Shanghai-HA, which harbor the native HA2-116D, were significantly higher than that of the parent virus rDk/ZJ (H7N3) (Figs 6B and S8A). In addition, the growth of recombinant viruses harboring the HA mutation [rDk/ZJ (H7N3) (HA2-N116D)] was significantly greater than that of their parent virus rDk/ZJ (H7N3), which lacks HA2-116D (Figs 6B and S8A). rDk/ZJ-Tk/Ita/214845-HA (HA2-N116D) showed a growth rate similar to that of rDk/ZJ (H7N3) (HA2-N116D), despite there being no significant difference between rDk/ZJ-Tk/Ita/214845-HA viruses with and without HA2-N116D (Fig 6B). The growth rate of rDk/ZJ-Wg/Os-HA (HA2-N116D) was also greater than that of the parent virus rDk/ZJ-Wg/Os-HA (Fig 6B). However, the growth kinetics of recombinant viruses harboring the HA mutation [rDk/ZJ-Anhui-HA (HA2-D116N) and rDk/ZJ-Anhui-HA (HA2-K58I)] were significantly lower than those of the parent virus rDk/ZJ-Anhui-HA (HA2-116D). The growth kinetics of rDk/ZJ-Shanghai-HA (HA2-D116N) were similar to those of rDk/ZJ-Anhui-HA (HA2-D116N), despite there being no significant difference between rDk/ZJ-Shanghai-HA with and without HA2-D116N (Fig 6B). The growth kinetics of rDk/ZJ-Ck/Wales-HA (HA2-D116N) and rDk/ZJ-Tk/Ita/3477-HA (HA2-D116N) were lower than those of the parent viruses rDk/ZJ-Ck/Wales-HA and rDk/ZJ-Tk/Ita/3477-HA (HA2-116D), respectively, despite the fact that there is no significant difference between rDk/ZJ-Ck/Wales-HA (HA2-D116N) and the parent virus rDk/ZJ-Ck/Wales-HA (Fig 6B). We confirmed a similar trend between the copy number and infectious titer of virions released from primary SAECs (Figs 6B, 6C, S8A and S8B).

### The role of HA2-116D as a H7N9 virulence factor in mice

**Fig 7:**
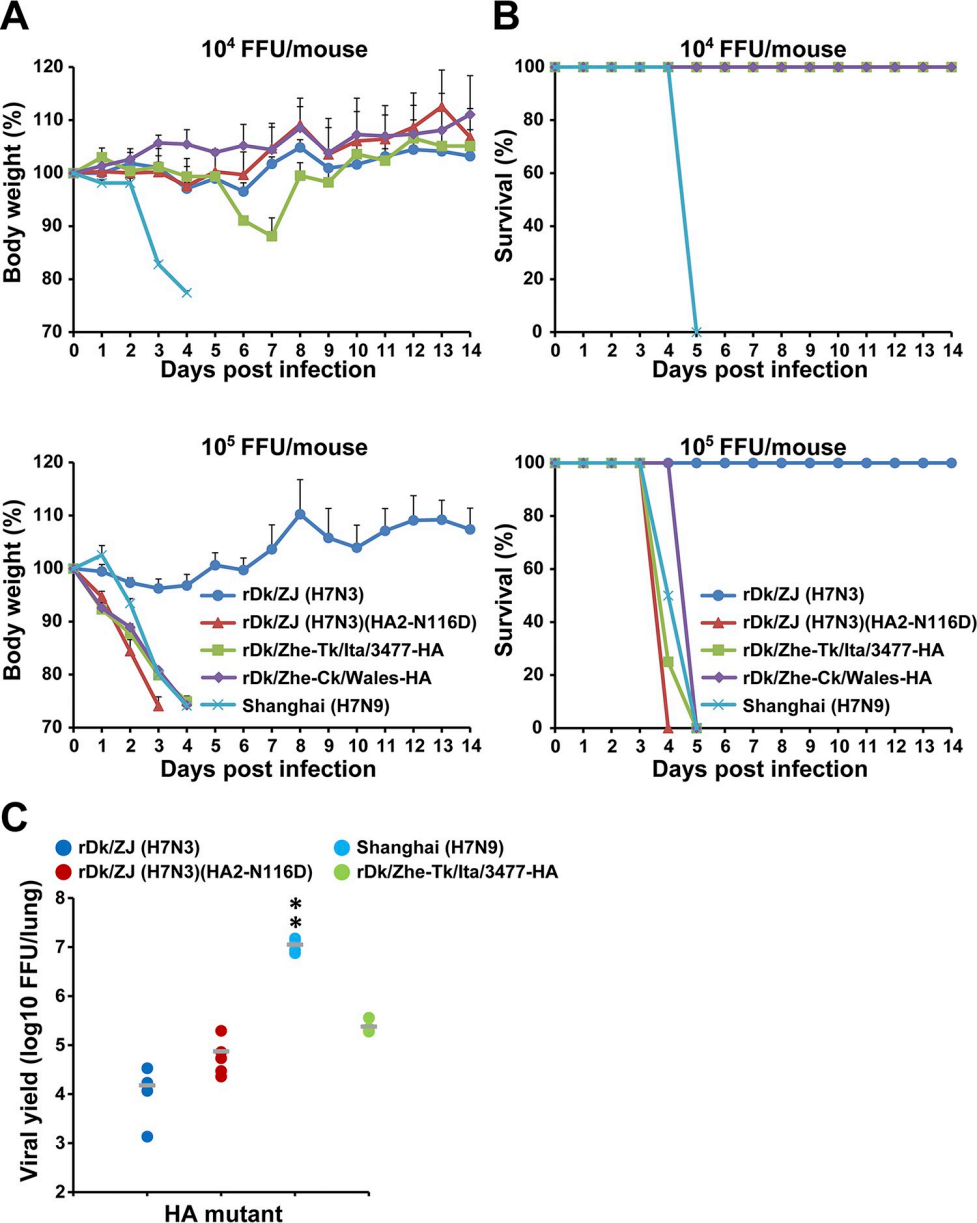
Effect of HA acid stability on mortality and weight loss in mice infected with H7 viruses. Five-week-old BALB/c mice were inoculated intranasally with 10^4^ FFU or 10^5^ FFU of the indicated virus. (A) Body weight of mice (four per group) infected with the indicated viruses was monitored for 14 days post-infection. The percentage change in body weight (mean ± SD) for each group of infected mice is shown. (B) Survival of infected mice (four per group). Mortality rates included mice that were euthanized after they had lost more than 20% of their body weight. (C) Virus titers in the lungs of infected mice (five per group, except Shanghai (H7N9), which was tested in a group of four) infected with 10^5^ FFU of the indicated viruses at 3 days post-infection. Each symbol marks the titer in an individual mouse. The asterisks indicate that the titer of each virus was significantly different from that of rDk/ZJ (H7N3) within the same graph. A p-value < 0.01 (double asterisk) was considered significant (one-way ANOVA followed by Tukey’s multiple comparisons post-hoc test).

Finally, to evaluate the effect of amino acid residue HA2-116D on H7 virus infection *in vivo*, mice were inoculated intranasally with 10^4^ or 10^5^ FFU of wild-type virus, or with recombinant viruses with a different pH threshold for HA activation to induce membrane fusion. The body weight and survival of each inoculated mouse were monitored daily for 14 days. At an inoculation dose of 10^5^ FFU, all viruses [Shanghai (H7N9), rDk/ZJ (H7N3)(HA2-N116D), rDk/ZJ-Ck/Wales-HA, and rDk/ZJ-Tk/Ita/3477-HA] harboring HA2-116D caused severe weight loss; the exception was rDk/ZJ (H7N3), which lacks HA2-116D (Fig 7A). At an inoculation dose of 10^4^ FFU, Shanghai (H7N9) and rDk/ZJ-Tk/Ita/3477-HA caused weight loss, but the other viruses did not. Shanghai (H7N9) caused severe weight loss at an inoculation dose of 10^4^ FFU, whereas rDk/ZJ-Tk/Ita/3477-HA induced only minor weight loss at this dose (Fig 7A). The survival rates of mice infected with these viruses were reflected in the mouse weight results (Fig 7A and 7B).

To measure the virus titer, the lungs of mice inoculated with 10^5^ FFU of each individual virus were collected at 3 days post-infection (dpi). The titers for individual virus were consistent with the clinical signs (Fig 7A, 7B and 7C). The amount of progeny virions in the lungs of mice infected with rDk/ZJ (H7N3) (HA2-N116D), rDk/ZJ-Tk/Ita/3477-HA, or Shanghai (H7N9) was higher than that in the lungs of mice infected with rDk/ZJ (H7N3) (Fig 7C). In particular, the amount of released Shanghai (H7N9) virions was much higher than the amount of released rDk/ZJ (H7N3) virions. These results suggest that HA2-116D in current zoonotic H7N9 viruses plays an important role in not only infectivity of human airway epithelial cells in *vitro*, but also in that of cells in the lungs of a mammalian model.

## Discussion

The main findings of this study were as follows: (i) HA2-116D plays a crucial role in reducing the HA acid stability of current zoonotic H7N9 viruses; (ii) the HA acid stability of H7 viruses plays an important role in infectivity of human airway epithelial cells; (iii) HA2-116D contributes to infection and replication of H7 viruses in human airway epithelial cells; and (vi) HA2-116D serves as a virulence factor for H7 viruses in mice.

As shown by the alignment of the HA amino acid sequences of Shanghai (H7N9) and Dk/ZJ (H7N3), as well as the pH threshold value of mutant viruses in the membrane fusion assay, Shanghai (H7N9) appears to have acquired HA2-116D in its HA protein, which was originally provided by the Dk/ZJ (H7N3); this supports our hypothesis that current zoonotic H7N9 viruses acquired the crucial amino acid that affects pathogenicity after obtaining the HA gene from a non-zoonotic avian virus other than the H7N9 subtype.

The difference in the pH value for HA activation between Dk/ZJ (H7N3) and Shanghai (H7N9) was explained by examining HA trimetric models for these two viruses. HA modeling revealed that HA2-116D, which has an electrostatically negative charge, repulses surrounding amino acids such as D112 (negative charge), E120 (negative charge), and HA2-116D located in the same or in the facing HA monomer, whereas HA2-116N, which is a neutral amino acid with no charge, does not. These notions are evidenced by our finding that the interaction energy of HA2-116D (4.91 kcal/mol) was larger than that of HA2-116N (-0.39 kcal/mol), and that the distance between each HA monomer within the HA trimer of Shanghai (H7N9) was greater than that of rDk/ZJ (H7N3). These results indicate that mutations in the HA stalk region, such as HA2-N116D, facilitate conformational changes in HA, followed by membrane fusion. The property conferred by HA2-116D in H7N9 viruses is associated with those caused by other similar mutations in the HA stalk region, such as HA2-K58I or HA2-D112G [41]. Byrd-Leotis [41] examined the effect of HA2-K58I and HA2-D112G mutations on HA stability. They found that HA2-K58I reduces the pH threshold of activation in avian HA subtypes H1 (ΔpH -0.4), H3 (ΔpH from -0.2 to -0.4), H5 (ΔpH from -0.4 to -0.6), and H7 (ΔpH from -0.4 to -0.5), whereas HA2-D112G increases the pH threshold in subtype H1 (ΔpH +0.2), H3 (ΔpH from +0.3 to +0.4), H5 (ΔpH +0.2), and H7 (ΔpH from +0.3 to +0.4). The parameters, together with our present results, indicate that amino acid mutations that alter the electric charge can drastically change the pH threshold that induces conformational changes in HA.

In general, influenza viruses infect cells via a viral-cell membrane fusion step [42–44], and the pH threshold for activation of the HA-mediated fusion step correlates with the level of viral infection [45,46]. After internalization into host cells via endocytosis, early uncoating and release of the viral genome at higher pH values can lead to rapid exit from the endosome rapidly, allowing viruses to escape transportation to the lysosome and subsequent degradation [45,46]. Some studies [47–49] support this notion. It is reported that serial passage of laboratory influenza virus strains in cultured cells or mouse lung results an increase in the pH threshold for activation of HA-mediated fusion, resulting in more efficient replication in cultured cells, mouse lung cells, or both [47–49]. Thus, the elevated pH threshold (pH 5.6–5.8) identified in the present and other studies [50–53] may be associated with the infectivity of current zoonotic H7N9 viruses.

To evaluate the relationship between the pH threshold for HA activation and host tropism, we conducted further analyses to determine the pH threshold for activation of the HA protein belonging to some H7 strains of zoonotic or non-zoonotic viruses. We found that even for HA proteins derived from multiple viral strains, zoonotic viruses such as Anhui (H7N9) and Shanghai (H7N9) showed membrane fusion activity at pH values (pH 5.625–5.75) higher than those of non-zoonotic H7 viruses (pH 5.375–5.5), the exception being A/dk/Hong Kong/293/1978 (H7N2)[Dk/Hk (H7N2)] (pH 5.625). Because endosomal pH values are inherently different in cultured cells derived from different species/tissues [44,54–65], or even in cells from the same respiratory region of one person [36], zoonotic H7 viruses such as Anhui (H7N9) and Shanghai (H7N9) may exhibit a diverse range of host tropisms (avian and human) due to the pH-labile HA, which can deal with a variety of endosomal pHs in different types of cells. This hypothesis led us to conduct infectious experiments using H7 viruses with HA proteins showing differing acid stability.

The infectious assay using 21E5 cells (an SAEC-T cell clone immortalized from primary human bronchiolar cells) expressing both avian and human sialic acid receptors [36], showed that the HA protein of current zoonotic H7N9 viruses, and the specific amino acid HA2-116D, play an important role in infection by increasing the pH threshold for HA activation. The results obtained herein agree with those in our previous report on the relationship between the pH threshold for activation and HA-mediated fusion and viral infection [35,36]. The results of these studies demonstrated that current zoonotic H5N1 viruses show strong infectivity in a larger number of cell clones (human SAEC-Ts), and revealed efficient viral propagation and enhanced infectivity of human tracheal cell clones (HTEpC-Ts; which are also immortalized from primary human tracheal cells). In a previous report [36], we also determined the pH threshold for activation of HA-mediated fusion of H5 viruses. The pH value of HA activation for zoonotic H5N1 viruses was 5.625–5.75, whereas that for non-zoonotic AIV such as H2N2, H4N5, H5N3, H5N9, H6N2, H7N7, and H8N4 subtypes ranged from pH 5.125–5.5 [36]. Zoonotic H5N1 strains (wild-type) and recombinant H5N3 strains harboring the zoonotic H5N1 HA protein described above showed higher infectivity than the non-zoonotic AIV described above (H2–H8 subtypes), as well as the parent non-zoonotic H5N3 strain, in SAEC-T clones [36]. These data, together with our present results regarding the infectivity of H7 viruses, lead us to conjecture that the pH threshold for HA-mediated membrane fusion robustly affects the infectivity and/or propagation of current zoonotic AIV in human airway epithelial cells, and that HA2-116D has a crucial effect on the pH threshold for HA activation.

The infectivity of H7 viruses observed in 21E5 cells was closely reflected in the viral replication kinetics of zoonotic or non-zoonotic H7 viruses, as shown by the large amount of released viral particles of current zoonotic H7N9 viruses and recombinant Dk/ZJ (H7N3) viruses harboring HA2-116D. These results provide evidence that the membrane fusion activity conferred by amino acid HA2-116D plays a key role not only in viral infectivity over a relatively short time period, but also in viral replication kinetics over a longer time period.

The recombinant H7 viruses harboring the HA gene of Canada (H7N3), Mexico (H7N3), or Netherland (H7N7), which carry multiple basic amino acids (i.e., the high pathotype in birds), showed higher replication kinetics than rDk/ZJ (H7N3), as well as moderately higher pH sensitivity (pH 5.5) than that of Dk/ZJ (H7N3) (pH 5.375). Because rDk/ZJ (H7N3) and the three recombinant H7 viruses (high pathotypes), which do and do not require supplemental proteases for multiple infection, respectively, were cultured under the same conditions in medium containing trypsin, the HA protein with increased pH sensitivity is thought to be the main contributor to viral replication, although multiple basic amino acid sequences within HA may support viral replication to a certain degree. The difference in the pH sensitivity for HA activation between Dk/ZJ (H7N3) and recombinant H7 viruses harboring the HA gene of Canada (H7N3), Mexico (H7N3), or Netherland (H7N7) can be explained by examining HA trimetric models for these four viruses. As shown by the higher interaction energies between helix (HA2-75–129) on the stalk and the adjacent HA molecule or other helix (HA2-75–129) on the opposite site within Canada (H7N3), Mexico (H7N3), and Netherland (H7N7), these three H7 strains (high pathotype) are structurally unstable and may support higher viral replication in infected cells. Interestingly, the HA protein of Canada (H7N3), Mexico (H7N3), and Netherland (H7N7) lack HA2-116D. This leads us to surmise that the structure of the HA protein of Canada (H7N3), Mexico (H7N3), and Netherland (H7N7) is relatively unstable in the absence of HA2-116D.

Because the infectivity and replication of current zoonotic H7N9 viruses is governed by HA2-116D, this amino acid has extreme importance with respect to influenza viruses. Murakami et al [65] conducted serial passage of seasonal influenza virus in mammalian cells to obtain cell-adapted virus, and demonstrated the role of amino acid mutations acquired during cell passage. After the 11th passage of A/Puerto Rico/8/34 (PR8) (H1N1), they identified three mutations (HA2-N117D, NA N255Y, and PB2 D740N), of which HA2-N117D corresponds to the same position we identified in the HA protein of Shanghai (H7N9). When they examined the replication kinetics of A/Puerto Rico/8/34 (PR8) (H1N1) with or without HA2-N117D, they found that the titer of A/Puerto Rico/8/34 (PR8) (H1N1) with HA2-N117D was higher (by approximately 4–5 log) at 48 and 60 hpi than that of the wild-type virus. Although HA2-N117D was obtained by serial passage, the results suggest that HA2-117D (corresponding to HA2-116D in our study) is critical for viral replication in mammalian cells.

Based on the importance of HA2-116D, we examined the prevalence of this amino acid in the HA protein of H7 viruses (H7N1–H7N9) from the database, i.e., NCBI Influenza Virus Resource [66]. The database search revealed that HA2-116D is highly conserved among H7N9 viruses isolated since 2013 (both avian and human isolates), but not in H7N9 viruses isolated before 2013. This further strengthens our hypothesis that current zoonotic H7N9, which emerged in 2013, acquired crucial amino acids that expand host tropism beyond that of the ancestor virus, which is non-zoonotic and was circulating before 2013. The database research also revealed the prevalence of HA2-116D in H7 subtypes other than H7N9. Although less common, HA2-116D was found in H7N2, H7N3, and H7N6 (small sample sizes mean that detailed information about the prevalence of HA2-116D in H7N6 is unavailable, but it is remarkable that the high prevalence of HA2-116D was observed in H7N6, as well as H7N2, in 2013–2020), suggesting that other H7 subtypes harboring HA2-116D have the potential to develop expanded tropism to both avian and human hosts. This hypothesis led us to conduct an infectious assay using recombinant virus harboring the HA protein of Ck/Wal (H7N2) or Tk/Ita/3477 (H7N3), which harbor HA2-116D, and have an elevated pH activation threshold for HA (pH 5.75 and pH 5.625, respectively). These recombinant viruses showed higher infectivity of 21E5 cells than their respective back mutants harboring HA2-D116N. Based on these results, it is plausible that other subtypes such as H7N2/H7N3 could transmit to humans from birds, as is the case with current zoonotic H7N9 viruses. Decades ago, outbreaks of avian H7N2 subtype occurred in Wales [67,68]. In this outbreak, several humans who had contact with infected birds developed flu-like symptoms. In addition, some of them were PCR-positive for H7 influenza virus, and harbored a H7 HA gene identical to that of the avian strain that was circulating simultaneously (A/Chicken/Wales/2007) [68]. These results suggest that Ck/Wal (H7N2) isolated from birds has the potential to infect humans, and that Tk/Ita/3477 (H7N3) harboring HA2-116D also has the potential to infect mammalian hosts; however, in both cases, other amino acid mutations such as PB2 E627K and PB2 D701N [69–76], which are known to change host tropism from bird to human, may also be required for mammalian adaptation to facilitate infection of a human host.

Given that HA2-116D can affect the behavior of H7 viruses markedly, we examined whether the HA acid stability governed by HA2-116D is linked directly to the pathogenesis of H7 viruses in mammalian hosts. The result obtained in 21E5 cells was confirmed in an infectious assay using primary human SAECs from different donors, although susceptibility to each H7 virus differed from donor to donor. Alterations brought about by amino acid mutations HA2-N116D or HA2-D116N were not as drastic as those observed in 21E5 cells; in particular, the difference between rDk/ZJ-Ck/Wales-HA and its back mutant (HA2-D116N), or between rDk/ZJ-Tk/Ita/3477-HA and its back mutant (HA2-D116N), was not so great. These result can be explained by our previous studies of the endosomal pH environment in human airway epithelial cells. According to our previous experiments, the human bronchiole comprises two different types of cells in which the endosome is acidic or mildly acidic. Because 21E5 cells are categorized as mildly acidic (the pH of endosome is >5.6) [36], the difference in viral infectivity induced by HA acid stability may be more apparent than in primary SAECs, which have a more heterogeneous endosomal pH (acidic to mildly acidic). In addition, the present results suggest that susceptibility to virus infection differs from human to human because the degree of endosomal pH heterogeneity (acidic to mildly acidic) in humans may vary according to ethnicity, gender, and age.

Finally, we confirmed that the expanded host tropism of H7N9 or other H7 viruses harboring HA2-116D was reflected in the pathogenic effects in a mammalian host. The results obtained in primary human SAECs agreed with those from the mouse experiment. In other words, current H7N9 or other recombinant H7 viruses harboring HA2-116D caused more weight loss and reduced survival mice compared with rDk/ZJ (H7N3) lacking HA2-116D. In addition, the amount of infectious virions derived from H7N9 or other H7 viruses harboring HA2-116D was higher than those derived from rDk/ZJ (H7N3) lacking HA2-116D, although the virus titer of rDk/ZJ (H7N3) (HA2-116D) in infected mice did not reach that of Shanghai (H7N9) (wild-type). These results suggest that the expanded host tropism of H7N9 or other H7 viruses harboring HA2-116D is linked directly to pathogenicity in a mammalian host. One limitation of this study is that we did not determine whether another factor acts alongside HA2-116D to affect replication of H7N9 viruses in mouse lung tissue. Further studies are required to assess amino acid mutations found in viral proteins other than HA of H7N9 viruses, e.g., polymerase (which is important for adaptation to a mammalian host) [69–76].

Recent reports show that human-to-human transmission of AIV requires that, together with recognition of human receptors, the HA protein must have a certain level of acid stability [77,78]; this is because such viruses have to replicate in the upper respiratory region (e.g., nasal mucosa) in which the pH is approximately 5.5–6.9 [79,80]. In the absence of a target membrane vesicle, conformational changes in HA result in irreversible virus inactivation. Therefore, AIV with an increased pH for HA activation are not ideally suited to replication in the upper respiratory region; however, they may be more suited to the lower respiratory tract (e.g., bronchioles or alveoli), in which the environment is mildly acidic [81]. This may be the reason why H5N1 viruses with an increased pH for HA activation [36,37,82–84] have not yet acquired the ability for human-to-human transmission, but still demonstrate high mortality caused by replication in the lower respiratory tract. This notion corresponds with our results showing that H7N9 viruses with an increased pH of HA activation show efficient replication in both SAECs (human bronchiolar cells) and mouse lung. The appropriate level of HA acid stability required for viral replication is likely to be determined by a balance between having adequate stability to stand up to extracellular environmental pH conditions and sufficient pH sensitivity to enable viral uncoating within endosomes [45]. However, it is difficult to define what is “appropriate” for AIV. Increasing the acid stabilization of the HA protein (i.e., a HA protein that has a lower pH threshold for activation) could allow AIV to replicate efficiently in the upper respiratory tract (in which the surface is acidic) [79,80]; such viruses could evolve into human-adapted viruses that could undergo human-to-human transmission. By contrast, AIV with an acid-stable HA protein seem to have to pay for this through poor infectivity and lower propagation, which lead to attenuation of pathogenicity. The notion that viral properties are governed by HA acid stability is supported by a report by Zaraket et al [54], who showed that A/Vietnam/1203/2004 (H5N1) (VN1203) and its mutant HA2-K58I, the HA proteins of which have a high (pH 6.0) and low pH threshold (pH 5.5) for activation, respectively, behaved differently when used to infect ferrets. The mutant virus (HA2-K58I) replicated more efficiently than the wild-type in the nasal cavity of infected ferrets at 1–3 days post-infection. However, the lungs contained more VN1203 particles than HA2-K58I particles [54]. Weight loss in ferrets inoculated with VN1203 was more severe than that of ferrets inoculated with the mutant virus (HA2-K58I), although the difference between the two groups was not significant. Further studies are required to reveal the viral characteristics governed by HA acid stability.

Recently, researchers reported the relationship between the pH threshold for HA activation and the transmissibility of H7N9 viruses in humans. The first-wave H7N9 viruses, some of which are the same as we used in this study, underwent airborne transmission [85,86] in ferrets (to a limited extent) in addition to contact-mediated transmission [52]. By contrast, ferret-adapted H7N9 virus harboring mutations that increase human receptor binding and/or alter the pH threshold for HA activation (from 5.6 to 5.8) showed lower efficiency for airborne transmission [51]. These reports led us to surmise that AIV need a certain level of HA acid stability for efficient airborne transmission among human hosts. In the third-wave, one H7N9 viral strain in which the HA protein had become relatively acid-stable (pH 5.6 for HA activation) showed limited airborne transmission among ferrets (the level was similar to that of the first-wave H7N9 virus with an acid-labile HA protein (pH 5.8 for HA activation)) [53]. In the fifth-wave, some H7N9 strains in which the HA proteins are more acid-stable (pH 5.4 for HA activation) also showed limited airborne transmission among ferrets (at the same level as H7N9 viruses with an acid labile HA protein (pH 5.7 for HA activation) isolated during a similar time period), but were more pathogenic, possibly due to the multi-basic amino acid sequence at the HA cleavage site rather than HA stability [40].

In conclusion, while current zoonotic H7N9 viruses are still unlikely to achieve efficient human-to-human transmission via the airborne route, the virus can be transmitted to humans from an avian host. In the case of bird-to-human transmission, viral particles that bypass the acidic nasal cavity will propagate efficiently in the mildly acidic lower respiratory tract due to the elevated pH threshold for activation of the HA protein, which can lead to high mortality rates. In future, novel zoonotic avian viruses that achieve avian-to-human transmission may emerge from non-zoonotic H7 viruses by acquiring mutations that lead to loss of HA acid stability, as is the case with current zoonotic H7N9 viruses. By contrast, zoonotic H7N9 viruses could also evolve to achieve human-to-human transmission after acquisition of mutations that increase their affinity for human receptors, as well as mutations that affect HA stability; they may also acquire mutations that alter polymerase and/or other viral activities. In addition, an intake of novel viral gene(s) from different subtypes through genetic reassortment could accelerate emergence of such human-adapted viruses because genetic reassortment has higher potential to markedly alter viral characteristics.

However, despite virus evolution, a more acid-stable HA could reduce replication in the lower respiratory tract, possibly weakening pathogenesis. During surveillance of AIV, it is important to pay attention to changes in HA acid stability as well as receptor specificity to monitor the possible appearance of both novel zoonotic viruses and human-adapted viruses that can achieve human-to-human transmission.

## Materials and methods

### Ethics statement

Primary human bronchiolar epithelial cells, obtained from lonza Corp. (Walkersville, MD) as described in *“*Viruses and Cells”, were isolated from donated human tissue after obtaining permission for their use in research applications through informed consent or legal authorization (written informed consent was obtained). All experiments using recombinant DNA were conducted under the relevant Japanese laws and were approved by the Biological Safety Committee of Kyoto Prefectural University of Medicine (approval numbers 2021–161; 2021–167; 2022–108) after risk assessments were conducted by the Living Modified Organisms Committee of Kyoto Prefectural University of Medicine, and (when required) by the Ministry of Education, Culture, Sports, Science, and Technology of Japan. All animal experiments were conducted in compliance with Japanese legislation (Act on Welfare and Management of Animals, 1973, revised in 2012), and with guidelines under the jurisdiction of the Ministry of Education, Culture, Sports, Science and Technology, Japan (Fundamental Guidelines for Proper Conduct of Animal Experiment and Related Activities in Academic Research Institutions, 2006). Animal care, housing, feeding, sampling, observation, and environment enrichment were performed in accordance with these guidelines. The protocols for the animal experiments were approved by the Animal Experiment Committee of the Kyoto Prefectural University of Medicine (approval number M2022-259).

### Database search

A database search was undertaken as described previously [37,87]. Briefly, the published sequences of HA amino acids from influenza A virus subtype H7N1-N9 strains isolated from both avian and human hosts from 2001 to 2020, were obtained from the NCBI Influenza Virus Resource [66]. These HA sequences were aligned using the MAFFT program [88]. Variations in the position of amino acid HA2-116 among H7N1-N9 viruses isolated from humans and birds during 2001–2012 and during 2013–2020 were calculated and compared.

### Biocontainment

All experiments involving live avian influenza viruses were conducted at Kyoto Prefectural University of Medicine under (Animal) Biosafety Level 3 (ABSL3) conditions (as approved by the Ministry of Agriculture, Forestry, and Fisheries, Japan). The ABSL3 facility of Kyoto Prefectural University of Medicine consists of negative-pressure laboratories in which all experimental work is carried out in class 2 biosafety cabinets, which also operate under negative pressure. Air exhausted from the biosafety cabinets is filtered by High Efficiency Particulate Air (HEPA) filters, and then leaves the facility via a second set of HEPA filters. Also, users must check the air pressure when entering and when inside the ABSL3 facility. The BSL3 has a dedicated electrical generator in the event of power loss. Only authorized personal that have received appropriate training can access the ABSL3 facility. All personnel working at the ABSL3 facility wear a full Tyvek suit, FFP3 facemasks, and multiple pairs of gloves. The facility is secured by procedures recognized as appropriate by the institutional biosafety officers at Kyoto Prefectural University of Medicine, and by inspectors of the Japan government. Antiviral drugs are available immediately to further mitigate risks in the event of an incident.

### Viruses and cells

Anhui (H7N9) and Shanghai (H7N9) were kindly provided by Dr. Shu Yuelong (WHO Collaborating Center for Reference and Research on Influenza, Chinese Center for Disease Control and Prevention, Beijing, China), through the National Institute of Infectious Diseases, Japan. A Standard Material Transfer Agreement 2 (SMTA2) with the WHO was arranged to allow experiments to be conducted with H7N9 viruses, which are classed as Pandemic Influenza Preparedness biological material. Dk/Hk (H7N2) and Wg/Os (H7N7) were a kind gift from Yoshinobu Okuno (Kanonji Institute, the Research Foundation for Microbial Diseases of Osaka University, Kagawa, Japan). Wg/Os (H7N7) was described previously [36]. Dk/ZJ (H7N3) and recombinant Dk/ZJ (H7N3) harboring the HA gene of other strains of H7 viruses were generated by reverse genetics, as described below. These influenza virus strains were grown in 9–10-day-old embryonated chicken eggs. For subsequent experiments, allantoic fluid collected from the eggs was precleared by centrifugation at 2,380 × g for 20 min, followed by passage through 0.45 μm filters. To purify and concentrate the viruses, the filtered allantoic fluid was centrifuged (112,500 × g for 2.5 h) on PBS (without calcium/magnesium) [PBS (-)] containing 20% sucrose (w/v). The resulting pellets were suspended in PBS (-), and the virus titer was determined in focus-forming assays using MDCK cells (results are presented as the number of focus-forming units (FFU)/mL) [89]. The details are described in the subsection “Focus-forming assay to measure infectious titers”. MDCK cells were purchased from the Riken BioResource Center Cell Bank (Ibaragi, Japan). Primary human bronchiolar epithelial cells [small airway epithelial cells (SAECs)] were purchased from Lonza Corp. (Walkersville, MD). Human SAEC-T cells (named 21E5 cells), which were immortalized by SV40 large T-antigen from primary SAECs, have been described previously [36].

### Focus-forming assay to measure infectious titers

MDCK cells in 96 well plates were washed twice with PBS (supplemented with calcium/magnesium) (PBS (+)) and then inoculated for 1 h at 37*°C* with sample fluid containing virions (serially diluted 10-fold). After that, the virus inoculum was removed, and the cells were washed twice with PBS (+) and overlaid with 1% methylcellulose in minimum essential medium supplemented with 0.2% bovine serum albumin (Merck, Darmstadt, Germany) and standard antibiotics (penicillin (100 units/mL), streptomycin (100 μg/mL), and amphotericin B (250 ng/mL)). At 16 h post-infection, and after removal of the medium containing methylcellulose, the cells were washed three times with PBS (+) and then fixed for over 40–60 min at room temperature with PBS (-) containing 4% paraformaldehyde/0.1% Triton X-100. After removing the PBS (+) containing paraformaldehyde and washing three times with PBS (-), the cells were stained as described in “Immunofluorescence Analysis” to detect viral antigens. The titers of individual samples (presented as FFU/mL) were determined by manually counting the number of fluorescent foci in the entire well under a fluorescence microscope fitted with filters to detect an Alexa Fluor 488-conjugated secondary antibody (see also “Immunofluorescence Analysis”).

### Reagents

MDCK cells were cultured in minimum essential medium (Merck) supplemented with 10% fetal bovine serum (FBS) and standard antibiotics [(penicillin (100 units/mL), streptomycin (100 μg/mL), and amphotericin B (250 ng/mL)]. Primary SAECs were cultured in Small Airway Epithelial Cell Growth Medium (SAGM, Lonza) according to the manufacturer’s instructions. 21E5 cells, an SAEC-T clone, were cultured in D/M medium (DMM), which is based on Dulbecco’s modified Eagle’s medium (DMEM), and MCDB153 (1:1), supplemented with growth factors [bovine pituitary extract, hydrocortisone, epidermal growth factor, epinephrine, transferrin, insulin, triiodothyronine, retinoic acid, and cholera toxin], as described previously [36], together with 5% FBS and antibiotics (penicillin (100 units/ml), streptomycin (100 μg/ml), and amphotericin B (250 ng/ml)). Primary SAECs were also cultured in DMM for the virus infectious assay and prior to use in the infectious assay.

### Plasmid construction

Viral RNA was isolated using a QIAamp Viral RNA Mini Kit (Qiagen, Hilden, Germany), and cDNA was synthesized using random hexamers. The full-length HA sequences of Dk/Hk (H7N2), Wg/Os (H7N7), Anhui (H7N9), and Shanghai (H7N9) were constructed by PCR of cDNA. The HA sequence of Ck/Wal (H7N2)(EF675618), Canada (H7N3)(CY015006), and A/mallard/Alberta/243/2006 (H7N3)(Mal/Alb(H7N3))(CY185761) was synthesized artificially (Eurofins Genomics, Tokyo, Japan). The NA (JQ906584), PB1 (JQ906568), and PB2 (JQ906564) sequences of Dk/ZJ (H7N3) were synthesized by Eurofins Genomics. Other segments [HA (JQ906576), M (JQ906588), NS (JQ906592), PA (JQ906572), and NP (JQ906580)] of Dk/ZJ (H7N3)] were obtained by site-directed mutagenesis of the PCR product obtained from the cDNA of the H7 viruses described above, or from the cDNA of A/Duck/Hong Kong/820/80 (H5N3) [36]. The HA sequences of A/chicken/Italy/1279/1999 (H7N1)(Ck/Ita (H7N1)(CY099597); Tk/Ita/214845 (H7N3)(AY586408); Tk/Ita/3477 (H7N3)(CY028684); Mexico (H7N3)(CY125728); Netherland (H7N7)(AY338459); Dk/Mon (H7N9) (AB828686); and Ck/JX (H7N9)(KP418030) were constructed in the same way.

The eight-segment sequence of Dk/ZJ (H7N3), and the HA genome of Ck/Wal (H7N2), Tk/Ita/214845 (H7N3), Tk/Ita/3477 (H7N3), Canada (H7N3), Mexico (H7N3), Wg/Os (H7N7), Netherland (H7N7), Anhui (H7N9), and Shanghai (H7N9), were cloned into the transcription plasmid pPOLI [90]. Non-coding regions of the HA genome of Ck/Wal (H7N2) were derived from the H7N1 strain A/mallard duck/Netherlands/60/2008(H7N1)(KX978337), which has a gene sequence that shares a higher degree of homology with the HA sequence of Ck/Wal (H7N2). The HA open reading frame sequences from Ck/Ita (H7N1), Dk/Hk (H7N2), Ck/Wal (H7N2), Dk/ZJ (H7N3), Tk/Ita/214845 (H7N3), Tk/Ita/3477 (H7N3), Canada (H7N3), Mal/Alb(H7N3), Mexico (H7N3), Wg/Os (H7N7), Netherland (H7N7), Dk/Mon (H7N9), Anhui (H7N9), Shanghai (H7N9), and Ck/JX (H7N9) were cloned into pCAGGS protein expression plasmids [91].

### Generation of recombinant H7N3 viruses

Recombinant viruses were generated using a previously described reverse genetics system [90–92], with slight modifications. Briefly, the pPOLI plasmid containing seven genomes of Dk/ZJ (H7N3) and each of the H7 HA genomes described above, was transfected together with pCAGGS expression plasmids [91] harboring WSNPA, PB1, PB2, and NP into 293T cells co-cultured with MDCK cells at a ratio of 7:3. Next, 5 μg/ml of acetylated trypsin (Merck) was added to the plates at 1 and 4 days post-transfection. At 7 days post-transfection, the culture supernatants were collected and injected into 9-day-old chicken eggs. The allantoic fluid was collected at 3 days post-injection. All recombinant Dk/ZJ (H7N3) viruses were confirmed by sequencing.

### Viral infection of cells

21E5 cells (an SAEC-T clone) and primary SAECs were seeded in 96 well plates (2.0 *×* 10^4^ cells/well to 3.0 *×* 10^4^ cells/well), cultured, washed twice with PBS (+), and infected with viruses at a multiplicity of infection (m.o.i.) of 1. The m.o.i. was based on the cell number and the titer of the viruses used (the virus titer was determined in focus-forming assays on MDCK cells, see “Focus-forming assay to measure infectious titers”). After two washes, the cells were incubated at 37*°C* for 1 h with virus contained in PBS (+). The viral solution was then removed and the cells were washed twice with PBS (+). The 21E5 cells and primary SAEC were cultured in DMM containing 5% FBS.

### Immunofluorescence analysis

At 24 h post-infection with the virus, cells were fixed for over 40–60 min at room temperature with PBS (-) containing 4% paraformaldehyde/0.1% Triton X-100, and washed three times with PBS (-). Viral antigens were detected by staining the infected cells with a rabbit polyclonal antibody specific for A/duck/HongKong/342/78 (H5N2) (1:1000 dilution in PBS (-) containing 1% bovine serum albumin), which recognizes the NP and M1 proteins of both avian and human influenza virus strains. After removal and three washes with PBS (-), the primary antibody bound to viral proteins was detected by an Alexa Fluor 488-conjugated secondary antibody (Thermo Fisher Scientific, Waltham, MA, USA) [1:500 dilution in PBS (-)]. Cell nuclei were counterstained with Hoechst 33342 (Merck). To determine the percentage of 21E5 cells or SAECs infected with the virus (expressed as “% infectivity” in the figures), an IN Cell Analyzer 2200 (Cytiva, Chicago, IL, USA) was used as described previously [35]. In brief, the IN Cell Analyzer 2200 was used to image cells, and the number of antigen-positive cells and cell nuclei within a single field were counted (the photographs comprise 16 different visual fields within the same well; all of the photographs obtained for each experiment using individual viral strains were analyzed at the same time by IN Cell Developer Toolbox software (Cytiva). The percentage of virus-infected cells within the 16-image field was calculated by dividing the number of antigen-positive cells by the total number of cell nuclei (*×*100). The number of cells per test well was >1000 (m.o.i. = 1) (the number of countable cells decreased in line with the strength of the cytopathic effects). Results are expressed as the mean ± S.D. of at least three independent wells.

### Cell fusion assay

MDCK cells (cultured to 90% confluency in 24-well plates) were transfected with pCAGGS plasmids encoding the HA gene sequences of different H7 virus strains using Lipofectamine 2000 (Thermo Fisher Scientific). At 24 h post-transfection, cells were washed twice with PBS (+) and cultured for 4–8 h at 37°C in DMEM/F-12 medium containing trypsin (2.5 μg/ml). The cells were washed twice with PBS, followed by treatment with fusion buffers at different pH (PBS adjusted to pH 5.0, 5.125, 5.25, 5.375, 5.5, 5.625, 5.75, 5.875, or 6.0). Cells were incubated with each fusion buffer for 5 min and then returned to DMEM/F-12 medium containing trypsin (2.5 μg/ml in the medium) for 2–3 h at 37°C. Cells were then fixed overnight with PBS (+) containing 0.5% (v/v) glutaraldehyde and stained with 0.025% (w/v) crystal violet. Then, fusion images were taken. The pH threshold was defined as the highest pH value at which polykaryon formation was observed. Fused cells containing more than five nuclei were regarded as polykaryon [36,93].

### HA modeling of H7 virus mutants

The crystal structure of the hemagglutinin trimer of influenza virus Shanghai (H7N9)(PDB ID: 4LN3) was used as a template for homology modeling of Dk/ZJ (H7N3) by the Molecular Operating Environment (MOE, http://www.chemcom.com). Structure preparation and optimization were performed using MOE structure preparation tools (with default parameters). After homology modeling, the interaction energy and distance between HA2-116N or HA2-116D and the other residues in the trimers was calculated.

### Production of progeny viral particles in human bronchiolar epithelial cells

After two washes, 21E5 cells (a clone of SAEC-T) or primary SAECs were infected (in triplicate) with viruses at an m.o.i. of 0.1. The cells were then incubated at 37*°C* for 1 h with virus suspended in PBS (+). The viral solution was removed, and the cells were washed twice with PBS (+). The 21E5 cells or primary SAECs were cultured at 37*°C* in DMM containing 0.2% bovine serum albumin and trypsin [2 μg for 21E5 cells or 1 μg/ml for primary SAECs]. At the indicated times post-infection, the number of viral RNA (vRNA) copies in the cell culture supernatants of 21E5 cells or SAECs was determined by quantitative real-time RT-PCR.

### Quantitative real-time RT-PCR

Progeny vRNA was extracted from the culture supernatant of infected cells using a QIAamp Viral RNA Mini Kit (Qiagen, Hilden, Germany) and subjected to quantitative real-time RT-PCR using the THUNDERBIRD Probe One-step qRT-PCR Kit (Toyobo, Osaka, Japan), a TaqMan probe, and primers targeting the M gene [87]. The primer sequences were as follows: 5’-CCMAGGTCGAAACGTAYGTTCTCTCTATC-3’ (forward) and 5’-TGACAGRATYGGTCTTGTCTTTAGCCAYTCCA-3’ (reverse). A TaqMan probe, which is an oligonucleotide with a fluorescent reporter dye attached to the 5’ end and a non-fluorescent quencher (NFQ) attached to the 3’ end, was used for the assay. The oligonucleotide sequence was FAM-ATYTCGGCTTTGAGGGGGCCTG-MGB-NFQ. One-step real-time RT-PCR was conducted using the CFX96 Real-Time PCR System (Bio-Rad, Hercules, CA). The cycling conditions were as follows: a reverse transcription step at 50*°C* for 10 min prior to an initial denaturation step at 95*°C* for 1 min, followed by amplification for 40 cycles (denaturation at 95*°C* for 15 s and annealing/extension at 60*°C* for 45 s).

### Thermostability

Viruses (128 hemagglutination units) were prepared in PBS and incubated for 15, 30, and 60 min at 54°C. After heat treatment, hemagglutination activity was measured in hemagglutination assays with 0.8% chicken red blood cells.

### Mouse experiments

Five-week-old female BALB/C mice (Japan SLC) under mixed anesthesia (medetomidine-butorphanol-midazolam) were inoculated intranasally with 75 μl of serial 10-fold dilutions of virus in PBS (10^4^ FFU or 10^5^ FFU virus in 75 μl PBS (-)). Weight loss and mortality were observed daily for 14. Mouse lungs were collected at 3 days post-inoculation with 10^5^ FFU virus, homogenized in PBS (-), and centrifuged at 2,380 × g for 30 min to obtain supernatants. The supernatants were inoculated onto MDCK cells, and the virus titer was determined as described in “Focus-forming assay to measure infectious titers”.

### Statistical analysis

One-way analysis of variance (ANOVA), followed by Tukey’s post-hoc test, was used for multiple comparisons. A p-value < 0.05 or < 0.01 was considered significant. Statistical analysis was performed using GraphPad Prism Version 8 software (GraphPad Software Inc.).

## Supporting information

S1 Fig Multiple alignment of the HA amino acid sequences of current zoonotic H7N9 viruses. The HA amino acid sequences of H7N9 viruses isolated since 2013 were aligned using Clustal W, and the results were visualized using MEGA [94]. The HA sequence of Dk/ZJ (H7N3)(JQ906576) was used as a reference. The sequences were obtained from the NCBI Influenza Virus Resource [66]; the exceptions were Anhui (H7N9)(EPI439507) and Shanghai (H7N9)(EPI439486), which were obtained from the GISAID database (https://gisaid.org/). The amino acid positions marked with an asterisk correspond to those depicted in Fig 1.
(PDF)

S2 Fig Virus-membrane fusion at low pH in MDCK cells expressing the HA protein of H7 viruses with or without HA2-116D. (A) MDCK cells were transfected with the influenza virus HA gene from Ck/Ita (H7N1), Tk/Ita/214845 (H7N3), or Wg/OS (H7N7), and their respective HA mutants (HA2 N116D). Fusion induction over a pH range of 5.375–5.875 was conducted as described in Fig 2. Red squares show polykaryon formation. Micrographs lacking a red square represent a pH above the fusion threshold. Values in red indicate the pH threshold for HA membrane fusion. The pH threshold was determined as described in “Materials and Methods.” Scale bars, 200 μm. (B) Summary of the pH thresholds for membrane fusion for HA mutant viruses in transfected cells. Representative results from the membrane fusion assay are shown.
(TIF)

S3 Fig Thermostability of wild-type and mutant HAs (HA2-N116D and HA2-D116N). A sample of each virus (128 hemagglutination units) was incubated for the indicated times at 54°C. The hemagglutination titers of the heat-treated samples were determined in hemagglutination assays. Isolated avian viruses and recombinant Dk/ZJ (H7N3) harboring the HA gene from avian isolates are shown with a blue line; isolated human viruses and recombinant Dk/ZJ (H7N3) harboring the HA gene from human isolates are shown with a red line. Data are expressed as the mean ± S.D. of three independent results.
(TIF)

S4 Fig Growth kinetics of current H7N9 and other H7 viruses in immortalized human bronchiolar epithelial cells. (A) 21E5 cells, derived from primary human bronchiolar epithelial cells [please see “Materials and Methods”] were infected as described in Fig 4. All cells were infected at an m.o.i. of 0.1. The amount of progeny vRNA within the culture supernatants at 12, 24, 48, 72, 96, and 120 h post-infection was determined by quantitative real-time PCR assays (the parameters of virions released at 48 and 72 h post-infection are shown in Fig 4C). (B) 21E5 cells were infected with the same virus used in (A) at an m.o.i. of 0.1. The infectious titer of released virions within the culture supernatants at 12, 24, 48, 72, 96, and 120 h post-infection was determined in a focus-forming assay (the growth curves based on the infectious virus titer of released virions at 48 and 72 h post-infection are shown in Fig 4D). Data are expressed as the mean ± S.D. of three (A) or four (B) independent results. Asterisks indicate that the value for a virus was significantly different from that of rDk/ZJ (H7N3) within the same graph. A p-value < 0.05 (single asterisk) or < 0.01 (double asterisk) was considered significant (one-way ANOVA followed by Tukey’s multiple comparisons post-hoc test). The growth kinetics of the parent strain [rDk/ZJ (H7N3)] and a recombinant Dk/ZJ (H7N3) virus harboring HA gene of other H7 viruses are shown in blue and orange, respectively. The growth kinetics of recombinant H7 viruses with a specific mutation (HA2-D116N, HA2-N116D, or HA2-K58I) are shown in red.
(TIF)

S5 Fig Graphs showing the correlation between the copy number of virions released from immortalized human bronchiolar epithelial cells, and the pH threshold of HA required to induce membrane fusion. All parameters were taken from Figs 2, S2 and S4.
(TIF)

S6 Fig Growth kinetics of recombinant H7 viruses harboring HA containing multiple basic amino acids in immortalized human bronchiolar epithelial cells. **(A)** 21E5 cells were infected with rDk/ZJ (H7N3) or with recombinant Dk/ZJ (H7N3) [rDk/ZJ-Anhui-HA, rDk/ZJ-Shanghai-HA, rDk/ZJ-Canada-HA, rDk/ZJ-Mexico-HA, or rDk/ZJ-Netherland-HA]. All cells were infected at an m.o.i. of 0.1. The amount of progeny viral RNA within the culture supernatants at 12, 24, 48, 72, 96, and 120 h post-infection was determined by measuring virus titers in quantitative real-time PCR assays. Data are expressed as the mean ± S.D. of four independent results. Asterisks indicate that the values for each virus were significantly different from that of rDk/ZJ (H7N3) within the same graph. A p-value < 0.05 (single asterisk or sharp) or < 0.01 (double asterisk or double sharp) was considered significant (one-way ANOVA followed by Tukey’s multiple comparisons post-hoc test). (B) MDCK cells were transfected with the influenza virus HA gene from Canada (H7N3), Mexico (H7N3), and Netherland (H7N7) and the acid stability of the HA protein was examined in a membrane fusion assay (conducted as described in Fig 2). Representative fields of cells transfected with each of the indicated viruses and exposed to low pH are shown. Red squares show polykaryon formation. Micrographs lacking a red square represent a pH above the fusion threshold. (C) The interaction energies between helix (HA2-75–129) on the stalk and adjacent HA molecules or the other helices (HA2-75–129) on the opposite site were analyzed to assess the structural stability of HA trimetric molecules (please see S1 and S2 Tables). The structural stability of the HA trimer of Canada (H7N3), Mexico (H7N3), and Netherland (H7N7) was compared with that of Dk/ZL (H7N3). Analyzed helix regions (HA2-75–129) are shown as green in the ribbon model of the HA trimer from Dk/ZJ (H7N3).
(TIF)

S7 Fig Differences in susceptibility of primary human bronchiolar epithelial from different donors to infection by current H7N9 and other H7 viruses. Primary human bronchiolar epithelial cells [SAECs, see “Materials and Methods”] from different donors (denoted as donor B and C; SAECs in Fig 6 are from donor A) were infected with wild-type and recombinant H7 viruses. An infectious assay was conducted as described in Fig 6. The experimental conditions and virus strains are same as in Fig 6. Viral infectivity was determined by calculating the percentage of antigen-positive cells after immunostaining at 24 h post-infection. Data are expressed as the mean ± S.D. of three independent results. Asterisks indicate that the value for each virus was significantly different from that of rDk/ZJ (H7N3) within the same graph; sharps indicate that the value for each virus harboring a mutation (HA2-N116D, HA2-D116N, or HA2-K58I) was significantly different from that of each virus without a mutation within the same graph. A p-value < 0.05 (single asterisk or sharps) or < 0.01 (double asterisk or sharp) was considered significant (one-way ANOVA followed by Tukey’s multiple comparisons post-hoc test). The mutated amino acids (HA2-N116D or HA2-D116N) are shown in red (HA2-N116D) and blue (HA2-D116N), respectively. The mutated amino acid HA2-K58I is shown in purple. The position of HA2-116D (native residue) in both rDk/ZJ-Ck/Wales-HA and rDk/ZJ-Tk/Ita/3477-HA is also shown in red. Please note that the mutated amino acid (HA2-116) in rDk/ZJ-Ck/Wales-HA (HA2-D116N) and rDk/ZJ-Tk/Ita/3477-HA (HA2-D116N) is shown in the same color as the background (blue).
(TIF)

S8 Fig Growth kinetics of current H7N9 and other H7 viruses in primary human bronchiolar epithelial cells. (A) Primary human bronchiolar epithelial cells [SAECs, see “Materials and Methods”] were infected as described in Fig 6. All cells were infected at an m.o.i. of 0.1. The amount of progeny vRNA within the culture supernatants at 12, 24, 48, 72, 96, and 120 h post-infection was determined by measuring virus titers in quantitative real-time PCR assays (the parameters of released virions at 24 and 48 h post-infection are shown in Fig 6B). (B) SAECs were infected with same virus used in (A) at an m.o.i. of 0.1. The infectious virus titer of the released virions within the culture supernatants at 12, 24, 48, 72, 96, and 120 h post-infection was determined in a focus-forming assay (the growth curves based on the infectious virus titer of released virions at 24 and 48 h post-infection are shown in Fig 6C). Data are expressed as the mean ± S.D. of three independent results. Asterisks indicate that the value for each virus was significantly different from that of rDk/ZJ (H7N3) within the same graph. A p-value < 0.05 (single asterisk) or < 0.01 (double asterisk) was considered significant (one-way ANOVA followed by Tukey’s multiple comparisons post-hoc test). The growth kinetics of parent strain [rDk/ZJ (H7N3)] and recombinant Dk/ZJ (H7N3) harboring the HA gene of other H7 viruses are shown as blue and orange lines, respectively. The growth kinetics of recombinant H7 viruses harboring a specific mutation (HA2-D116N, HA2-N116D, or HA2-K58I) are shown as a red line.
(TIF)

S1 Table Interaction energies between the helix within the HA2 domain of one monomer and the other two HA molecules. The amino acid sequence of the analyzed helix within the HA2 domain corresponds to HA2-75–129 (please see S6C Fig).
(DOCX)

S2 Table Interaction energies between the helix within the HA2 domain of one monomer and the helices within the other two HA molecules. The amino acid sequence of the analyzed helix within the HA2 domain corresponds to HA2-75–129 (please see S6C Fig).
(DOCX)

S1 Data Relevant parameters used to draw the graphics. (XLSX)
