# Supplementary material for: The host tropism of current zoonotic H7N9 viruses depends mainly on an acid-labile hemagglutinin with a single amino acid mutation in the stalk region
Source: PLoS Pathog. 2024 Oct 22;20(10):e1012427. doi: 10.1371/journal.ppat.1012427 (PMC11495601; doi:10.1371/journal.ppat.1012427)
Supplement: S1 Fig — The HA amino acid sequences of H7N9 viruses isolated since 2013 were aligned using Clustal W, and the results were visualized using MEGA [94]. The HA sequence of Dk/ZJ (H7N3)(JQ906576) was used as a reference. The sequences were obtained from the NCBI Influenza Virus Resource [66]; the exceptions were Anhui (H7N9)(EPI439507) and Shanghai (H7N9)(EPI439486), which were obtained from the GISAID database (https://gisaid.org/). The amino acid positions marked with an asterisk correspond to those depicted in Fig 1. (PDF) [file ppat.1012427.s001.pdf]

Avian viruses

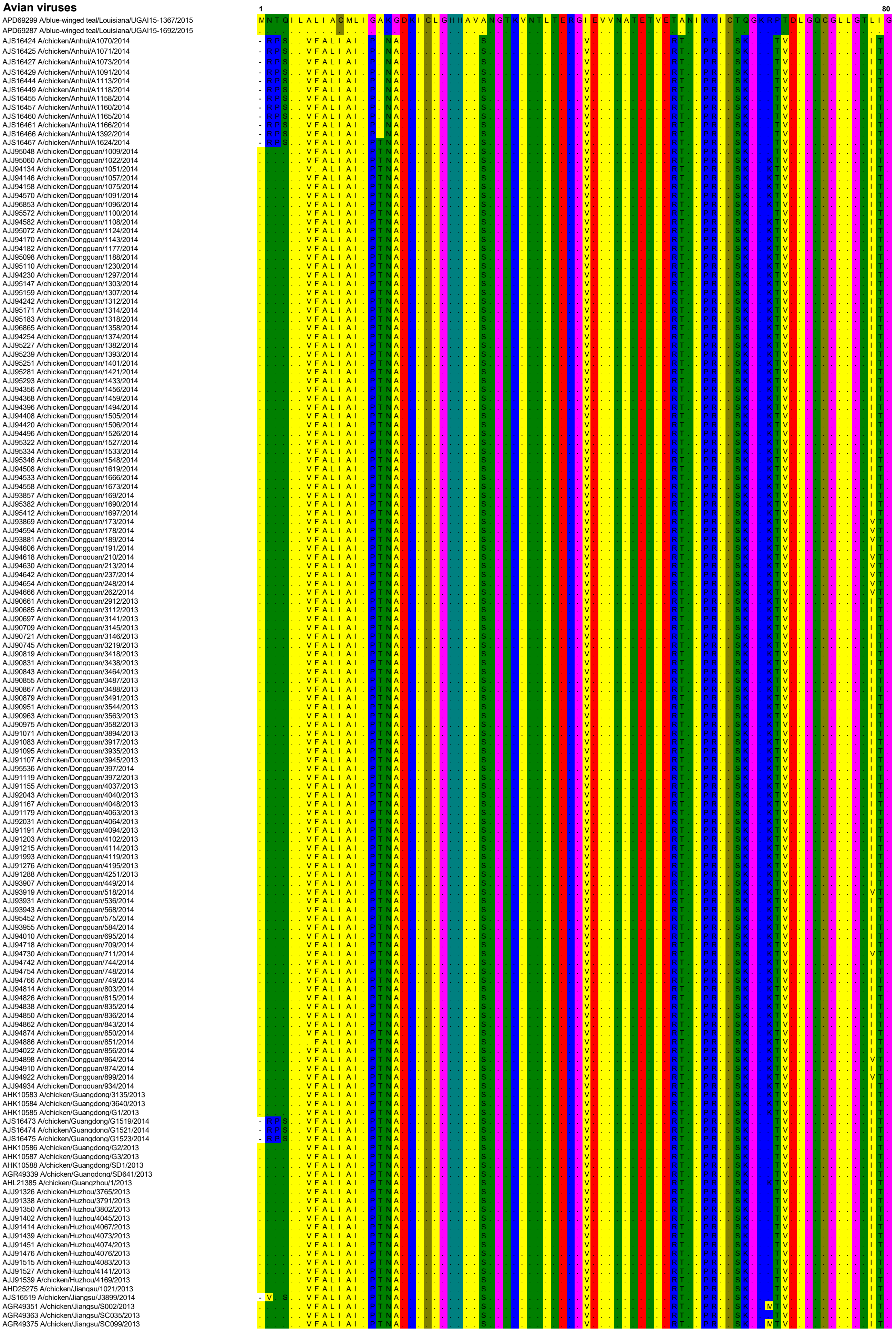

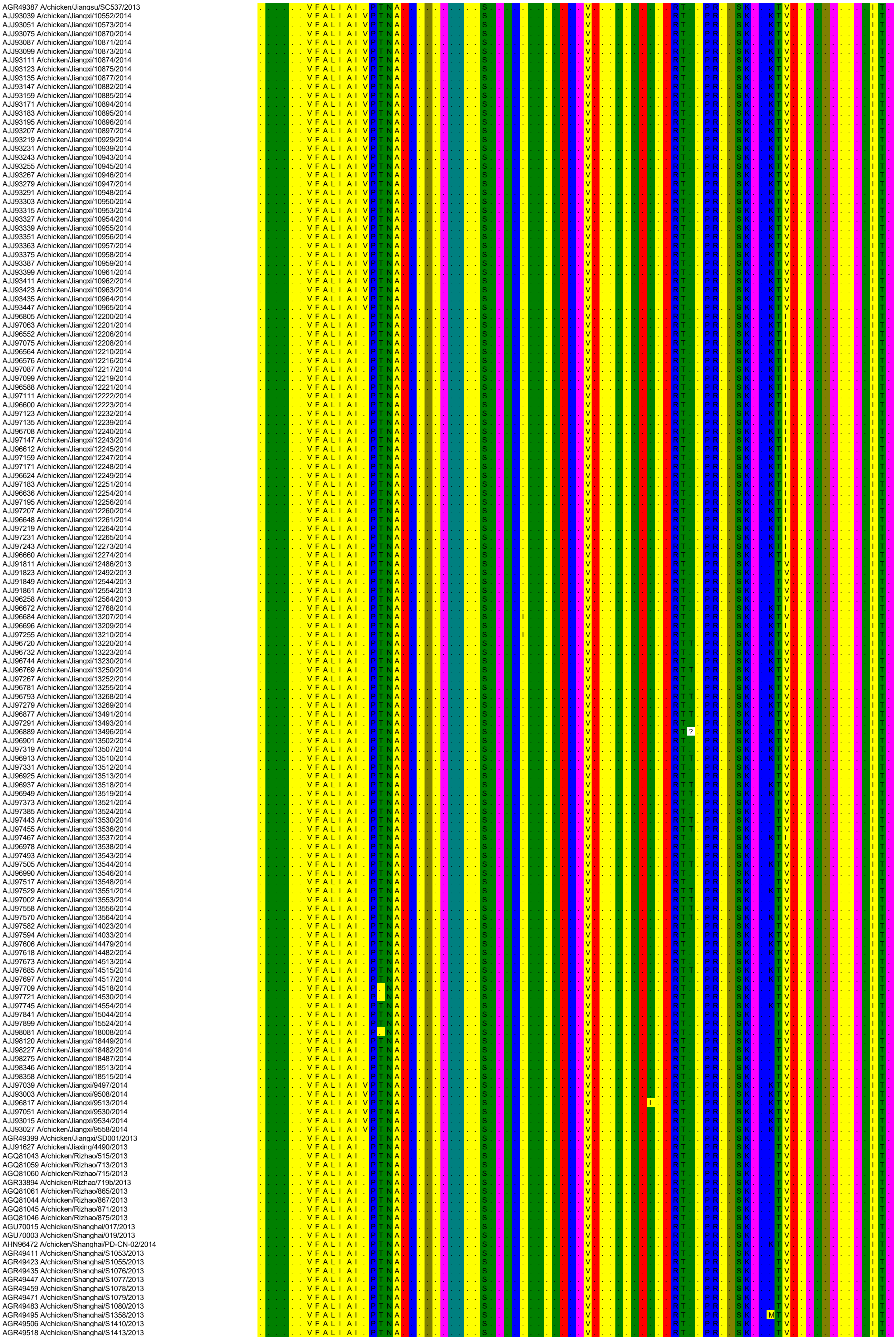
AGR49387 A/chicken/Jiangsu/SC537/2013
AJJ93039 A/chicken/Jiangxi/10552/2014
AJJ93051 A/chicken/Jiangxi/10573/2014
AJJ93075 A/chicken/Jiangxi/10870/2014
AJJ93087 A/chicken/Jiangxi/10871/2014
AJJ93099 A/chicken/Jiangxi/10873/2014
AJJ93111 A/chicken/Jiangxi/10874/2014
AJJ93123 A/chicken/Jiangxi/10875/2014
AJJ93135 A/chicken/Jiangxi/10877/2014
AJJ93147 A/chicken/Jiangxi/10882/2014
AJJ93159 A/chicken/Jiangxi/10885/2014
AJJ93171 A/chicken/Jiangxi/10894/2014
AJJ93183 A/chicken/Jiangxi/10895/2014
AJJ93195 A/chicken/Jiangxi/10896/2014
AJJ93207 A/chicken/Jiangxi/10897/2014
AJJ93219 A/chicken/Jiangxi/10929/2014
AJJ93231 A/chicken/Jiangxi/10939/2014
AJJ93243 A/chicken/Jiangxi/10943/2014
AJJ93255 A/chicken/Jiangxi/10945/2014
AJJ93267 A/chicken/Jiangxi/10946/2014
AJJ93279 A/chicken/Jiangxi/10947/2014
AJJ93291 A/chicken/Jiangxi/10948/2014
AJJ93303 A/chicken/Jiangxi/10950/2014
AJJ93315 A/chicken/Jiangxi/10953/2014
AJJ93327 A/chicken/Jiangxi/10954/2014
AJJ93339 A/chicken/Jiangxi/10955/2014
AJJ93351 A/chicken/Jiangxi/10956/2014
AJJ93363 A/chicken/Jiangxi/10957/2014
AJJ93375 A/chicken/Jiangxi/10958/2014
AJJ93387 A/chicken/Jiangxi/10959/2014
AJJ93399 A/chicken/Jiangxi/10961/2014
AJJ93411 A/chicken/Jiangxi/10962/2014
AJJ93423 A/chicken/Jiangxi/10963/2014
AJJ93435 A/chicken/Jiangxi/10964/2014
AJJ93447 A/chicken/Jiangxi/10965/2014
AJJ96805 A/chicken/Jiangxi/12200/2014
AJJ97063 A/chicken/Jiangxi/12201/2014
AJJ96552 A/chicken/Jiangxi/12206/2014
AJJ97075 A/chicken/Jiangxi/12208/2014
AJJ96564 A/chicken/Jiangxi/12210/2014
AJJ96576 A/chicken/Jiangxi/12216/2014
AJJ97087 A/chicken/Jiangxi/12217/2014
AJJ97099 A/chicken/Jiangxi/12219/2014
AJJ96588 A/chicken/Jiangxi/12221/2014
AJJ97111 A/chicken/Jiangxi/12222/2014
AJJ96600 A/chicken/Jiangxi/12223/2014
AJJ97123 A/chicken/Jiangxi/12232/2014
AJJ97135 A/chicken/Jiangxi/12239/2014
AJJ96708 A/chicken/Jiangxi/12240/2014
AJJ97147 A/chicken/Jiangxi/12243/2014
AJJ96612 A/chicken/Jiangxi/12245/2014
AJJ97159 A/chicken/Jiangxi/12247/2014
AJJ97171 A/chicken/Jiangxi/12248/2014
AJJ96624 A/chicken/Jiangxi/12249/2014
AJJ97183 A/chicken/Jiangxi/12251/2014
AJJ96636 A/chicken/Jiangxi/12254/2014
AJJ97195 A/chicken/Jiangxi/12256/2014
AJJ97207 A/chicken/Jiangxi/12260/2014
AJJ96648 A/chicken/Jiangxi/12261/2014
AJJ97219 A/chicken/Jiangxi/12264/2014
AJJ97231 A/chicken/Jiangxi/12265/2014
AJJ97243 A/chicken/Jiangxi/12273/2014
AJJ96660 A/chicken/Jiangxi/12274/2014
AJJ91811 A/chicken/Jiangxi/12486/2013
AJJ91823 A/chicken/Jiangxi/12492/2013
AJJ91849 A/chicken/Jiangxi/12544/2013
AJJ91861 A/chicken/Jiangxi/12554/2013
AJJ96258 A/chicken/Jiangxi/12564/2013
AJJ96672 A/chicken/Jiangxi/12768/2014
AJJ96684 A/chicken/Jiangxi/13207/2014
AJJ96696 A/chicken/Jiangxi/13209/2014
AJJ97255 A/chicken/Jiangxi/13210/2014
AJJ96720 A/chicken/Jiangxi/13220/2014
AJJ96732 A/chicken/Jiangxi/13223/2014
AJJ96744 A/chicken/Jiangxi/13230/2014
AJJ96769 A/chicken/Jiangxi/13250/2014
AJJ97267 A/chicken/Jiangxi/13252/2014
AJJ96781 A/chicken/Jiangxi/13255/2014
AJJ96793 A/chicken/Jiangxi/13268/2014
AJJ97279 A/chicken/Jiangxi/13269/2014
AJJ96877 A/chicken/Jiangxi/13491/2014
AJJ97291 A/chicken/Jiangxi/13493/2014
AJJ96889 A/chicken/Jiangxi/13496/2014
AJJ96901 A/chicken/Jiangxi/13502/2014
AJJ97319 A/chicken/Jiangxi/13507/2014
AJJ96913 A/chicken/Jiangxi/13510/2014
AJJ97331 A/chicken/Jiangxi/13512/2014
AJJ96925 A/chicken/Jiangxi/13513/2014
AJJ96937 A/chicken/Jiangxi/13518/2014
AJJ96949 A/chicken/Jiangxi/13519/2014
AJJ97373 A/chicken/Jiangxi/13521/2014
AJJ97385 A/chicken/Jiangxi/13524/2014
AJJ97443 A/chicken/Jiangxi/13530/2014
AJJ97455 A/chicken/Jiangxi/13536/2014
AJJ97467 A/chicken/Jiangxi/13537/2014
AJJ96978 A/chicken/Jiangxi/13538/2014
AJJ97493 A/chicken/Jiangxi/13543/2014
AJJ97505 A/chicken/Jiangxi/13544/2014
AJJ96990 A/chicken/Jiangxi/13546/2014
AJJ97517 A/chicken/Jiangxi/13548/2014
AJJ97529 A/chicken/Jiangxi/13551/2014
AJJ97002 A/chicken/Jiangxi/13553/2014
AJJ97558 A/chicken/Jiangxi/13556/2014
AJJ97570 A/chicken/Jiangxi/13564/2014
AJJ97582 A/chicken/Jiangxi/14023/2014
AJJ97594 A/chicken/Jiangxi/14033/2014
AJJ97606 A/chicken/Jiangxi/14479/2014
AJJ97618 A/chicken/Jiangxi/14482/2014
AJJ97673 A/chicken/Jiangxi/14513/2014
AJJ97685 A/chicken/Jiangxi/14515/2014
AJJ97697 A/chicken/Jiangxi/14517/2014
AJJ97709 A/chicken/Jiangxi/14518/2014
AJJ97721 A/chicken/Jiangxi/14530/2014
AJJ97745 A/chicken/Jiangxi/14554/2014
AJJ97841 A/chicken/Jiangxi/15044/2014
AJJ97899 A/chicken/Jiangxi/15524/2014
AJJ98081 A/chicken/Jiangxi/18008/2014
AJJ98120 A/chicken/Jiangxi/18449/2014
AJJ98227 A/chicken/Jiangxi/18482/2014
AJJ98275 A/chicken/Jiangxi/18487/2014
AJJ98346 A/chicken/Jiangxi/18513/2014
AJJ98358 A/chicken/Jiangxi/18515/2014
AJJ97039 A/chicken/Jiangxi/9497/2014
AJJ93003 A/chicken/Jiangxi/9508/2014
AJJ96817 A/chicken/Jiangxi/9513/2014
AJJ97051 A/chicken/Jiangxi/9530/2014
AJJ93015 A/chicken/Jiangxi/9534/2014
AJJ93027 A/chicken/Jiangxi/9558/2014
AGR49399 A/chicken/Jiangxi/SD001/2013
AJJ91627 A/chicken/Jiaxing/4490/2013
AGQ81043 A/chicken/Rizhao/515/2013
AGQ81059 A/chicken/Rizhao/713/2013
AGQ81060 A/chicken/Rizhao/715/2013
AGR33894 A/chicken/Rizhao/719b/2013
AGQ81061 A/chicken/Rizhao/865/2013
AGQ81044 A/chicken/Rizhao/867/2013
AGQ81045 A/chicken/Rizhao/871/2013
AGQ81046 A/chicken/Rizhao/875/2013
AGU70015 A/chicken/Shanghai/017/2013
AGU70003 A/chicken/Shanghai/019/2013
AHN96472 A/chicken/Shanghai/PD-CN-02/2014
AGR49411 A/chicken/Shanghai/S1053/2013
AGR49423 A/chicken/Shanghai/S1055/2013
AGR49435 A/chicken/Shanghai/S1076/2013
AGR49447 A/chicken/Shanghai/S1077/2013
AGR49459 A/chicken/Shanghai/S1078/2013
AGR49471 A/chicken/Shanghai/S1079/2013
AGR49483 A/chicken/Shanghai/S1080/2013
AGR49495 A/chicken/Shanghai/S1358/2013
AGR49506 A/chicken/Shanghai/S1410/2013
AGR49518 A/chicken/Shanghai/S1413/2013

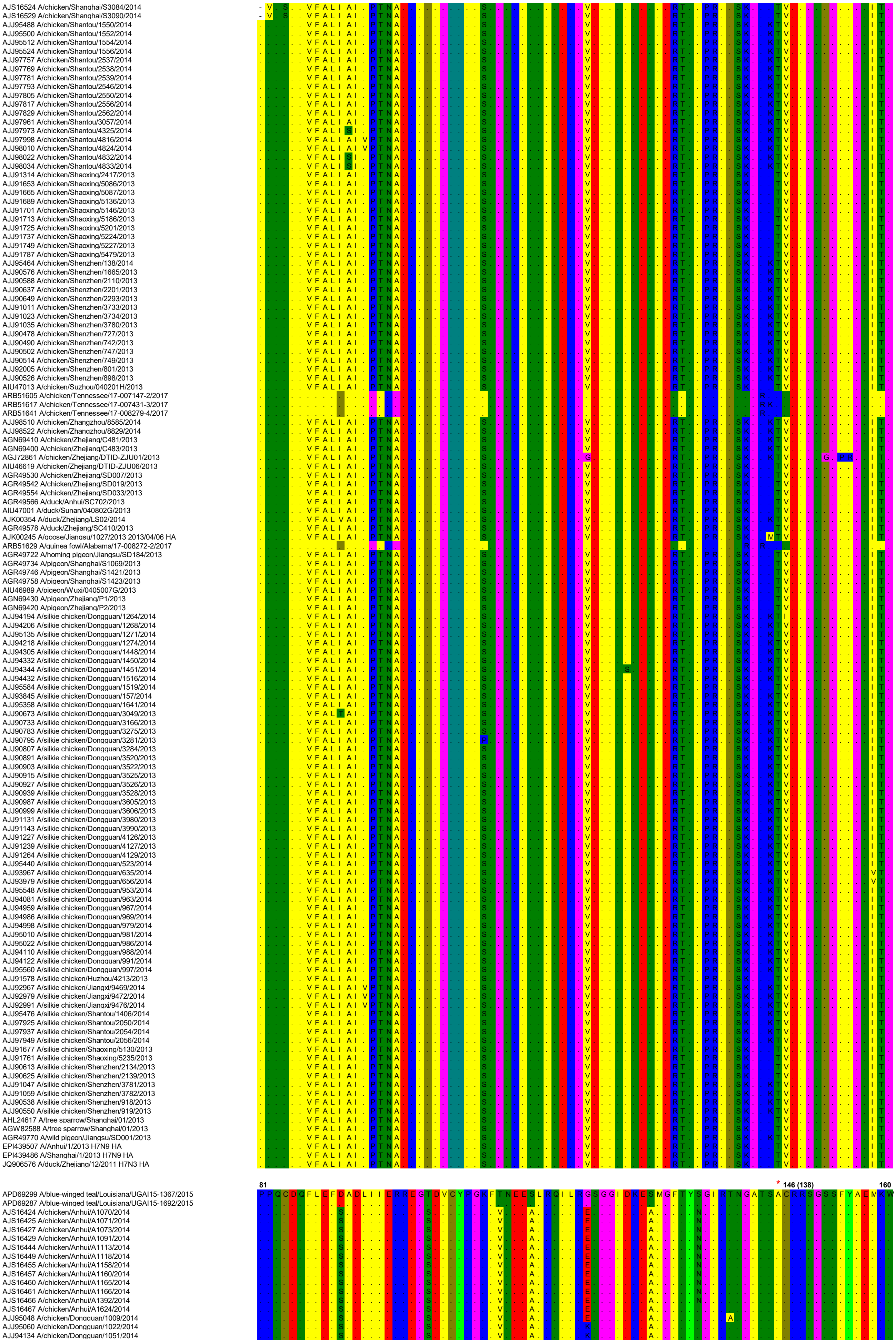

AJS16524 A/chicken/Shanghai/S3084/2014
AJS16529 A/chicken/Shanghai/S3090/2014
AJJ95488 A/chicken/Shantou/1550/2014
AJJ95500 A/chicken/Shantou/1552/2014
AJJ95512 A/chicken/Shantou/1554/2014
AJJ95524 A/chicken/Shantou/1556/2014
AJJ97757 A/chicken/Shantou/2537/2014
AJJ97769 A/chicken/Shantou/2538/2014
AJJ97781 A/chicken/Shantou/2539/2014
AJJ97793 A/chicken/Shantou/2546/2014
AJJ97805 A/chicken/Shantou/2550/2014
AJJ97817 A/chicken/Shantou/2556/2014
AJJ97829 A/chicken/Shantou/2562/2014
AJJ97961 A/chicken/Shantou/3057/2014
AJJ97973 A/chicken/Shantou/4325/2014
AJJ97998 A/chicken/Shantou/4816/2014
AJJ98010 A/chicken/Shantou/4824/2014
AJJ98022 A/chicken/Shantou/4832/2014
AJJ98034 A/chicken/Shantou/4833/2014
AJJ91314 A/chicken/Shaoxing/2417/2013
AJJ91653 A/chicken/Shaoxing/5086/2013
AJJ91665 A/chicken/Shaoxing/5087/2013
AJJ91689 A/chicken/Shaoxing/5136/2013
AJJ91701 A/chicken/Shaoxing/5146/2013
AJJ91713 A/chicken/Shaoxing/5186/2013
AJJ91725 A/chicken/Shaoxing/5201/2013
AJJ91737 A/chicken/Shaoxing/5224/2013
AJJ91749 A/chicken/Shaoxing/5227/2013
AJJ91787 A/chicken/Shaoxing/5479/2013
AJJ95464 A/chicken/Shenzhen/138/2014
AJJ90576 A/chicken/Shenzhen/1665/2013
AJJ90588 A/chicken/Shenzhen/2110/2013
AJJ90637 A/chicken/Shenzhen/2201/2013
AJJ90649 A/chicken/Shenzhen/2293/2013
AJJ91011 A/chicken/Shenzhen/3733/2013
AJJ91023 A/chicken/Shenzhen/3734/2013
AJJ91035 A/chicken/Shenzhen/3780/2013
AJJ90478 A/chicken/Shenzhen/727/2013
AJJ90490 A/chicken/Shenzhen/742/2013
AJJ90502 A/chicken/Shenzhen/747/2013
AJJ90514 A/chicken/Shenzhen/749/2013
AJJ92005 A/chicken/Shenzhen/801/2013
AJJ90526 A/chicken/Shenzhen/898/2013
AIU47013 A/chicken/Suzhou/040201H/2013
ARB51605 A/chicken/Tennessee/17-007147-2/2017
ARB51617 A/chicken/Tennessee/17-007431-3/2017
ARB51641 A/chicken/Tennessee/17-008279-4/2017
AJJ98510 A/chicken/Zhangzhou/8585/2014
AJJ98522 A/chicken/Zhangzhou/8829/2014
AGN69410 A/chicken/Zhejiang/C481/2013
AGN69400 A/chicken/Zhejiang/C483/2013
AGJ72861 A/chicken/Zhejiang/DTID-ZJU01/2013
AIU46619 A/chicken/Zhejiang/DTID-ZJU06/2013
AGR49530 A/chicken/Zhejiang/SD007/2013
AGR49542 A/chicken/Zhejiang/SD019/2013
AGR49554 A/chicken/Zhejiang/SD033/2013
AGR49566 A/duck/Anhui/SC702/2013
AIU47001 A/duck/Sunan/040802G/2013
AJK00354 A/duck/Zhejiang/LS02/2014
AGR49578 A/duck/Zhejiang/SC410/2013
AJK00245 A/goose/Jiangsu/1027/2013 2013/04/06 HA
ARB51629 A/guinea fowl/Alabama/17-008272-2/2017
AGR49722 A/homing pigeon/Jiangsu/SD184/2013
AGR49734 A/pigeon/Shanghai/S1069/2013
AGR49746 A/pigeon/Shanghai/S1421/2013
AGR49758 A/pigeon/Shanghai/S1423/2013
AIU46989 A/pigeon/Wuxi/0405007G/2013
AGN69430 A/pigeon/Zhejiang/P1/2013
AGN69420 A/pigeon/Zhejiang/P2/2013
AJJ94194 A/silkie chicken/Dongguan/1264/2014
AJJ94206 A/silkie chicken/Dongguan/1268/2014
AJJ95135 A/silkie chicken/Dongguan/1271/2014
AJJ94218 A/silkie chicken/Dongguan/1274/2014
AJJ94305 A/silkie chicken/Dongguan/1448/2014
AJJ94332 A/silkie chicken/Dongguan/1450/2014
AJJ94344 A/silkie chicken/Dongguan/1451/2014
AJJ94432 A/silkie chicken/Dongguan/1516/2014
AJJ95584 A/silkie chicken/Dongguan/1519/2014
AJJ93845 A/silkie chicken/Dongguan/157/2014
AJJ95358 A/silkie chicken/Dongguan/1641/2014
AJJ90673 A/silkie chicken/Dongguan/3049/2013
AJJ90733 A/silkie chicken/Dongguan/3166/2013
AJJ90783 A/silkie chicken/Dongguan/3275/2013
AJJ90795 A/silkie chicken/Dongguan/3281/2013
AJJ90807 A/silkie chicken/Dongguan/3284/2013
AJJ90891 A/silkie chicken/Dongguan/3520/2013
AJJ90903 A/silkie chicken/Dongguan/3522/2013
AJJ90915 A/silkie chicken/Dongguan/3525/2013
AJJ90927 A/silkie chicken/Dongguan/3526/2013
AJJ90939 A/silkie chicken/Dongguan/3528/2013
AJJ90987 A/silkie chicken/Dongguan/3605/2013
AJJ90999 A/silkie chicken/Dongguan/3606/2013
AJJ91131 A/silkie chicken/Dongguan/3980/2013
AJJ91143 A/silkie chicken/Dongguan/3990/2013
AJJ91227 A/silkie chicken/Dongguan/4126/2013
AJJ91239 A/silkie chicken/Dongguan/4127/2013
AJJ91264 A/silkie chicken/Dongguan/4129/2013
AJJ95440 A/silkie chicken/Dongguan/523/2014
AJJ93967 A/silkie chicken/Dongguan/635/2014
AJJ93979 A/silkie chicken/Dongguan/656/2014
AJJ95548 A/silkie chicken/Dongguan/953/2014
AJJ94081 A/silkie chicken/Dongguan/963/2014
AJJ94959 A/silkie chicken/Dongguan/967/2014
AJJ94986 A/silkie chicken/Dongguan/969/2014
AJJ94998 A/silkie chicken/Dongguan/979/2014
AJJ95010 A/silkie chicken/Dongguan/981/2014
AJJ95022 A/silkie chicken/Dongguan/986/2014
AJJ94110 A/silkie chicken/Dongguan/988/2014
AJJ94122 A/silkie chicken/Dongguan/991/2014
AJJ95560 A/silkie chicken/Dongguan/997/2014
AJJ91578 A/silkie chicken/Huzhou/4213/2013
AJJ92967 A/silkie chicken/Jiangxi/9469/2014
AJJ92979 A/silkie chicken/Jiangxi/9472/2014
AJJ92991 A/silkie chicken/Jiangxi/9476/2014
AJJ95476 A/silkie chicken/Shantou/1406/2014
AJJ97925 A/silkie chicken/Shantou/2050/2014
AJJ97937 A/silkie chicken/Shantou/2054/2014
AJJ97949 A/silkie chicken/Shantou/2056/2014
AJJ91677 A/silkie chicken/Shaoxing/5130/2013
AJJ91761 A/silkie chicken/Shaoxing/5235/2013
AJJ90613 A/silkie chicken/Shenzhen/2134/2013
AJJ90625 A/silkie chicken/Shenzhen/2139/2013
AJJ91047 A/silkie chicken/Shenzhen/3781/2013
AJJ91059 A/silkie chicken/Shenzhen/3782/2013
AJJ90538 A/silkie chicken/Shenzhen/918/2013
AJJ90550 A/silkie chicken/Shenzhen/919/2013
AHL24617 A/tree sparrow/Shanghai/01/2013
AGW82588 A/tree sparrow/Shanghai/01/2013
AGR49770 A/wild pigeon/Jiangsu/SD001/2013
EPI439507 A/Anhui/1/2013 H7N9 HA
EPI439486 A/Shanghai/1/2013 H7N9 HA
JQ906576 A/duck/Zhejiang/12/2011 H7N3 HA
81
146 (138)
160
APD69299 A/blue-winged teal/Louisiana/UGAI15-1367/2015
APD69287 A/blue-winged teal/Louisiana/UGAI15-1692/2015
AJS16424 A/chicken/Anhui/A1070/2014
AJS16425 A/chicken/Anhui/A1071/2014
AJS16427 A/chicken/Anhui/A1073/2014
AJS16429 A/chicken/Anhui/A1091/2014
AJS16444 A/chicken/Anhui/A1113/2014
AJS16449 A/chicken/Anhui/A1118/2014
AJS16455 A/chicken/Anhui/A1158/2014
AJS16457 A/chicken/Anhui/A1160/2014
AJS16460 A/chicken/Anhui/A1165/2014
AJS16461 A/chicken/Anhui/A1166/2014
AJS16466 A/chicken/Anhui/A1392/2014
AJS16467 A/chicken/Anhui/A1624/2014
AJJ95048 A/chicken/Dongguan/1009/2014
AJJ95060 A/chicken/Dongguan/1022/2014
AJJ94134 A/chicken/Dongguan/1051/2014

AJJ94146 A/chicken/Dongguan/1057/2014
AJJ94158 A/chicken/Dongguan/1075/2014
AJJ94570 A/chicken/Dongguan/1091/2014
AJJ96853 A/chicken/Dongguan/1096/2014
AJJ95572 A/chicken/Dongguan/1100/2014
AJJ94582 A/chicken/Dongguan/1108/2014
AJJ95072 A/chicken/Dongguan/1124/2014
AJJ94170 A/chicken/Dongguan/1143/2014
AJJ94182 A/chicken/Dongguan/1177/2014
AJJ95098 A/chicken/Dongguan/1188/2014
AJJ95110 A/chicken/Dongguan/1230/2014
AJJ94230 A/chicken/Dongguan/1297/2014
AJJ95147 A/chicken/Dongguan/1303/2014
AJJ95159 A/chicken/Dongguan/1307/2014
AJJ94242 A/chicken/Dongguan/1312/2014
AJJ95171 A/chicken/Dongguan/1314/2014
AJJ95183 A/chicken/Dongguan/1318/2014
AJJ96865 A/chicken/Dongguan/1358/2014
AJJ94254 A/chicken/Dongguan/1374/2014
AJJ95227 A/chicken/Dongguan/1382/2014
AJJ95239 A/chicken/Dongguan/1393/2014
AJJ95251 A/chicken/Dongguan/1401/2014
AJJ95281 A/chicken/Dongguan/1421/2014
AJJ95293 A/chicken/Dongguan/1433/2014
AJJ94356 A/chicken/Dongguan/1456/2014
AJJ94368 A/chicken/Dongguan/1459/2014
AJJ94396 A/chicken/Dongguan/1494/2014
AJJ94408 A/chicken/Dongguan/1505/2014
AJJ94420 A/chicken/Dongguan/1506/2014
AJJ94496 A/chicken/Dongguan/1526/2014
AJJ95322 A/chicken/Dongguan/1527/2014
AJJ95334 A/chicken/Dongguan/1533/2014
AJJ95346 A/chicken/Dongguan/1548/2014
AJJ94508 A/chicken/Dongguan/1619/2014
AJJ94533 A/chicken/Dongguan/1666/2014
AJJ94558 A/chicken/Dongguan/1673/2014
AJJ93857 A/chicken/Dongguan/169/2014
AJJ95382 A/chicken/Dongguan/1690/2014
AJJ95412 A/chicken/Dongguan/1697/2014
AJJ93869 A/chicken/Dongguan/173/2014
AJJ94594 A/chicken/Dongguan/178/2014
AJJ93881 A/chicken/Dongguan/189/2014
AJJ94606 A/chicken/Dongguan/191/2014
AJJ94618 A/chicken/Dongguan/210/2014
AJJ94630 A/chicken/Dongguan/213/2014
AJJ94642 A/chicken/Dongguan/237/2014
AJJ94654 A/chicken/Dongguan/248/2014
AJJ94666 A/chicken/Dongguan/262/2014
AJJ90661 A/chicken/Dongguan/2912/2013
AJJ90685 A/chicken/Dongguan/3112/2013
AJJ90697 A/chicken/Dongguan/3141/2013
AJJ90709 A/chicken/Dongguan/3145/2013
AJJ90721 A/chicken/Dongguan/3146/2013
AJJ90745 A/chicken/Dongguan/3219/2013
AJJ90819 A/chicken/Dongguan/3418/2013
AJJ90831 A/chicken/Dongguan/3438/2013
AJJ90843 A/chicken/Dongguan/3464/2013
AJJ90855 A/chicken/Dongguan/3487/2013
AJJ90867 A/chicken/Dongguan/3488/2013
AJJ90879 A/chicken/Dongguan/3491/2013
AJJ90951 A/chicken/Dongguan/3544/2013
AJJ90963 A/chicken/Dongguan/3563/2013
AJJ90975 A/chicken/Dongguan/3582/2013
AJJ91071 A/chicken/Dongguan/3894/2013
AJJ91083 A/chicken/Dongguan/3917/2013
AJJ91095 A/chicken/Dongguan/3935/2013
AJJ91107 A/chicken/Dongguan/3945/2013
AJJ95536 A/chicken/Dongguan/397/2014
AJJ91119 A/chicken/Dongguan/3972/2013
AJJ91155 A/chicken/Dongguan/4037/2013
AJJ92043 A/chicken/Dongguan/4040/2013
AJJ91167 A/chicken/Dongguan/4048/2013
AJJ91179 A/chicken/Dongguan/4063/2013
AJJ92031 A/chicken/Dongguan/4064/2013
AJJ91191 A/chicken/Dongguan/4094/2013
AJJ91203 A/chicken/Dongguan/4102/2013
AJJ91215 A/chicken/Dongguan/4114/2013
AJJ91993 A/chicken/Dongguan/4119/2013
AJJ91276 A/chicken/Dongguan/4195/2013
AJJ91288 A/chicken/Dongguan/4251/2013
AJJ93907 A/chicken/Dongguan/449/2014
AJJ93919 A/chicken/Dongguan/518/2014
AJJ93931 A/chicken/Dongguan/536/2014
AJJ93943 A/chicken/Dongguan/568/2014
AJJ95452 A/chicken/Dongguan/575/2014
AJJ93955 A/chicken/Dongguan/584/2014
AJJ94010 A/chicken/Dongguan/695/2014
AJJ94718 A/chicken/Dongguan/709/2014
AJJ94730 A/chicken/Dongguan/711/2014
AJJ94742 A/chicken/Dongguan/744/2014
AJJ94754 A/chicken/Dongguan/748/2014
AJJ94766 A/chicken/Dongguan/749/2014
AJJ94814 A/chicken/Dongguan/803/2014
AJJ94826 A/chicken/Dongguan/815/2014
AJJ94838 A/chicken/Dongguan/835/2014
AJJ94850 A/chicken/Dongguan/836/2014
AJJ94862 A/chicken/Dongguan/843/2014
AJJ94874 A/chicken/Dongguan/850/2014
AJJ94886 A/chicken/Dongguan/851/2014
AJJ94022 A/chicken/Dongguan/856/2014
AJJ94898 A/chicken/Dongguan/864/2014
AJJ94910 A/chicken/Dongguan/874/2014
AJJ94922 A/chicken/Dongguan/899/2014
AJJ94934 A/chicken/Dongguan/934/2014
AHK10583 A/chicken/Guangdong/3135/2013
AHK10584 A/chicken/Guangdong/3640/2013
AHK10585 A/chicken/Guangdong/G1/2013
AJS16473 A/chicken/Guangdong/G1519/2014
AJS16474 A/chicken/Guangdong/G1521/2014
AJS16475 A/chicken/Guangdong/G1523/2014
AHK10586 A/chicken/Guangdong/G2/2013
AHK10587 A/chicken/Guangdong/G3/2013
AHK10588 A/chicken/Guangdong/SD1/2013
AGR49339 A/chicken/Guangdong/SD641/2013
AHL21385 A/chicken/Guangzhou/1/2013
AJJ91326 A/chicken/Huzhou/3765/2013
AJJ91338 A/chicken/Huzhou/3791/2013
AJJ91350 A/chicken/Huzhou/3802/2013
AJJ91402 A/chicken/Huzhou/4045/2013
AJJ91414 A/chicken/Huzhou/4067/2013
AJJ91439 A/chicken/Huzhou/4073/2013
AJJ91451 A/chicken/Huzhou/4074/2013
AJJ91476 A/chicken/Huzhou/4076/2013
AJJ91515 A/chicken/Huzhou/4083/2013
AJJ91527 A/chicken/Huzhou/4141/2013
AJJ91539 A/chicken/Huzhou/4169/2013
AHD25275 A/chicken/Jiangsu/1021/2013
AJS16519 A/chicken/Jiangsu/J3899/2014
AGR49351 A/chicken/Jiangsu/S002/2013
AGR49363 A/chicken/Jiangsu/SC035/2013
AGR49375 A/chicken/Jiangsu/SC099/2013
AGR49387 A/chicken/Jiangsu/SC537/2013
AJJ93039 A/chicken/Jiangxi/10552/2014
AJJ93051 A/chicken/Jiangxi/10573/2014
AJJ93075 A/chicken/Jiangxi/10870/2014
AJJ93087 A/chicken/Jiangxi/10871/2014
AJJ93099 A/chicken/Jiangxi/10873/2014
AJJ93111 A/chicken/Jiangxi/10874/2014
AJJ93123 A/chicken/Jiangxi/10875/2014
AJJ93135 A/chicken/Jiangxi/10877/2014
AJJ93147 A/chicken/Jiangxi/10882/2014
AJJ93159 A/chicken/Jiangxi/10885/2014
AJJ93171 A/chicken/Jiangxi/10894/2014
AJJ93183 A/chicken/Jiangxi/10895/2014
AJJ93195 A/chicken/Jiangxi/10896/2014
AJJ93207 A/chicken/Jiangxi/10897/2014
AJJ93219 A/chicken/Jiangxi/10929/2014
AJJ93231 A/chicken/Jiangxi/10939/2014
AJJ93243 A/chicken/Jiangxi/10943/2014
AJJ93255 A/chicken/Jiangxi/10945/2014
AJJ93267 A/chicken/Jiangxi/10946/2014

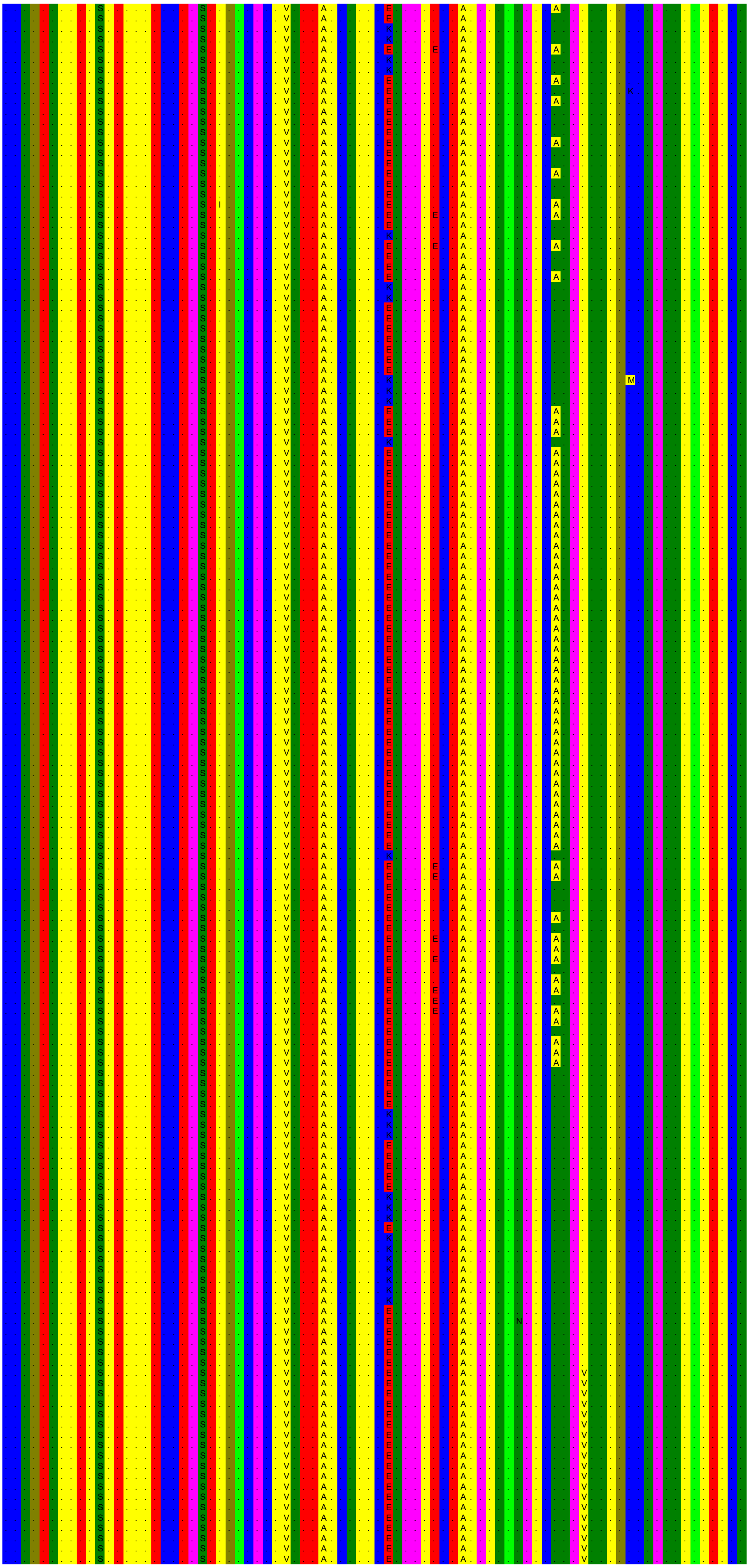

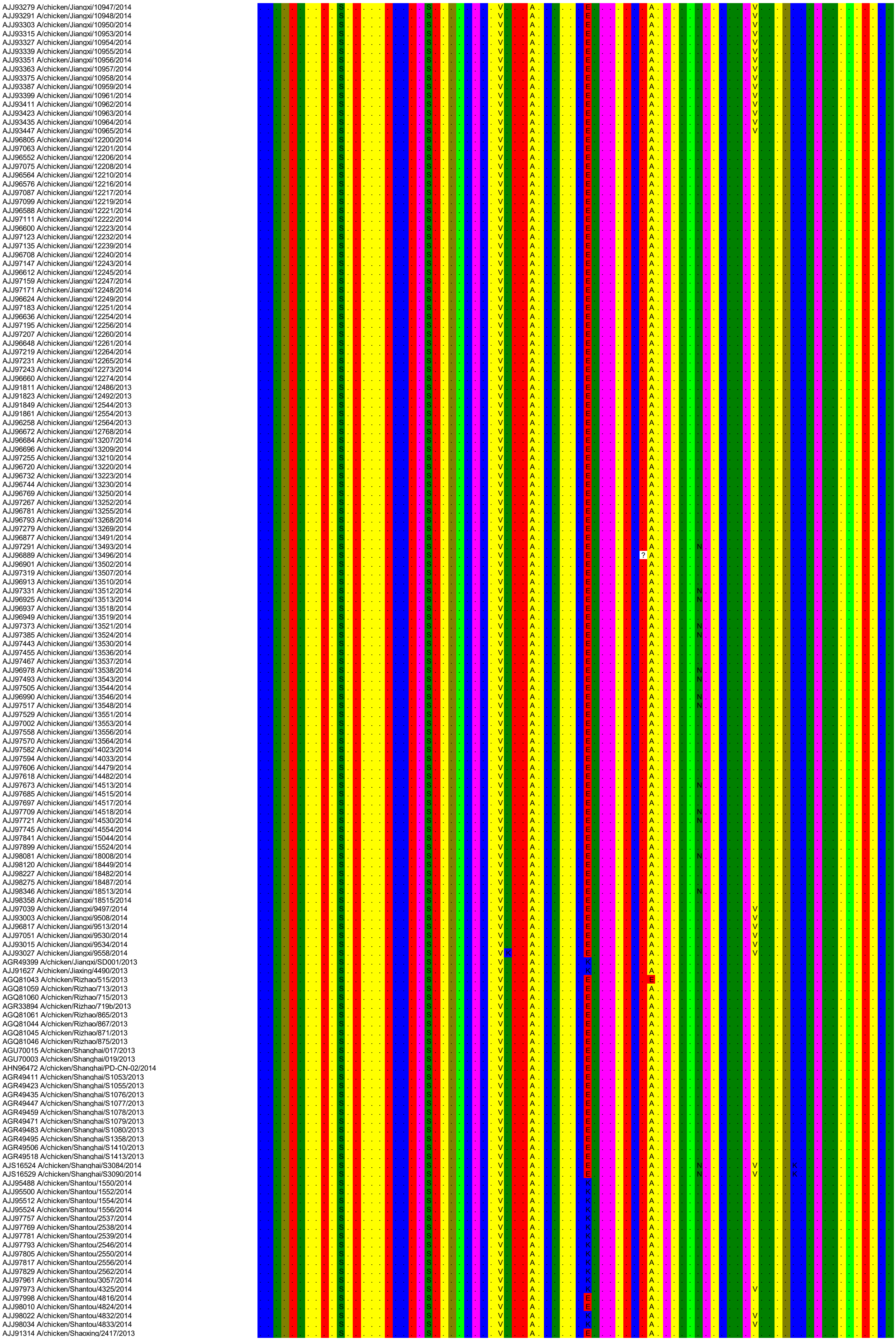
AJJ93279 A/chicken/Jiangxi/10947/2014
AJJ93291 A/chicken/Jiangxi/10948/2014
AJJ93303 A/chicken/Jiangxi/10950/2014
AJJ93315 A/chicken/Jiangxi/10953/2014
AJJ93327 A/chicken/Jiangxi/10954/2014
AJJ93339 A/chicken/Jiangxi/10955/2014
AJJ93351 A/chicken/Jiangxi/10956/2014
AJJ93363 A/chicken/Jiangxi/10957/2014
AJJ93375 A/chicken/Jiangxi/10958/2014
AJJ93387 A/chicken/Jiangxi/10959/2014
AJJ93399 A/chicken/Jiangxi/10961/2014
AJJ93411 A/chicken/Jiangxi/10962/2014
AJJ93423 A/chicken/Jiangxi/10963/2014
AJJ93435 A/chicken/Jiangxi/10964/2014
AJJ93447 A/chicken/Jiangxi/10965/2014
AJJ96805 A/chicken/Jiangxi/12200/2014
AJJ97063 A/chicken/Jiangxi/12201/2014
AJJ96552 A/chicken/Jiangxi/12206/2014
AJJ97075 A/chicken/Jiangxi/12208/2014
AJJ96564 A/chicken/Jiangxi/12210/2014
AJJ96576 A/chicken/Jiangxi/12216/2014
AJJ97087 A/chicken/Jiangxi/12217/2014
AJJ97099 A/chicken/Jiangxi/12219/2014
AJJ96588 A/chicken/Jiangxi/12221/2014
AJJ97111 A/chicken/Jiangxi/12222/2014
AJJ96600 A/chicken/Jiangxi/12223/2014
AJJ97123 A/chicken/Jiangxi/12232/2014
AJJ97135 A/chicken/Jiangxi/12239/2014
AJJ96708 A/chicken/Jiangxi/12240/2014
AJJ97147 A/chicken/Jiangxi/12243/2014
AJJ96612 A/chicken/Jiangxi/12245/2014
AJJ97159 A/chicken/Jiangxi/12247/2014
AJJ97171 A/chicken/Jiangxi/12248/2014
AJJ96624 A/chicken/Jiangxi/12249/2014
AJJ97183 A/chicken/Jiangxi/12251/2014
AJJ96636 A/chicken/Jiangxi/12254/2014
AJJ97195 A/chicken/Jiangxi/12256/2014
AJJ97207 A/chicken/Jiangxi/12260/2014
AJJ96648 A/chicken/Jiangxi/12261/2014
AJJ97219 A/chicken/Jiangxi/12264/2014
AJJ97231 A/chicken/Jiangxi/12265/2014
AJJ97243 A/chicken/Jiangxi/12273/2014
AJJ96660 A/chicken/Jiangxi/12274/2014
AJJ91811 A/chicken/Jiangxi/12486/2013
AJJ91823 A/chicken/Jiangxi/12492/2013
AJJ91849 A/chicken/Jiangxi/12544/2013
AJJ91861 A/chicken/Jiangxi/12554/2013
AJJ96258 A/chicken/Jiangxi/12564/2013
AJJ96672 A/chicken/Jiangxi/12768/2014
AJJ96684 A/chicken/Jiangxi/13207/2014
AJJ96696 A/chicken/Jiangxi/13209/2014
AJJ97255 A/chicken/Jiangxi/13210/2014
AJJ96720 A/chicken/Jiangxi/13220/2014
AJJ96732 A/chicken/Jiangxi/13223/2014
AJJ96744 A/chicken/Jiangxi/13230/2014
AJJ96769 A/chicken/Jiangxi/13250/2014
AJJ97267 A/chicken/Jiangxi/13252/2014
AJJ96781 A/chicken/Jiangxi/13255/2014
AJJ96793 A/chicken/Jiangxi/13268/2014
AJJ97279 A/chicken/Jiangxi/13269/2014
AJJ96877 A/chicken/Jiangxi/13491/2014
AJJ97291 A/chicken/Jiangxi/13493/2014
AJJ96889 A/chicken/Jiangxi/13496/2014
AJJ96901 A/chicken/Jiangxi/13502/2014
AJJ97319 A/chicken/Jiangxi/13507/2014
AJJ96913 A/chicken/Jiangxi/13510/2014
AJJ97331 A/chicken/Jiangxi/13512/2014
AJJ96925 A/chicken/Jiangxi/13513/2014
AJJ96937 A/chicken/Jiangxi/13518/2014
AJJ96949 A/chicken/Jiangxi/13519/2014
AJJ97373 A/chicken/Jiangxi/13521/2014
AJJ97385 A/chicken/Jiangxi/13524/2014
AJJ97443 A/chicken/Jiangxi/13530/2014
AJJ97455 A/chicken/Jiangxi/13536/2014
AJJ97467 A/chicken/Jiangxi/13537/2014
AJJ96978 A/chicken/Jiangxi/13538/2014
AJJ97493 A/chicken/Jiangxi/13543/2014
AJJ97505 A/chicken/Jiangxi/13544/2014
AJJ96990 A/chicken/Jiangxi/13546/2014
AJJ97517 A/chicken/Jiangxi/13548/2014
AJJ97529 A/chicken/Jiangxi/13551/2014
AJJ97002 A/chicken/Jiangxi/13553/2014
AJJ97558 A/chicken/Jiangxi/13556/2014
AJJ97570 A/chicken/Jiangxi/13564/2014
AJJ97582 A/chicken/Jiangxi/14023/2014
AJJ97594 A/chicken/Jiangxi/14033/2014
AJJ97606 A/chicken/Jiangxi/14479/2014
AJJ97618 A/chicken/Jiangxi/14482/2014
AJJ97673 A/chicken/Jiangxi/14513/2014
AJJ97685 A/chicken/Jiangxi/14515/2014
AJJ97697 A/chicken/Jiangxi/14517/2014
AJJ97709 A/chicken/Jiangxi/14518/2014
AJJ97721 A/chicken/Jiangxi/14530/2014
AJJ97745 A/chicken/Jiangxi/14554/2014
AJJ97841 A/chicken/Jiangxi/15044/2014
AJJ97899 A/chicken/Jiangxi/15524/2014
AJJ98081 A/chicken/Jiangxi/18008/2014
AJJ98120 A/chicken/Jiangxi/18449/2014
AJJ98227 A/chicken/Jiangxi/18482/2014
AJJ98275 A/chicken/Jiangxi/18487/2014
AJJ98346 A/chicken/Jiangxi/18513/2014
AJJ98358 A/chicken/Jiangxi/18515/2014
AJJ97039 A/chicken/Jiangxi/9497/2014
AJJ93003 A/chicken/Jiangxi/9508/2014
AJJ96817 A/chicken/Jiangxi/9513/2014
AJJ97051 A/chicken/Jiangxi/9530/2014
AJJ93015 A/chicken/Jiangxi/9534/2014
AJJ93027 A/chicken/Jiangxi/9558/2014
AGR49399 A/chicken/Jiangxi/SD001/2013
AJJ91627 A/chicken/Jiaxing/4490/2013
AGQ81043 A/chicken/Rizhao/515/2013
AGQ81059 A/chicken/Rizhao/713/2013
AGQ81060 A/chicken/Rizhao/715/2013
AGR33894 A/chicken/Rizhao/719b/2013
AGQ81061 A/chicken/Rizhao/865/2013
AGQ81044 A/chicken/Rizhao/867/2013
AGQ81045 A/chicken/Rizhao/871/2013
AGQ81046 A/chicken/Rizhao/875/2013
AGU70015 A/chicken/Shanghai/017/2013
AGU70003 A/chicken/Shanghai/019/2013
AHN96472 A/chicken/Shanghai/PD-CN-02/2014
AGR49411 A/chicken/Shanghai/S1053/2013
AGR49423 A/chicken/Shanghai/S1055/2013
AGR49435 A/chicken/Shanghai/S1076/2013
AGR49447 A/chicken/Shanghai/S1077/2013
AGR49459 A/chicken/Shanghai/S1078/2013
AGR49471 A/chicken/Shanghai/S1079/2013
AGR49483 A/chicken/Shanghai/S1080/2013
AGR49495 A/chicken/Shanghai/S1358/2013
AGR49506 A/chicken/Shanghai/S1410/2013
AGR49518 A/chicken/Shanghai/S1413/2013
AJS16524 A/chicken/Shanghai/S3084/2014
AJS16529 A/chicken/Shanghai/S3090/2014
AJJ95488 A/chicken/Shantou/1550/2014
AJJ95500 A/chicken/Shantou/1552/2014
AJJ95512 A/chicken/Shantou/1554/2014
AJJ95524 A/chicken/Shantou/1556/2014
AJJ97757 A/chicken/Shantou/2537/2014
AJJ97769 A/chicken/Shantou/2538/2014
AJJ97781 A/chicken/Shantou/2539/2014
AJJ97793 A/chicken/Shantou/2546/2014
AJJ97805 A/chicken/Shantou/2550/2014
AJJ97817 A/chicken/Shantou/2556/2014
AJJ97829 A/chicken/Shantou/2562/2014
AJJ97961 A/chicken/Shantou/3057/2014
AJJ97973 A/chicken/Shantou/4325/2014
AJJ97998 A/chicken/Shantou/4816/2014
AJJ98010 A/chicken/Shantou/4824/2014
AJJ98022 A/chicken/Shantou/4832/2014
AJJ98034 A/chicken/Shantou/4833/2014
AJJ91314 A/chicken/Shaoxing/2417/2013

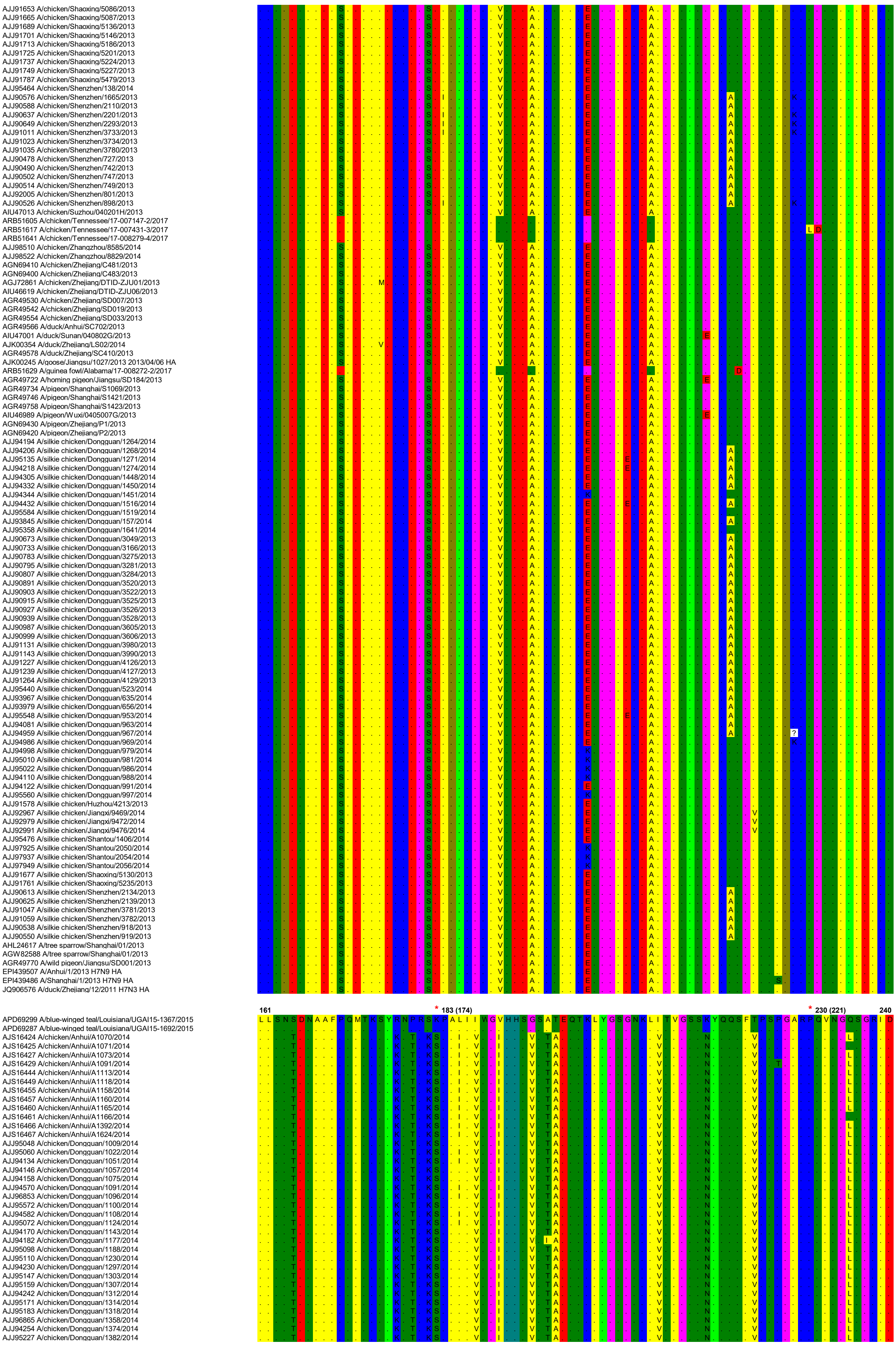
AJJ91653 A/chicken/Shaoxing/5086/2013
AJJ91665 A/chicken/Shaoxing/5087/2013
AJJ91689 A/chicken/Shaoxing/5136/2013
AJJ91701 A/chicken/Shaoxing/5146/2013
AJJ91713 A/chicken/Shaoxing/5186/2013
AJJ91725 A/chicken/Shaoxing/5201/2013
AJJ91737 A/chicken/Shaoxing/5224/2013
AJJ91749 A/chicken/Shaoxing/5227/2013
AJJ91787 A/chicken/Shaoxing/5479/2013
AJJ95464 A/chicken/Shenzhen/138/2014
AJJ90576 A/chicken/Shenzhen/1665/2013
AJJ90588 A/chicken/Shenzhen/2110/2013
AJJ90637 A/chicken/Shenzhen/2201/2013
AJJ90649 A/chicken/Shenzhen/2293/2013
AJJ91011 A/chicken/Shenzhen/3733/2013
AJJ91023 A/chicken/Shenzhen/3734/2013
AJJ91035 A/chicken/Shenzhen/3780/2013
AJJ90478 A/chicken/Shenzhen/727/2013
AJJ90490 A/chicken/Shenzhen/742/2013
AJJ90502 A/chicken/Shenzhen/747/2013
AJJ90514 A/chicken/Shenzhen/749/2013
AJJ92005 A/chicken/Shenzhen/801/2013
AJJ90526 A/chicken/Shenzhen/898/2013
AIU47013 A/chicken/Suzhou/040201H/2013
ARB51605 A/chicken/Tennessee/17-007147-2/2017
ARB51617 A/chicken/Tennessee/17-007431-3/2017
ARB51641 A/chicken/Tennessee/17-008279-4/2017
AJJ98510 A/chicken/Zhangzhou/8585/2014
AJJ98522 A/chicken/Zhangzhou/8829/2014
AGN69410 A/chicken/Zhejiang/C481/2013
AGN69400 A/chicken/Zhejiang/C483/2013
AGJ72861 A/chicken/Zhejiang/DTID-ZJU01/2013
AIU46619 A/chicken/Zhejiang/DTID-ZJU06/2013
AGR49530 A/chicken/Zhejiang/SD007/2013
AGR49542 A/chicken/Zhejiang/SD019/2013
AGR49554 A/chicken/Zhejiang/SD033/2013
AGR49566 A/duck/Anhui/SC702/2013
AIU47001 A/duck/Sunan/040802G/2013
AJK00354 A/duck/Zhejiang/LS02/2014
AGR49578 A/duck/Zhejiang/SC410/2013
AJK00245 A/goose/Jiangsu/1027/2013 2013/04/06 HA
ARB51629 A/guinea fowl/Alabama/17-008272-2/2017
AGR49722 A/homing pigeon/Jiangsu/SD184/2013
AGR49734 A/pigeon/Shanghai/S1069/2013
AGR49746 A/pigeon/Shanghai/S1421/2013
AGR49758 A/pigeon/Shanghai/S1423/2013
AIU46989 A/pigeon/Wuxi/0405007G/2013
AGN69430 A/pigeon/Zhejiang/P1/2013
AGN69420 A/pigeon/Zhejiang/P2/2013
AJJ94194 A/silkie chicken/Dongguan/1264/2014
AJJ94206 A/silkie chicken/Dongguan/1268/2014
AJJ95135 A/silkie chicken/Dongguan/1271/2014
AJJ94218 A/silkie chicken/Dongguan/1274/2014
AJJ94305 A/silkie chicken/Dongguan/1448/2014
AJJ94332 A/silkie chicken/Dongguan/1450/2014
AJJ94344 A/silkie chicken/Dongguan/1451/2014
AJJ94432 A/silkie chicken/Dongguan/1516/2014
AJJ95584 A/silkie chicken/Dongguan/1519/2014
AJJ93845 A/silkie chicken/Dongguan/157/2014
AJJ95358 A/silkie chicken/Dongguan/1641/2014
AJJ90673 A/silkie chicken/Dongguan/3049/2013
AJJ90733 A/silkie chicken/Dongguan/3166/2013
AJJ90783 A/silkie chicken/Dongguan/3275/2013
AJJ90795 A/silkie chicken/Dongguan/3281/2013
AJJ90807 A/silkie chicken/Dongguan/3284/2013
AJJ90891 A/silkie chicken/Dongguan/3520/2013
AJJ90903 A/silkie chicken/Dongguan/3522/2013
AJJ90915 A/silkie chicken/Dongguan/3525/2013
AJJ90927 A/silkie chicken/Dongguan/3526/2013
AJJ90939 A/silkie chicken/Dongguan/3528/2013
AJJ90987 A/silkie chicken/Dongguan/3605/2013
AJJ90999 A/silkie chicken/Dongguan/3606/2013
AJJ91131 A/silkie chicken/Dongguan/3980/2013
AJJ91143 A/silkie chicken/Dongguan/3990/2013
AJJ91227 A/silkie chicken/Dongguan/4126/2013
AJJ91239 A/silkie chicken/Dongguan/4127/2013
AJJ91264 A/silkie chicken/Dongguan/4129/2013
AJJ95440 A/silkie chicken/Dongguan/523/2014
AJJ93967 A/silkie chicken/Dongguan/635/2014
AJJ93979 A/silkie chicken/Dongguan/656/2014
AJJ95548 A/silkie chicken/Dongguan/953/2014
AJJ94081 A/silkie chicken/Dongguan/963/2014
AJJ94959 A/silkie chicken/Dongguan/967/2014
AJJ94986 A/silkie chicken/Dongguan/969/2014
AJJ94998 A/silkie chicken/Dongguan/979/2014
AJJ95010 A/silkie chicken/Dongguan/981/2014
AJJ95022 A/silkie chicken/Dongguan/986/2014
AJJ94110 A/silkie chicken/Dongguan/988/2014
AJJ94122 A/silkie chicken/Dongguan/991/2014
AJJ95560 A/silkie chicken/Dongguan/997/2014
AJJ91578 A/silkie chicken/Huzhou/4213/2013
AJJ92967 A/silkie chicken/Jiangxi/9469/2014
AJJ92979 A/silkie chicken/Jiangxi/9472/2014
AJJ92991 A/silkie chicken/Jiangxi/9476/2014
AJJ95476 A/silkie chicken/Shantou/1406/2014
AJJ97925 A/silkie chicken/Shantou/2050/2014
AJJ97937 A/silkie chicken/Shantou/2054/2014
AJJ97949 A/silkie chicken/Shantou/2056/2014
AJJ91677 A/silkie chicken/Shaoxing/5130/2013
AJJ91761 A/silkie chicken/Shaoxing/5235/2013
AJJ90613 A/silkie chicken/Shenzhen/2134/2013
AJJ90625 A/silkie chicken/Shenzhen/2139/2013
AJJ91047 A/silkie chicken/Shenzhen/3781/2013
AJJ91059 A/silkie chicken/Shenzhen/3782/2013
AJJ90538 A/silkie chicken/Shenzhen/918/2013
AJJ90550 A/silkie chicken/Shenzhen/919/2013
AHL24617 A/tree sparrow/Shanghai/01/2013
AGW82588 A/tree sparrow/Shanghai/01/2013
AGR49770 A/wild pigeon/Jiangsu/SD001/2013
EPI439507 A/Anhui/1/2013 H7N9 HA
EPI439486 A/Shanghai/1/2013 H7N9 HA
JQ906576 A/duck/Zhejiang/12/2011 H7N3 HA
161
* 183 (174)
* 230 (221)
240
APD69299 A/blue-winged teal/Louisiana/UGAI15-1367/2015
APD69287 A/blue-winged teal/Louisiana/UGAI15-1692/2015
AJS16424 A/chicken/Anhui/A1070/2014
AJS16425 A/chicken/Anhui/A1071/2014
AJS16427 A/chicken/Anhui/A1073/2014
AJS16429 A/chicken/Anhui/A1091/2014
AJS16444 A/chicken/Anhui/A1113/2014
AJS16449 A/chicken/Anhui/A1118/2014
AJS16455 A/chicken/Anhui/A1158/2014
AJS16457 A/chicken/Anhui/A1160/2014
AJS16460 A/chicken/Anhui/A1165/2014
AJS16461 A/chicken/Anhui/A1166/2014
AJS16466 A/chicken/Anhui/A1392/2014
AJS16467 A/chicken/Anhui/A1624/2014
AJJ95048 A/chicken/Dongguan/1009/2014
AJJ95060 A/chicken/Dongguan/1022/2014
AJJ94134 A/chicken/Dongguan/1051/2014
AJJ94146 A/chicken/Dongguan/1057/2014
AJJ94158 A/chicken/Dongguan/1075/2014
AJJ94570 A/chicken/Dongguan/1091/2014
AJJ96853 A/chicken/Dongguan/1096/2014
AJJ95572 A/chicken/Dongguan/1100/2014
AJJ94582 A/chicken/Dongguan/1108/2014
AJJ95072 A/chicken/Dongguan/1124/2014
AJJ94170 A/chicken/Dongguan/1143/2014
AJJ94182 A/chicken/Dongguan/1177/2014
AJJ95098 A/chicken/Dongguan/1188/2014
AJJ95110 A/chicken/Dongguan/1230/2014
AJJ94230 A/chicken/Dongguan/1297/2014
AJJ95147 A/chicken/Dongguan/1303/2014
AJJ95159 A/chicken/Dongguan/1307/2014
AJJ94242 A/chicken/Dongguan/1312/2014
AJJ95171 A/chicken/Dongguan/1314/2014
AJJ95183 A/chicken/Dongguan/1318/2014
AJJ96865 A/chicken/Dongguan/1358/2014
AJJ94254 A/chicken/Dongguan/1374/2014
AJJ95227 A/chicken/Dongguan/1382/2014

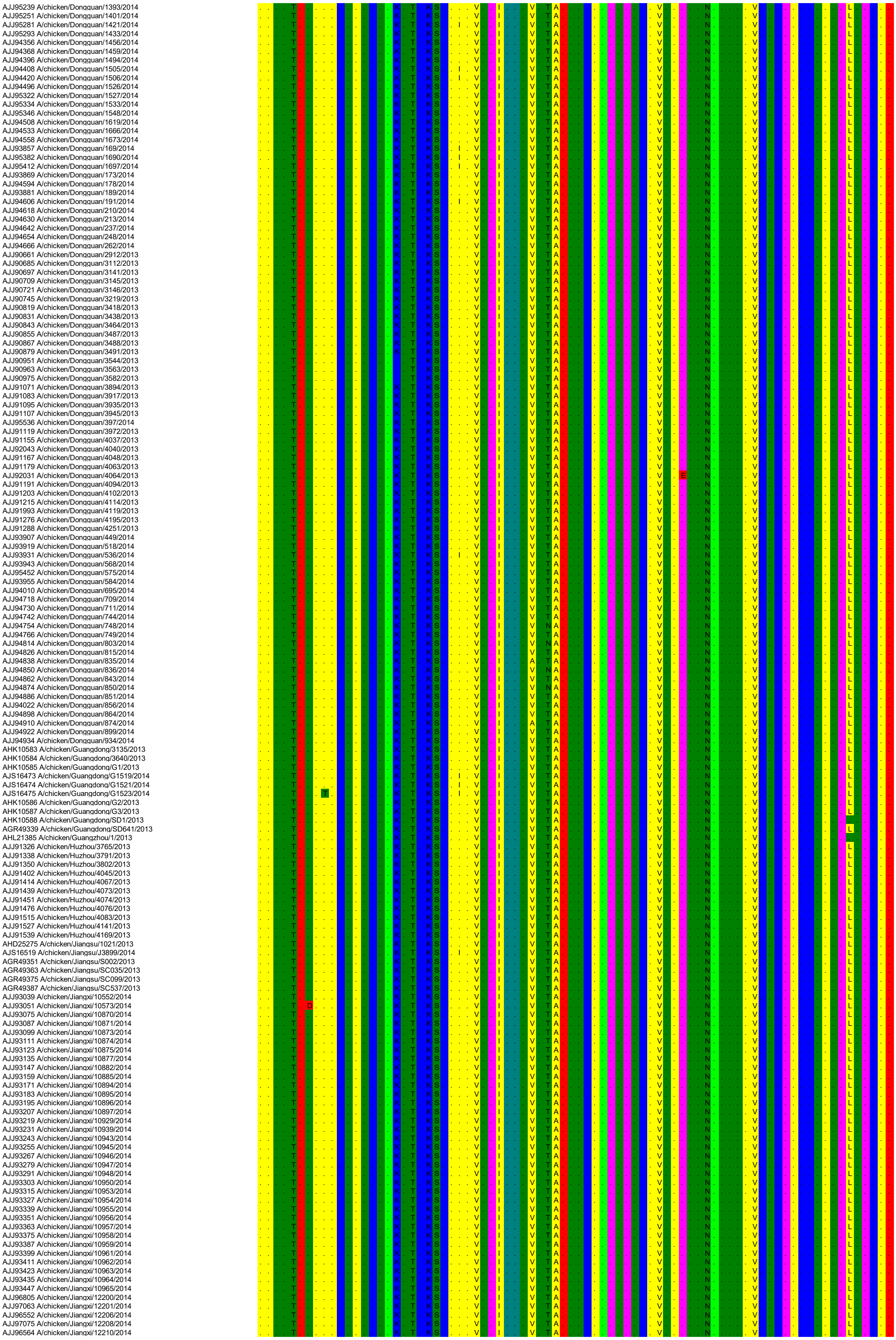
AJJ95239 A/chicken/Dongguan/1393/2014
AJJ95251 A/chicken/Dongguan/1401/2014
AJJ95281 A/chicken/Dongguan/1421/2014
AJJ95293 A/chicken/Dongguan/1433/2014
AJJ94356 A/chicken/Dongguan/1456/2014
AJJ94368 A/chicken/Dongguan/1459/2014
AJJ94396 A/chicken/Dongguan/1494/2014
AJJ94408 A/chicken/Dongguan/1505/2014
AJJ94420 A/chicken/Dongguan/1506/2014
AJJ94496 A/chicken/Dongguan/1526/2014
AJJ95322 A/chicken/Dongguan/1527/2014
AJJ95334 A/chicken/Dongguan/1533/2014
AJJ95346 A/chicken/Dongguan/1548/2014
AJJ94508 A/chicken/Dongguan/1619/2014
AJJ94533 A/chicken/Dongguan/1666/2014
AJJ94558 A/chicken/Dongguan/1673/2014
AJJ93857 A/chicken/Dongguan/169/2014
AJJ95382 A/chicken/Dongguan/1690/2014
AJJ95412 A/chicken/Dongguan/1697/2014
AJJ93869 A/chicken/Dongguan/173/2014
AJJ94594 A/chicken/Dongguan/178/2014
AJJ93881 A/chicken/Dongguan/189/2014
AJJ94606 A/chicken/Dongguan/191/2014
AJJ94618 A/chicken/Dongguan/210/2014
AJJ94630 A/chicken/Dongguan/213/2014
AJJ94642 A/chicken/Dongguan/237/2014
AJJ94654 A/chicken/Dongguan/248/2014
AJJ94666 A/chicken/Dongguan/262/2014
AJJ90661 A/chicken/Dongguan/2912/2013
AJJ90685 A/chicken/Dongguan/3112/2013
AJJ90697 A/chicken/Dongguan/3141/2013
AJJ90709 A/chicken/Dongguan/3145/2013
AJJ90721 A/chicken/Dongguan/3146/2013
AJJ90745 A/chicken/Dongguan/3219/2013
AJJ90819 A/chicken/Dongguan/3418/2013
AJJ90831 A/chicken/Dongguan/3438/2013
AJJ90843 A/chicken/Dongguan/3464/2013
AJJ90855 A/chicken/Dongguan/3487/2013
AJJ90867 A/chicken/Dongguan/3488/2013
AJJ90879 A/chicken/Dongguan/3491/2013
AJJ90951 A/chicken/Dongguan/3544/2013
AJJ90963 A/chicken/Dongguan/3563/2013
AJJ90975 A/chicken/Dongguan/3582/2013
AJJ91071 A/chicken/Dongguan/3894/2013
AJJ91083 A/chicken/Dongguan/3917/2013
AJJ91095 A/chicken/Dongguan/3935/2013
AJJ91107 A/chicken/Dongguan/3945/2013
AJJ95536 A/chicken/Dongguan/397/2014
AJJ91119 A/chicken/Dongguan/3972/2013
AJJ91155 A/chicken/Dongguan/4037/2013
AJJ92043 A/chicken/Dongguan/4040/2013
AJJ91167 A/chicken/Dongguan/4048/2013
AJJ91179 A/chicken/Dongguan/4063/2013
AJJ92031 A/chicken/Dongguan/4064/2013
AJJ91191 A/chicken/Dongguan/4094/2013
AJJ91203 A/chicken/Dongguan/4102/2013
AJJ91215 A/chicken/Dongguan/4114/2013
AJJ91993 A/chicken/Dongguan/4119/2013
AJJ91276 A/chicken/Dongguan/4195/2013
AJJ91288 A/chicken/Dongguan/4251/2013
AJJ93907 A/chicken/Dongguan/449/2014
AJJ93919 A/chicken/Dongguan/518/2014
AJJ93931 A/chicken/Dongguan/536/2014
AJJ93943 A/chicken/Dongguan/568/2014
AJJ95452 A/chicken/Dongguan/575/2014
AJJ93955 A/chicken/Dongguan/584/2014
AJJ94010 A/chicken/Dongguan/695/2014
AJJ94718 A/chicken/Dongguan/709/2014
AJJ94730 A/chicken/Dongguan/711/2014
AJJ94742 A/chicken/Dongguan/744/2014
AJJ94754 A/chicken/Dongguan/748/2014
AJJ94766 A/chicken/Dongguan/749/2014
AJJ94814 A/chicken/Dongguan/803/2014
AJJ94826 A/chicken/Dongguan/815/2014
AJJ94838 A/chicken/Dongguan/835/2014
AJJ94850 A/chicken/Dongguan/836/2014
AJJ94862 A/chicken/Dongguan/843/2014
AJJ94874 A/chicken/Dongguan/850/2014
AJJ94886 A/chicken/Dongguan/851/2014
AJJ94022 A/chicken/Dongguan/856/2014
AJJ94898 A/chicken/Dongguan/864/2014
AJJ94910 A/chicken/Dongguan/874/2014
AJJ94922 A/chicken/Dongguan/899/2014
AJJ94934 A/chicken/Dongguan/934/2014
AHK10583 A/chicken/Guangdong/3135/2013
AHK10584 A/chicken/Guangdong/3640/2013
AHK10585 A/chicken/Guangdong/G1/2013
AJS16473 A/chicken/Guangdong/G1519/2014
AJS16474 A/chicken/Guangdong/G1521/2014
AJS16475 A/chicken/Guangdong/G1523/2014
AHK10586 A/chicken/Guangdong/G2/2013
AHK10587 A/chicken/Guangdong/G3/2013
AHK10588 A/chicken/Guangdong/SD1/2013
AGR49339 A/chicken/Guangdong/SD641/2013
AHL21385 A/chicken/Guangzhou/1/2013
AJJ91326 A/chicken/Huzhou/3765/2013
AJJ91338 A/chicken/Huzhou/3791/2013
AJJ91350 A/chicken/Huzhou/3802/2013
AJJ91402 A/chicken/Huzhou/4045/2013
AJJ91414 A/chicken/Huzhou/4067/2013
AJJ91439 A/chicken/Huzhou/4073/2013
AJJ91451 A/chicken/Huzhou/4074/2013
AJJ91476 A/chicken/Huzhou/4076/2013
AJJ91515 A/chicken/Huzhou/4083/2013
AJJ91527 A/chicken/Huzhou/4141/2013
AJJ91539 A/chicken/Huzhou/4169/2013
AHD25275 A/chicken/Jiangsu/1021/2013
AJS16519 A/chicken/Jiangsu/J3899/2014
AGR49351 A/chicken/Jiangsu/S002/2013
AGR49363 A/chicken/Jiangsu/SC035/2013
AGR49375 A/chicken/Jiangsu/SC099/2013
AGR49387 A/chicken/Jiangsu/SC537/2013
AJJ93039 A/chicken/Jiangxi/10552/2014
AJJ93051 A/chicken/Jiangxi/10573/2014
AJJ93075 A/chicken/Jiangxi/10870/2014
AJJ93087 A/chicken/Jiangxi/10871/2014
AJJ93099 A/chicken/Jiangxi/10873/2014
AJJ93111 A/chicken/Jiangxi/10874/2014
AJJ93123 A/chicken/Jiangxi/10875/2014
AJJ93135 A/chicken/Jiangxi/10877/2014
AJJ93147 A/chicken/Jiangxi/10882/2014
AJJ93159 A/chicken/Jiangxi/10885/2014
AJJ93171 A/chicken/Jiangxi/10894/2014
AJJ93183 A/chicken/Jiangxi/10895/2014
AJJ93195 A/chicken/Jiangxi/10896/2014
AJJ93207 A/chicken/Jiangxi/10897/2014
AJJ93219 A/chicken/Jiangxi/10929/2014
AJJ93231 A/chicken/Jiangxi/10939/2014
AJJ93243 A/chicken/Jiangxi/10943/2014
AJJ93255 A/chicken/Jiangxi/10945/2014
AJJ93267 A/chicken/Jiangxi/10946/2014
AJJ93279 A/chicken/Jiangxi/10947/2014
AJJ93291 A/chicken/Jiangxi/10948/2014
AJJ93303 A/chicken/Jiangxi/10950/2014
AJJ93315 A/chicken/Jiangxi/10953/2014
AJJ93327 A/chicken/Jiangxi/10954/2014
AJJ93339 A/chicken/Jiangxi/10955/2014
AJJ93351 A/chicken/Jiangxi/10956/2014
AJJ93363 A/chicken/Jiangxi/10957/2014
AJJ93375 A/chicken/Jiangxi/10958/2014
AJJ93387 A/chicken/Jiangxi/10959/2014
AJJ93399 A/chicken/Jiangxi/10961/2014
AJJ93411 A/chicken/Jiangxi/10962/2014
AJJ93423 A/chicken/Jiangxi/10963/2014
AJJ93435 A/chicken/Jiangxi/10964/2014
AJJ93447 A/chicken/Jiangxi/10965/2014
AJJ96805 A/chicken/Jiangxi/12200/2014
AJJ97063 A/chicken/Jiangxi/12201/2014
AJJ96552 A/chicken/Jiangxi/12206/2014
AJJ97075 A/chicken/Jiangxi/12208/2014
AJJ96564 A/chicken/Jiangxi/12210/2014

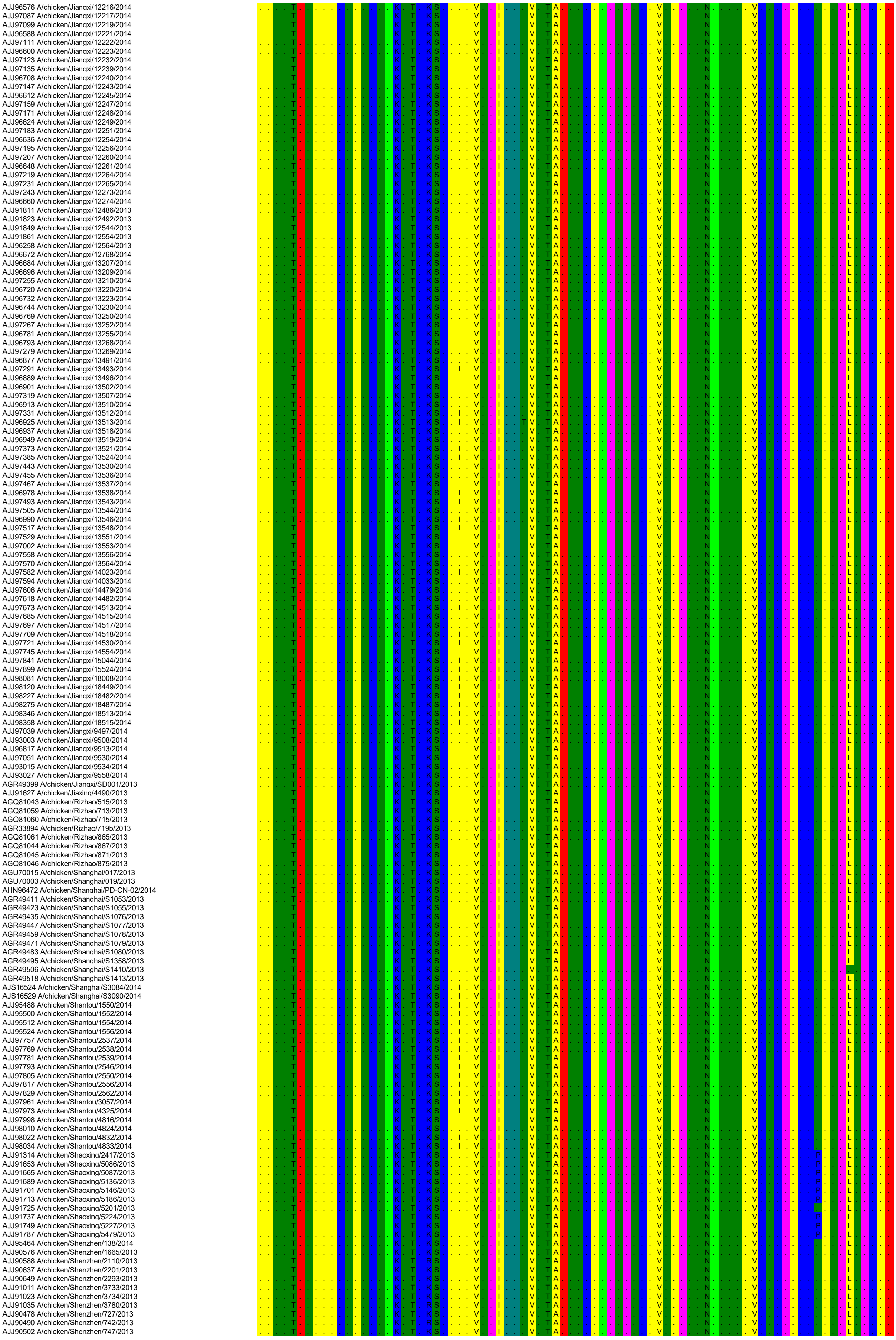
AJJ96576 A/chicken/Jiangxi/12216/2014
AJJ97087 A/chicken/Jiangxi/12217/2014
AJJ97099 A/chicken/Jiangxi/12219/2014
AJJ96588 A/chicken/Jiangxi/12221/2014
AJJ97111 A/chicken/Jiangxi/12222/2014
AJJ96600 A/chicken/Jiangxi/12223/2014
AJJ97123 A/chicken/Jiangxi/12232/2014
AJJ97135 A/chicken/Jiangxi/12239/2014
AJJ96708 A/chicken/Jiangxi/12240/2014
AJJ97147 A/chicken/Jiangxi/12243/2014
AJJ96612 A/chicken/Jiangxi/12245/2014
AJJ97159 A/chicken/Jiangxi/12247/2014
AJJ97171 A/chicken/Jiangxi/12248/2014
AJJ96624 A/chicken/Jiangxi/12249/2014
AJJ97183 A/chicken/Jiangxi/12251/2014
AJJ96636 A/chicken/Jiangxi/12254/2014
AJJ97195 A/chicken/Jiangxi/12256/2014
AJJ97207 A/chicken/Jiangxi/12260/2014
AJJ96648 A/chicken/Jiangxi/12261/2014
AJJ97219 A/chicken/Jiangxi/12264/2014
AJJ97231 A/chicken/Jiangxi/12265/2014
AJJ97243 A/chicken/Jiangxi/12273/2014
AJJ96660 A/chicken/Jiangxi/12274/2014
AJJ91811 A/chicken/Jiangxi/12486/2013
AJJ91823 A/chicken/Jiangxi/12492/2013
AJJ91849 A/chicken/Jiangxi/12544/2013
AJJ91861 A/chicken/Jiangxi/12554/2013
AJJ96258 A/chicken/Jiangxi/12564/2013
AJJ96672 A/chicken/Jiangxi/12768/2014
AJJ96684 A/chicken/Jiangxi/13207/2014
AJJ96696 A/chicken/Jiangxi/13209/2014
AJJ97255 A/chicken/Jiangxi/13210/2014
AJJ96720 A/chicken/Jiangxi/13220/2014
AJJ96732 A/chicken/Jiangxi/13223/2014
AJJ96744 A/chicken/Jiangxi/13230/2014
AJJ96769 A/chicken/Jiangxi/13250/2014
AJJ97267 A/chicken/Jiangxi/13252/2014
AJJ96781 A/chicken/Jiangxi/13255/2014
AJJ96793 A/chicken/Jiangxi/13268/2014
AJJ97279 A/chicken/Jiangxi/13269/2014
AJJ96877 A/chicken/Jiangxi/13491/2014
AJJ97291 A/chicken/Jiangxi/13493/2014
AJJ96889 A/chicken/Jiangxi/13496/2014
AJJ96901 A/chicken/Jiangxi/13502/2014
AJJ97319 A/chicken/Jiangxi/13507/2014
AJJ96913 A/chicken/Jiangxi/13510/2014
AJJ97331 A/chicken/Jiangxi/13512/2014
AJJ96925 A/chicken/Jiangxi/13513/2014
AJJ96937 A/chicken/Jiangxi/13518/2014
AJJ96949 A/chicken/Jiangxi/13519/2014
AJJ97373 A/chicken/Jiangxi/13521/2014
AJJ97385 A/chicken/Jiangxi/13524/2014
AJJ97443 A/chicken/Jiangxi/13530/2014
AJJ97455 A/chicken/Jiangxi/13536/2014
AJJ97467 A/chicken/Jiangxi/13537/2014
AJJ96978 A/chicken/Jiangxi/13538/2014
AJJ97493 A/chicken/Jiangxi/13543/2014
AJJ97505 A/chicken/Jiangxi/13544/2014
AJJ96990 A/chicken/Jiangxi/13546/2014
AJJ97517 A/chicken/Jiangxi/13548/2014
AJJ97529 A/chicken/Jiangxi/13551/2014
AJJ97002 A/chicken/Jiangxi/13553/2014
AJJ97558 A/chicken/Jiangxi/13556/2014
AJJ97570 A/chicken/Jiangxi/13564/2014
AJJ97582 A/chicken/Jiangxi/14023/2014
AJJ97594 A/chicken/Jiangxi/14033/2014
AJJ97606 A/chicken/Jiangxi/14479/2014
AJJ97618 A/chicken/Jiangxi/14482/2014
AJJ97673 A/chicken/Jiangxi/14513/2014
AJJ97685 A/chicken/Jiangxi/14515/2014
AJJ97697 A/chicken/Jiangxi/14517/2014
AJJ97709 A/chicken/Jiangxi/14518/2014
AJJ97721 A/chicken/Jiangxi/14530/2014
AJJ97745 A/chicken/Jiangxi/14554/2014
AJJ97841 A/chicken/Jiangxi/15044/2014
AJJ97899 A/chicken/Jiangxi/15524/2014
AJJ98081 A/chicken/Jiangxi/18008/2014
AJJ98120 A/chicken/Jiangxi/18449/2014
AJJ98227 A/chicken/Jiangxi/18482/2014
AJJ98275 A/chicken/Jiangxi/18487/2014
AJJ98346 A/chicken/Jiangxi/18513/2014
AJJ98358 A/chicken/Jiangxi/18515/2014
AJJ97039 A/chicken/Jiangxi/9497/2014
AJJ93003 A/chicken/Jiangxi/9508/2014
AJJ96817 A/chicken/Jiangxi/9513/2014
AJJ97051 A/chicken/Jiangxi/9530/2014
AJJ93015 A/chicken/Jiangxi/9534/2014
AJJ93027 A/chicken/Jiangxi/9558/2014
AGR49399 A/chicken/Jiangxi/SD001/2013
AJJ91627 A/chicken/Jiaxing/4490/2013
AGQ81043 A/chicken/Rizhao/515/2013
AGQ81059 A/chicken/Rizhao/713/2013
AGQ81060 A/chicken/Rizhao/715/2013
AGR33894 A/chicken/Rizhao/719b/2013
AGQ81061 A/chicken/Rizhao/865/2013
AGQ81044 A/chicken/Rizhao/867/2013
AGQ81045 A/chicken/Rizhao/871/2013
AGQ81046 A/chicken/Rizhao/875/2013
AGU70015 A/chicken/Shanghai/017/2013
AGU70003 A/chicken/Shanghai/019/2013
AHN96472 A/chicken/Shanghai/PD-CN-02/2014
AGR49411 A/chicken/Shanghai/S1053/2013
AGR49423 A/chicken/Shanghai/S1055/2013
AGR49435 A/chicken/Shanghai/S1076/2013
AGR49447 A/chicken/Shanghai/S1077/2013
AGR49459 A/chicken/Shanghai/S1078/2013
AGR49471 A/chicken/Shanghai/S1079/2013
AGR49483 A/chicken/Shanghai/S1080/2013
AGR49495 A/chicken/Shanghai/S1358/2013
AGR49506 A/chicken/Shanghai/S1410/2013
AGR49518 A/chicken/Shanghai/S1413/2013
AJS16524 A/chicken/Shanghai/S3084/2014
AJS16529 A/chicken/Shanghai/S3090/2014
AJJ95488 A/chicken/Shantou/1550/2014
AJJ95500 A/chicken/Shantou/1552/2014
AJJ95512 A/chicken/Shantou/1554/2014
AJJ95524 A/chicken/Shantou/1556/2014
AJJ97757 A/chicken/Shantou/2537/2014
AJJ97769 A/chicken/Shantou/2538/2014
AJJ97781 A/chicken/Shantou/2539/2014
AJJ97793 A/chicken/Shantou/2546/2014
AJJ97805 A/chicken/Shantou/2550/2014
AJJ97817 A/chicken/Shantou/2556/2014
AJJ97829 A/chicken/Shantou/2562/2014
AJJ97961 A/chicken/Shantou/3057/2014
AJJ97973 A/chicken/Shantou/4325/2014
AJJ97998 A/chicken/Shantou/4816/2014
AJJ98010 A/chicken/Shantou/4824/2014
AJJ98022 A/chicken/Shantou/4832/2014
AJJ98034 A/chicken/Shantou/4833/2014
AJJ91314 A/chicken/Shaoxing/2417/2013
AJJ91653 A/chicken/Shaoxing/5086/2013
AJJ91665 A/chicken/Shaoxing/5087/2013
AJJ91689 A/chicken/Shaoxing/5136/2013
AJJ91701 A/chicken/Shaoxing/5146/2013
AJJ91713 A/chicken/Shaoxing/5186/2013
AJJ91725 A/chicken/Shaoxing/5201/2013
AJJ91737 A/chicken/Shaoxing/5224/2013
AJJ91749 A/chicken/Shaoxing/5227/2013
AJJ91787 A/chicken/Shaoxing/5479/2013
AJJ95464 A/chicken/Shenzhen/138/2014
AJJ90576 A/chicken/Shenzhen/1665/2013
AJJ90588 A/chicken/Shenzhen/2110/2013
AJJ90637 A/chicken/Shenzhen/2201/2013
AJJ90649 A/chicken/Shenzhen/2293/2013
AJJ91011 A/chicken/Shenzhen/3733/2013
AJJ91023 A/chicken/Shenzhen/3734/2013
AJJ91035 A/chicken/Shenzhen/3780/2013
AJJ90478 A/chicken/Shenzhen/727/2013
AJJ90490 A/chicken/Shenzhen/742/2013
AJJ90502 A/chicken/Shenzhen/747/2013

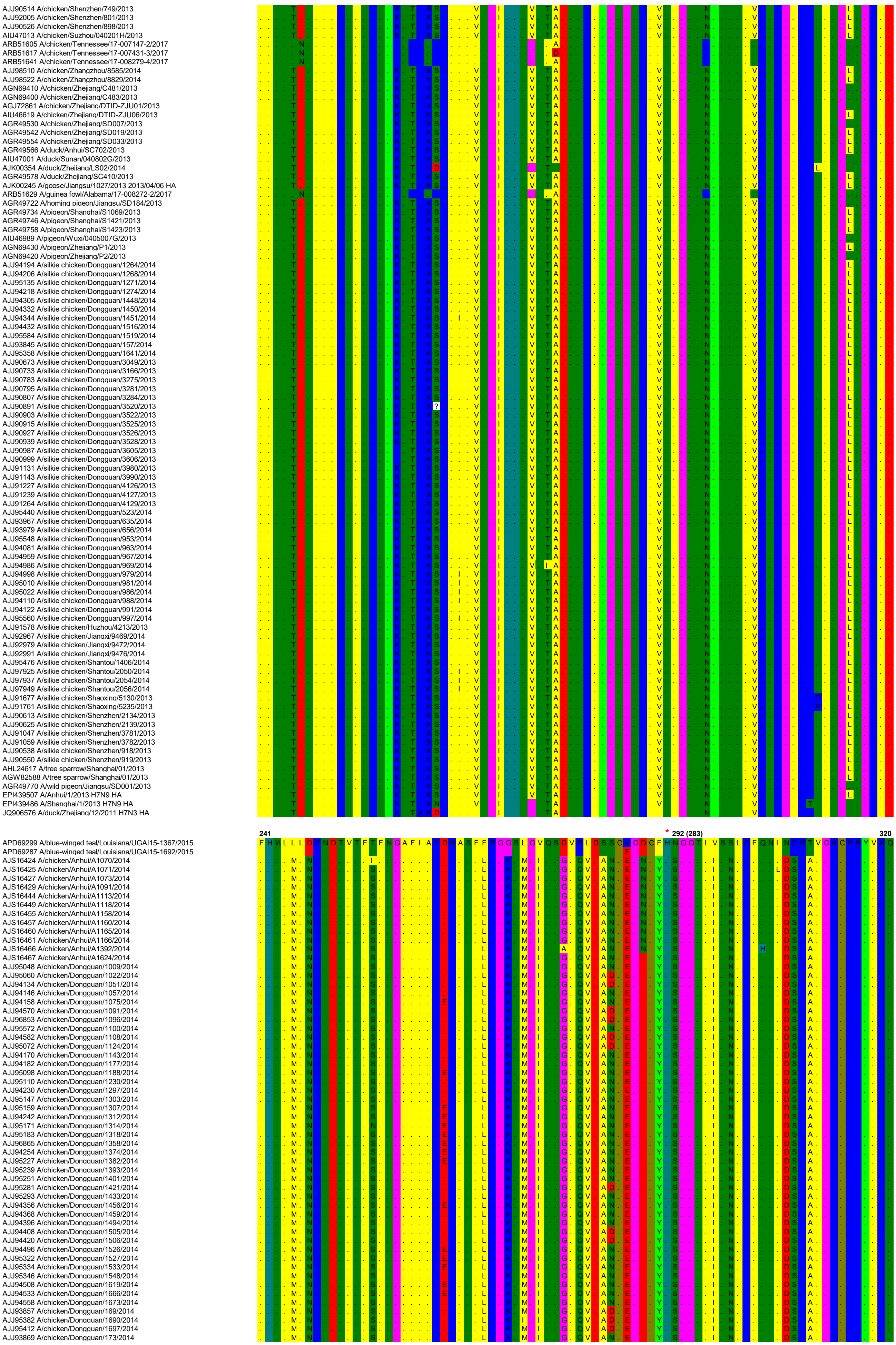
AJJ90514 A/chicken/Shenzhen/749/2013
AJJ92005 A/chicken/Shenzhen/801/2013
AJJ90526 A/chicken/Shenzhen/898/2013
AIU47013 A/chicken/Suzhou/040201H/2013
ARB51605 A/chicken/Tennessee/17-007147-2/2017
ARB51617 A/chicken/Tennessee/17-007431-3/2017
ARB51641 A/chicken/Tennessee/17-008279-4/2017
AJJ98510 A/chicken/Zhangzhou/8585/2014
AJJ98522 A/chicken/Zhangzhou/8829/2014
AGN69410 A/chicken/Zhejiang/C481/2013
AGN69400 A/chicken/Zhejiang/C483/2013
AGJ72861 A/chicken/Zhejiang/DTID-ZJU01/2013
AIU46619 A/chicken/Zhejiang/DTID-ZJU06/2013
AGR49530 A/chicken/Zhejiang/SD007/2013
AGR49542 A/chicken/Zhejiang/SD019/2013
AGR49554 A/chicken/Zhejiang/SD033/2013
AGR49566 A/duck/Anhui/SC702/2013
AIU47001 A/duck/Sunan/040802G/2013
AJK00354 A/duck/Zhejiang/LS02/2014
AGR49578 A/duck/Zhejiang/SC410/2013
AJK00245 A/goose/Jiangsu/1027/2013 2013/04/06 HA
ARB51629 A/guinea fowl/Alabama/17-008272-2/2017
AGR49722 A/homing pigeon/Jiangsu/SD184/2013
AGR49734 A/pigeon/Shanghai/S1069/2013
AGR49746 A/pigeon/Shanghai/S1421/2013
AGR49758 A/pigeon/Shanghai/S1423/2013
AIU46989 A/pigeon/Wuxi/0405007G/2013
AGN69430 A/pigeon/Zhejiang/P1/2013
AGN69420 A/pigeon/Zhejiang/P2/2013
AJJ94194 A/silkie chicken/Dongguan/1264/2014
AJJ94206 A/silkie chicken/Dongguan/1268/2014
AJJ95135 A/silkie chicken/Dongguan/1271/2014
AJJ94218 A/silkie chicken/Dongguan/1274/2014
AJJ94305 A/silkie chicken/Dongguan/1448/2014
AJJ94332 A/silkie chicken/Dongguan/1450/2014
AJJ94344 A/silkie chicken/Dongguan/1451/2014
AJJ94432 A/silkie chicken/Dongguan/1516/2014
AJJ95584 A/silkie chicken/Dongguan/1519/2014
AJJ93845 A/silkie chicken/Dongguan/157/2014
AJJ95358 A/silkie chicken/Dongguan/1641/2014
AJJ90673 A/silkie chicken/Dongguan/3049/2013
AJJ90733 A/silkie chicken/Dongguan/3166/2013
AJJ90783 A/silkie chicken/Dongguan/3275/2013
AJJ90795 A/silkie chicken/Dongguan/3281/2013
AJJ90807 A/silkie chicken/Dongguan/3284/2013
AJJ90891 A/silkie chicken/Dongguan/3520/2013
AJJ90903 A/silkie chicken/Dongguan/3522/2013
AJJ90915 A/silkie chicken/Dongguan/3525/2013
AJJ90927 A/silkie chicken/Dongguan/3526/2013
AJJ90939 A/silkie chicken/Dongguan/3528/2013
AJJ90987 A/silkie chicken/Dongguan/3605/2013
AJJ90999 A/silkie chicken/Dongguan/3606/2013
AJJ91131 A/silkie chicken/Dongguan/3980/2013
AJJ91143 A/silkie chicken/Dongguan/3990/2013
AJJ91227 A/silkie chicken/Dongguan/4126/2013
AJJ91239 A/silkie chicken/Dongguan/4127/2013
AJJ91264 A/silkie chicken/Dongguan/4129/2013
AJJ95440 A/silkie chicken/Dongguan/523/2014
AJJ93967 A/silkie chicken/Dongguan/635/2014
AJJ93979 A/silkie chicken/Dongguan/656/2014
AJJ95548 A/silkie chicken/Dongguan/953/2014
AJJ94081 A/silkie chicken/Dongguan/963/2014
AJJ94959 A/silkie chicken/Dongguan/967/2014
AJJ94986 A/silkie chicken/Dongguan/969/2014
AJJ94998 A/silkie chicken/Dongguan/979/2014
AJJ95010 A/silkie chicken/Dongguan/981/2014
AJJ95022 A/silkie chicken/Dongguan/986/2014
AJJ94110 A/silkie chicken/Dongguan/988/2014
AJJ94122 A/silkie chicken/Dongguan/991/2014
AJJ95560 A/silkie chicken/Dongguan/997/2014
AJJ91578 A/silkie chicken/Huzhou/4213/2013
AJJ92967 A/silkie chicken/Jiangxi/9469/2014
AJJ92979 A/silkie chicken/Jiangxi/9472/2014
AJJ92991 A/silkie chicken/Jiangxi/9476/2014
AJJ95476 A/silkie chicken/Shantou/1406/2014
AJJ97925 A/silkie chicken/Shantou/2050/2014
AJJ97937 A/silkie chicken/Shantou/2054/2014
AJJ97949 A/silkie chicken/Shantou/2056/2014
AJJ91677 A/silkie chicken/Shaoxing/5130/2013
AJJ91761 A/silkie chicken/Shaoxing/5235/2013
AJJ90613 A/silkie chicken/Shenzhen/2134/2013
AJJ90625 A/silkie chicken/Shenzhen/2139/2013
AJJ91047 A/silkie chicken/Shenzhen/3781/2013
AJJ91059 A/silkie chicken/Shenzhen/3782/2013
AJJ90538 A/silkie chicken/Shenzhen/918/2013
AJJ90550 A/silkie chicken/Shenzhen/919/2013
AHL24617 A/tree sparrow/Shanghai/01/2013
AGW82588 A/tree sparrow/Shanghai/01/2013
AGR49770 A/wild pigeon/Jiangsu/SD001/2013
EPI439507 A/Anhui/1/2013 H7N9 HA
EPI439486 A/Shanghai/1/2013 H7N9 HA
JQ906576 A/duck/Zhejiang/12/2011 H7N3 HA
241
* 292 (283)
320
APD69299 A/blue-winged teal/Louisiana/UGAI15-1367/2015
APD69287 A/blue-winged teal/Louisiana/UGAI15-1692/2015
AJS16424 A/chicken/Anhui/A1070/2014
AJS16425 A/chicken/Anhui/A1071/2014
AJS16427 A/chicken/Anhui/A1073/2014
AJS16429 A/chicken/Anhui/A1091/2014
AJS16444 A/chicken/Anhui/A1113/2014
AJS16449 A/chicken/Anhui/A1118/2014
AJS16455 A/chicken/Anhui/A1158/2014
AJS16457 A/chicken/Anhui/A1160/2014
AJS16460 A/chicken/Anhui/A1165/2014
AJS16461 A/chicken/Anhui/A1166/2014
AJS16466 A/chicken/Anhui/A1392/2014
AJS16467 A/chicken/Anhui/A1624/2014
AJJ95048 A/chicken/Dongguan/1009/2014
AJJ95060 A/chicken/Dongguan/1022/2014
AJJ94134 A/chicken/Dongguan/1051/2014
AJJ94146 A/chicken/Dongguan/1057/2014
AJJ94158 A/chicken/Dongguan/1075/2014
AJJ94570 A/chicken/Dongguan/1091/2014
AJJ96853 A/chicken/Dongguan/1096/2014
AJJ95572 A/chicken/Dongguan/1100/2014
AJJ94582 A/chicken/Dongguan/1108/2014
AJJ95072 A/chicken/Dongguan/1124/2014
AJJ94170 A/chicken/Dongguan/1143/2014
AJJ94182 A/chicken/Dongguan/1177/2014
AJJ95098 A/chicken/Dongguan/1188/2014
AJJ95110 A/chicken/Dongguan/1230/2014
AJJ94230 A/chicken/Dongguan/1297/2014
AJJ95147 A/chicken/Dongguan/1303/2014
AJJ95159 A/chicken/Dongguan/1307/2014
AJJ94242 A/chicken/Dongguan/1312/2014
AJJ95171 A/chicken/Dongguan/1314/2014
AJJ95183 A/chicken/Dongguan/1318/2014
AJJ96865 A/chicken/Dongguan/1358/2014
AJJ94254 A/chicken/Dongguan/1374/2014
AJJ95227 A/chicken/Dongguan/1382/2014
AJJ95239 A/chicken/Dongguan/1393/2014
AJJ95251 A/chicken/Dongguan/1401/2014
AJJ95281 A/chicken/Dongguan/1421/2014
AJJ95293 A/chicken/Dongguan/1433/2014
AJJ94356 A/chicken/Dongguan/1456/2014
AJJ94368 A/chicken/Dongguan/1459/2014
AJJ94396 A/chicken/Dongguan/1494/2014
AJJ94408 A/chicken/Dongguan/1505/2014
AJJ94420 A/chicken/Dongguan/1506/2014
AJJ94496 A/chicken/Dongguan/1526/2014
AJJ95322 A/chicken/Dongguan/1527/2014
AJJ95334 A/chicken/Dongguan/1533/2014
AJJ95346 A/chicken/Dongguan/1548/2014
AJJ94508 A/chicken/Dongguan/1619/2014
AJJ94533 A/chicken/Dongguan/1666/2014
AJJ94558 A/chicken/Dongguan/1673/2014
AJJ93857 A/chicken/Dongguan/169/2014
AJJ95382 A/chicken/Dongguan/1690/2014
AJJ95412 A/chicken/Dongguan/1697/2014
AJJ93869 A/chicken/Dongguan/173/2014

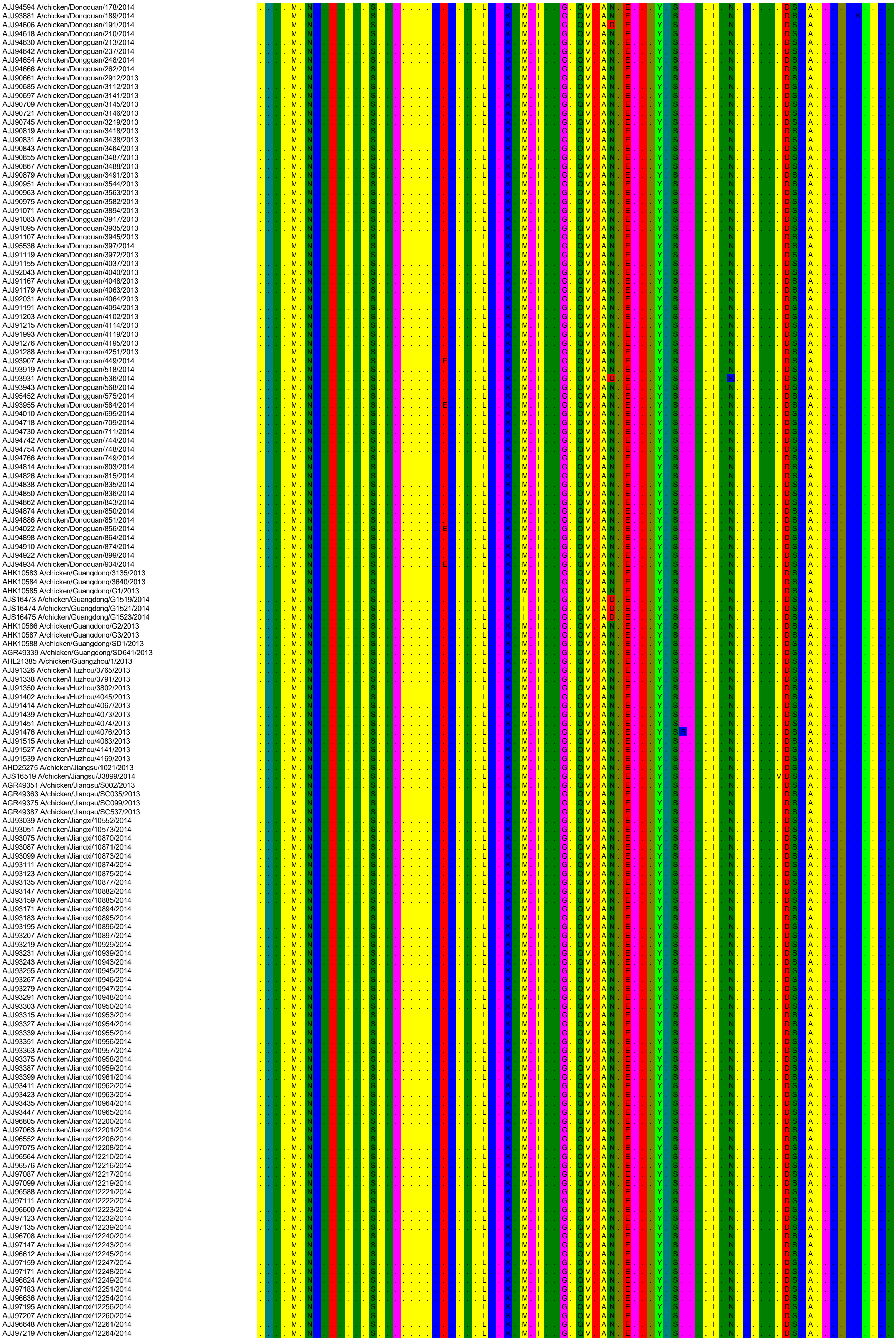

AJJ94594 A/chicken/Dongguan/178/2014
AJJ93881 A/chicken/Dongguan/189/2014
AJJ94606 A/chicken/Dongguan/191/2014
AJJ94618 A/chicken/Dongguan/210/2014
AJJ94630 A/chicken/Dongguan/213/2014
AJJ94642 A/chicken/Dongguan/237/2014
AJJ94654 A/chicken/Dongguan/248/2014
AJJ94666 A/chicken/Dongguan/262/2014
AJJ90661 A/chicken/Dongguan/2912/2013
AJJ90685 A/chicken/Dongguan/3112/2013
AJJ90697 A/chicken/Dongguan/3141/2013
AJJ90709 A/chicken/Dongguan/3145/2013
AJJ90721 A/chicken/Dongguan/3146/2013
AJJ90745 A/chicken/Dongguan/3219/2013
AJJ90819 A/chicken/Dongguan/3418/2013
AJJ90831 A/chicken/Dongguan/3438/2013
AJJ90843 A/chicken/Dongguan/3464/2013
AJJ90855 A/chicken/Dongguan/3487/2013
AJJ90867 A/chicken/Dongguan/3488/2013
AJJ90879 A/chicken/Dongguan/3491/2013
AJJ90951 A/chicken/Dongguan/3544/2013
AJJ90963 A/chicken/Dongguan/3563/2013
AJJ90975 A/chicken/Dongguan/3582/2013
AJJ91071 A/chicken/Dongguan/3894/2013
AJJ91083 A/chicken/Dongguan/3917/2013
AJJ91095 A/chicken/Dongguan/3935/2013
AJJ91107 A/chicken/Dongguan/3945/2013
AJJ95536 A/chicken/Dongguan/397/2014
AJJ91119 A/chicken/Dongguan/3972/2013
AJJ91155 A/chicken/Dongguan/4037/2013
AJJ92043 A/chicken/Dongguan/4040/2013
AJJ91167 A/chicken/Dongguan/4048/2013
AJJ91179 A/chicken/Dongguan/4063/2013
AJJ92031 A/chicken/Dongguan/4064/2013
AJJ91191 A/chicken/Dongguan/4094/2013
AJJ91203 A/chicken/Dongguan/4102/2013
AJJ91215 A/chicken/Dongguan/4114/2013
AJJ91993 A/chicken/Dongguan/4119/2013
AJJ91276 A/chicken/Dongguan/4195/2013
AJJ91288 A/chicken/Dongguan/4251/2013
AJJ93907 A/chicken/Dongguan/449/2014
AJJ93919 A/chicken/Dongguan/518/2014
AJJ93931 A/chicken/Dongguan/536/2014
AJJ93943 A/chicken/Dongguan/568/2014
AJJ95452 A/chicken/Dongguan/575/2014
AJJ93955 A/chicken/Dongguan/584/2014
AJJ94010 A/chicken/Dongguan/695/2014
AJJ94718 A/chicken/Dongguan/709/2014
AJJ94730 A/chicken/Dongguan/711/2014
AJJ94742 A/chicken/Dongguan/744/2014
AJJ94754 A/chicken/Dongguan/748/2014
AJJ94766 A/chicken/Dongguan/749/2014
AJJ94814 A/chicken/Dongguan/803/2014
AJJ94826 A/chicken/Dongguan/815/2014
AJJ94838 A/chicken/Dongguan/835/2014
AJJ94850 A/chicken/Dongguan/836/2014
AJJ94862 A/chicken/Dongguan/843/2014
AJJ94874 A/chicken/Dongguan/850/2014
AJJ94886 A/chicken/Dongguan/851/2014
AJJ94022 A/chicken/Dongguan/856/2014
AJJ94898 A/chicken/Dongguan/864/2014
AJJ94910 A/chicken/Dongguan/874/2014
AJJ94922 A/chicken/Dongguan/899/2014
AJJ94934 A/chicken/Dongguan/934/2014
AHK10583 A/chicken/Guangdong/3135/2013
AHK10584 A/chicken/Guangdong/3640/2013
AHK10585 A/chicken/Guangdong/G1/2013
AJS16473 A/chicken/Guangdong/G1519/2014
AJS16474 A/chicken/Guangdong/G1521/2014
AJS16475 A/chicken/Guangdong/G1523/2014
AHK10586 A/chicken/Guangdong/G2/2013
AHK10587 A/chicken/Guangdong/G3/2013
AHK10588 A/chicken/Guangdong/SD1/2013
AGR49339 A/chicken/Guangdong/SD641/2013
AHL21385 A/chicken/Guangzhou/1/2013
AJJ91326 A/chicken/Huzhou/3765/2013
AJJ91338 A/chicken/Huzhou/3791/2013
AJJ91350 A/chicken/Huzhou/3802/2013
AJJ91402 A/chicken/Huzhou/4045/2013
AJJ91414 A/chicken/Huzhou/4067/2013
AJJ91439 A/chicken/Huzhou/4073/2013
AJJ91451 A/chicken/Huzhou/4074/2013
AJJ91476 A/chicken/Huzhou/4076/2013
AJJ91515 A/chicken/Huzhou/4083/2013
AJJ91527 A/chicken/Huzhou/4141/2013
AJJ91539 A/chicken/Huzhou/4169/2013
AHD25275 A/chicken/Jiangsu/1021/2013
AJS16519 A/chicken/Jiangsu/J3899/2014
AGR49351 A/chicken/Jiangsu/S002/2013
AGR49363 A/chicken/Jiangsu/SC035/2013
AGR49375 A/chicken/Jiangsu/SC099/2013
AGR49387 A/chicken/Jiangsu/SC537/2013
AJJ93039 A/chicken/Jiangxi/10552/2014
AJJ93051 A/chicken/Jiangxi/10573/2014
AJJ93075 A/chicken/Jiangxi/10870/2014
AJJ93087 A/chicken/Jiangxi/10871/2014
AJJ93099 A/chicken/Jiangxi/10873/2014
AJJ93111 A/chicken/Jiangxi/10874/2014
AJJ93123 A/chicken/Jiangxi/10875/2014
AJJ93135 A/chicken/Jiangxi/10877/2014
AJJ93147 A/chicken/Jiangxi/10882/2014
AJJ93159 A/chicken/Jiangxi/10885/2014
AJJ93171 A/chicken/Jiangxi/10894/2014
AJJ93183 A/chicken/Jiangxi/10895/2014
AJJ93195 A/chicken/Jiangxi/10896/2014
AJJ93207 A/chicken/Jiangxi/10897/2014
AJJ93219 A/chicken/Jiangxi/10929/2014
AJJ93231 A/chicken/Jiangxi/10939/2014
AJJ93243 A/chicken/Jiangxi/10943/2014
AJJ93255 A/chicken/Jiangxi/10945/2014
AJJ93267 A/chicken/Jiangxi/10946/2014
AJJ93279 A/chicken/Jiangxi/10947/2014
AJJ93291 A/chicken/Jiangxi/10948/2014
AJJ93303 A/chicken/Jiangxi/10950/2014
AJJ93315 A/chicken/Jiangxi/10953/2014
AJJ93327 A/chicken/Jiangxi/10954/2014
AJJ93339 A/chicken/Jiangxi/10955/2014
AJJ93351 A/chicken/Jiangxi/10956/2014
AJJ93363 A/chicken/Jiangxi/10957/2014
AJJ93375 A/chicken/Jiangxi/10958/2014
AJJ93387 A/chicken/Jiangxi/10959/2014
AJJ93399 A/chicken/Jiangxi/10961/2014
AJJ93411 A/chicken/Jiangxi/10962/2014
AJJ93423 A/chicken/Jiangxi/10963/2014
AJJ93435 A/chicken/Jiangxi/10964/2014
AJJ93447 A/chicken/Jiangxi/10965/2014
AJJ96805 A/chicken/Jiangxi/12200/2014
AJJ97063 A/chicken/Jiangxi/12201/2014
AJJ96552 A/chicken/Jiangxi/12206/2014
AJJ97075 A/chicken/Jiangxi/12208/2014
AJJ96564 A/chicken/Jiangxi/12210/2014
AJJ96576 A/chicken/Jiangxi/12216/2014
AJJ97087 A/chicken/Jiangxi/12217/2014
AJJ97099 A/chicken/Jiangxi/12219/2014
AJJ96588 A/chicken/Jiangxi/12221/2014
AJJ97111 A/chicken/Jiangxi/12222/2014
AJJ96600 A/chicken/Jiangxi/12223/2014
AJJ97123 A/chicken/Jiangxi/12232/2014
AJJ97135 A/chicken/Jiangxi/12239/2014
AJJ96708 A/chicken/Jiangxi/12240/2014
AJJ97147 A/chicken/Jiangxi/12243/2014
AJJ96612 A/chicken/Jiangxi/12245/2014
AJJ97159 A/chicken/Jiangxi/12247/2014
AJJ97171 A/chicken/Jiangxi/12248/2014
AJJ96624 A/chicken/Jiangxi/12249/2014
AJJ97183 A/chicken/Jiangxi/12251/2014
AJJ96636 A/chicken/Jiangxi/12254/2014
AJJ97195 A/chicken/Jiangxi/12256/2014
AJJ97207 A/chicken/Jiangxi/12260/2014
AJJ96648 A/chicken/Jiangxi/12261/2014
AJJ97219 A/chicken/Jiangxi/12264/2014

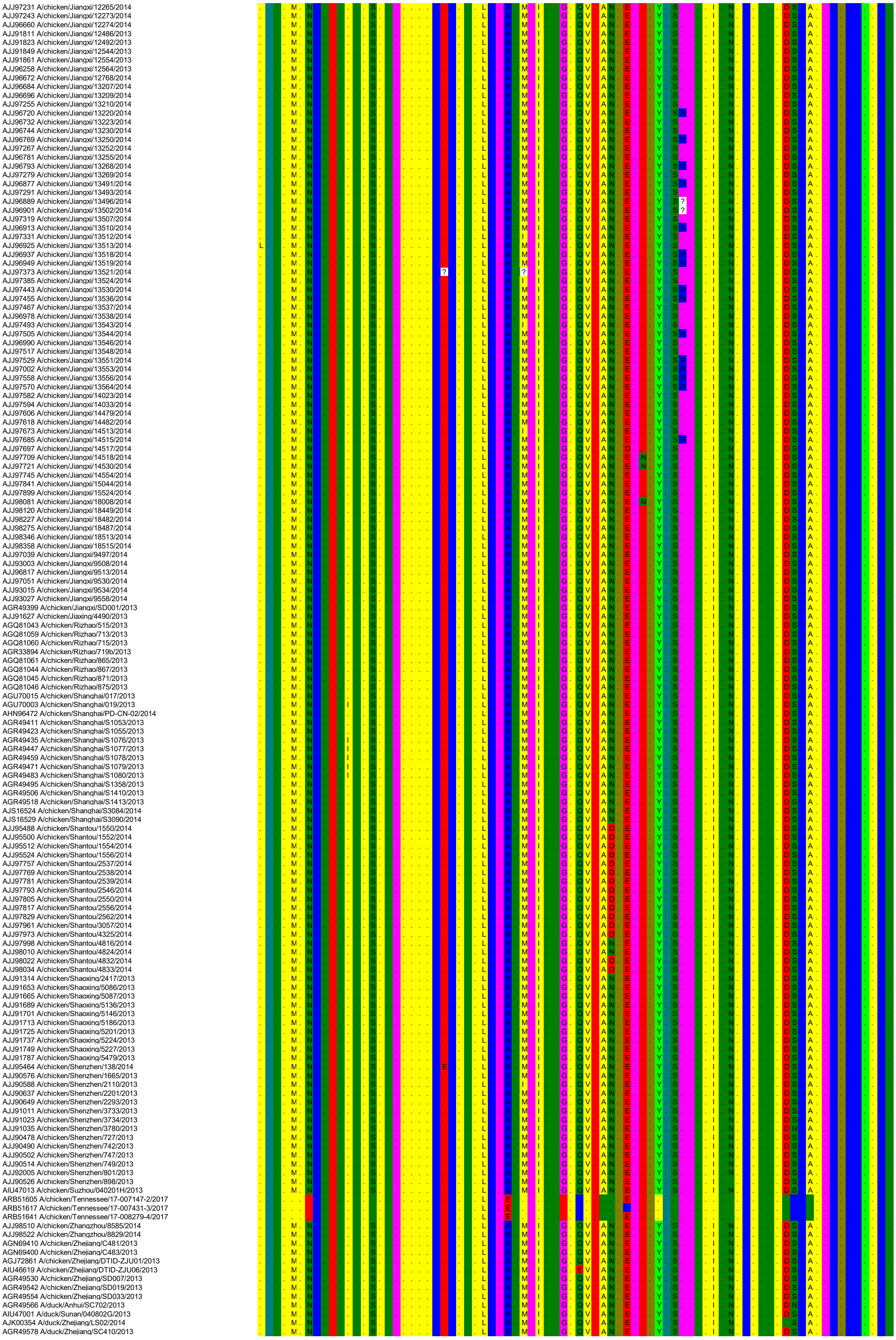

AJJ97231 A/chicken/Jiangxi/12265/2014
AJJ97243 A/chicken/Jiangxi/12273/2014
AJJ96660 A/chicken/Jiangxi/12274/2014
AJJ91811 A/chicken/Jiangxi/12486/2013
AJJ91823 A/chicken/Jiangxi/12492/2013
AJJ91849 A/chicken/Jiangxi/12544/2013
AJJ91861 A/chicken/Jiangxi/12554/2013
AJJ96258 A/chicken/Jiangxi/12564/2013
AJJ96672 A/chicken/Jiangxi/12768/2014
AJJ96684 A/chicken/Jiangxi/13207/2014
AJJ96696 A/chicken/Jiangxi/13209/2014
AJJ97255 A/chicken/Jiangxi/13210/2014
AJJ96720 A/chicken/Jiangxi/13220/2014
AJJ96732 A/chicken/Jiangxi/13223/2014
AJJ96744 A/chicken/Jiangxi/13230/2014
AJJ96769 A/chicken/Jiangxi/13250/2014
AJJ97267 A/chicken/Jiangxi/13252/2014
AJJ96781 A/chicken/Jiangxi/13255/2014
AJJ96793 A/chicken/Jiangxi/13268/2014
AJJ97279 A/chicken/Jiangxi/13269/2014
AJJ96877 A/chicken/Jiangxi/13491/2014
AJJ97291 A/chicken/Jiangxi/13493/2014
AJJ96889 A/chicken/Jiangxi/13496/2014
AJJ96901 A/chicken/Jiangxi/13502/2014
AJJ97319 A/chicken/Jiangxi/13507/2014
AJJ96913 A/chicken/Jiangxi/13510/2014
AJJ97331 A/chicken/Jiangxi/13512/2014
AJJ96925 A/chicken/Jiangxi/13513/2014
AJJ96937 A/chicken/Jiangxi/13518/2014
AJJ96949 A/chicken/Jiangxi/13519/2014
AJJ97373 A/chicken/Jiangxi/13521/2014
AJJ97385 A/chicken/Jiangxi/13524/2014
AJJ97443 A/chicken/Jiangxi/13530/2014
AJJ97455 A/chicken/Jiangxi/13536/2014
AJJ97467 A/chicken/Jiangxi/13537/2014
AJJ96978 A/chicken/Jiangxi/13538/2014
AJJ97493 A/chicken/Jiangxi/13543/2014
AJJ97505 A/chicken/Jiangxi/13544/2014
AJJ96990 A/chicken/Jiangxi/13546/2014
AJJ97517 A/chicken/Jiangxi/13548/2014
AJJ97529 A/chicken/Jiangxi/13551/2014
AJJ97002 A/chicken/Jiangxi/13553/2014
AJJ97558 A/chicken/Jiangxi/13556/2014
AJJ97570 A/chicken/Jiangxi/13564/2014
AJJ97582 A/chicken/Jiangxi/14023/2014
AJJ97594 A/chicken/Jiangxi/14033/2014
AJJ97606 A/chicken/Jiangxi/14479/2014
AJJ97618 A/chicken/Jiangxi/14482/2014
AJJ97673 A/chicken/Jiangxi/14513/2014
AJJ97685 A/chicken/Jiangxi/14515/2014
AJJ97697 A/chicken/Jiangxi/14517/2014
AJJ97709 A/chicken/Jiangxi/14518/2014
AJJ97721 A/chicken/Jiangxi/14530/2014
AJJ97745 A/chicken/Jiangxi/14554/2014
AJJ97841 A/chicken/Jiangxi/15044/2014
AJJ97899 A/chicken/Jiangxi/15524/2014
AJJ98081 A/chicken/Jiangxi/18008/2014
AJJ98120 A/chicken/Jiangxi/18449/2014
AJJ98227 A/chicken/Jiangxi/18482/2014
AJJ98275 A/chicken/Jiangxi/18487/2014
AJJ98346 A/chicken/Jiangxi/18513/2014
AJJ98358 A/chicken/Jiangxi/18515/2014
AJJ97039 A/chicken/Jiangxi/9497/2014
AJJ93003 A/chicken/Jiangxi/9508/2014
AJJ96817 A/chicken/Jiangxi/9513/2014
AJJ97051 A/chicken/Jiangxi/9530/2014
AJJ93015 A/chicken/Jiangxi/9534/2014
AJJ93027 A/chicken/Jiangxi/9558/2014
AGR49399 A/chicken/Jiangxi/SD001/2013
AJJ91627 A/chicken/Jiaxing/4490/2013
AGQ81043 A/chicken/Rizhao/515/2013
AGQ81059 A/chicken/Rizhao/713/2013
AGQ81060 A/chicken/Rizhao/715/2013
AGR33894 A/chicken/Rizhao/719b/2013
AGQ81061 A/chicken/Rizhao/865/2013
AGQ81044 A/chicken/Rizhao/867/2013
AGQ81045 A/chicken/Rizhao/871/2013
AGQ81046 A/chicken/Rizhao/875/2013
AGU70015 A/chicken/Shanghai/017/2013
AGU70003 A/chicken/Shanghai/019/2013
AHN96472 A/chicken/Shanghai/PD-CN-02/2014
AGR49411 A/chicken/Shanghai/S1053/2013
AGR49423 A/chicken/Shanghai/S1055/2013
AGR49435 A/chicken/Shanghai/S1076/2013
AGR49447 A/chicken/Shanghai/S1077/2013
AGR49459 A/chicken/Shanghai/S1078/2013
AGR49471 A/chicken/Shanghai/S1079/2013
AGR49483 A/chicken/Shanghai/S1080/2013
AGR49495 A/chicken/Shanghai/S1358/2013
AGR49506 A/chicken/Shanghai/S1410/2013
AGR49518 A/chicken/Shanghai/S1413/2013
AJS16524 A/chicken/Shanghai/S3084/2014
AJS16529 A/chicken/Shanghai/S3090/2014
AJJ95488 A/chicken/Shantou/1550/2014
AJJ95500 A/chicken/Shantou/1552/2014
AJJ95512 A/chicken/Shantou/1554/2014
AJJ95524 A/chicken/Shantou/1556/2014
AJJ97757 A/chicken/Shantou/2537/2014
AJJ97769 A/chicken/Shantou/2538/2014
AJJ97781 A/chicken/Shantou/2539/2014
AJJ97793 A/chicken/Shantou/2546/2014
AJJ97805 A/chicken/Shantou/2550/2014
AJJ97817 A/chicken/Shantou/2556/2014
AJJ97829 A/chicken/Shantou/2562/2014
AJJ97961 A/chicken/Shantou/3057/2014
AJJ97973 A/chicken/Shantou/4325/2014
AJJ97998 A/chicken/Shantou/4816/2014
AJJ98010 A/chicken/Shantou/4824/2014
AJJ98022 A/chicken/Shantou/4832/2014
AJJ98034 A/chicken/Shantou/4833/2014
AJJ91314 A/chicken/Shaoxing/2417/2013
AJJ91653 A/chicken/Shaoxing/5086/2013
AJJ91665 A/chicken/Shaoxing/5087/2013
AJJ91689 A/chicken/Shaoxing/5136/2013
AJJ91701 A/chicken/Shaoxing/5146/2013
AJJ91713 A/chicken/Shaoxing/5186/2013
AJJ91725 A/chicken/Shaoxing/5201/2013
AJJ91737 A/chicken/Shaoxing/5224/2013
AJJ91749 A/chicken/Shaoxing/5227/2013
AJJ91787 A/chicken/Shaoxing/5479/2013
AJJ95464 A/chicken/Shenzhen/138/2014
AJJ90576 A/chicken/Shenzhen/1665/2013
AJJ90588 A/chicken/Shenzhen/2110/2013
AJJ90637 A/chicken/Shenzhen/2201/2013
AJJ90649 A/chicken/Shenzhen/2293/2013
AJJ91011 A/chicken/Shenzhen/3733/2013
AJJ91023 A/chicken/Shenzhen/3734/2013
AJJ91035 A/chicken/Shenzhen/3780/2013
AJJ90478 A/chicken/Shenzhen/727/2013
AJJ90490 A/chicken/Shenzhen/742/2013
AJJ90502 A/chicken/Shenzhen/747/2013
AJJ90514 A/chicken/Shenzhen/749/2013
AJJ92005 A/chicken/Shenzhen/801/2013
AJJ90526 A/chicken/Shenzhen/898/2013
AIU47013 A/chicken/Suzhou/040201H/2013
ARB51605 A/chicken/Tennessee/17-007147-2/2017
ARB51617 A/chicken/Tennessee/17-007431-3/2017
ARB51641 A/chicken/Tennessee/17-008279-4/2017
AJJ98510 A/chicken/Zhangzhou/8585/2014
AJJ98522 A/chicken/Zhangzhou/8829/2014
AGN69410 A/chicken/Zhejiang/C481/2013
AGN69400 A/chicken/Zhejiang/C483/2013
AGJ72861 A/chicken/Zhejiang/DTID-ZJU01/2013
AIU46619 A/chicken/Zhejiang/DTID-ZJU06/2013
AGR49530 A/chicken/Zhejiang/SD007/2013
AGR49542 A/chicken/Zhejiang/SD019/2013
AGR49554 A/chicken/Zhejiang/SD033/2013
AGR49566 A/duck/Anhui/SC702/2013
AIU47001 A/duck/Sunan/040802G/2013
AJK00354 A/duck/Zhejiang/LS02/2014
AGR49578 A/duck/Zhejiang/SC410/2013

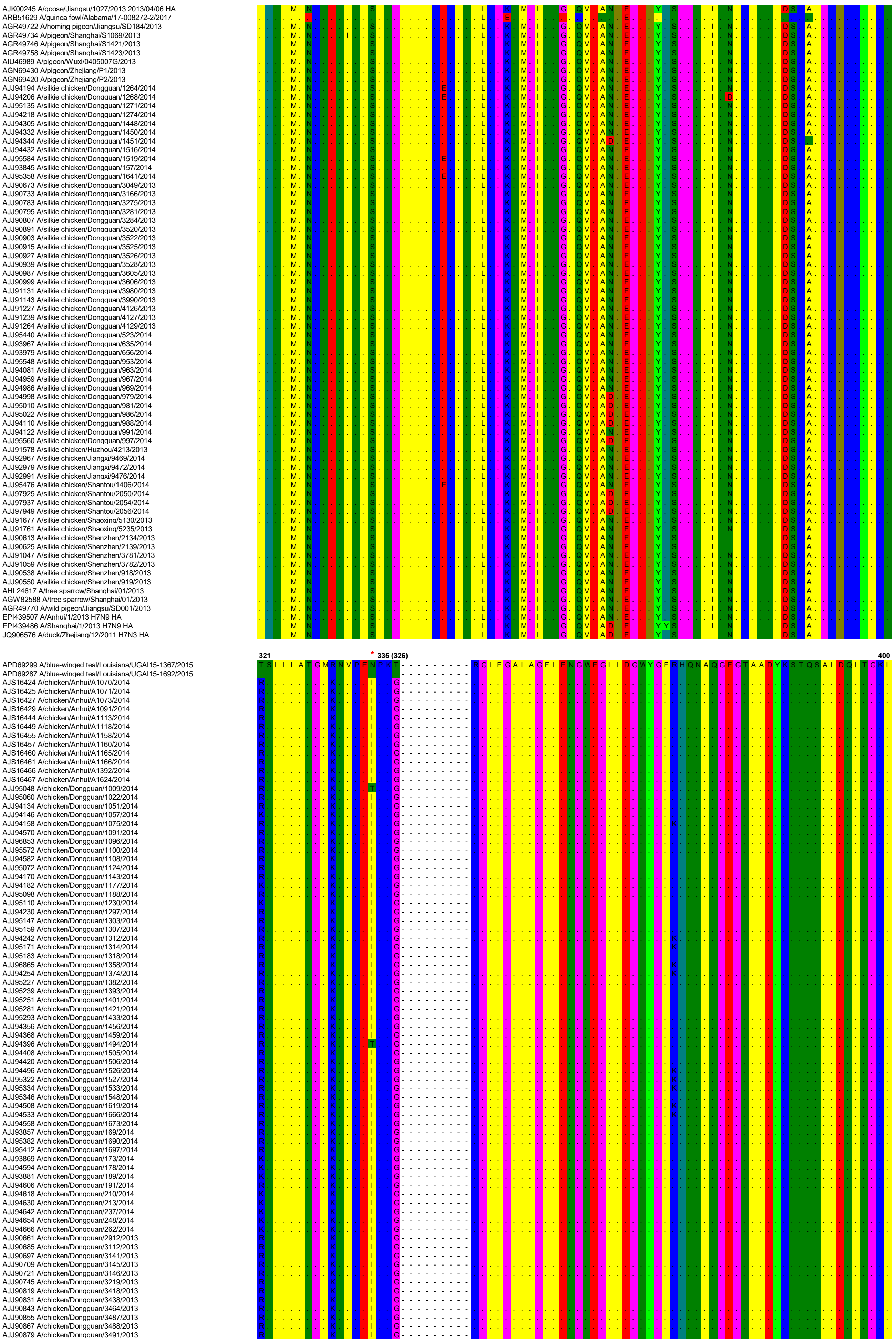
AJK00245 A/goose/Jiangsu/1027/2013 2013/04/06 HA
ARB51629 A/guinea fowl/Alabama/17-008272-2/2017
AGR49722 A/homing pigeon/Jiangsu/SD184/2013
AGR49734 A/pigeon/Shanghai/S1069/2013
AGR49746 A/pigeon/Shanghai/S1421/2013
AGR49758 A/pigeon/Shanghai/S1423/2013
AIU46989 A/pigeon/Wuxi/0405007G/2013
AGN69430 A/pigeon/Zhejiang/P1/2013
AGN69420 A/pigeon/Zhejiang/P2/2013
AJJ94194 A/silkie chicken/Dongguan/1264/2014
AJJ94206 A/silkie chicken/Dongguan/1268/2014
AJJ95135 A/silkie chicken/Dongguan/1271/2014
AJJ94218 A/silkie chicken/Dongguan/1274/2014
AJJ94305 A/silkie chicken/Dongguan/1448/2014
AJJ94332 A/silkie chicken/Dongguan/1450/2014
AJJ94344 A/silkie chicken/Dongguan/1451/2014
AJJ94432 A/silkie chicken/Dongguan/1516/2014
AJJ95584 A/silkie chicken/Dongguan/1519/2014
AJJ93845 A/silkie chicken/Dongguan/157/2014
AJJ95358 A/silkie chicken/Dongguan/1641/2014
AJJ90673 A/silkie chicken/Dongguan/3049/2013
AJJ90733 A/silkie chicken/Dongguan/3166/2013
AJJ90783 A/silkie chicken/Dongguan/3275/2013
AJJ90795 A/silkie chicken/Dongguan/3281/2013
AJJ90807 A/silkie chicken/Dongguan/3284/2013
AJJ90891 A/silkie chicken/Dongguan/3520/2013
AJJ90903 A/silkie chicken/Dongguan/3522/2013
AJJ90915 A/silkie chicken/Dongguan/3525/2013
AJJ90927 A/silkie chicken/Dongguan/3526/2013
AJJ90939 A/silkie chicken/Dongguan/3528/2013
AJJ90987 A/silkie chicken/Dongguan/3605/2013
AJJ90999 A/silkie chicken/Dongguan/3606/2013
AJJ91131 A/silkie chicken/Dongguan/3980/2013
AJJ91143 A/silkie chicken/Dongguan/3990/2013
AJJ91227 A/silkie chicken/Dongguan/4126/2013
AJJ91239 A/silkie chicken/Dongguan/4127/2013
AJJ91264 A/silkie chicken/Dongguan/4129/2013
AJJ95440 A/silkie chicken/Dongguan/523/2014
AJJ93967 A/silkie chicken/Dongguan/635/2014
AJJ93979 A/silkie chicken/Dongguan/656/2014
AJJ95548 A/silkie chicken/Dongguan/953/2014
AJJ94081 A/silkie chicken/Dongguan/963/2014
AJJ94959 A/silkie chicken/Dongguan/967/2014
AJJ94986 A/silkie chicken/Dongguan/969/2014
AJJ94998 A/silkie chicken/Dongguan/979/2014
AJJ95010 A/silkie chicken/Dongguan/981/2014
AJJ95022 A/silkie chicken/Dongguan/986/2014
AJJ94110 A/silkie chicken/Dongguan/988/2014
AJJ94122 A/silkie chicken/Dongguan/991/2014
AJJ95560 A/silkie chicken/Dongguan/997/2014
AJJ91578 A/silkie chicken/Huzhou/4213/2013
AJJ92967 A/silkie chicken/Jiangxi/9469/2014
AJJ92979 A/silkie chicken/Jiangxi/9472/2014
AJJ92991 A/silkie chicken/Jiangxi/9476/2014
AJJ95476 A/silkie chicken/Shantou/1406/2014
AJJ97925 A/silkie chicken/Shantou/2050/2014
AJJ97937 A/silkie chicken/Shantou/2054/2014
AJJ97949 A/silkie chicken/Shantou/2056/2014
AJJ91677 A/silkie chicken/Shaoxing/5130/2013
AJJ91761 A/silkie chicken/Shaoxing/5235/2013
AJJ90613 A/silkie chicken/Shenzhen/2134/2013
AJJ90625 A/silkie chicken/Shenzhen/2139/2013
AJJ91047 A/silkie chicken/Shenzhen/3781/2013
AJJ91059 A/silkie chicken/Shenzhen/3782/2013
AJJ90538 A/silkie chicken/Shenzhen/918/2013
AJJ90550 A/silkie chicken/Shenzhen/919/2013
AHL24617 A/tree sparrow/Shanghai/01/2013
AGW82588 A/tree sparrow/Shanghai/01/2013
AGR49770 A/wild pigeon/Jiangsu/SD001/2013
EPI439507 A/Anhui/1/2013 H7N9 HA
EPI439486 A/Shanghai/1/2013 H7N9 HA
JQ906576 A/duck/Zhejiang/12/2011 H7N3 HA
321
*
335 (326)
400
APD69299 A/blue-winged teal/Louisiana/UGAI15-1367/2015
APD69287 A/blue-winged teal/Louisiana/UGAI15-1692/2015
AJS16424 A/chicken/Anhui/A1070/2014
AJS16425 A/chicken/Anhui/A1071/2014
AJS16427 A/chicken/Anhui/A1073/2014
AJS16429 A/chicken/Anhui/A1091/2014
AJS16444 A/chicken/Anhui/A1113/2014
AJS16449 A/chicken/Anhui/A1118/2014
AJS16455 A/chicken/Anhui/A1158/2014
AJS16457 A/chicken/Anhui/A1160/2014
AJS16460 A/chicken/Anhui/A1165/2014
AJS16461 A/chicken/Anhui/A1166/2014
AJS16466 A/chicken/Anhui/A1392/2014
AJS16467 A/chicken/Anhui/A1624/2014
AJJ95048 A/chicken/Dongguan/1009/2014
AJJ95060 A/chicken/Dongguan/1022/2014
AJJ94134 A/chicken/Dongguan/1051/2014
AJJ94146 A/chicken/Dongguan/1057/2014
AJJ94158 A/chicken/Dongguan/1075/2014
AJJ94570 A/chicken/Dongguan/1091/2014
AJJ96853 A/chicken/Dongguan/1096/2014
AJJ95572 A/chicken/Dongguan/1100/2014
AJJ94582 A/chicken/Dongguan/1108/2014
AJJ95072 A/chicken/Dongguan/1124/2014
AJJ94170 A/chicken/Dongguan/1143/2014
AJJ94182 A/chicken/Dongguan/1177/2014
AJJ95098 A/chicken/Dongguan/1188/2014
AJJ95110 A/chicken/Dongguan/1230/2014
AJJ94230 A/chicken/Dongguan/1297/2014
AJJ95147 A/chicken/Dongguan/1303/2014
AJJ95159 A/chicken/Dongguan/1307/2014
AJJ94242 A/chicken/Dongguan/1312/2014
AJJ95171 A/chicken/Dongguan/1314/2014
AJJ95183 A/chicken/Dongguan/1318/2014
AJJ96865 A/chicken/Dongguan/1358/2014
AJJ94254 A/chicken/Dongguan/1374/2014
AJJ95227 A/chicken/Dongguan/1382/2014
AJJ95239 A/chicken/Dongguan/1393/2014
AJJ95251 A/chicken/Dongguan/1401/2014
AJJ95281 A/chicken/Dongguan/1421/2014
AJJ95293 A/chicken/Dongguan/1433/2014
AJJ94356 A/chicken/Dongguan/1456/2014
AJJ94368 A/chicken/Dongguan/1459/2014
AJJ94396 A/chicken/Dongguan/1494/2014
AJJ94408 A/chicken/Dongguan/1505/2014
AJJ94420 A/chicken/Dongguan/1506/2014
AJJ94496 A/chicken/Dongguan/1526/2014
AJJ95322 A/chicken/Dongguan/1527/2014
AJJ95334 A/chicken/Dongguan/1533/2014
AJJ95346 A/chicken/Dongguan/1548/2014
AJJ94508 A/chicken/Dongguan/1619/2014
AJJ94533 A/chicken/Dongguan/1666/2014
AJJ94558 A/chicken/Dongguan/1673/2014
AJJ93857 A/chicken/Dongguan/169/2014
AJJ95382 A/chicken/Dongguan/1690/2014
AJJ95412 A/chicken/Dongguan/1697/2014
AJJ93869 A/chicken/Dongguan/173/2014
AJJ94594 A/chicken/Dongguan/178/2014
AJJ93881 A/chicken/Dongguan/189/2014
AJJ94606 A/chicken/Dongguan/191/2014
AJJ94618 A/chicken/Dongguan/210/2014
AJJ94630 A/chicken/Dongguan/213/2014
AJJ94642 A/chicken/Dongguan/237/2014
AJJ94654 A/chicken/Dongguan/248/2014
AJJ94666 A/chicken/Dongguan/262/2014
AJJ90661 A/chicken/Dongguan/2912/2013
AJJ90685 A/chicken/Dongguan/3112/2013
AJJ90697 A/chicken/Dongguan/3141/2013
AJJ90709 A/chicken/Dongguan/3145/2013
AJJ90721 A/chicken/Dongguan/3146/2013
AJJ90745 A/chicken/Dongguan/3219/2013
AJJ90819 A/chicken/Dongguan/3418/2013
AJJ90831 A/chicken/Dongguan/3438/2013
AJJ90843 A/chicken/Dongguan/3464/2013
AJJ90855 A/chicken/Dongguan/3487/2013
AJJ90867 A/chicken/Dongguan/3488/2013
AJJ90879 A/chicken/Dongguan/3491/2013

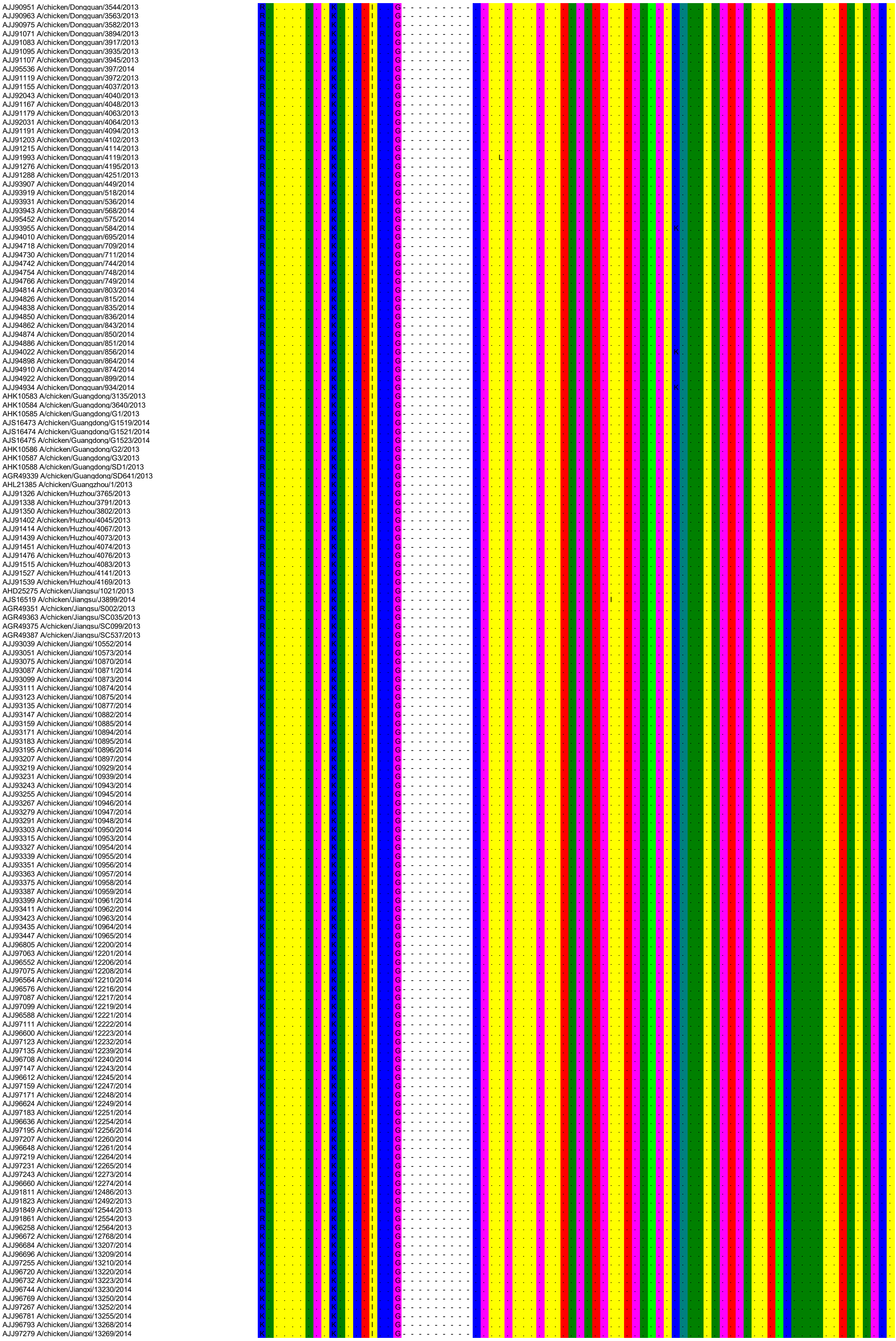
AJJ90951 A/chicken/Dongguan/3544/2013
AJJ90963 A/chicken/Dongguan/3563/2013
AJJ90975 A/chicken/Dongguan/3582/2013
AJJ91071 A/chicken/Dongguan/3894/2013
AJJ91083 A/chicken/Dongguan/3917/2013
AJJ91095 A/chicken/Dongguan/3935/2013
AJJ91107 A/chicken/Dongguan/3945/2013
AJJ95536 A/chicken/Dongguan/397/2014
AJJ91119 A/chicken/Dongguan/3972/2013
AJJ91155 A/chicken/Dongguan/4037/2013
AJJ92043 A/chicken/Dongguan/4040/2013
AJJ91167 A/chicken/Dongguan/4048/2013
AJJ91179 A/chicken/Dongguan/4063/2013
AJJ92031 A/chicken/Dongguan/4064/2013
AJJ91191 A/chicken/Dongguan/4094/2013
AJJ91203 A/chicken/Dongguan/4102/2013
AJJ91215 A/chicken/Dongguan/4114/2013
AJJ91993 A/chicken/Dongguan/4119/2013
AJJ91276 A/chicken/Dongguan/4195/2013
AJJ91288 A/chicken/Dongguan/4251/2013
AJJ93907 A/chicken/Dongguan/449/2014
AJJ93919 A/chicken/Dongguan/518/2014
AJJ93931 A/chicken/Dongguan/536/2014
AJJ93943 A/chicken/Dongguan/568/2014
AJJ95452 A/chicken/Dongguan/575/2014
AJJ93955 A/chicken/Dongguan/584/2014
AJJ94010 A/chicken/Dongguan/695/2014
AJJ94718 A/chicken/Dongguan/709/2014
AJJ94730 A/chicken/Dongguan/711/2014
AJJ94742 A/chicken/Dongguan/744/2014
AJJ94754 A/chicken/Dongguan/748/2014
AJJ94766 A/chicken/Dongguan/749/2014
AJJ94814 A/chicken/Dongguan/803/2014
AJJ94826 A/chicken/Dongguan/815/2014
AJJ94838 A/chicken/Dongguan/835/2014
AJJ94850 A/chicken/Dongguan/836/2014
AJJ94862 A/chicken/Dongguan/843/2014
AJJ94874 A/chicken/Dongguan/850/2014
AJJ94886 A/chicken/Dongguan/851/2014
AJJ94022 A/chicken/Dongguan/856/2014
AJJ94898 A/chicken/Dongguan/864/2014
AJJ94910 A/chicken/Dongguan/874/2014
AJJ94922 A/chicken/Dongguan/899/2014
AJJ94934 A/chicken/Dongguan/934/2014
AHK10583 A/chicken/Guangdong/3135/2013
AHK10584 A/chicken/Guangdong/3640/2013
AHK10585 A/chicken/Guangdong/G1/2013
AJS16473 A/chicken/Guangdong/G1519/2014
AJS16474 A/chicken/Guangdong/G1521/2014
AJS16475 A/chicken/Guangdong/G1523/2014
AHK10586 A/chicken/Guangdong/G2/2013
AHK10587 A/chicken/Guangdong/G3/2013
AHK10588 A/chicken/Guangdong/SD1/2013
AGR49339 A/chicken/Guangdong/SD641/2013
AHL21385 A/chicken/Guangzhou/1/2013
AJJ91326 A/chicken/Huzhou/3765/2013
AJJ91338 A/chicken/Huzhou/3791/2013
AJJ91350 A/chicken/Huzhou/3802/2013
AJJ91402 A/chicken/Huzhou/4045/2013
AJJ91414 A/chicken/Huzhou/4067/2013
AJJ91439 A/chicken/Huzhou/4073/2013
AJJ91451 A/chicken/Huzhou/4074/2013
AJJ91476 A/chicken/Huzhou/4076/2013
AJJ91515 A/chicken/Huzhou/4083/2013
AJJ91527 A/chicken/Huzhou/4141/2013
AJJ91539 A/chicken/Huzhou/4169/2013
AHD25275 A/chicken/Jiangsu/1021/2013
AJS16519 A/chicken/Jiangsu/J3899/2014
AGR49351 A/chicken/Jiangsu/S002/2013
AGR49363 A/chicken/Jiangsu/SC035/2013
AGR49375 A/chicken/Jiangsu/SC099/2013
AGR49387 A/chicken/Jiangsu/SC537/2013
AJJ93039 A/chicken/Jiangxi/10552/2014
AJJ93051 A/chicken/Jiangxi/10573/2014
AJJ93075 A/chicken/Jiangxi/10870/2014
AJJ93087 A/chicken/Jiangxi/10871/2014
AJJ93099 A/chicken/Jiangxi/10873/2014
AJJ93111 A/chicken/Jiangxi/10874/2014
AJJ93123 A/chicken/Jiangxi/10875/2014
AJJ93135 A/chicken/Jiangxi/10877/2014
AJJ93147 A/chicken/Jiangxi/10882/2014
AJJ93159 A/chicken/Jiangxi/10885/2014
AJJ93171 A/chicken/Jiangxi/10894/2014
AJJ93183 A/chicken/Jiangxi/10895/2014
AJJ93195 A/chicken/Jiangxi/10896/2014
AJJ93207 A/chicken/Jiangxi/10897/2014
AJJ93219 A/chicken/Jiangxi/10929/2014
AJJ93231 A/chicken/Jiangxi/10939/2014
AJJ93243 A/chicken/Jiangxi/10943/2014
AJJ93255 A/chicken/Jiangxi/10945/2014
AJJ93267 A/chicken/Jiangxi/10946/2014
AJJ93279 A/chicken/Jiangxi/10947/2014
AJJ93291 A/chicken/Jiangxi/10948/2014
AJJ93303 A/chicken/Jiangxi/10950/2014
AJJ93315 A/chicken/Jiangxi/10953/2014
AJJ93327 A/chicken/Jiangxi/10954/2014
AJJ93339 A/chicken/Jiangxi/10955/2014
AJJ93351 A/chicken/Jiangxi/10956/2014
AJJ93363 A/chicken/Jiangxi/10957/2014
AJJ93375 A/chicken/Jiangxi/10958/2014
AJJ93387 A/chicken/Jiangxi/10959/2014
AJJ93399 A/chicken/Jiangxi/10961/2014
AJJ93411 A/chicken/Jiangxi/10962/2014
AJJ93423 A/chicken/Jiangxi/10963/2014
AJJ93435 A/chicken/Jiangxi/10964/2014
AJJ93447 A/chicken/Jiangxi/10965/2014
AJJ96805 A/chicken/Jiangxi/12200/2014
AJJ97063 A/chicken/Jiangxi/12201/2014
AJJ96552 A/chicken/Jiangxi/12206/2014
AJJ97075 A/chicken/Jiangxi/12208/2014
AJJ96564 A/chicken/Jiangxi/12210/2014
AJJ96576 A/chicken/Jiangxi/12216/2014
AJJ97087 A/chicken/Jiangxi/12217/2014
AJJ97099 A/chicken/Jiangxi/12219/2014
AJJ96588 A/chicken/Jiangxi/12221/2014
AJJ97111 A/chicken/Jiangxi/12222/2014
AJJ96600 A/chicken/Jiangxi/12223/2014
AJJ97123 A/chicken/Jiangxi/12232/2014
AJJ97135 A/chicken/Jiangxi/12239/2014
AJJ96708 A/chicken/Jiangxi/12240/2014
AJJ97147 A/chicken/Jiangxi/12243/2014
AJJ96612 A/chicken/Jiangxi/12245/2014
AJJ97159 A/chicken/Jiangxi/12247/2014
AJJ97171 A/chicken/Jiangxi/12248/2014
AJJ96624 A/chicken/Jiangxi/12249/2014
AJJ97183 A/chicken/Jiangxi/12251/2014
AJJ96636 A/chicken/Jiangxi/12254/2014
AJJ97195 A/chicken/Jiangxi/12256/2014
AJJ97207 A/chicken/Jiangxi/12260/2014
AJJ96648 A/chicken/Jiangxi/12261/2014
AJJ97219 A/chicken/Jiangxi/12264/2014
AJJ97231 A/chicken/Jiangxi/12265/2014
AJJ97243 A/chicken/Jiangxi/12273/2014
AJJ96660 A/chicken/Jiangxi/12274/2014
AJJ91811 A/chicken/Jiangxi/12486/2013
AJJ91823 A/chicken/Jiangxi/12492/2013
AJJ91849 A/chicken/Jiangxi/12544/2013
AJJ91861 A/chicken/Jiangxi/12554/2013
AJJ96258 A/chicken/Jiangxi/12564/2013
AJJ96672 A/chicken/Jiangxi/12768/2014
AJJ96684 A/chicken/Jiangxi/13207/2014
AJJ96696 A/chicken/Jiangxi/13209/2014
AJJ97255 A/chicken/Jiangxi/13210/2014
AJJ96720 A/chicken/Jiangxi/13220/2014
AJJ96732 A/chicken/Jiangxi/13223/2014
AJJ96744 A/chicken/Jiangxi/13230/2014
AJJ96769 A/chicken/Jiangxi/13250/2014
AJJ97267 A/chicken/Jiangxi/13252/2014
AJJ96781 A/chicken/Jiangxi/13255/2014
AJJ96793 A/chicken/Jiangxi/13268/2014
AJJ97279 A/chicken/Jiangxi/13269/2014

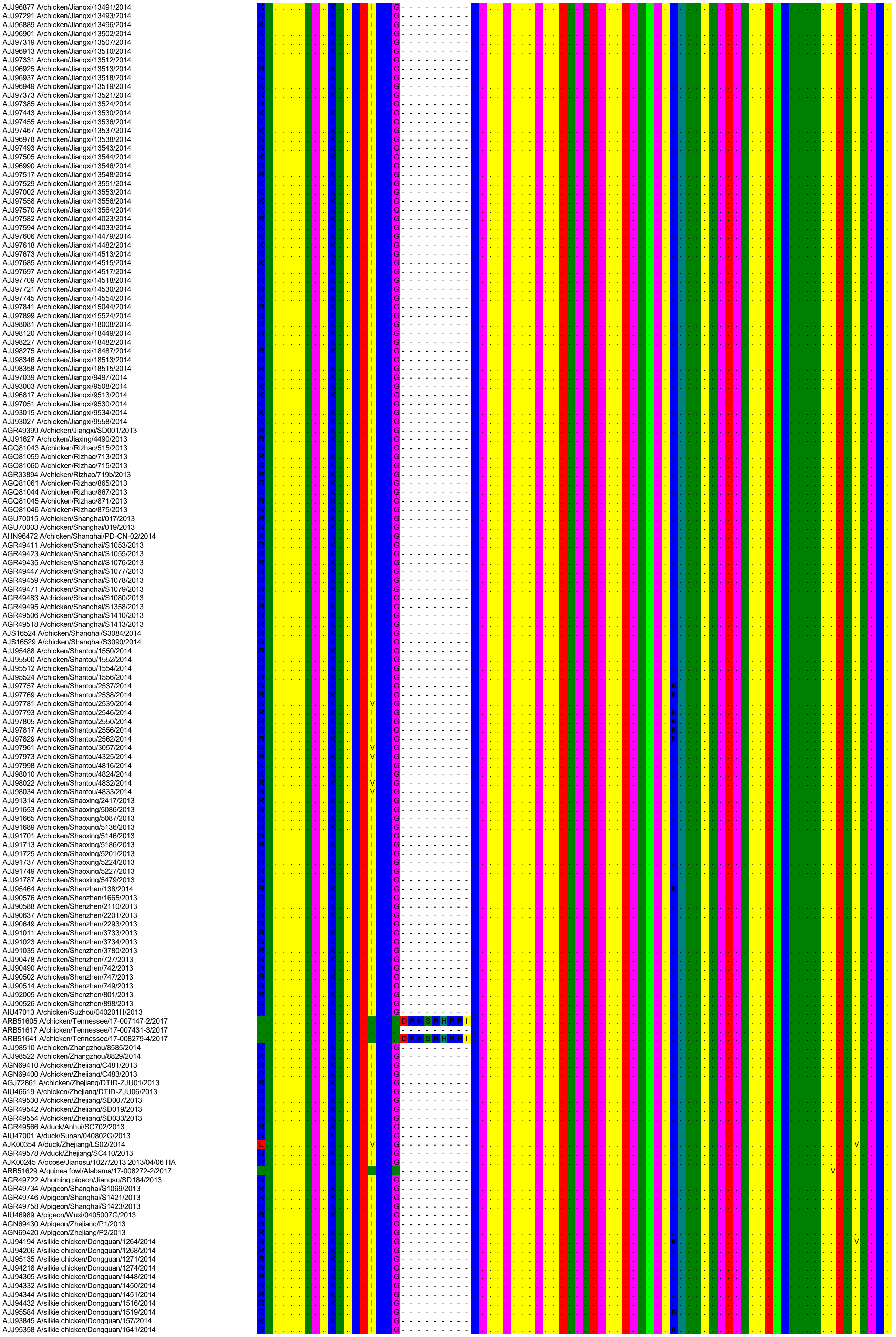

AJJ96877 A/chicken/Jiangxi/13491/2014
AJJ97291 A/chicken/Jiangxi/13493/2014
AJJ96889 A/chicken/Jiangxi/13496/2014
AJJ96901 A/chicken/Jiangxi/13502/2014
AJJ97319 A/chicken/Jiangxi/13507/2014
AJJ96913 A/chicken/Jiangxi/13510/2014
AJJ97331 A/chicken/Jiangxi/13512/2014
AJJ96925 A/chicken/Jiangxi/13513/2014
AJJ96937 A/chicken/Jiangxi/13518/2014
AJJ96949 A/chicken/Jiangxi/13519/2014
AJJ97373 A/chicken/Jiangxi/13521/2014
AJJ97385 A/chicken/Jiangxi/13524/2014
AJJ97443 A/chicken/Jiangxi/13530/2014
AJJ97455 A/chicken/Jiangxi/13536/2014
AJJ97467 A/chicken/Jiangxi/13537/2014
AJJ96978 A/chicken/Jiangxi/13538/2014
AJJ97493 A/chicken/Jiangxi/13543/2014
AJJ97505 A/chicken/Jiangxi/13544/2014
AJJ96990 A/chicken/Jiangxi/13546/2014
AJJ97517 A/chicken/Jiangxi/13548/2014
AJJ97529 A/chicken/Jiangxi/13551/2014
AJJ97002 A/chicken/Jiangxi/13553/2014
AJJ97558 A/chicken/Jiangxi/13556/2014
AJJ97570 A/chicken/Jiangxi/13564/2014
AJJ97582 A/chicken/Jiangxi/14023/2014
AJJ97594 A/chicken/Jiangxi/14033/2014
AJJ97606 A/chicken/Jiangxi/14479/2014
AJJ97618 A/chicken/Jiangxi/14482/2014
AJJ97673 A/chicken/Jiangxi/14513/2014
AJJ97685 A/chicken/Jiangxi/14515/2014
AJJ97697 A/chicken/Jiangxi/14517/2014
AJJ97709 A/chicken/Jiangxi/14518/2014
AJJ97721 A/chicken/Jiangxi/14530/2014
AJJ97745 A/chicken/Jiangxi/14554/2014
AJJ97841 A/chicken/Jiangxi/15044/2014
AJJ97899 A/chicken/Jiangxi/15524/2014
AJJ98081 A/chicken/Jiangxi/18008/2014
AJJ98120 A/chicken/Jiangxi/18449/2014
AJJ98227 A/chicken/Jiangxi/18482/2014
AJJ98275 A/chicken/Jiangxi/18487/2014
AJJ98346 A/chicken/Jiangxi/18513/2014
AJJ98358 A/chicken/Jiangxi/18515/2014
AJJ97039 A/chicken/Jiangxi/9497/2014
AJJ93003 A/chicken/Jiangxi/9508/2014
AJJ96817 A/chicken/Jiangxi/9513/2014
AJJ97051 A/chicken/Jiangxi/9530/2014
AJJ93015 A/chicken/Jiangxi/9534/2014
AJJ93027 A/chicken/Jiangxi/9558/2014
AGR49399 A/chicken/Jiangxi/SD001/2013
AJJ91627 A/chicken/Jiaxing/4490/2013
AGQ81043 A/chicken/Rizhao/515/2013
AGQ81059 A/chicken/Rizhao/713/2013
AGQ81060 A/chicken/Rizhao/715/2013
AGR33894 A/chicken/Rizhao/719b/2013
AGQ81061 A/chicken/Rizhao/865/2013
AGQ81044 A/chicken/Rizhao/867/2013
AGQ81045 A/chicken/Rizhao/871/2013
AGQ81046 A/chicken/Rizhao/875/2013
AGU70015 A/chicken/Shanghai/017/2013
AGU70003 A/chicken/Shanghai/019/2013
AHN96472 A/chicken/Shanghai/PD-CN-02/2014
AGR49411 A/chicken/Shanghai/S1053/2013
AGR49423 A/chicken/Shanghai/S1055/2013
AGR49435 A/chicken/Shanghai/S1076/2013
AGR49447 A/chicken/Shanghai/S1077/2013
AGR49459 A/chicken/Shanghai/S1078/2013
AGR49471 A/chicken/Shanghai/S1079/2013
AGR49483 A/chicken/Shanghai/S1080/2013
AGR49495 A/chicken/Shanghai/S1358/2013
AGR49506 A/chicken/Shanghai/S1410/2013
AGR49518 A/chicken/Shanghai/S1413/2013
AJS16524 A/chicken/Shanghai/S3084/2014
AJS16529 A/chicken/Shanghai/S3090/2014
AJJ95488 A/chicken/Shantou/1550/2014
AJJ95500 A/chicken/Shantou/1552/2014
AJJ95512 A/chicken/Shantou/1554/2014
AJJ95524 A/chicken/Shantou/1556/2014
AJJ97757 A/chicken/Shantou/2537/2014
AJJ97769 A/chicken/Shantou/2538/2014
AJJ97781 A/chicken/Shantou/2539/2014
AJJ97793 A/chicken/Shantou/2546/2014
AJJ97805 A/chicken/Shantou/2550/2014
AJJ97817 A/chicken/Shantou/2556/2014
AJJ97829 A/chicken/Shantou/2562/2014
AJJ97961 A/chicken/Shantou/3057/2014
AJJ97973 A/chicken/Shantou/4325/2014
AJJ97998 A/chicken/Shantou/4816/2014
AJJ98010 A/chicken/Shantou/4824/2014
AJJ98022 A/chicken/Shantou/4832/2014
AJJ98034 A/chicken/Shantou/4833/2014
AJJ91314 A/chicken/Shaoxing/2417/2013
AJJ91653 A/chicken/Shaoxing/5086/2013
AJJ91665 A/chicken/Shaoxing/5087/2013
AJJ91689 A/chicken/Shaoxing/5136/2013
AJJ91701 A/chicken/Shaoxing/5146/2013
AJJ91713 A/chicken/Shaoxing/5186/2013
AJJ91725 A/chicken/Shaoxing/5201/2013
AJJ91737 A/chicken/Shaoxing/5224/2013
AJJ91749 A/chicken/Shaoxing/5227/2013
AJJ91787 A/chicken/Shaoxing/5479/2013
AJJ95464 A/chicken/Shenzhen/138/2014
AJJ90576 A/chicken/Shenzhen/1665/2013
AJJ90588 A/chicken/Shenzhen/2110/2013
AJJ90637 A/chicken/Shenzhen/2201/2013
AJJ90649 A/chicken/Shenzhen/2293/2013
AJJ91011 A/chicken/Shenzhen/3733/2013
AJJ91023 A/chicken/Shenzhen/3734/2013
AJJ91035 A/chicken/Shenzhen/3780/2013
AJJ90478 A/chicken/Shenzhen/727/2013
AJJ90490 A/chicken/Shenzhen/742/2013
AJJ90502 A/chicken/Shenzhen/747/2013
AJJ90514 A/chicken/Shenzhen/749/2013
AJJ92005 A/chicken/Shenzhen/801/2013
AJJ90526 A/chicken/Shenzhen/898/2013
AIU47013 A/chicken/Suzhou/040201H/2013
ARB51605 A/chicken/Tennessee/17-007147-2/2017
ARB51617 A/chicken/Tennessee/17-007431-3/2017
ARB51641 A/chicken/Tennessee/17-008279-4/2017
AJJ98510 A/chicken/Zhangzhou/8585/2014
AJJ98522 A/chicken/Zhangzhou/8829/2014
AGN69410 A/chicken/Zhejiang/C481/2013
AGN69400 A/chicken/Zhejiang/C483/2013
AGJ72861 A/chicken/Zhejiang/DTID-ZJU01/2013
AIU46619 A/chicken/Zhejiang/DTID-ZJU06/2013
AGR49530 A/chicken/Zhejiang/SD007/2013
AGR49542 A/chicken/Zhejiang/SD019/2013
AGR49554 A/chicken/Zhejiang/SD033/2013
AGR49566 A/duck/Anhui/SC702/2013
AIU47001 A/duck/Sunan/040802G/2013
AJK00354 A/duck/Zhejiang/LS02/2014
AGR49578 A/duck/Zhejiang/SC410/2013
AJK00245 A/goose/Jiangsu/1027/2013 2013/04/06 HA
ARB51629 A/guinea fowl/Alabama/17-008272-2/2017
AGR49722 A/homing pigeon/Jiangsu/SD184/2013
AGR49734 A/pigeon/Shanghai/S1069/2013
AGR49746 A/pigeon/Shanghai/S1421/2013
AGR49758 A/pigeon/Shanghai/S1423/2013
AIU46989 A/pigeon/Wuxi/0405007G/2013
AGN69430 A/pigeon/Zhejiang/P1/2013
AGN69420 A/pigeon/Zhejiang/P2/2013
AJJ94194 A/silkie chicken/Dongguan/1264/2014
AJJ94206 A/silkie chicken/Dongguan/1268/2014
AJJ95135 A/silkie chicken/Dongguan/1271/2014
AJJ94218 A/silkie chicken/Dongguan/1274/2014
AJJ94305 A/silkie chicken/Dongguan/1448/2014
AJJ94332 A/silkie chicken/Dongguan/1450/2014
AJJ94344 A/silkie chicken/Dongguan/1451/2014
AJJ94432 A/silkie chicken/Dongguan/1516/2014
AJJ95584 A/silkie chicken/Dongguan/1519/2014
AJJ93845 A/silkie chicken/Dongguan/157/2014
AJJ95358 A/silkie chicken/Dongguan/1641/2014

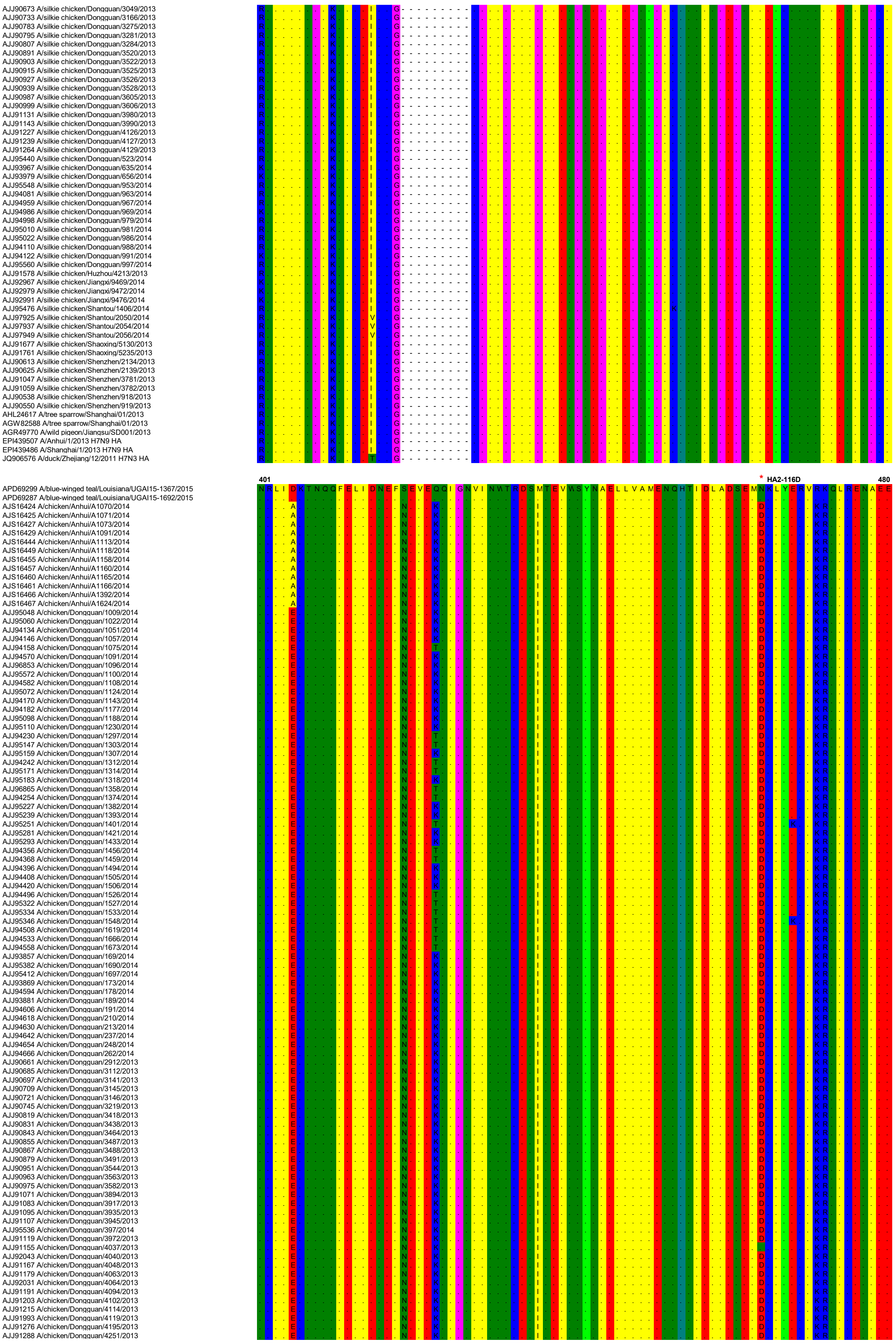
AJJ90673 A/silkie chicken/Dongguan/3049/2013
AJJ90733 A/silkie chicken/Dongguan/3166/2013
AJJ90783 A/silkie chicken/Dongguan/3275/2013
AJJ90795 A/silkie chicken/Dongguan/3281/2013
AJJ90807 A/silkie chicken/Dongguan/3284/2013
AJJ90891 A/silkie chicken/Dongguan/3520/2013
AJJ90903 A/silkie chicken/Dongguan/3522/2013
AJJ90915 A/silkie chicken/Dongguan/3525/2013
AJJ90927 A/silkie chicken/Dongguan/3526/2013
AJJ90939 A/silkie chicken/Dongguan/3528/2013
AJJ90987 A/silkie chicken/Dongguan/3605/2013
AJJ90999 A/silkie chicken/Dongguan/3606/2013
AJJ91131 A/silkie chicken/Dongguan/3980/2013
AJJ91143 A/silkie chicken/Dongguan/3990/2013
AJJ91227 A/silkie chicken/Dongguan/4126/2013
AJJ91239 A/silkie chicken/Dongguan/4127/2013
AJJ91264 A/silkie chicken/Dongguan/4129/2013
AJJ95440 A/silkie chicken/Dongguan/523/2014
AJJ93967 A/silkie chicken/Dongguan/635/2014
AJJ93979 A/silkie chicken/Dongguan/656/2014
AJJ95548 A/silkie chicken/Dongguan/953/2014
AJJ94081 A/silkie chicken/Dongguan/963/2014
AJJ94959 A/silkie chicken/Dongguan/967/2014
AJJ94986 A/silkie chicken/Dongguan/969/2014
AJJ94998 A/silkie chicken/Dongguan/979/2014
AJJ95010 A/silkie chicken/Dongguan/981/2014
AJJ95022 A/silkie chicken/Dongguan/986/2014
AJJ94110 A/silkie chicken/Dongguan/988/2014
AJJ94122 A/silkie chicken/Dongguan/991/2014
AJJ95560 A/silkie chicken/Dongguan/997/2014
AJJ91578 A/silkie chicken/Huzhou/4213/2013
AJJ92967 A/silkie chicken/Jiangxi/9469/2014
AJJ92979 A/silkie chicken/Jiangxi/9472/2014
AJJ92991 A/silkie chicken/Jiangxi/9476/2014
AJJ95476 A/silkie chicken/Shantou/1406/2014
AJJ97925 A/silkie chicken/Shantou/2050/2014
AJJ97937 A/silkie chicken/Shantou/2054/2014
AJJ97949 A/silkie chicken/Shantou/2056/2014
AJJ91677 A/silkie chicken/Shaoxing/5130/2013
AJJ91761 A/silkie chicken/Shaoxing/5235/2013
AJJ90613 A/silkie chicken/Shenzhen/2134/2013
AJJ90625 A/silkie chicken/Shenzhen/2139/2013
AJJ91047 A/silkie chicken/Shenzhen/3781/2013
AJJ91059 A/silkie chicken/Shenzhen/3782/2013
AJJ90538 A/silkie chicken/Shenzhen/918/2013
AJJ90550 A/silkie chicken/Shenzhen/919/2013
AHL24617 A/tree sparrow/Shanghai/01/2013
AGW82588 A/tree sparrow/Shanghai/01/2013
AGR49770 A/wild pigeon/Jiangsu/SD001/2013
EPI439507 A/Anhui/1/2013 H7N9 HA
EPI439486 A/Shanghai/1/2013 H7N9 HA
JQ906576 A/duck/Zhejiang/12/2011 H7N3 HA
401
* HA2-116D
480
APD69299 A/blue-winged teal/Louisiana/UGAI15-1367/2015
APD69287 A/blue-winged teal/Louisiana/UGAI15-1692/2015
AJS16424 A/chicken/Anhui/A1070/2014
AJS16425 A/chicken/Anhui/A1071/2014
AJS16427 A/chicken/Anhui/A1073/2014
AJS16429 A/chicken/Anhui/A1091/2014
AJS16444 A/chicken/Anhui/A1113/2014
AJS16449 A/chicken/Anhui/A1118/2014
AJS16455 A/chicken/Anhui/A1158/2014
AJS16457 A/chicken/Anhui/A1160/2014
AJS16460 A/chicken/Anhui/A1165/2014
AJS16461 A/chicken/Anhui/A1166/2014
AJS16466 A/chicken/Anhui/A1392/2014
AJS16467 A/chicken/Anhui/A1624/2014
AJJ95048 A/chicken/Dongguan/1009/2014
AJJ95060 A/chicken/Dongguan/1022/2014
AJJ94134 A/chicken/Dongguan/1051/2014
AJJ94146 A/chicken/Dongguan/1057/2014
AJJ94158 A/chicken/Dongguan/1075/2014
AJJ94570 A/chicken/Dongguan/1091/2014
AJJ96853 A/chicken/Dongguan/1096/2014
AJJ95572 A/chicken/Dongguan/1100/2014
AJJ94582 A/chicken/Dongguan/1108/2014
AJJ95072 A/chicken/Dongguan/1124/2014
AJJ94170 A/chicken/Dongguan/1143/2014
AJJ94182 A/chicken/Dongguan/1177/2014
AJJ95098 A/chicken/Dongguan/1188/2014
AJJ95110 A/chicken/Dongguan/1230/2014
AJJ94230 A/chicken/Dongguan/1297/2014
AJJ95147 A/chicken/Dongguan/1303/2014
AJJ95159 A/chicken/Dongguan/1307/2014
AJJ94242 A/chicken/Dongguan/1312/2014
AJJ95171 A/chicken/Dongguan/1314/2014
AJJ95183 A/chicken/Dongguan/1318/2014
AJJ96865 A/chicken/Dongguan/1358/2014
AJJ94254 A/chicken/Dongguan/1374/2014
AJJ95227 A/chicken/Dongguan/1382/2014
AJJ95239 A/chicken/Dongguan/1393/2014
AJJ95251 A/chicken/Dongguan/1401/2014
AJJ95281 A/chicken/Dongguan/1421/2014
AJJ95293 A/chicken/Dongguan/1433/2014
AJJ94356 A/chicken/Dongguan/1456/2014
AJJ94368 A/chicken/Dongguan/1459/2014
AJJ94396 A/chicken/Dongguan/1494/2014
AJJ94408 A/chicken/Dongguan/1505/2014
AJJ94420 A/chicken/Dongguan/1506/2014
AJJ94496 A/chicken/Dongguan/1526/2014
AJJ95322 A/chicken/Dongguan/1527/2014
AJJ95334 A/chicken/Dongguan/1533/2014
AJJ95346 A/chicken/Dongguan/1548/2014
AJJ94508 A/chicken/Dongguan/1619/2014
AJJ94533 A/chicken/Dongguan/1666/2014
AJJ94558 A/chicken/Dongguan/1673/2014
AJJ93857 A/chicken/Dongguan/169/2014
AJJ95382 A/chicken/Dongguan/1690/2014
AJJ95412 A/chicken/Dongguan/1697/2014
AJJ93869 A/chicken/Dongguan/173/2014
AJJ94594 A/chicken/Dongguan/178/2014
AJJ93881 A/chicken/Dongguan/189/2014
AJJ94606 A/chicken/Dongguan/191/2014
AJJ94618 A/chicken/Dongguan/210/2014
AJJ94630 A/chicken/Dongguan/213/2014
AJJ94642 A/chicken/Dongguan/237/2014
AJJ94654 A/chicken/Dongguan/248/2014
AJJ94666 A/chicken/Dongguan/262/2014
AJJ90661 A/chicken/Dongguan/2912/2013
AJJ90685 A/chicken/Dongguan/3112/2013
AJJ90697 A/chicken/Dongguan/3141/2013
AJJ90709 A/chicken/Dongguan/3145/2013
AJJ90721 A/chicken/Dongguan/3146/2013
AJJ90745 A/chicken/Dongguan/3219/2013
AJJ90819 A/chicken/Dongguan/3418/2013
AJJ90831 A/chicken/Dongguan/3438/2013
AJJ90843 A/chicken/Dongguan/3464/2013
AJJ90855 A/chicken/Dongguan/3487/2013
AJJ90867 A/chicken/Dongguan/3488/2013
AJJ90879 A/chicken/Dongguan/3491/2013
AJJ90951 A/chicken/Dongguan/3544/2013
AJJ90963 A/chicken/Dongguan/3563/2013
AJJ90975 A/chicken/Dongguan/3582/2013
AJJ91071 A/chicken/Dongguan/3894/2013
AJJ91083 A/chicken/Dongguan/3917/2013
AJJ91095 A/chicken/Dongguan/3935/2013
AJJ91107 A/chicken/Dongguan/3945/2013
AJJ95536 A/chicken/Dongguan/397/2014
AJJ91119 A/chicken/Dongguan/3972/2013
AJJ91155 A/chicken/Dongguan/4037/2013
AJJ92043 A/chicken/Dongguan/4040/2013
AJJ91167 A/chicken/Dongguan/4048/2013
AJJ91179 A/chicken/Dongguan/4063/2013
AJJ92031 A/chicken/Dongguan/4064/2013
AJJ91191 A/chicken/Dongguan/4094/2013
AJJ91203 A/chicken/Dongguan/4102/2013
AJJ91215 A/chicken/Dongguan/4114/2013
AJJ91993 A/chicken/Dongguan/4119/2013
AJJ91276 A/chicken/Dongguan/4195/2013
AJJ91288 A/chicken/Dongguan/4251/2013

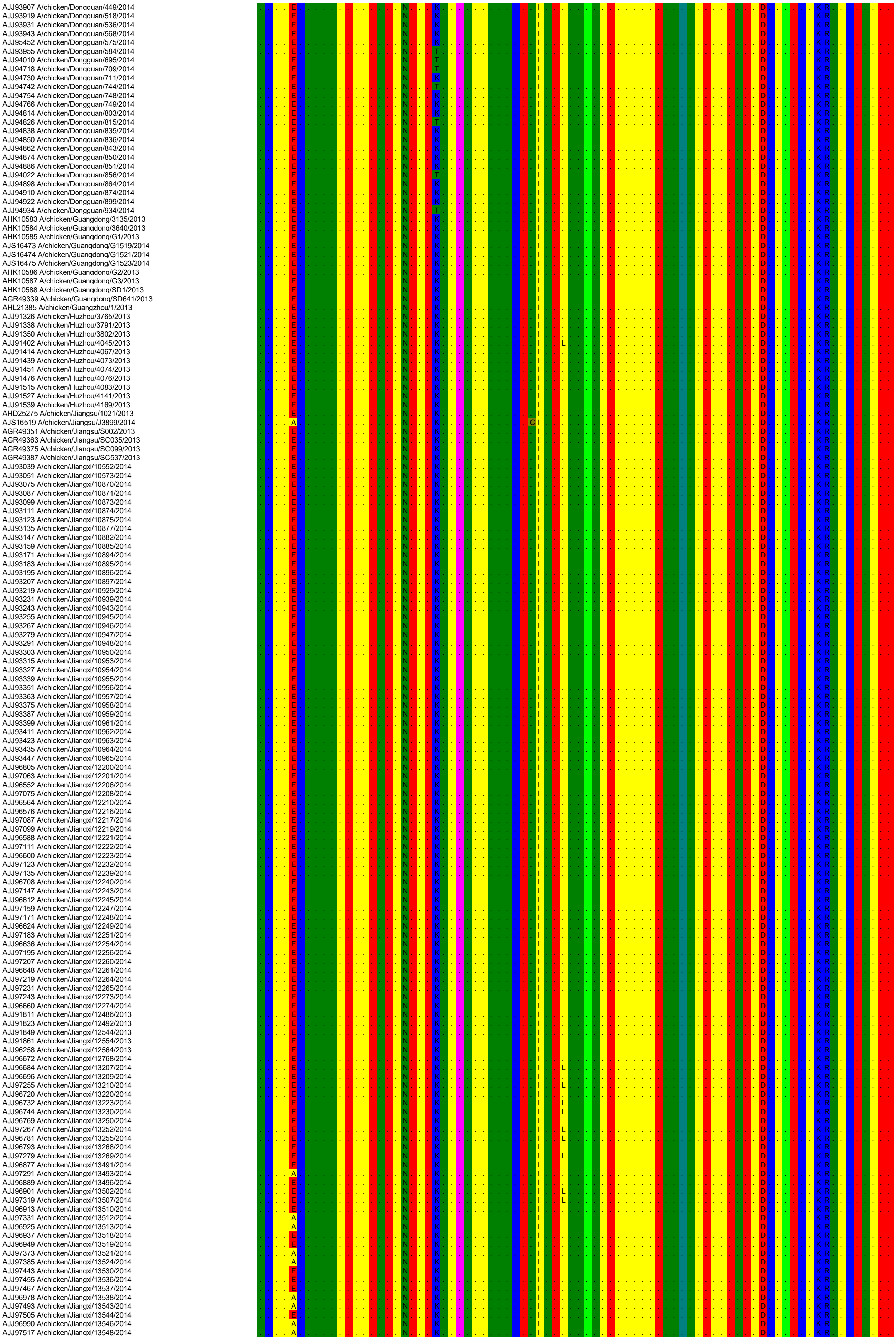

AJJ93907 A/chicken/Dongguan/449/2014
AJJ93919 A/chicken/Dongguan/518/2014
AJJ93931 A/chicken/Dongguan/536/2014
AJJ93943 A/chicken/Dongguan/568/2014
AJJ95452 A/chicken/Dongguan/575/2014
AJJ93955 A/chicken/Dongguan/584/2014
AJJ94010 A/chicken/Dongguan/695/2014
AJJ94718 A/chicken/Dongguan/709/2014
AJJ94730 A/chicken/Dongguan/711/2014
AJJ94742 A/chicken/Dongguan/744/2014
AJJ94754 A/chicken/Dongguan/748/2014
AJJ94766 A/chicken/Dongguan/749/2014
AJJ94814 A/chicken/Dongguan/803/2014
AJJ94826 A/chicken/Dongguan/815/2014
AJJ94838 A/chicken/Dongguan/835/2014
AJJ94850 A/chicken/Dongguan/836/2014
AJJ94862 A/chicken/Dongguan/843/2014
AJJ94874 A/chicken/Dongguan/850/2014
AJJ94886 A/chicken/Dongguan/851/2014
AJJ94022 A/chicken/Dongguan/856/2014
AJJ94898 A/chicken/Dongguan/864/2014
AJJ94910 A/chicken/Dongguan/874/2014
AJJ94922 A/chicken/Dongguan/899/2014
AJJ94934 A/chicken/Dongguan/934/2014
AHK10583 A/chicken/Guangdong/3135/2013
AHK10584 A/chicken/Guangdong/3640/2013
AHK10585 A/chicken/Guangdong/G1/2013
AJS16473 A/chicken/Guangdong/G1519/2014
AJS16474 A/chicken/Guangdong/G1521/2014
AJS16475 A/chicken/Guangdong/G1523/2014
AHK10586 A/chicken/Guangdong/G2/2013
AHK10587 A/chicken/Guangdong/G3/2013
AHK10588 A/chicken/Guangdong/SD1/2013
AGR49339 A/chicken/Guangdong/SD641/2013
AHL21385 A/chicken/Guangzhou/1/2013
AJJ91326 A/chicken/Huzhou/3765/2013
AJJ91338 A/chicken/Huzhou/3791/2013
AJJ91350 A/chicken/Huzhou/3802/2013
AJJ91402 A/chicken/Huzhou/4045/2013
AJJ91414 A/chicken/Huzhou/4067/2013
AJJ91439 A/chicken/Huzhou/4073/2013
AJJ91451 A/chicken/Huzhou/4074/2013
AJJ91476 A/chicken/Huzhou/4076/2013
AJJ91515 A/chicken/Huzhou/4083/2013
AJJ91527 A/chicken/Huzhou/4141/2013
AJJ91539 A/chicken/Huzhou/4169/2013
AHD25275 A/chicken/Jiangsu/1021/2013
AJS16519 A/chicken/Jiangsu/J3899/2014
AGR49351 A/chicken/Jiangsu/S002/2013
AGR49363 A/chicken/Jiangsu/SC035/2013
AGR49375 A/chicken/Jiangsu/SC099/2013
AGR49387 A/chicken/Jiangsu/SC537/2013
AJJ93039 A/chicken/Jiangxi/10552/2014
AJJ93051 A/chicken/Jiangxi/10573/2014
AJJ93075 A/chicken/Jiangxi/10870/2014
AJJ93087 A/chicken/Jiangxi/10871/2014
AJJ93099 A/chicken/Jiangxi/10873/2014
AJJ93111 A/chicken/Jiangxi/10874/2014
AJJ93123 A/chicken/Jiangxi/10875/2014
AJJ93135 A/chicken/Jiangxi/10877/2014
AJJ93147 A/chicken/Jiangxi/10882/2014
AJJ93159 A/chicken/Jiangxi/10885/2014
AJJ93171 A/chicken/Jiangxi/10894/2014
AJJ93183 A/chicken/Jiangxi/10895/2014
AJJ93195 A/chicken/Jiangxi/10896/2014
AJJ93207 A/chicken/Jiangxi/10897/2014
AJJ93219 A/chicken/Jiangxi/10929/2014
AJJ93231 A/chicken/Jiangxi/10939/2014
AJJ93243 A/chicken/Jiangxi/10943/2014
AJJ93255 A/chicken/Jiangxi/10945/2014
AJJ93267 A/chicken/Jiangxi/10946/2014
AJJ93279 A/chicken/Jiangxi/10947/2014
AJJ93291 A/chicken/Jiangxi/10948/2014
AJJ93303 A/chicken/Jiangxi/10950/2014
AJJ93315 A/chicken/Jiangxi/10953/2014
AJJ93327 A/chicken/Jiangxi/10954/2014
AJJ93339 A/chicken/Jiangxi/10955/2014
AJJ93351 A/chicken/Jiangxi/10956/2014
AJJ93363 A/chicken/Jiangxi/10957/2014
AJJ93375 A/chicken/Jiangxi/10958/2014
AJJ93387 A/chicken/Jiangxi/10959/2014
AJJ93399 A/chicken/Jiangxi/10961/2014
AJJ93411 A/chicken/Jiangxi/10962/2014
AJJ93423 A/chicken/Jiangxi/10963/2014
AJJ93435 A/chicken/Jiangxi/10964/2014
AJJ93447 A/chicken/Jiangxi/10965/2014
AJJ96805 A/chicken/Jiangxi/12200/2014
AJJ97063 A/chicken/Jiangxi/12201/2014
AJJ96552 A/chicken/Jiangxi/12206/2014
AJJ97075 A/chicken/Jiangxi/12208/2014
AJJ96564 A/chicken/Jiangxi/12210/2014
AJJ96576 A/chicken/Jiangxi/12216/2014
AJJ97087 A/chicken/Jiangxi/12217/2014
AJJ97099 A/chicken/Jiangxi/12219/2014
AJJ96588 A/chicken/Jiangxi/12221/2014
AJJ97111 A/chicken/Jiangxi/12222/2014
AJJ96600 A/chicken/Jiangxi/12223/2014
AJJ97123 A/chicken/Jiangxi/12232/2014
AJJ97135 A/chicken/Jiangxi/12239/2014
AJJ96708 A/chicken/Jiangxi/12240/2014
AJJ97147 A/chicken/Jiangxi/12243/2014
AJJ96612 A/chicken/Jiangxi/12245/2014
AJJ97159 A/chicken/Jiangxi/12247/2014
AJJ97171 A/chicken/Jiangxi/12248/2014
AJJ96624 A/chicken/Jiangxi/12249/2014
AJJ97183 A/chicken/Jiangxi/12251/2014
AJJ96636 A/chicken/Jiangxi/12254/2014
AJJ97195 A/chicken/Jiangxi/12256/2014
AJJ97207 A/chicken/Jiangxi/12260/2014
AJJ96648 A/chicken/Jiangxi/12261/2014
AJJ97219 A/chicken/Jiangxi/12264/2014
AJJ97231 A/chicken/Jiangxi/12265/2014
AJJ97243 A/chicken/Jiangxi/12273/2014
AJJ96660 A/chicken/Jiangxi/12274/2014
AJJ91811 A/chicken/Jiangxi/12486/2013
AJJ91823 A/chicken/Jiangxi/12492/2013
AJJ91849 A/chicken/Jiangxi/12544/2013
AJJ91861 A/chicken/Jiangxi/12554/2013
AJJ96258 A/chicken/Jiangxi/12564/2013
AJJ96672 A/chicken/Jiangxi/12768/2014
AJJ96684 A/chicken/Jiangxi/13207/2014
AJJ96696 A/chicken/Jiangxi/13209/2014
AJJ97255 A/chicken/Jiangxi/13210/2014
AJJ96720 A/chicken/Jiangxi/13220/2014
AJJ96732 A/chicken/Jiangxi/13223/2014
AJJ96744 A/chicken/Jiangxi/13230/2014
AJJ96769 A/chicken/Jiangxi/13250/2014
AJJ97267 A/chicken/Jiangxi/13252/2014
AJJ96781 A/chicken/Jiangxi/13255/2014
AJJ96793 A/chicken/Jiangxi/13268/2014
AJJ97279 A/chicken/Jiangxi/13269/2014
AJJ96877 A/chicken/Jiangxi/13491/2014
AJJ97291 A/chicken/Jiangxi/13493/2014
AJJ96889 A/chicken/Jiangxi/13496/2014
AJJ96901 A/chicken/Jiangxi/13502/2014
AJJ97319 A/chicken/Jiangxi/13507/2014
AJJ96913 A/chicken/Jiangxi/13510/2014
AJJ97331 A/chicken/Jiangxi/13512/2014
AJJ96925 A/chicken/Jiangxi/13513/2014
AJJ96937 A/chicken/Jiangxi/13518/2014
AJJ96949 A/chicken/Jiangxi/13519/2014
AJJ97373 A/chicken/Jiangxi/13521/2014
AJJ97385 A/chicken/Jiangxi/13524/2014
AJJ97443 A/chicken/Jiangxi/13530/2014
AJJ97455 A/chicken/Jiangxi/13536/2014
AJJ97467 A/chicken/Jiangxi/13537/2014
AJJ96978 A/chicken/Jiangxi/13538/2014
AJJ97493 A/chicken/Jiangxi/13543/2014
AJJ97505 A/chicken/Jiangxi/13544/2014
AJJ96990 A/chicken/Jiangxi/13546/2014
AJJ97517 A/chicken/Jiangxi/13548/2014

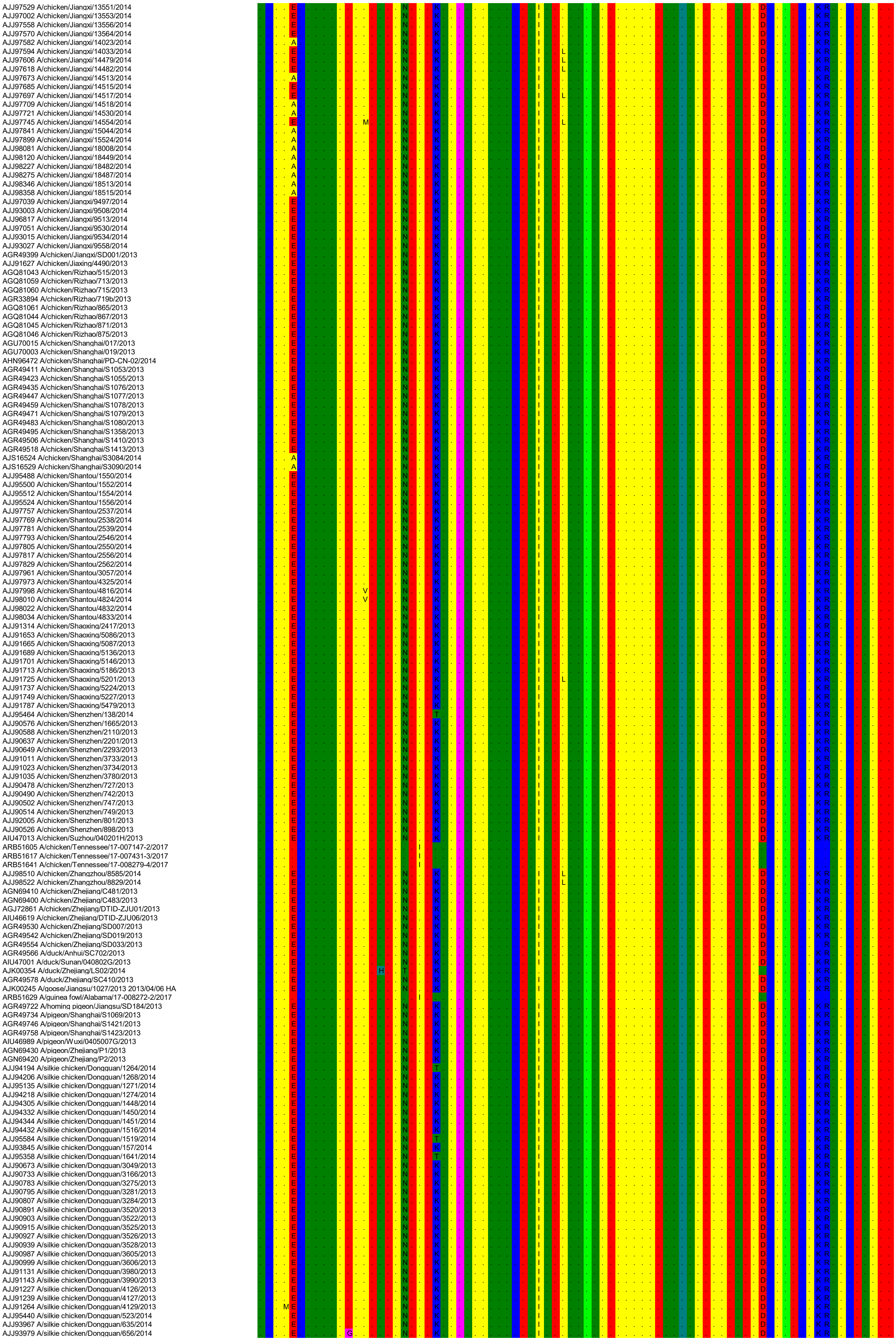
AJJ97529 A/chicken/Jiangxi/13551/2014
AJJ97002 A/chicken/Jiangxi/13553/2014
AJJ97558 A/chicken/Jiangxi/13556/2014
AJJ97570 A/chicken/Jiangxi/13564/2014
AJJ97582 A/chicken/Jiangxi/14023/2014
AJJ97594 A/chicken/Jiangxi/14033/2014
AJJ97606 A/chicken/Jiangxi/14479/2014
AJJ97618 A/chicken/Jiangxi/14482/2014
AJJ97673 A/chicken/Jiangxi/14513/2014
AJJ97685 A/chicken/Jiangxi/14515/2014
AJJ97697 A/chicken/Jiangxi/14517/2014
AJJ97709 A/chicken/Jiangxi/14518/2014
AJJ97721 A/chicken/Jiangxi/14530/2014
AJJ97745 A/chicken/Jiangxi/14554/2014
AJJ97841 A/chicken/Jiangxi/15044/2014
AJJ97899 A/chicken/Jiangxi/15524/2014
AJJ98081 A/chicken/Jiangxi/18008/2014
AJJ98120 A/chicken/Jiangxi/18449/2014
AJJ98227 A/chicken/Jiangxi/18482/2014
AJJ98275 A/chicken/Jiangxi/18487/2014
AJJ98346 A/chicken/Jiangxi/18513/2014
AJJ98358 A/chicken/Jiangxi/18515/2014
AJJ97039 A/chicken/Jiangxi/9497/2014
AJJ93003 A/chicken/Jiangxi/9508/2014
AJJ96817 A/chicken/Jiangxi/9513/2014
AJJ97051 A/chicken/Jiangxi/9530/2014
AJJ93015 A/chicken/Jiangxi/9534/2014
AJJ93027 A/chicken/Jiangxi/9558/2014
AGR49399 A/chicken/Jiangxi/SD001/2013
AJJ91627 A/chicken/Jiaxing/4490/2013
AGQ81043 A/chicken/Rizhao/515/2013
AGQ81059 A/chicken/Rizhao/713/2013
AGQ81060 A/chicken/Rizhao/715/2013
AGR33894 A/chicken/Rizhao/719b/2013
AGQ81061 A/chicken/Rizhao/865/2013
AGQ81044 A/chicken/Rizhao/867/2013
AGQ81045 A/chicken/Rizhao/871/2013
AGQ81046 A/chicken/Rizhao/875/2013
AGU70015 A/chicken/Shanghai/017/2013
AGU70003 A/chicken/Shanghai/019/2013
AHN96472 A/chicken/Shanghai/PD-CN-02/2014
AGR49411 A/chicken/Shanghai/S1053/2013
AGR49423 A/chicken/Shanghai/S1055/2013
AGR49435 A/chicken/Shanghai/S1076/2013
AGR49447 A/chicken/Shanghai/S1077/2013
AGR49459 A/chicken/Shanghai/S1078/2013
AGR49471 A/chicken/Shanghai/S1079/2013
AGR49483 A/chicken/Shanghai/S1080/2013
AGR49495 A/chicken/Shanghai/S1358/2013
AGR49506 A/chicken/Shanghai/S1410/2013
AGR49518 A/chicken/Shanghai/S1413/2013
AJS16524 A/chicken/Shanghai/S3084/2014
AJS16529 A/chicken/Shanghai/S3090/2014
AJJ95488 A/chicken/Shantou/1550/2014
AJJ95500 A/chicken/Shantou/1552/2014
AJJ95512 A/chicken/Shantou/1554/2014
AJJ95524 A/chicken/Shantou/1556/2014
AJJ97757 A/chicken/Shantou/2537/2014
AJJ97769 A/chicken/Shantou/2538/2014
AJJ97781 A/chicken/Shantou/2539/2014
AJJ97793 A/chicken/Shantou/2546/2014
AJJ97805 A/chicken/Shantou/2550/2014
AJJ97817 A/chicken/Shantou/2556/2014
AJJ97829 A/chicken/Shantou/2562/2014
AJJ97961 A/chicken/Shantou/3057/2014
AJJ97973 A/chicken/Shantou/4325/2014
AJJ97998 A/chicken/Shantou/4816/2014
AJJ98010 A/chicken/Shantou/4824/2014
AJJ98022 A/chicken/Shantou/4832/2014
AJJ98034 A/chicken/Shantou/4833/2014
AJJ91314 A/chicken/Shaoxing/2417/2013
AJJ91653 A/chicken/Shaoxing/5086/2013
AJJ91665 A/chicken/Shaoxing/5087/2013
AJJ91689 A/chicken/Shaoxing/5136/2013
AJJ91701 A/chicken/Shaoxing/5146/2013
AJJ91713 A/chicken/Shaoxing/5186/2013
AJJ91725 A/chicken/Shaoxing/5201/2013
AJJ91737 A/chicken/Shaoxing/5224/2013
AJJ91749 A/chicken/Shaoxing/5227/2013
AJJ91787 A/chicken/Shaoxing/5479/2013
AJJ95464 A/chicken/Shenzhen/138/2014
AJJ90576 A/chicken/Shenzhen/1665/2013
AJJ90588 A/chicken/Shenzhen/2110/2013
AJJ90637 A/chicken/Shenzhen/2201/2013
AJJ90649 A/chicken/Shenzhen/2293/2013
AJJ91011 A/chicken/Shenzhen/3733/2013
AJJ91023 A/chicken/Shenzhen/3734/2013
AJJ91035 A/chicken/Shenzhen/3780/2013
AJJ90478 A/chicken/Shenzhen/727/2013
AJJ90490 A/chicken/Shenzhen/742/2013
AJJ90502 A/chicken/Shenzhen/747/2013
AJJ90514 A/chicken/Shenzhen/749/2013
AJJ92005 A/chicken/Shenzhen/801/2013
AJJ90526 A/chicken/Shenzhen/898/2013
AIU47013 A/chicken/Suzhou/040201H/2013
ARB51605 A/chicken/Tennessee/17-007147-2/2017
ARB51617 A/chicken/Tennessee/17-007431-3/2017
ARB51641 A/chicken/Tennessee/17-008279-4/2017
AJJ98510 A/chicken/Zhangzhou/8585/2014
AJJ98522 A/chicken/Zhangzhou/8829/2014
AGN69410 A/chicken/Zhejiang/C481/2013
AGN69400 A/chicken/Zhejiang/C483/2013
AGJ72861 A/chicken/Zhejiang/DTID-ZJU01/2013
AIU46619 A/chicken/Zhejiang/DTID-ZJU06/2013
AGR49530 A/chicken/Zhejiang/SD007/2013
AGR49542 A/chicken/Zhejiang/SD019/2013
AGR49554 A/chicken/Zhejiang/SD033/2013
AGR49566 A/duck/Anhui/SC702/2013
AIU47001 A/duck/Sunan/040802G/2013
AJK00354 A/duck/Zhejiang/LS02/2014
AGR49578 A/duck/Zhejiang/SC410/2013
AJK00245 A/goose/Jiangsu/1027/2013 2013/04/06 HA
ARB51629 A/guinea fowl/Alabama/17-008272-2/2017
AGR49722 A/homing pigeon/Jiangsu/SD184/2013
AGR49734 A/pigeon/Shanghai/S1069/2013
AGR49746 A/pigeon/Shanghai/S1421/2013
AGR49758 A/pigeon/Shanghai/S1423/2013
AIU46989 A/pigeon/Wuxi/0405007G/2013
AGN69430 A/pigeon/Zhejiang/P1/2013
AGN69420 A/pigeon/Zhejiang/P2/2013
AJJ94194 A/silkie chicken/Dongguan/1264/2014
AJJ94206 A/silkie chicken/Dongguan/1268/2014
AJJ95135 A/silkie chicken/Dongguan/1271/2014
AJJ94218 A/silkie chicken/Dongguan/1274/2014
AJJ94305 A/silkie chicken/Dongguan/1448/2014
AJJ94332 A/silkie chicken/Dongguan/1450/2014
AJJ94344 A/silkie chicken/Dongguan/1451/2014
AJJ94432 A/silkie chicken/Dongguan/1516/2014
AJJ95584 A/silkie chicken/Dongguan/1519/2014
AJJ93845 A/silkie chicken/Dongguan/157/2014
AJJ95358 A/silkie chicken/Dongguan/1641/2014
AJJ90673 A/silkie chicken/Dongguan/3049/2013
AJJ90733 A/silkie chicken/Dongguan/3166/2013
AJJ90783 A/silkie chicken/Dongguan/3275/2013
AJJ90795 A/silkie chicken/Dongguan/3281/2013
AJJ90807 A/silkie chicken/Dongguan/3284/2013
AJJ90891 A/silkie chicken/Dongguan/3520/2013
AJJ90903 A/silkie chicken/Dongguan/3522/2013
AJJ90915 A/silkie chicken/Dongguan/3525/2013
AJJ90927 A/silkie chicken/Dongguan/3526/2013
AJJ90939 A/silkie chicken/Dongguan/3528/2013
AJJ90987 A/silkie chicken/Dongguan/3605/2013
AJJ90999 A/silkie chicken/Dongguan/3606/2013
AJJ91131 A/silkie chicken/Dongguan/3980/2013
AJJ91143 A/silkie chicken/Dongguan/3990/2013
AJJ91227 A/silkie chicken/Dongguan/4126/2013
AJJ91239 A/silkie chicken/Dongguan/4127/2013
AJJ91264 A/silkie chicken/Dongguan/4129/2013
AJJ95440 A/silkie chicken/Dongguan/523/2014
AJJ93967 A/silkie chicken/Dongguan/635/2014
AJJ93979 A/silkie chicken/Dongguan/656/2014

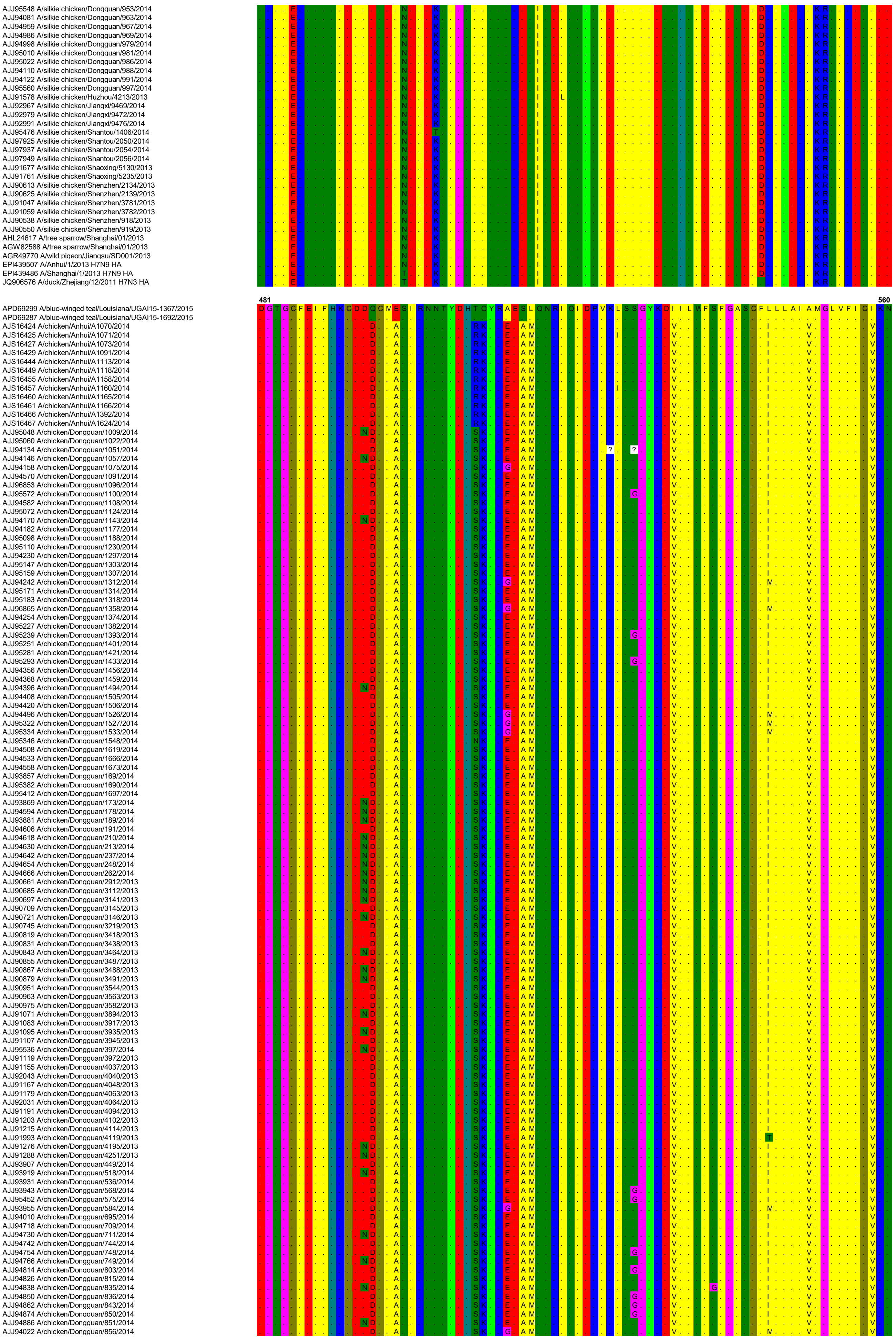
AJJ95548 A/silkie chicken/Dongguan/953/2014
AJJ94081 A/silkie chicken/Dongguan/963/2014
AJJ94959 A/silkie chicken/Dongguan/967/2014
AJJ94986 A/silkie chicken/Dongguan/969/2014
AJJ94998 A/silkie chicken/Dongguan/979/2014
AJJ95010 A/silkie chicken/Dongguan/981/2014
AJJ95022 A/silkie chicken/Dongguan/986/2014
AJJ94110 A/silkie chicken/Dongguan/988/2014
AJJ94122 A/silkie chicken/Dongguan/991/2014
AJJ95560 A/silkie chicken/Dongguan/997/2014
AJJ91578 A/silkie chicken/Huzhou/4213/2013
AJJ92967 A/silkie chicken/Jiangxi/9469/2014
AJJ92979 A/silkie chicken/Jiangxi/9472/2014
AJJ92991 A/silkie chicken/Jiangxi/9476/2014
AJJ95476 A/silkie chicken/Shantou/1406/2014
AJJ97925 A/silkie chicken/Shantou/2050/2014
AJJ97937 A/silkie chicken/Shantou/2054/2014
AJJ97949 A/silkie chicken/Shantou/2056/2014
AJJ91677 A/silkie chicken/Shaoxing/5130/2013
AJJ91761 A/silkie chicken/Shaoxing/5235/2013
AJJ90613 A/silkie chicken/Shenzhen/2134/2013
AJJ90625 A/silkie chicken/Shenzhen/2139/2013
AJJ91047 A/silkie chicken/Shenzhen/3781/2013
AJJ91059 A/silkie chicken/Shenzhen/3782/2013
AJJ90538 A/silkie chicken/Shenzhen/918/2013
AJJ90550 A/silkie chicken/Shenzhen/919/2013
AHL24617 A/tree sparrow/Shanghai/01/2013
AGW82588 A/tree sparrow/Shanghai/01/2013
AGR49770 A/wild pigeon/Jiangsu/SD001/2013
EPI439507 A/Anhui/1/2013 H7N9 HA
EPI439486 A/Shanghai/1/2013 H7N9 HA
JQ906576 A/duck/Zhejiang/12/2011 H7N3 HA
481
560
APD69299 A/blue-winged teal/Louisiana/UGAI15-1367/2015
APD69287 A/blue-winged teal/Louisiana/UGAI15-1692/2015
AJS16424 A/chicken/Anhui/A1070/2014
AJS16425 A/chicken/Anhui/A1071/2014
AJS16427 A/chicken/Anhui/A1073/2014
AJS16429 A/chicken/Anhui/A1091/2014
AJS16444 A/chicken/Anhui/A1113/2014
AJS16449 A/chicken/Anhui/A1118/2014
AJS16455 A/chicken/Anhui/A1158/2014
AJS16457 A/chicken/Anhui/A1160/2014
AJS16460 A/chicken/Anhui/A1165/2014
AJS16461 A/chicken/Anhui/A1166/2014
AJS16466 A/chicken/Anhui/A1392/2014
AJS16467 A/chicken/Anhui/A1624/2014
AJJ95048 A/chicken/Dongguan/1009/2014
AJJ95060 A/chicken/Dongguan/1022/2014
AJJ94134 A/chicken/Dongguan/1051/2014
AJJ94146 A/chicken/Dongguan/1057/2014
AJJ94158 A/chicken/Dongguan/1075/2014
AJJ94570 A/chicken/Dongguan/1091/2014
AJJ96853 A/chicken/Dongguan/1096/2014
AJJ95572 A/chicken/Dongguan/1100/2014
AJJ94582 A/chicken/Dongguan/1108/2014
AJJ95072 A/chicken/Dongguan/1124/2014
AJJ94170 A/chicken/Dongguan/1143/2014
AJJ94182 A/chicken/Dongguan/1177/2014
AJJ95098 A/chicken/Dongguan/1188/2014
AJJ95110 A/chicken/Dongguan/1230/2014
AJJ94230 A/chicken/Dongguan/1297/2014
AJJ95147 A/chicken/Dongguan/1303/2014
AJJ95159 A/chicken/Dongguan/1307/2014
AJJ94242 A/chicken/Dongguan/1312/2014
AJJ95171 A/chicken/Dongguan/1314/2014
AJJ95183 A/chicken/Dongguan/1318/2014
AJJ96865 A/chicken/Dongguan/1358/2014
AJJ94254 A/chicken/Dongguan/1374/2014
AJJ95227 A/chicken/Dongguan/1382/2014
AJJ95239 A/chicken/Dongguan/1393/2014
AJJ95251 A/chicken/Dongguan/1401/2014
AJJ95281 A/chicken/Dongguan/1421/2014
AJJ95293 A/chicken/Dongguan/1433/2014
AJJ94356 A/chicken/Dongguan/1456/2014
AJJ94368 A/chicken/Dongguan/1459/2014
AJJ94396 A/chicken/Dongguan/1494/2014
AJJ94408 A/chicken/Dongguan/1505/2014
AJJ94420 A/chicken/Dongguan/1506/2014
AJJ94496 A/chicken/Dongguan/1526/2014
AJJ95322 A/chicken/Dongguan/1527/2014
AJJ95334 A/chicken/Dongguan/1533/2014
AJJ95346 A/chicken/Dongguan/1548/2014
AJJ94508 A/chicken/Dongguan/1619/2014
AJJ94533 A/chicken/Dongguan/1666/2014
AJJ94558 A/chicken/Dongguan/1673/2014
AJJ93857 A/chicken/Dongguan/169/2014
AJJ95382 A/chicken/Dongguan/1690/2014
AJJ95412 A/chicken/Dongguan/1697/2014
AJJ93869 A/chicken/Dongguan/173/2014
AJJ94594 A/chicken/Dongguan/178/2014
AJJ93881 A/chicken/Dongguan/189/2014
AJJ94606 A/chicken/Dongguan/191/2014
AJJ94618 A/chicken/Dongguan/210/2014
AJJ94630 A/chicken/Dongguan/213/2014
AJJ94642 A/chicken/Dongguan/237/2014
AJJ94654 A/chicken/Dongguan/248/2014
AJJ94666 A/chicken/Dongguan/262/2014
AJJ90661 A/chicken/Dongguan/2912/2013
AJJ90685 A/chicken/Dongguan/3112/2013
AJJ90697 A/chicken/Dongguan/3141/2013
AJJ90709 A/chicken/Dongguan/3145/2013
AJJ90721 A/chicken/Dongguan/3146/2013
AJJ90745 A/chicken/Dongguan/3219/2013
AJJ90819 A/chicken/Dongguan/3418/2013
AJJ90831 A/chicken/Dongguan/3438/2013
AJJ90843 A/chicken/Dongguan/3464/2013
AJJ90855 A/chicken/Dongguan/3487/2013
AJJ90867 A/chicken/Dongguan/3488/2013
AJJ90879 A/chicken/Dongguan/3491/2013
AJJ90951 A/chicken/Dongguan/3544/2013
AJJ90963 A/chicken/Dongguan/3563/2013
AJJ90975 A/chicken/Dongguan/3582/2013
AJJ91071 A/chicken/Dongguan/3894/2013
AJJ91083 A/chicken/Dongguan/3917/2013
AJJ91095 A/chicken/Dongguan/3935/2013
AJJ91107 A/chicken/Dongguan/3945/2013
AJJ95536 A/chicken/Dongguan/397/2014
AJJ91119 A/chicken/Dongguan/3972/2013
AJJ91155 A/chicken/Dongguan/4037/2013
AJJ92043 A/chicken/Dongguan/4040/2013
AJJ91167 A/chicken/Dongguan/4048/2013
AJJ91179 A/chicken/Dongguan/4063/2013
AJJ92031 A/chicken/Dongguan/4064/2013
AJJ91191 A/chicken/Dongguan/4094/2013
AJJ91203 A/chicken/Dongguan/4102/2013
AJJ91215 A/chicken/Dongguan/4114/2013
AJJ91993 A/chicken/Dongguan/4119/2013
AJJ91276 A/chicken/Dongguan/4195/2013
AJJ91288 A/chicken/Dongguan/4251/2013
AJJ93907 A/chicken/Dongguan/449/2014
AJJ93919 A/chicken/Dongguan/518/2014
AJJ93931 A/chicken/Dongguan/536/2014
AJJ93943 A/chicken/Dongguan/568/2014
AJJ95452 A/chicken/Dongguan/575/2014
AJJ93955 A/chicken/Dongguan/584/2014
AJJ94010 A/chicken/Dongguan/695/2014
AJJ94718 A/chicken/Dongguan/709/2014
AJJ94730 A/chicken/Dongguan/711/2014
AJJ94742 A/chicken/Dongguan/744/2014
AJJ94754 A/chicken/Dongguan/748/2014
AJJ94766 A/chicken/Dongguan/749/2014
AJJ94814 A/chicken/Dongguan/803/2014
AJJ94826 A/chicken/Dongguan/815/2014
AJJ94838 A/chicken/Dongguan/835/2014
AJJ94850 A/chicken/Dongguan/836/2014
AJJ94862 A/chicken/Dongguan/843/2014
AJJ94874 A/chicken/Dongguan/850/2014
AJJ94886 A/chicken/Dongguan/851/2014
AJJ94022 A/chicken/Dongguan/856/2014

AJJ94898 A/chicken/Dongguan/864/2014
AJJ94910 A/chicken/Dongguan/874/2014
AJJ94922 A/chicken/Dongguan/899/2014
AJJ94934 A/chicken/Dongguan/934/2014
AHK10583 A/chicken/Guangdong/3135/2013
AHK10584 A/chicken/Guangdong/3640/2013
AHK10585 A/chicken/Guangdong/G1/2013
AJS16473 A/chicken/Guangdong/G1519/2014
AJS16474 A/chicken/Guangdong/G1521/2014
AJS16475 A/chicken/Guangdong/G1523/2014
AHK10586 A/chicken/Guangdong/G2/2013
AHK10587 A/chicken/Guangdong/G3/2013
AHK10588 A/chicken/Guangdong/SD1/2013
AGR49339 A/chicken/Guangdong/SD641/2013
AHL21385 A/chicken/Guangzhou/1/2013
AJJ91326 A/chicken/Huzhou/3765/2013
AJJ91338 A/chicken/Huzhou/3791/2013
AJJ91350 A/chicken/Huzhou/3802/2013
AJJ91402 A/chicken/Huzhou/4045/2013
AJJ91414 A/chicken/Huzhou/4067/2013
AJJ91439 A/chicken/Huzhou/4073/2013
AJJ91451 A/chicken/Huzhou/4074/2013
AJJ91476 A/chicken/Huzhou/4076/2013
AJJ91515 A/chicken/Huzhou/4083/2013
AJJ91527 A/chicken/Huzhou/4141/2013
AJJ91539 A/chicken/Huzhou/4169/2013
AHD25275 A/chicken/Jiangsu/1021/2013
AJS16519 A/chicken/Jiangsu/J3899/2014
AGR49351 A/chicken/Jiangsu/S002/2013
AGR49363 A/chicken/Jiangsu/SC035/2013
AGR49375 A/chicken/Jiangsu/SC099/2013
AGR49387 A/chicken/Jiangsu/SC537/2013
AJJ93039 A/chicken/Jiangxi/10552/2014
AJJ93051 A/chicken/Jiangxi/10573/2014
AJJ93075 A/chicken/Jiangxi/10870/2014
AJJ93087 A/chicken/Jiangxi/10871/2014
AJJ93099 A/chicken/Jiangxi/10873/2014
AJJ93111 A/chicken/Jiangxi/10874/2014
AJJ93123 A/chicken/Jiangxi/10875/2014
AJJ93135 A/chicken/Jiangxi/10877/2014
AJJ93147 A/chicken/Jiangxi/10882/2014
AJJ93159 A/chicken/Jiangxi/10885/2014
AJJ93171 A/chicken/Jiangxi/10894/2014
AJJ93183 A/chicken/Jiangxi/10895/2014
AJJ93195 A/chicken/Jiangxi/10896/2014
AJJ93207 A/chicken/Jiangxi/10897/2014
AJJ93219 A/chicken/Jiangxi/10929/2014
AJJ93231 A/chicken/Jiangxi/10939/2014
AJJ93243 A/chicken/Jiangxi/10943/2014
AJJ93255 A/chicken/Jiangxi/10945/2014
AJJ93267 A/chicken/Jiangxi/10946/2014
AJJ93279 A/chicken/Jiangxi/10947/2014
AJJ93291 A/chicken/Jiangxi/10948/2014
AJJ93303 A/chicken/Jiangxi/10950/2014
AJJ93315 A/chicken/Jiangxi/10953/2014
AJJ93327 A/chicken/Jiangxi/10954/2014
AJJ93339 A/chicken/Jiangxi/10955/2014
AJJ93351 A/chicken/Jiangxi/10956/2014
AJJ93363 A/chicken/Jiangxi/10957/2014
AJJ93375 A/chicken/Jiangxi/10958/2014
AJJ93387 A/chicken/Jiangxi/10959/2014
AJJ93399 A/chicken/Jiangxi/10961/2014
AJJ93411 A/chicken/Jiangxi/10962/2014
AJJ93423 A/chicken/Jiangxi/10963/2014
AJJ93435 A/chicken/Jiangxi/10964/2014
AJJ93447 A/chicken/Jiangxi/10965/2014
AJJ96805 A/chicken/Jiangxi/12200/2014
AJJ97063 A/chicken/Jiangxi/12201/2014
AJJ96552 A/chicken/Jiangxi/12206/2014
AJJ97075 A/chicken/Jiangxi/12208/2014
AJJ96564 A/chicken/Jiangxi/12210/2014
AJJ96576 A/chicken/Jiangxi/12216/2014
AJJ97087 A/chicken/Jiangxi/12217/2014
AJJ97099 A/chicken/Jiangxi/12219/2014
AJJ96588 A/chicken/Jiangxi/12221/2014
AJJ97111 A/chicken/Jiangxi/12222/2014
AJJ96600 A/chicken/Jiangxi/12223/2014
AJJ97123 A/chicken/Jiangxi/12232/2014
AJJ97135 A/chicken/Jiangxi/12239/2014
AJJ96708 A/chicken/Jiangxi/12240/2014
AJJ97147 A/chicken/Jiangxi/12243/2014
AJJ96612 A/chicken/Jiangxi/12245/2014
AJJ97159 A/chicken/Jiangxi/12247/2014
AJJ97171 A/chicken/Jiangxi/12248/2014
AJJ96624 A/chicken/Jiangxi/12249/2014
AJJ97183 A/chicken/Jiangxi/12251/2014
AJJ96636 A/chicken/Jiangxi/12254/2014
AJJ97195 A/chicken/Jiangxi/12256/2014
AJJ97207 A/chicken/Jiangxi/12260/2014
AJJ96648 A/chicken/Jiangxi/12261/2014
AJJ97219 A/chicken/Jiangxi/12264/2014
AJJ97231 A/chicken/Jiangxi/12265/2014
AJJ97243 A/chicken/Jiangxi/12273/2014
AJJ96660 A/chicken/Jiangxi/12274/2014
AJJ91811 A/chicken/Jiangxi/12486/2013
AJJ91823 A/chicken/Jiangxi/12492/2013
AJJ91849 A/chicken/Jiangxi/12544/2013
AJJ91861 A/chicken/Jiangxi/12554/2013
AJJ96258 A/chicken/Jiangxi/12564/2013
AJJ96672 A/chicken/Jiangxi/12768/2014
AJJ96684 A/chicken/Jiangxi/13207/2014
AJJ96696 A/chicken/Jiangxi/13209/2014
AJJ97255 A/chicken/Jiangxi/13210/2014
AJJ96720 A/chicken/Jiangxi/13220/2014
AJJ96732 A/chicken/Jiangxi/13223/2014
AJJ96744 A/chicken/Jiangxi/13230/2014
AJJ96769 A/chicken/Jiangxi/13250/2014
AJJ97267 A/chicken/Jiangxi/13252/2014
AJJ96781 A/chicken/Jiangxi/13255/2014
AJJ96793 A/chicken/Jiangxi/13268/2014
AJJ97279 A/chicken/Jiangxi/13269/2014
AJJ96877 A/chicken/Jiangxi/13491/2014
AJJ97291 A/chicken/Jiangxi/13493/2014
AJJ96889 A/chicken/Jiangxi/13496/2014
AJJ96901 A/chicken/Jiangxi/13502/2014
AJJ97319 A/chicken/Jiangxi/13507/2014
AJJ96913 A/chicken/Jiangxi/13510/2014
AJJ97331 A/chicken/Jiangxi/13512/2014
AJJ96925 A/chicken/Jiangxi/13513/2014
AJJ96937 A/chicken/Jiangxi/13518/2014
AJJ96949 A/chicken/Jiangxi/13519/2014
AJJ97373 A/chicken/Jiangxi/13521/2014
AJJ97385 A/chicken/Jiangxi/13524/2014
AJJ97443 A/chicken/Jiangxi/13530/2014
AJJ97455 A/chicken/Jiangxi/13536/2014
AJJ97467 A/chicken/Jiangxi/13537/2014
AJJ96978 A/chicken/Jiangxi/13538/2014
AJJ97493 A/chicken/Jiangxi/13543/2014
AJJ97505 A/chicken/Jiangxi/13544/2014
AJJ96990 A/chicken/Jiangxi/13546/2014
AJJ97517 A/chicken/Jiangxi/13548/2014
AJJ97529 A/chicken/Jiangxi/13551/2014
AJJ97002 A/chicken/Jiangxi/13553/2014
AJJ97558 A/chicken/Jiangxi/13556/2014
AJJ97570 A/chicken/Jiangxi/13564/2014
AJJ97582 A/chicken/Jiangxi/14023/2014
AJJ97594 A/chicken/Jiangxi/14033/2014
AJJ97606 A/chicken/Jiangxi/14479/2014
AJJ97618 A/chicken/Jiangxi/14482/2014
AJJ97673 A/chicken/Jiangxi/14513/2014
AJJ97685 A/chicken/Jiangxi/14515/2014
AJJ97697 A/chicken/Jiangxi/14517/2014
AJJ97709 A/chicken/Jiangxi/14518/2014
AJJ97721 A/chicken/Jiangxi/14530/2014
AJJ97745 A/chicken/Jiangxi/14554/2014
AJJ97841 A/chicken/Jiangxi/15044/2014
AJJ97899 A/chicken/Jiangxi/15524/2014
AJJ98081 A/chicken/Jiangxi/18008/2014
AJJ98120 A/chicken/Jiangxi/18449/2014
AJJ98227 A/chicken/Jiangxi/18482/2014
AJJ98275 A/chicken/Jiangxi/18487/2014

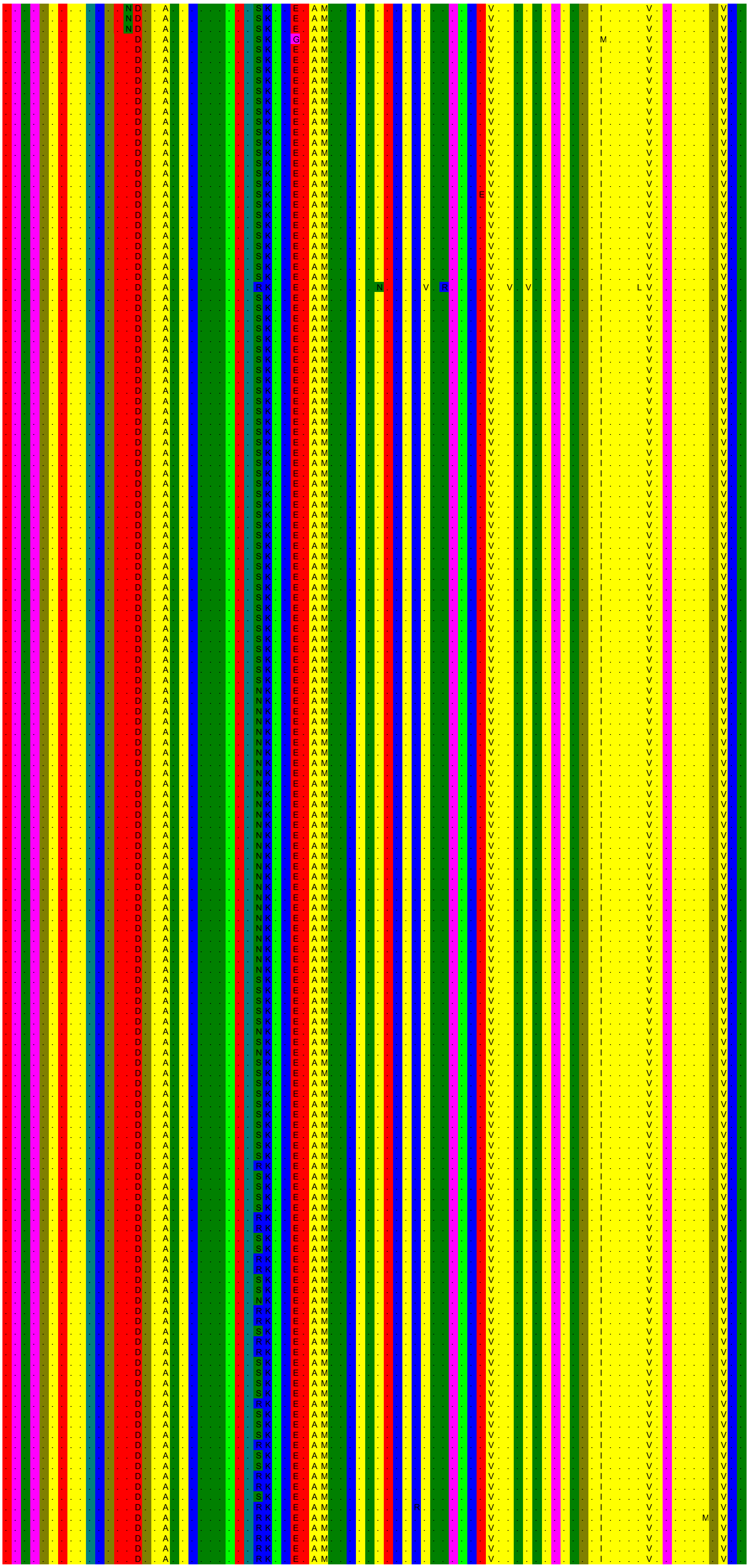

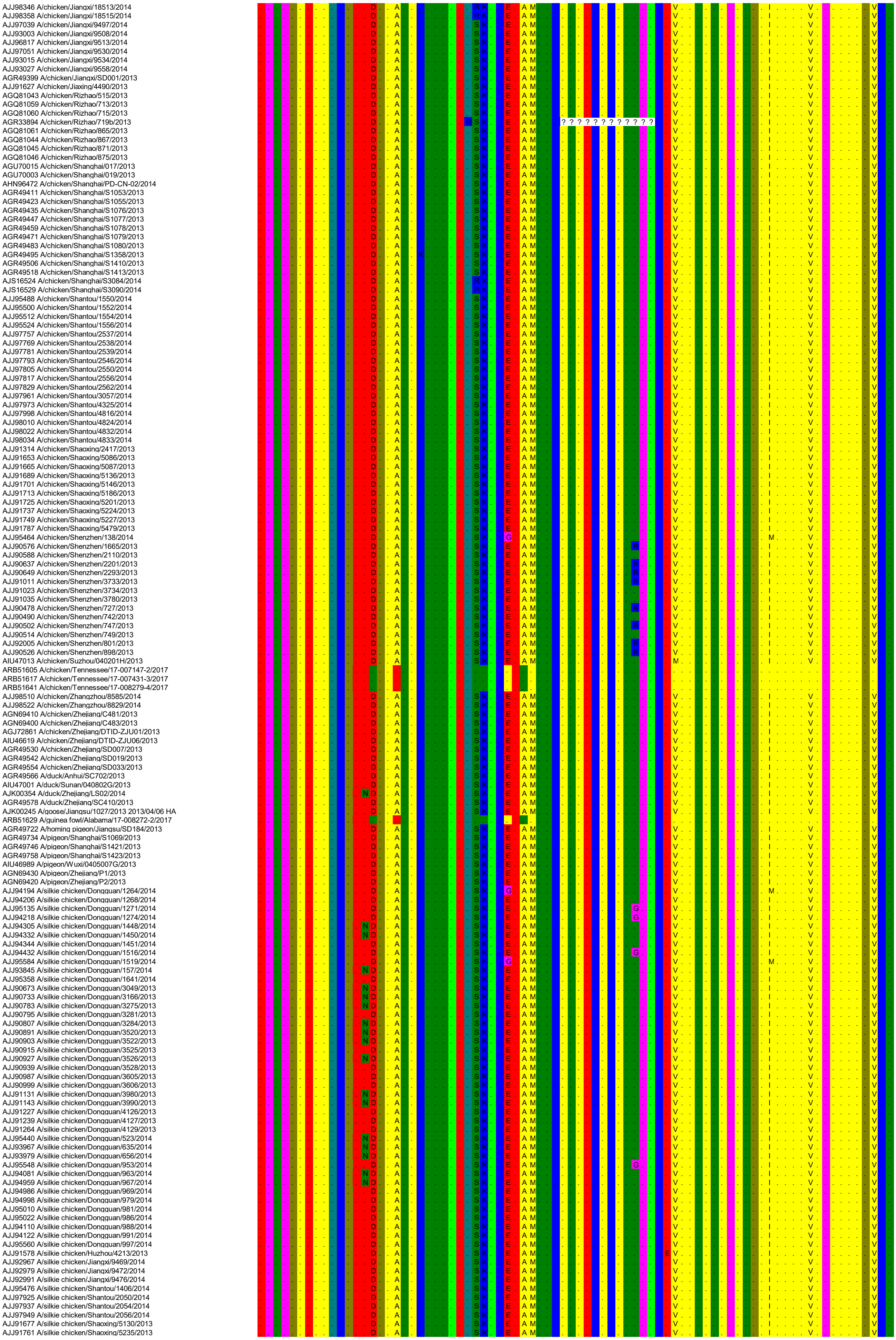
AJJ98346 A/chicken/Jiangxi/18513/2014
AJJ98358 A/chicken/Jiangxi/18515/2014
AJJ97039 A/chicken/Jiangxi/9497/2014
AJJ93003 A/chicken/Jiangxi/9508/2014
AJJ96817 A/chicken/Jiangxi/9513/2014
AJJ97051 A/chicken/Jiangxi/9530/2014
AJJ93015 A/chicken/Jiangxi/9534/2014
AJJ93027 A/chicken/Jiangxi/9558/2014
AGR49399 A/chicken/Jiangxi/SD001/2013
AJJ91627 A/chicken/Jiaxing/4490/2013
AGQ81043 A/chicken/Rizhao/515/2013
AGQ81059 A/chicken/Rizhao/713/2013
AGQ81060 A/chicken/Rizhao/715/2013
AGR33894 A/chicken/Rizhao/719b/2013
AGQ81061 A/chicken/Rizhao/865/2013
AGQ81044 A/chicken/Rizhao/867/2013
AGQ81045 A/chicken/Rizhao/871/2013
AGQ81046 A/chicken/Rizhao/875/2013
AGU70015 A/chicken/Shanghai/017/2013
AGU70003 A/chicken/Shanghai/019/2013
AHN96472 A/chicken/Shanghai/PD-CN-02/2014
AGR49411 A/chicken/Shanghai/S1053/2013
AGR49423 A/chicken/Shanghai/S1055/2013
AGR49435 A/chicken/Shanghai/S1076/2013
AGR49447 A/chicken/Shanghai/S1077/2013
AGR49459 A/chicken/Shanghai/S1078/2013
AGR49471 A/chicken/Shanghai/S1079/2013
AGR49483 A/chicken/Shanghai/S1080/2013
AGR49495 A/chicken/Shanghai/S1358/2013
AGR49506 A/chicken/Shanghai/S1410/2013
AGR49518 A/chicken/Shanghai/S1413/2013
AJS16524 A/chicken/Shanghai/S3084/2014
AJS16529 A/chicken/Shanghai/S3090/2014
AJJ95488 A/chicken/Shantou/1550/2014
AJJ95500 A/chicken/Shantou/1552/2014
AJJ95512 A/chicken/Shantou/1554/2014
AJJ95524 A/chicken/Shantou/1556/2014
AJJ97757 A/chicken/Shantou/2537/2014
AJJ97769 A/chicken/Shantou/2538/2014
AJJ97781 A/chicken/Shantou/2539/2014
AJJ97793 A/chicken/Shantou/2546/2014
AJJ97805 A/chicken/Shantou/2550/2014
AJJ97817 A/chicken/Shantou/2556/2014
AJJ97829 A/chicken/Shantou/2562/2014
AJJ97961 A/chicken/Shantou/3057/2014
AJJ97973 A/chicken/Shantou/4325/2014
AJJ97998 A/chicken/Shantou/4816/2014
AJJ98010 A/chicken/Shantou/4824/2014
AJJ98022 A/chicken/Shantou/4832/2014
AJJ98034 A/chicken/Shantou/4833/2014
AJJ91314 A/chicken/Shaoxing/2417/2013
AJJ91653 A/chicken/Shaoxing/5086/2013
AJJ91665 A/chicken/Shaoxing/5087/2013
AJJ91689 A/chicken/Shaoxing/5136/2013
AJJ91701 A/chicken/Shaoxing/5146/2013
AJJ91713 A/chicken/Shaoxing/5186/2013
AJJ91725 A/chicken/Shaoxing/5201/2013
AJJ91737 A/chicken/Shaoxing/5224/2013
AJJ91749 A/chicken/Shaoxing/5227/2013
AJJ91787 A/chicken/Shaoxing/5479/2013
AJJ95464 A/chicken/Shenzhen/138/2014
AJJ90576 A/chicken/Shenzhen/1665/2013
AJJ90588 A/chicken/Shenzhen/2110/2013
AJJ90637 A/chicken/Shenzhen/2201/2013
AJJ90649 A/chicken/Shenzhen/2293/2013
AJJ91011 A/chicken/Shenzhen/3733/2013
AJJ91023 A/chicken/Shenzhen/3734/2013
AJJ91035 A/chicken/Shenzhen/3780/2013
AJJ90478 A/chicken/Shenzhen/727/2013
AJJ90490 A/chicken/Shenzhen/742/2013
AJJ90502 A/chicken/Shenzhen/747/2013
AJJ90514 A/chicken/Shenzhen/749/2013
AJJ92005 A/chicken/Shenzhen/801/2013
AJJ90526 A/chicken/Shenzhen/898/2013
AIU47013 A/chicken/Suzhou/040201H/2013
ARB51605 A/chicken/Tennessee/17-007147-2/2017
ARB51617 A/chicken/Tennessee/17-007431-3/2017
ARB51641 A/chicken/Tennessee/17-008279-4/2017
AJJ98510 A/chicken/Zhangzhou/8585/2014
AJJ98522 A/chicken/Zhangzhou/8829/2014
AGN69410 A/chicken/Zhejiang/C481/2013
AGN69400 A/chicken/Zhejiang/C483/2013
AGJ72861 A/chicken/Zhejiang/DTID-ZJU01/2013
AIU46619 A/chicken/Zhejiang/DTID-ZJU06/2013
AGR49530 A/chicken/Zhejiang/SD007/2013
AGR49542 A/chicken/Zhejiang/SD019/2013
AGR49554 A/chicken/Zhejiang/SD033/2013
AGR49566 A/duck/Anhui/SC702/2013
AIU47001 A/duck/Sunan/040802G/2013
AJK00354 A/duck/Zhejiang/LS02/2014
AGR49578 A/duck/Zhejiang/SC410/2013
AJK00245 A/goose/Jiangsu/1027/2013 2013/04/06 HA
ARB51629 A/guinea fowl/Alabama/17-008272-2/2017
AGR49722 A/homing pigeon/Jiangsu/SD184/2013
AGR49734 A/pigeon/Shanghai/S1069/2013
AGR49746 A/pigeon/Shanghai/S1421/2013
AGR49758 A/pigeon/Shanghai/S1423/2013
AIU46989 A/pigeon/Wuxi/0405007G/2013
AGN69430 A/pigeon/Zhejiang/P1/2013
AGN69420 A/pigeon/Zhejiang/P2/2013
AJJ94194 A/silkie chicken/Dongguan/1264/2014
AJJ94206 A/silkie chicken/Dongguan/1268/2014
AJJ95135 A/silkie chicken/Dongguan/1271/2014
AJJ94218 A/silkie chicken/Dongguan/1274/2014
AJJ94305 A/silkie chicken/Dongguan/1448/2014
AJJ94332 A/silkie chicken/Dongguan/1450/2014
AJJ94344 A/silkie chicken/Dongguan/1451/2014
AJJ94432 A/silkie chicken/Dongguan/1516/2014
AJJ95584 A/silkie chicken/Dongguan/1519/2014
AJJ93845 A/silkie chicken/Dongguan/157/2014
AJJ95358 A/silkie chicken/Dongguan/1641/2014
AJJ90673 A/silkie chicken/Dongguan/3049/2013
AJJ90733 A/silkie chicken/Dongguan/3166/2013
AJJ90783 A/silkie chicken/Dongguan/3275/2013
AJJ90795 A/silkie chicken/Dongguan/3281/2013
AJJ90807 A/silkie chicken/Dongguan/3284/2013
AJJ90891 A/silkie chicken/Dongguan/3520/2013
AJJ90903 A/silkie chicken/Dongguan/3522/2013
AJJ90915 A/silkie chicken/Dongguan/3525/2013
AJJ90927 A/silkie chicken/Dongguan/3526/2013
AJJ90939 A/silkie chicken/Dongguan/3528/2013
AJJ90987 A/silkie chicken/Dongguan/3605/2013
AJJ90999 A/silkie chicken/Dongguan/3606/2013
AJJ91131 A/silkie chicken/Dongguan/3980/2013
AJJ91143 A/silkie chicken/Dongguan/3990/2013
AJJ91227 A/silkie chicken/Dongguan/4126/2013
AJJ91239 A/silkie chicken/Dongguan/4127/2013
AJJ91264 A/silkie chicken/Dongguan/4129/2013
AJJ95440 A/silkie chicken/Dongguan/523/2014
AJJ93967 A/silkie chicken/Dongguan/635/2014
AJJ93979 A/silkie chicken/Dongguan/656/2014
AJJ95548 A/silkie chicken/Dongguan/953/2014
AJJ94081 A/silkie chicken/Dongguan/963/2014
AJJ94959 A/silkie chicken/Dongguan/967/2014
AJJ94986 A/silkie chicken/Dongguan/969/2014
AJJ94998 A/silkie chicken/Dongguan/979/2014
AJJ95010 A/silkie chicken/Dongguan/981/2014
AJJ95022 A/silkie chicken/Dongguan/986/2014
AJJ94110 A/silkie chicken/Dongguan/988/2014
AJJ94122 A/silkie chicken/Dongguan/991/2014
AJJ95560 A/silkie chicken/Dongguan/997/2014
AJJ91578 A/silkie chicken/Huzhou/4213/2013
AJJ92967 A/silkie chicken/Jiangxi/9469/2014
AJJ92979 A/silkie chicken/Jiangxi/9472/2014
AJJ92991 A/silkie chicken/Jiangxi/9476/2014
AJJ95476 A/silkie chicken/Shantou/1406/2014
AJJ97925 A/silkie chicken/Shantou/2050/2014
AJJ97937 A/silkie chicken/Shantou/2054/2014
AJJ97949 A/silkie chicken/Shantou/2056/2014
AJJ91677 A/silkie chicken/Shaoxing/5130/2013
AJJ91761 A/silkie chicken/Shaoxing/5235/2013

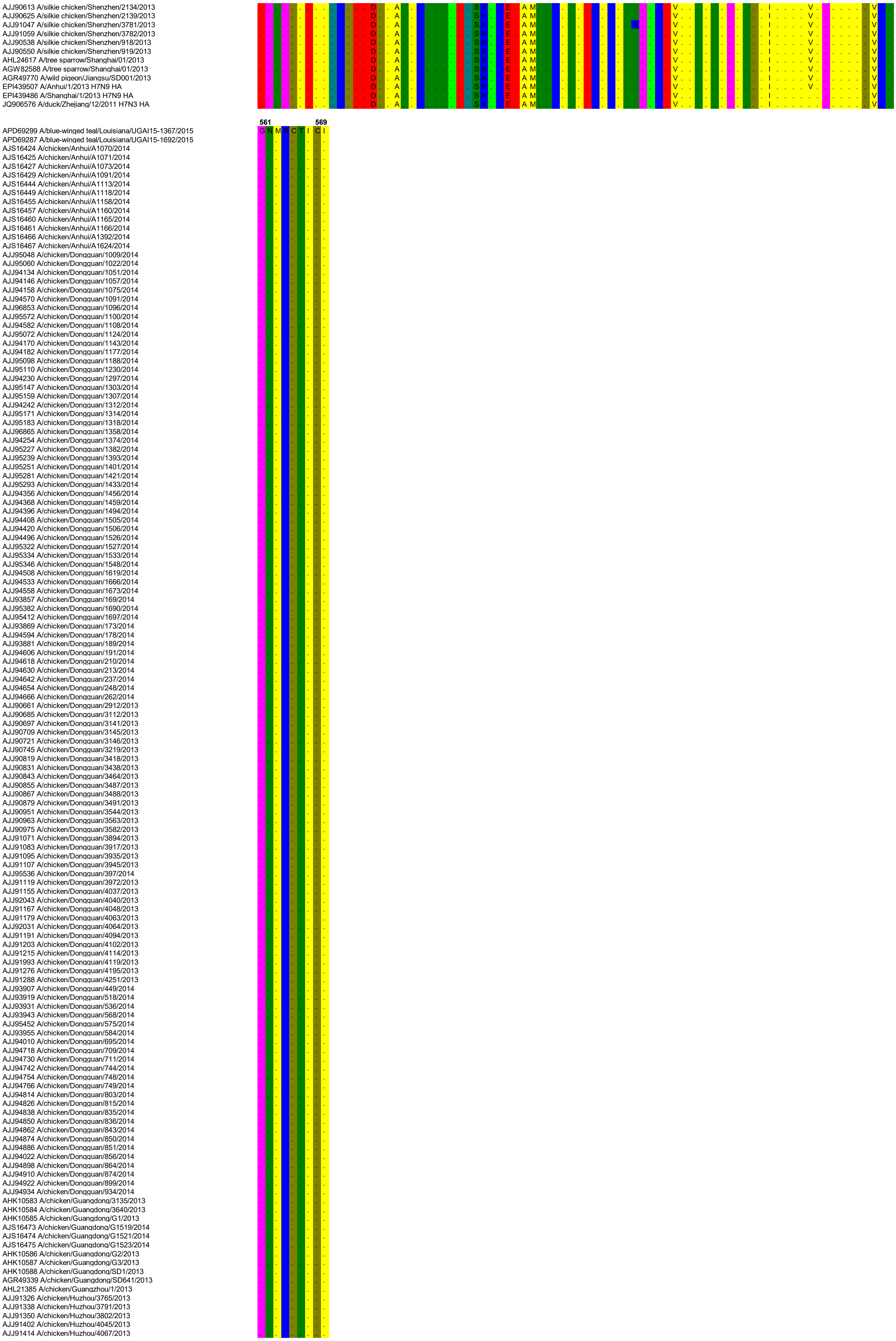
AJJ90613 A/silkie chicken/Shenzhen/2134/2013
AJJ90625 A/silkie chicken/Shenzhen/2139/2013
AJJ91047 A/silkie chicken/Shenzhen/3781/2013
AJJ91059 A/silkie chicken/Shenzhen/3782/2013
AJJ90538 A/silkie chicken/Shenzhen/918/2013
AJJ90550 A/silkie chicken/Shenzhen/919/2013
AHL24617 A/tree sparrow/Shanghai/01/2013
AGW82588 A/tree sparrow/Shanghai/01/2013
AGR49770 A/wild pigeon/Jiangsu/SD001/2013
EPI439507 A/Anhui/1/2013 H7N9 HA
EPI439486 A/Shanghai/1/2013 H7N9 HA
JQ906576 A/duck/Zhejiang/12/2011 H7N3 HA
561
569
G N M R C T I C I
APD69299 A/blue-winged teal/Louisiana/UGAI15-1367/2015
APD69287 A/blue-winged teal/Louisiana/UGAI15-1692/2015
AJS16424 A/chicken/Anhui/A1070/2014
AJS16425 A/chicken/Anhui/A1071/2014
AJS16427 A/chicken/Anhui/A1073/2014
AJS16429 A/chicken/Anhui/A1091/2014
AJS16444 A/chicken/Anhui/A1113/2014
AJS16449 A/chicken/Anhui/A1118/2014
AJS16455 A/chicken/Anhui/A1158/2014
AJS16457 A/chicken/Anhui/A1160/2014
AJS16460 A/chicken/Anhui/A1165/2014
AJS16461 A/chicken/Anhui/A1166/2014
AJS16466 A/chicken/Anhui/A1392/2014
AJS16467 A/chicken/Anhui/A1624/2014
AJJ95048 A/chicken/Dongguan/1009/2014
AJJ95060 A/chicken/Dongguan/1022/2014
AJJ94134 A/chicken/Dongguan/1051/2014
AJJ94146 A/chicken/Dongguan/1057/2014
AJJ94158 A/chicken/Dongguan/1075/2014
AJJ94570 A/chicken/Dongguan/1091/2014
AJJ96853 A/chicken/Dongguan/1096/2014
AJJ95572 A/chicken/Dongguan/1100/2014
AJJ94582 A/chicken/Dongguan/1108/2014
AJJ95072 A/chicken/Dongguan/1124/2014
AJJ94170 A/chicken/Dongguan/1143/2014
AJJ94182 A/chicken/Dongguan/1177/2014
AJJ95098 A/chicken/Dongguan/1188/2014
AJJ95110 A/chicken/Dongguan/1230/2014
AJJ94230 A/chicken/Dongguan/1297/2014
AJJ95147 A/chicken/Dongguan/1303/2014
AJJ95159 A/chicken/Dongguan/1307/2014
AJJ94242 A/chicken/Dongguan/1312/2014
AJJ95171 A/chicken/Dongguan/1314/2014
AJJ95183 A/chicken/Dongguan/1318/2014
AJJ96865 A/chicken/Dongguan/1358/2014
AJJ94254 A/chicken/Dongguan/1374/2014
AJJ95227 A/chicken/Dongguan/1382/2014
AJJ95239 A/chicken/Dongguan/1393/2014
AJJ95251 A/chicken/Dongguan/1401/2014
AJJ95281 A/chicken/Dongguan/1421/2014
AJJ95293 A/chicken/Dongguan/1433/2014
AJJ94356 A/chicken/Dongguan/1456/2014
AJJ94368 A/chicken/Dongguan/1459/2014
AJJ94396 A/chicken/Dongguan/1494/2014
AJJ94408 A/chicken/Dongguan/1505/2014
AJJ94420 A/chicken/Dongguan/1506/2014
AJJ94496 A/chicken/Dongguan/1526/2014
AJJ95322 A/chicken/Dongguan/1527/2014
AJJ95334 A/chicken/Dongguan/1533/2014
AJJ95346 A/chicken/Dongguan/1548/2014
AJJ94508 A/chicken/Dongguan/1619/2014
AJJ94533 A/chicken/Dongguan/1666/2014
AJJ94558 A/chicken/Dongguan/1673/2014
AJJ93857 A/chicken/Dongguan/169/2014
AJJ95382 A/chicken/Dongguan/1690/2014
AJJ95412 A/chicken/Dongguan/1697/2014
AJJ93869 A/chicken/Dongguan/173/2014
AJJ94594 A/chicken/Dongguan/178/2014
AJJ93881 A/chicken/Dongguan/189/2014
AJJ94606 A/chicken/Dongguan/191/2014
AJJ94618 A/chicken/Dongguan/210/2014
AJJ94630 A/chicken/Dongguan/213/2014
AJJ94642 A/chicken/Dongguan/237/2014
AJJ94654 A/chicken/Dongguan/248/2014
AJJ94666 A/chicken/Dongguan/262/2014
AJJ90661 A/chicken/Dongguan/2912/2013
AJJ90685 A/chicken/Dongguan/3112/2013
AJJ90697 A/chicken/Dongguan/3141/2013
AJJ90709 A/chicken/Dongguan/3145/2013
AJJ90721 A/chicken/Dongguan/3146/2013
AJJ90745 A/chicken/Dongguan/3219/2013
AJJ90819 A/chicken/Dongguan/3418/2013
AJJ90831 A/chicken/Dongguan/3438/2013
AJJ90843 A/chicken/Dongguan/3464/2013
AJJ90855 A/chicken/Dongguan/3487/2013
AJJ90867 A/chicken/Dongguan/3488/2013
AJJ90879 A/chicken/Dongguan/3491/2013
AJJ90951 A/chicken/Dongguan/3544/2013
AJJ90963 A/chicken/Dongguan/3563/2013
AJJ90975 A/chicken/Dongguan/3582/2013
AJJ91071 A/chicken/Dongguan/3894/2013
AJJ91083 A/chicken/Dongguan/3917/2013
AJJ91095 A/chicken/Dongguan/3935/2013
AJJ91107 A/chicken/Dongguan/3945/2013
AJJ95536 A/chicken/Dongguan/397/2014
AJJ91119 A/chicken/Dongguan/3972/2013
AJJ91155 A/chicken/Dongguan/4037/2013
AJJ92043 A/chicken/Dongguan/4040/2013
AJJ91167 A/chicken/Dongguan/4048/2013
AJJ91179 A/chicken/Dongguan/4063/2013
AJJ92031 A/chicken/Dongguan/4064/2013
AJJ91191 A/chicken/Dongguan/4094/2013
AJJ91203 A/chicken/Dongguan/4102/2013
AJJ91215 A/chicken/Dongguan/4114/2013
AJJ91993 A/chicken/Dongguan/4119/2013
AJJ91276 A/chicken/Dongguan/4195/2013
AJJ91288 A/chicken/Dongguan/4251/2013
AJJ93907 A/chicken/Dongguan/449/2014
AJJ93919 A/chicken/Dongguan/518/2014
AJJ93931 A/chicken/Dongguan/536/2014
AJJ93943 A/chicken/Dongguan/568/2014
AJJ95452 A/chicken/Dongguan/575/2014
AJJ93955 A/chicken/Dongguan/584/2014
AJJ94010 A/chicken/Dongguan/695/2014
AJJ94718 A/chicken/Dongguan/709/2014
AJJ94730 A/chicken/Dongguan/711/2014
AJJ94742 A/chicken/Dongguan/744/2014
AJJ94754 A/chicken/Dongguan/748/2014
AJJ94766 A/chicken/Dongguan/749/2014
AJJ94814 A/chicken/Dongguan/803/2014
AJJ94826 A/chicken/Dongguan/815/2014
AJJ94838 A/chicken/Dongguan/835/2014
AJJ94850 A/chicken/Dongguan/836/2014
AJJ94862 A/chicken/Dongguan/843/2014
AJJ94874 A/chicken/Dongguan/850/2014
AJJ94886 A/chicken/Dongguan/851/2014
AJJ94022 A/chicken/Dongguan/856/2014
AJJ94898 A/chicken/Dongguan/864/2014
AJJ94910 A/chicken/Dongguan/874/2014
AJJ94922 A/chicken/Dongguan/899/2014
AJJ94934 A/chicken/Dongguan/934/2014
AHK10583 A/chicken/Guangdong/3135/2013
AHK10584 A/chicken/Guangdong/3640/2013
AHK10585 A/chicken/Guangdong/G1/2013
AJS16473 A/chicken/Guangdong/G1519/2014
AJS16474 A/chicken/Guangdong/G1521/2014
AJS16475 A/chicken/Guangdong/G1523/2014
AHK10586 A/chicken/Guangdong/G2/2013
AHK10587 A/chicken/Guangdong/G3/2013
AHK10588 A/chicken/Guangdong/SD1/2013
AGR49339 A/chicken/Guangdong/SD641/2013
AHL21385 A/chicken/Guangzhou/1/2013
AJJ91326 A/chicken/Huzhou/3765/2013
AJJ91338 A/chicken/Huzhou/3791/2013
AJJ91350 A/chicken/Huzhou/3802/2013
AJJ91402 A/chicken/Huzhou/4045/2013
AJJ91414 A/chicken/Huzhou/4067/2013

AJJ91439 A/chicken/Huzhou/4073/2013	.	.	.	.	.	.	.	.	.
AJJ91451 A/chicken/Huzhou/4074/2013	.	.	.	.	.	.	.	.	.
AJJ91476 A/chicken/Huzhou/4076/2013	.	.	.	.	.	.	.	.	.
AJJ91515 A/chicken/Huzhou/4083/2013	.	.	.	.	.	.	.	.	.
AJJ91527 A/chicken/Huzhou/4141/2013	.	.	.	.	.	.	.	.	.
AJJ91539 A/chicken/Huzhou/4169/2013	.	.	.	.	.	.	.	.	.
AHD25275 A/chicken/Jiangsu/1021/2013	.	.	.	.	.	.	.	.	.
AJS16519 A/chicken/Jiangsu/J3899/2014	V	.	I	.	.	A	.	.	.
AGR49351 A/chicken/Jiangsu/S002/2013	.	.	.	.	.	.	.	.	.
AGR49363 A/chicken/Jiangsu/SC035/2013	.	.	.	.	.	.	.	.	.
AGR49375 A/chicken/Jiangsu/SC099/2013	.	.	.	.	.	.	.	.	.
AGR49387 A/chicken/Jiangsu/SC537/2013	.	.	.	.	.	.	.	.	.
AJJ93039 A/chicken/Jiangxi/10552/2014	.	.	.	.	.	.	.	.	.
AJJ93051 A/chicken/Jiangxi/10573/2014	.	.	.	.	.	.	.	.	.
AJJ93075 A/chicken/Jiangxi/10870/2014	.	.	.	.	.	.	.	.	.
AJJ93087 A/chicken/Jiangxi/10871/2014	.	.	.	.	.	.	.	.	.
AJJ93099 A/chicken/Jiangxi/10873/2014	.	.	.	.	.	.	.	.	.
AJJ93111 A/chicken/Jiangxi/10874/2014	.	.	.	.	.	.	.	.	.
AJJ93123 A/chicken/Jiangxi/10875/2014	.	.	.	.	.	.	.	.	.
AJJ93135 A/chicken/Jiangxi/10877/2014	.	.	.	.	.	.	.	.	.
AJJ93147 A/chicken/Jiangxi/10882/2014	.	.	.	.	.	.	.	.	.
AJJ93159 A/chicken/Jiangxi/10885/2014	.	.	.	.	.	.	.	.	.
AJJ93171 A/chicken/Jiangxi/10894/2014	.	.	.	.	.	.	.	.	.
AJJ93183 A/chicken/Jiangxi/10895/2014	.	.	.	.	.	.	.	.	.
AJJ93195 A/chicken/Jiangxi/10896/2014	.	.	.	.	.	.	.	.	.
AJJ93207 A/chicken/Jiangxi/10897/2014	.	.	.	.	.	.	.	.	.
AJJ93219 A/chicken/Jiangxi/10929/2014	.	.	.	.	.	.	.	.	.
AJJ93231 A/chicken/Jiangxi/10939/2014	.	.	.	.	.	.	.	.	.
AJJ93243 A/chicken/Jiangxi/10943/2014	.	.	.	.	.	.	.	.	.
AJJ93255 A/chicken/Jiangxi/10945/2014	.	.	.	.	.	.	.	.	.
AJJ93267 A/chicken/Jiangxi/10946/2014	.	.	.	.	.	.	.	.	.
AJJ93279 A/chicken/Jiangxi/10947/2014	.	.	.	.	.	.	.	.	.
AJJ93291 A/chicken/Jiangxi/10948/2014	.	.	.	.	.	.	.	.	.
AJJ93303 A/chicken/Jiangxi/10950/2014	.	.	.	.	.	.	.	.	.
AJJ93315 A/chicken/Jiangxi/10953/2014	.	.	.	.	.	.	.	.	.
AJJ93327 A/chicken/Jiangxi/10954/2014	.	.	.	.	.	.	.	.	.
AJJ93339 A/chicken/Jiangxi/10955/2014	.	.	.	.	.	.	.	.	.
AJJ93351 A/chicken/Jiangxi/10956/2014	.	.	.	.	.	.	.	.	.
AJJ93363 A/chicken/Jiangxi/10957/2014	.	.	.	.	.	.	.	.	.
AJJ93375 A/chicken/Jiangxi/10958/2014	.	.	.	.	.	.	.	.	.
AJJ93387 A/chicken/Jiangxi/10959/2014	.	.	.	.	.	.	.	.	.
AJJ93399 A/chicken/Jiangxi/10961/2014	.	.	.	.	.	.	.	.	.
AJJ93411 A/chicken/Jiangxi/10962/2014	.	.	.	.	.	.	.	.	.
AJJ93423 A/chicken/Jiangxi/10963/2014	.	.	.	.	.	.	.	.	.
AJJ93435 A/chicken/Jiangxi/10964/2014	.	.	.	.	.	.	.	.	.
AJJ93447 A/chicken/Jiangxi/10965/2014	.	.	.	.	.	.	.	.	.
AJJ96805 A/chicken/Jiangxi/12200/2014	.	.	.	.	.	.	.	.	.
AJJ97063 A/chicken/Jiangxi/12201/2014	.	.	.	.	.	.	.	.	.
AJJ96552 A/chicken/Jiangxi/12206/2014	.	.	.	.	.	.	.	.	.
AJJ97075 A/chicken/Jiangxi/12208/2014	.	.	.	.	.	.	.	.	.
AJJ96564 A/chicken/Jiangxi/12210/2014	.	.	.	.	.	.	.	.	.
AJJ96576 A/chicken/Jiangxi/12216/2014	.	.	.	.	.	.	.	.	.
AJJ97087 A/chicken/Jiangxi/12217/2014	.	.	.	.	.	.	.	.	.
AJJ97099 A/chicken/Jiangxi/12219/2014	.	.	.	.	.	.	.	.	.
AJJ96588 A/chicken/Jiangxi/12221/2014	.	.	.	.	.	.	.	.	.
AJJ97111 A/chicken/Jiangxi/12222/2014	.	.	.	.	.	.	.	.	.
AJJ96600 A/chicken/Jiangxi/12223/2014	.	.	.	.	.	.	.	.	.
AJJ97123 A/chicken/Jiangxi/12232/2014	.	.	.	.	.	.	.	.	.
AJJ97135 A/chicken/Jiangxi/12239/2014	.	.	.	.	.	.	.	.	.
AJJ96708 A/chicken/Jiangxi/12240/2014	.	.	.	.	.	.	.	.	.
AJJ97147 A/chicken/Jiangxi/12243/2014	.	.	.	.	.	.	.	.	.
AJJ96612 A/chicken/Jiangxi/12245/2014	.	.	.	.	.	.	.	.	.
AJJ97159 A/chicken/Jiangxi/12247/2014	.	.	.	.	.	.	.	.	.
AJJ97171 A/chicken/Jiangxi/12248/2014	.	.	.	.	.	.	.	.	.
AJJ96624 A/chicken/Jiangxi/12249/2014	.	.	.	.	.	.	.	.	.
AJJ97183 A/chicken/Jiangxi/12251/2014	.	.	.	.	.	.	.	.	.
AJJ96636 A/chicken/Jiangxi/12254/2014	.	.	.	.	.	.	.	.	.
AJJ97195 A/chicken/Jiangxi/12256/2014	.	.	.	.	.	.	.	.	.
AJJ97207 A/chicken/Jiangxi/12260/2014	.	.	.	.	.	.	.	.	.
AJJ96648 A/chicken/Jiangxi/12261/2014	.	.	.	.	.	.	.	.	.
AJJ97219 A/chicken/Jiangxi/12264/2014	.	.	.	.	.	.	.	.	.
AJJ97231 A/chicken/Jiangxi/12265/2014	.	.	.	.	.	.	.	.	.
AJJ97243 A/chicken/Jiangxi/12273/2014	.	.	.	.	.	.	.	.	.
AJJ96660 A/chicken/Jiangxi/12274/2014	.	.	.	.	.	.	.	.	.
AJJ91811 A/chicken/Jiangxi/12486/2013	.	.	.	.	.	.	.	.	.
AJJ91823 A/chicken/Jiangxi/12492/2013	.	.	.	.	.	.	.	.	.
AJJ91849 A/chicken/Jiangxi/12544/2013	.	.	.	.	.	.	.	.	.
AJJ91861 A/chicken/Jiangxi/12554/2013	.	.	.	.	.	.	.	.	.
AJJ96258 A/chicken/Jiangxi/12564/2013	.	.	.	.	.	.	.	.	.
AJJ96672 A/chicken/Jiangxi/12768/2014	.	.	.	.	.	.	.	.	.
AJJ96684 A/chicken/Jiangxi/13207/2014	.	.	.	.	.	.	.	.	.
AJJ96696 A/chicken/Jiangxi/13209/2014	.	.	.	.	.	.	.	.	.
AJJ97255 A/chicken/Jiangxi/13210/2014	.	.	.	.	.	.	.	.	.
AJJ96720 A/chicken/Jiangxi/13220/2014	.	.	.	.	.	.	.	.	.
AJJ96732 A/chicken/Jiangxi/13223/2014	.	.	.	.	.	.	.	.	.
AJJ96744 A/chicken/Jiangxi/13230/2014	.	.	.	.	.	.	.	.	.
AJJ96769 A/chicken/Jiangxi/13250/2014	.	.	.	.	.	.	.	.	.
AJJ97267 A/chicken/Jiangxi/13252/2014	.	.	.	.	.	.	.	.	.
AJJ96781 A/chicken/Jiangxi/13255/2014	.	.	.	.	.	.	.	.	.
AJJ96793 A/chicken/Jiangxi/13268/2014	.	.	.	.	.	.	.	.	.
AJJ97279 A/chicken/Jiangxi/13269/2014	.	.	.	.	.	.	.	.	.
AJJ96877 A/chicken/Jiangxi/13491/2014	.	.	.	.	.	.	.	.	.
AJJ97291 A/chicken/Jiangxi/13493/2014	.	.	.	.	.	.	.	.	.
AJJ96889 A/chicken/Jiangxi/13496/2014	.	.	.	.	.	.	.	.	.
AJJ96901 A/chicken/Jiangxi/13502/2014	.	.	.	.	.	.	.	.	.
AJJ97319 A/chicken/Jiangxi/13507/2014	.	.	.	.	.	.	.	.	.
AJJ96913 A/chicken/Jiangxi/13510/2014	.	.	.	.	.	.	.	.	.
AJJ97331 A/chicken/Jiangxi/13512/2014	.	.	.	.	.	.	.	.	.
AJJ96925 A/chicken/Jiangxi/13513/2014	.	.	.	.	.	.	.	.	.
AJJ96937 A/chicken/Jiangxi/13518/2014	.	.	.	.	.	.	.	.	.
AJJ96949 A/chicken/Jiangxi/13519/2014	.	.	.	.	.	.	.	.	.
AJJ97373 A/chicken/Jiangxi/13521/2014	.	.	.	.	.	.	.	.	.
AJJ97385 A/chicken/Jiangxi/13524/2014	.	.	.	.	.	.	.	.	.
AJJ97443 A/chicken/Jiangxi/13530/2014	.	.	.	.	.	.	.	.	.
AJJ97455 A/chicken/Jiangxi/13536/2014	.	.	.	.	.	.	.	.	.
AJJ97467 A/chicken/Jiangxi/13537/2014	.	.	.	.	.	.	.	.	.
AJJ96978 A/chicken/Jiangxi/13538/2014	.	.	.	.	.	.	.	.	.
AJJ97493 A/chicken/Jiangxi/13543/2014	.	.	.	.	.	.	.	.	.
AJJ97505 A/chicken/Jiangxi/13544/2014	.	.	.	.	.	.	.	.	.
AJJ96990 A/chicken/Jiangxi/13546/2014	.	.	.	.	.	.	.	.	.
AJJ97517 A/chicken/Jiangxi/13548/2014	.	.	.	.	.	.	.	.	.
AJJ97529 A/chicken/Jiangxi/13551/2014	.	.	.	.	.	.	.	.	.
AJJ97002 A/chicken/Jiangxi/13553/2014	.	.	.	.	.	.	.	.	.
AJJ97558 A/chicken/Jiangxi/13556/2014	.	.	.	.	.	.	.	.	.
AJJ97570 A/chicken/Jiangxi/13564/2014	.	.	.	.	.	.	.	.	.
AJJ97582 A/chicken/Jiangxi/14023/2014	.	.	.	.	.	.	.	.	.
AJJ97594 A/chicken/Jiangxi/14033/2014	.	.	.	.	.	.	.	.	.
AJJ97606 A/chicken/Jiangxi/14479/2014	.	.	.	.	.	.	.	.	.
AJJ97618 A/chicken/Jiangxi/14482/2014	.	.	.	.	.	.	.	.	.
AJJ97673 A/chicken/Jiangxi/14513/2014	.	.	.	.	.	.	.	.	.
AJJ97685 A/chicken/Jiangxi/14515/2014	.	.	.	.	.	.	.	.	.
AJJ97697 A/chicken/Jiangxi/14517/2014	.	.	.	.	.	.	.	.	.
AJJ97709 A/chicken/Jiangxi/14518/2014	.	.	.	.	.	.	.	.	.
AJJ97721 A/chicken/Jiangxi/14530/2014	.	.	.	.	.	.	.	.	.
AJJ97745 A/chicken/Jiangxi/14554/2014	.	.	.	.	.	.	.	.	.
AJJ97841 A/chicken/Jiangxi/15044/2014	.	.	.	.	.	.	.	.	.
AJJ97899 A/chicken/Jiangxi/15524/2014	.	.	.	.	.	.	.	.	.
AJJ98081 A/chicken/Jiangxi/18008/2014	.	.	.	.	.	.	.	.	.
AJJ98120 A/chicken/Jiangxi/18449/2014	.	.	.	.	.	.	.	.	.
AJJ98227 A/chicken/Jiangxi/18482/2014	.	.	.	.	.	.	.	.	.
AJJ98275 A/chicken/Jiangxi/18487/2014	.	.	.	.	.	.	.	.	.
AJJ98346 A/chicken/Jiangxi/18513/2014	.	.	.	.	.	.	.	.	.
AJJ98358 A/chicken/Jiangxi/18515/2014	.	.	.	.	.	.	.	.	.
AJJ97039 A/chicken/Jiangxi/9497/2014	.	.	.	.	.	.	.	.	.
AJJ93003 A/chicken/Jiangxi/9508/2014	.	.	.	.	.	.	.	.	.
AJJ96817 A/chicken/Jiangxi/9513/2014	.	.	.	.	.	.	.	.	.
AJJ97051 A/chicken/Jiangxi/9530/2014	.	.	.	.	.	.	.	.	.
AJJ93015 A/chicken/Jiangxi/9534/2014	.	.	.	.	.	.	.	.	.
AJJ93027 A/chicken/Jiangxi/9558/2014	.	.	.	.	.	.	.	.	.
AGR49399 A/chicken/Jiangxi/SD001/2013	.	.	.	.	.	.	.	.	.
AJJ91627 A/chicken/Jiaxing/4490/2013	.	.	.	.	.	.	.	.	.
AGQ81043 A/chicken/Rizhao/515/2013	.	.	.	.	.	.	.	.	.
AGQ81059 A/chicken/Rizhao/713/2013	.	.	.	.	.	.	.	.	.
AGQ81060 A/chicken/Rizhao/715/2013	.	.	.	.	.	.	.	.	.
AGR33894 A/chicken/Rizhao/719b/2013	.	.	.	.	.	.	.	.	.
AGQ81061 A/chicken/Rizhao/865/2013	.	.	.	.	.	.	.	.	.
AGQ81044 A/chicken/Rizhao/867/2013	.	.	.	.	.	.	.	.	.
AGQ81045 A/chicken/Rizhao/871/2013	.	.	.	.	.	.	.	.	.
AGQ81046 A/chicken/Rizhao/875/2013	.	.	.	.	.	.	.	.	.
AGU70015 A/chicken/Shanghai/017/2013	.	.	.	.	.	.	.	.	.
AGU70003 A/chicken/Shanghai/019/2013	.	.	.	.	.	.	.	.	.

AHN96472 A/chicken/Shanghai/PD-CN-02/2014
AGR49411 A/chicken/Shanghai/S1053/2013
AGR49423 A/chicken/Shanghai/S1055/2013
AGR49435 A/chicken/Shanghai/S1076/2013
AGR49447 A/chicken/Shanghai/S1077/2013
AGR49459 A/chicken/Shanghai/S1078/2013
AGR49471 A/chicken/Shanghai/S1079/2013
AGR49483 A/chicken/Shanghai/S1080/2013
AGR49495 A/chicken/Shanghai/S1358/2013
AGR49506 A/chicken/Shanghai/S1410/2013
AGR49518 A/chicken/Shanghai/S1413/2013
AJS16524 A/chicken/Shanghai/S3084/2014
AJS16529 A/chicken/Shanghai/S3090/2014
AJJ95488 A/chicken/Shantou/1550/2014
AJJ95500 A/chicken/Shantou/1552/2014
AJJ95512 A/chicken/Shantou/1554/2014
AJJ95524 A/chicken/Shantou/1556/2014
AJJ97757 A/chicken/Shantou/2537/2014
AJJ97769 A/chicken/Shantou/2538/2014
AJJ97781 A/chicken/Shantou/2539/2014
AJJ97793 A/chicken/Shantou/2546/2014
AJJ97805 A/chicken/Shantou/2550/2014
AJJ97817 A/chicken/Shantou/2556/2014
AJJ97829 A/chicken/Shantou/2562/2014
AJJ97961 A/chicken/Shantou/3057/2014
AJJ97973 A/chicken/Shantou/4325/2014
AJJ97998 A/chicken/Shantou/4816/2014
AJJ98010 A/chicken/Shantou/4824/2014
AJJ98022 A/chicken/Shantou/4832/2014
AJJ98034 A/chicken/Shantou/4833/2014
AJJ91314 A/chicken/Shaoxing/2417/2013
AJJ91653 A/chicken/Shaoxing/5086/2013
AJJ91665 A/chicken/Shaoxing/5087/2013
AJJ91689 A/chicken/Shaoxing/5136/2013
AJJ91701 A/chicken/Shaoxing/5146/2013
AJJ91713 A/chicken/Shaoxing/5186/2013
AJJ91725 A/chicken/Shaoxing/5201/2013
AJJ91737 A/chicken/Shaoxing/5224/2013
AJJ91749 A/chicken/Shaoxing/5227/2013
AJJ91787 A/chicken/Shaoxing/5479/2013
AJJ95464 A/chicken/Shenzhen/138/2014
AJJ90576 A/chicken/Shenzhen/1665/2013
AJJ90588 A/chicken/Shenzhen/2110/2013
AJJ90637 A/chicken/Shenzhen/2201/2013
AJJ90649 A/chicken/Shenzhen/2293/2013
AJJ91011 A/chicken/Shenzhen/3733/2013
AJJ91023 A/chicken/Shenzhen/3734/2013
AJJ91035 A/chicken/Shenzhen/3780/2013
AJJ90478 A/chicken/Shenzhen/727/2013
AJJ90490 A/chicken/Shenzhen/742/2013
AJJ90502 A/chicken/Shenzhen/747/2013
AJJ90514 A/chicken/Shenzhen/749/2013
AJJ92005 A/chicken/Shenzhen/801/2013
AJJ90526 A/chicken/Shenzhen/898/2013
AIU47013 A/chicken/Suzhou/040201H/2013
ARB51605 A/chicken/Tennessee/17-007147-2/2017
ARB51617 A/chicken/Tennessee/17-007431-3/2017
ARB51641 A/chicken/Tennessee/17-008279-4/2017
AJJ98510 A/chicken/Zhangzhou/8585/2014
AJJ98522 A/chicken/Zhangzhou/8829/2014
AGN69410 A/chicken/Zhejiang/C481/2013
AGN69400 A/chicken/Zhejiang/C483/2013
AGJ72861 A/chicken/Zhejiang/DTID-ZJU01/2013
AIU46619 A/chicken/Zhejiang/DTID-ZJU06/2013
AGR49530 A/chicken/Zhejiang/SD007/2013
AGR49542 A/chicken/Zhejiang/SD019/2013
AGR49554 A/chicken/Zhejiang/SD033/2013
AGR49566 A/duck/Anhui/SC702/2013
AIU47001 A/duck/Sunan/040802G/2013
AJK00354 A/duck/Zhejiang/LS02/2014
AGR49578 A/duck/Zhejiang/SC410/2013
AJK00245 A/goose/Jiangsu/1027/2013 2013/04/06 HA
ARB51629 A/guinea fowl/Alabama/17-008272-2/2017
AGR49722 A/homing pigeon/Jiangsu/SD184/2013
AGR49734 A/pigeon/Shanghai/S1069/2013
AGR49746 A/pigeon/Shanghai/S1421/2013
AGR49758 A/pigeon/Shanghai/S1423/2013
AIU46989 A/pigeon/Wuxi/0405007G/2013
AGN69430 A/pigeon/Zhejiang/P1/2013
AGN69420 A/pigeon/Zhejiang/P2/2013
AJJ94194 A/silkie chicken/Dongguan/1264/2014
AJJ94206 A/silkie chicken/Dongguan/1268/2014
AJJ95135 A/silkie chicken/Dongguan/1271/2014
AJJ94218 A/silkie chicken/Dongguan/1274/2014
AJJ94305 A/silkie chicken/Dongguan/1448/2014
AJJ94332 A/silkie chicken/Dongguan/1450/2014
AJJ94344 A/silkie chicken/Dongguan/1451/2014
AJJ94432 A/silkie chicken/Dongguan/1516/2014
AJJ95584 A/silkie chicken/Dongguan/1519/2014
AJJ93845 A/silkie chicken/Dongguan/157/2014
AJJ95358 A/silkie chicken/Dongguan/1641/2014
AJJ90673 A/silkie chicken/Dongguan/3049/2013
AJJ90733 A/silkie chicken/Dongguan/3166/2013
AJJ90783 A/silkie chicken/Dongguan/3275/2013
AJJ90795 A/silkie chicken/Dongguan/3281/2013
AJJ90807 A/silkie chicken/Dongguan/3284/2013
AJJ90891 A/silkie chicken/Dongguan/3520/2013
AJJ90903 A/silkie chicken/Dongguan/3522/2013
AJJ90915 A/silkie chicken/Dongguan/3525/2013
AJJ90927 A/silkie chicken/Dongguan/3526/2013
AJJ90939 A/silkie chicken/Dongguan/3528/2013
AJJ90987 A/silkie chicken/Dongguan/3605/2013
AJJ90999 A/silkie chicken/Dongguan/3606/2013
AJJ91131 A/silkie chicken/Dongguan/3980/2013
AJJ91143 A/silkie chicken/Dongguan/3990/2013
AJJ91227 A/silkie chicken/Dongguan/4126/2013
AJJ91239 A/silkie chicken/Dongguan/4127/2013
AJJ91264 A/silkie chicken/Dongguan/4129/2013
AJJ95440 A/silkie chicken/Dongguan/523/2014
AJJ93967 A/silkie chicken/Dongguan/635/2014
AJJ93979 A/silkie chicken/Dongguan/656/2014
AJJ95548 A/silkie chicken/Dongguan/953/2014
AJJ94081 A/silkie chicken/Dongguan/963/2014
AJJ94959 A/silkie chicken/Dongguan/967/2014
AJJ94986 A/silkie chicken/Dongguan/969/2014
AJJ94998 A/silkie chicken/Dongguan/979/2014
AJJ95010 A/silkie chicken/Dongguan/981/2014
AJJ95022 A/silkie chicken/Dongguan/986/2014
AJJ94110 A/silkie chicken/Dongguan/988/2014
AJJ94122 A/silkie chicken/Dongguan/991/2014
AJJ95560 A/silkie chicken/Dongguan/997/2014
AJJ91578 A/silkie chicken/Huzhou/4213/2013
AJJ92967 A/silkie chicken/Jiangxi/9469/2014
AJJ92979 A/silkie chicken/Jiangxi/9472/2014
AJJ92991 A/silkie chicken/Jiangxi/9476/2014
AJJ95476 A/silkie chicken/Shantou/1406/2014
AJJ97925 A/silkie chicken/Shantou/2050/2014
AJJ97937 A/silkie chicken/Shantou/2054/2014
AJJ97949 A/silkie chicken/Shantou/2056/2014
AJJ91677 A/silkie chicken/Shaoxing/5130/2013
AJJ91761 A/silkie chicken/Shaoxing/5235/2013
AJJ90613 A/silkie chicken/Shenzhen/2134/2013
AJJ90625 A/silkie chicken/Shenzhen/2139/2013
AJJ91047 A/silkie chicken/Shenzhen/3781/2013
AJJ91059 A/silkie chicken/Shenzhen/3782/2013
AJJ90538 A/silkie chicken/Shenzhen/918/2013
AJJ90550 A/silkie chicken/Shenzhen/919/2013
AHL24617 A/tree sparrow/Shanghai/01/2013
AGW82588 A/tree sparrow/Shanghai/01/2013
AGR49770 A/wild pigeon/Jiangsu/SD001/2013
EPI439507 A/Anhui/1/2013 H7N9 HA
EPI439486 A/Shanghai/1/2013 H7N9 HA
JQ906576 A/duck/Zhejiang/12/2011 H7N3 HA

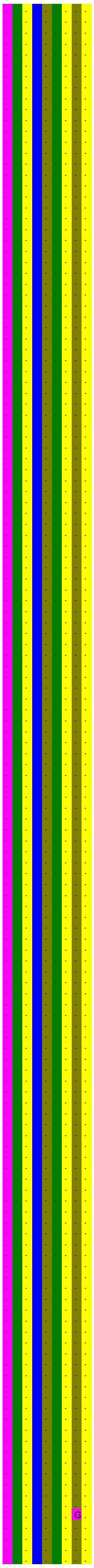

Human viruses

AHZ60096 A/Anhui/1-DEWH730/2013
AHZ39686 A/Anhui/DEWH72-01/2013
AHZ39698 A/Anhui/DEWH72-02/2013

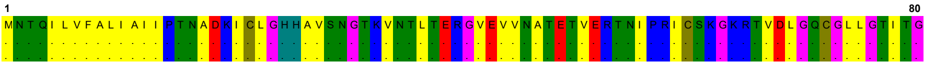

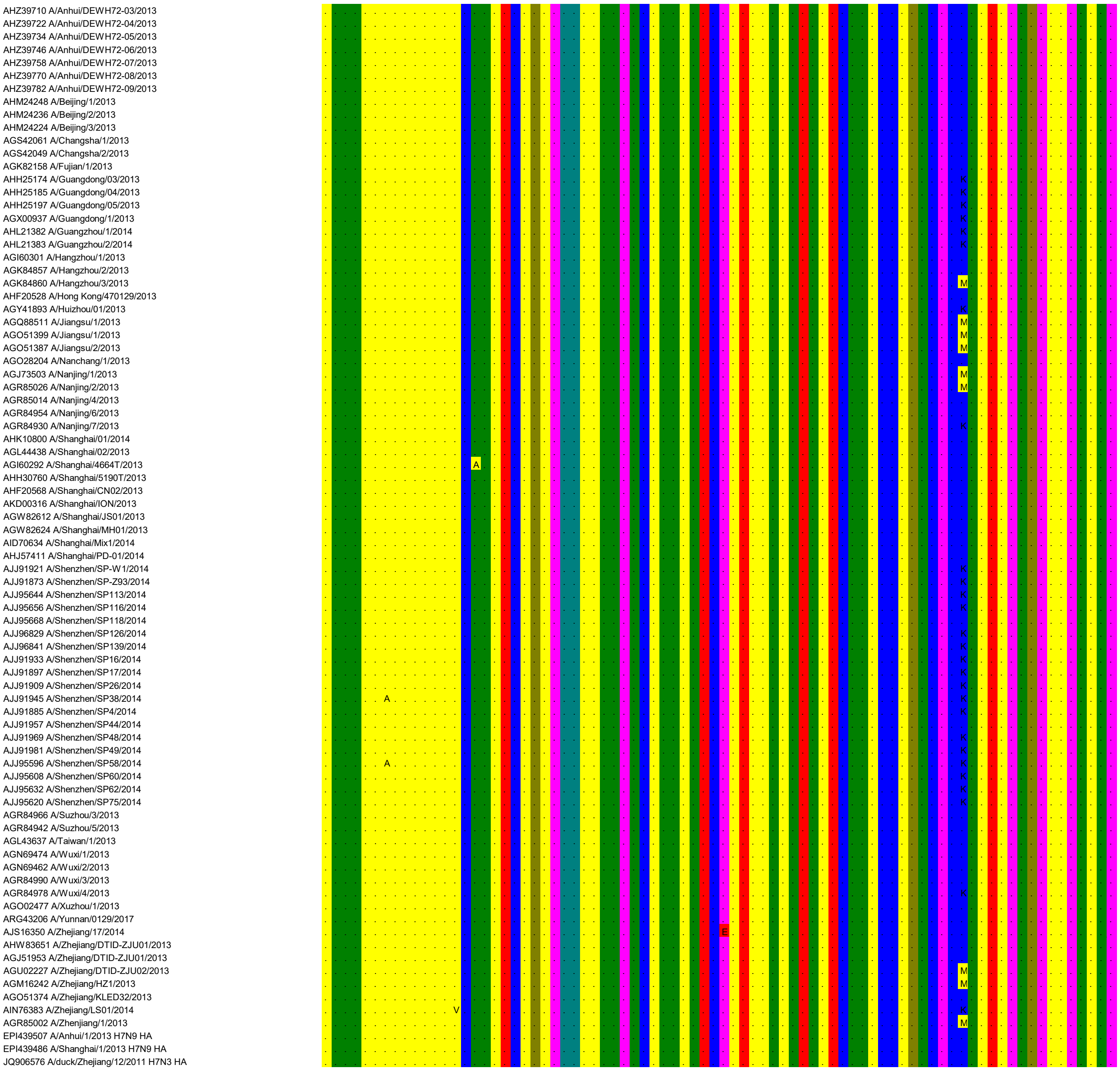
AHZ39710 A/Anhui/DEWH72-03/2013
AHZ39722 A/Anhui/DEWH72-04/2013
AHZ39734 A/Anhui/DEWH72-05/2013
AHZ39746 A/Anhui/DEWH72-06/2013
AHZ39758 A/Anhui/DEWH72-07/2013
AHZ39770 A/Anhui/DEWH72-08/2013
AHZ39782 A/Anhui/DEWH72-09/2013
AHM24248 A/Beijing/1/2013
AHM24236 A/Beijing/2/2013
AHM24224 A/Beijing/3/2013
AGS42061 A/Changsha/1/2013
AGS42049 A/Changsha/2/2013
AGK82158 A/Fujian/1/2013
AHH25174 A/Guangdong/03/2013
AHH25185 A/Guangdong/04/2013
AHH25197 A/Guangdong/05/2013
AGX00937 A/Guangdong/1/2013
AHL21382 A/Guangzhou/1/2014
AHL21383 A/Guangzhou/2/2014
AGI60301 A/Hangzhou/1/2013
AGK84857 A/Hangzhou/2/2013
AGK84860 A/Hangzhou/3/2013
AHF20528 A/Hong Kong/470129/2013
AGY41893 A/Huizhou/01/2013
AGQ88511 A/Jiangsu/1/2013
AGO51399 A/Jiangsu/1/2013
AGO51387 A/Jiangsu/2/2013
AGO28204 A/Nanchang/1/2013
AGJ73503 A/Nanjing/1/2013
AGR85026 A/Nanjing/2/2013
AGR85014 A/Nanjing/4/2013
AGR84954 A/Nanjing/6/2013
AGR84930 A/Nanjing/7/2013
AHK10800 A/Shanghai/01/2014
AGL44438 A/Shanghai/02/2013
AGI60292 A/Shanghai/4664T/2013
AHH30760 A/Shanghai/5190T/2013
AHF20568 A/Shanghai/CN02/2013
AKD00316 A/Shanghai/ION/2013
AGW82612 A/Shanghai/JS01/2013
AGW82624 A/Shanghai/MH01/2013
AID70634 A/Shanghai/Mix1/2014
AHJ57411 A/Shanghai/PD-01/2014
AJJ91921 A/Shenzhen/SP-W1/2014
AJJ91873 A/Shenzhen/SP-Z93/2014
AJJ95644 A/Shenzhen/SP113/2014
AJJ95656 A/Shenzhen/SP116/2014
AJJ95668 A/Shenzhen/SP118/2014
AJJ96829 A/Shenzhen/SP126/2014
AJJ96841 A/Shenzhen/SP139/2014
AJJ91933 A/Shenzhen/SP16/2014
AJJ91897 A/Shenzhen/SP17/2014
AJJ91909 A/Shenzhen/SP26/2014
AJJ91945 A/Shenzhen/SP38/2014
AJJ91885 A/Shenzhen/SP4/2014
AJJ91957 A/Shenzhen/SP44/2014
AJJ91969 A/Shenzhen/SP48/2014
AJJ91981 A/Shenzhen/SP49/2014
AJJ95596 A/Shenzhen/SP58/2014
AJJ95608 A/Shenzhen/SP60/2014
AJJ95632 A/Shenzhen/SP62/2014
AJJ95620 A/Shenzhen/SP75/2014
AGR84966 A/Suzhou/3/2013
AGR84942 A/Suzhou/5/2013
AGL43637 A/Taiwan/1/2013
AGN69474 A/Wuxi/1/2013
AGN69462 A/Wuxi/2/2013
AGR84990 A/Wuxi/3/2013
AGR84978 A/Wuxi/4/2013
AGO02477 A/Xuzhou/1/2013
ARG43206 A/Yunnan/0129/2017
AJS16350 A/Zhejiang/17/2014
AHW83651 A/Zhejiang/DTID-ZJU01/2013
AGJ51953 A/Zhejiang/DTID-ZJU01/2013
AGU02227 A/Zhejiang/DTID-ZJU02/2013
AGM16242 A/Zhejiang/HZ1/2013
AGO51374 A/Zhejiang/KLED32/2013
AIN76383 A/Zhejiang/LS01/2014
AGR85002 A/Zhenjiang/1/2013
EPI439507 A/Anhui/1/2013 H7N9 HA
EPI439486 A/Shanghai/1/2013 H7N9 HA
JQ906576 A/duck/Zhejiang/12/2011 H7N3 HA

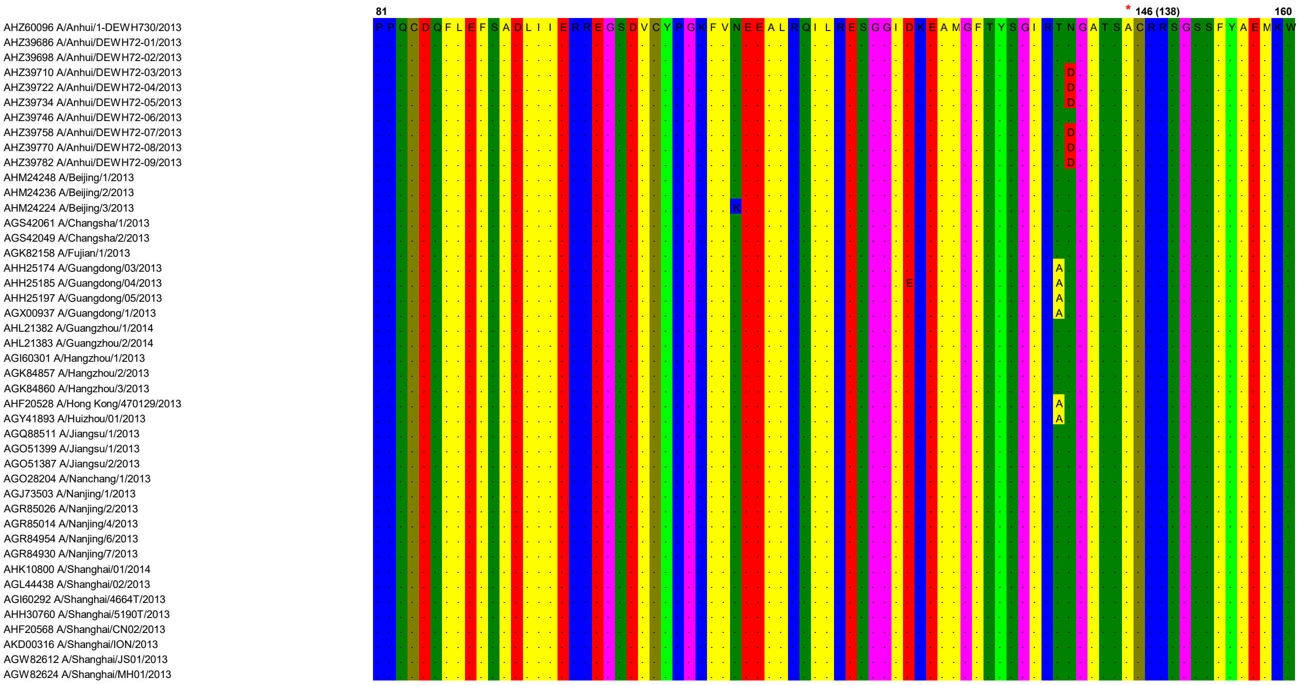
81
146 (138)
160
AHZ60096 A/Anhui/1-DEWH730/2013
AHZ39686 A/Anhui/DEWH72-01/2013
AHZ39698 A/Anhui/DEWH72-02/2013
AHZ39710 A/Anhui/DEWH72-03/2013
AHZ39722 A/Anhui/DEWH72-04/2013
AHZ39734 A/Anhui/DEWH72-05/2013
AHZ39746 A/Anhui/DEWH72-06/2013
AHZ39758 A/Anhui/DEWH72-07/2013
AHZ39770 A/Anhui/DEWH72-08/2013
AHZ39782 A/Anhui/DEWH72-09/2013
AHM24248 A/Beijing/1/2013
AHM24236 A/Beijing/2/2013
AHM24224 A/Beijing/3/2013
AGS42061 A/Changsha/1/2013
AGS42049 A/Changsha/2/2013
AGK82158 A/Fujian/1/2013
AHH25174 A/Guangdong/03/2013
AHH25185 A/Guangdong/04/2013
AHH25197 A/Guangdong/05/2013
AGX00937 A/Guangdong/1/2013
AHL21382 A/Guangzhou/1/2014
AHL21383 A/Guangzhou/2/2014
AGI60301 A/Hangzhou/1/2013
AGK84857 A/Hangzhou/2/2013
AGK84860 A/Hangzhou/3/2013
AHF20528 A/Hong Kong/470129/2013
AGY41893 A/Huizhou/01/2013
AGQ88511 A/Jiangsu/1/2013
AGO51399 A/Jiangsu/1/2013
AGO51387 A/Jiangsu/2/2013
AGO28204 A/Nanchang/1/2013
AGJ73503 A/Nanjing/1/2013
AGR85026 A/Nanjing/2/2013
AGR85014 A/Nanjing/4/2013
AGR84954 A/Nanjing/6/2013
AGR84930 A/Nanjing/7/2013
AHK10800 A/Shanghai/01/2014
AGL44438 A/Shanghai/02/2013
AGI60292 A/Shanghai/4664T/2013
AHH30760 A/Shanghai/5190T/2013
AHF20568 A/Shanghai/CN02/2013
AKD00316 A/Shanghai/ION/2013
AGW82612 A/Shanghai/JS01/2013
AGW82624 A/Shanghai/MH01/2013

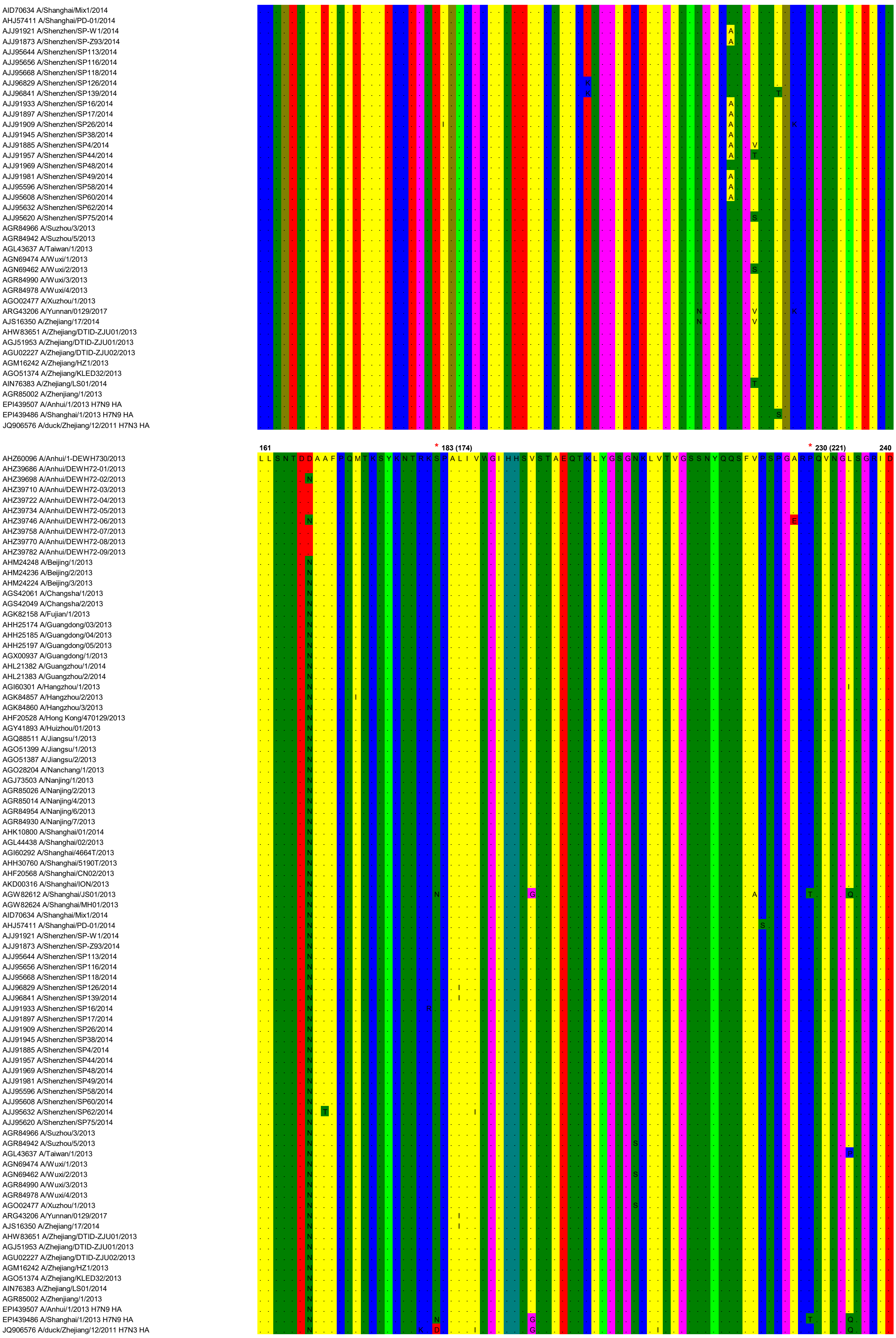
AID70634 A/Shanghai/Mix1/2014
AHJ57411 A/Shanghai/PD-01/2014
AJJ91921 A/Shenzhen/SP-W1/2014
AJJ91873 A/Shenzhen/SP-Z93/2014
AJJ95644 A/Shenzhen/SP113/2014
AJJ95656 A/Shenzhen/SP116/2014
AJJ95668 A/Shenzhen/SP118/2014
AJJ96829 A/Shenzhen/SP126/2014
AJJ96841 A/Shenzhen/SP139/2014
AJJ91933 A/Shenzhen/SP16/2014
AJJ91897 A/Shenzhen/SP17/2014
AJJ91909 A/Shenzhen/SP26/2014
AJJ91945 A/Shenzhen/SP38/2014
AJJ91885 A/Shenzhen/SP4/2014
AJJ91957 A/Shenzhen/SP44/2014
AJJ91969 A/Shenzhen/SP48/2014
AJJ91981 A/Shenzhen/SP49/2014
AJJ95596 A/Shenzhen/SP58/2014
AJJ95608 A/Shenzhen/SP60/2014
AJJ95632 A/Shenzhen/SP62/2014
AJJ95620 A/Shenzhen/SP75/2014
AGR84966 A/Suzhou/3/2013
AGR84942 A/Suzhou/5/2013
AGL43637 A/Taiwan/1/2013
AGN69474 A/Wuxi/1/2013
AGN69462 A/Wuxi/2/2013
AGR84990 A/Wuxi/3/2013
AGR84978 A/Wuxi/4/2013
AGO02477 A/Xuzhou/1/2013
ARG43206 A/Yunnan/0129/2017
AJS16350 A/Zhejiang/17/2014
AHW83651 A/Zhejiang/DTID-ZJU01/2013
AGJ51953 A/Zhejiang/DTID-ZJU01/2013
AGU02227 A/Zhejiang/DTID-ZJU02/2013
AGM16242 A/Zhejiang/HZ1/2013
AGO51374 A/Zhejiang/KLED32/2013
AIN76383 A/Zhejiang/LS01/2014
AGR85002 A/Zhenjiang/1/2013
EPI439507 A/Anhui/1/2013 H7N9 HA
EPI439486 A/Shanghai/1/2013 H7N9 HA
JQ906576 A/duck/Zhejiang/12/2011 H7N3 HA
161
* 183 (174)
* 230 (221)
240
AHZ60096 A/Anhui/1-DEWH730/2013
AHZ39686 A/Anhui/DEWH72-01/2013
AHZ39698 A/Anhui/DEWH72-02/2013
AHZ39710 A/Anhui/DEWH72-03/2013
AHZ39722 A/Anhui/DEWH72-04/2013
AHZ39734 A/Anhui/DEWH72-05/2013
AHZ39746 A/Anhui/DEWH72-06/2013
AHZ39758 A/Anhui/DEWH72-07/2013
AHZ39770 A/Anhui/DEWH72-08/2013
AHZ39782 A/Anhui/DEWH72-09/2013
AHM24248 A/Beijing/1/2013
AHM24236 A/Beijing/2/2013
AHM24224 A/Beijing/3/2013
AGS42061 A/Changsha/1/2013
AGS42049 A/Changsha/2/2013
AGK82158 A/Fujian/1/2013
AHH25174 A/Guangdong/03/2013
AHH25185 A/Guangdong/04/2013
AHH25197 A/Guangdong/05/2013
AGX00937 A/Guangdong/1/2013
AHL21382 A/Guangzhou/1/2014
AHL21383 A/Guangzhou/2/2014
AGI60301 A/Hangzhou/1/2013
AGK84857 A/Hangzhou/2/2013
AGK84860 A/Hangzhou/3/2013
AHF20528 A/Hong Kong/470129/2013
AGY41893 A/Huizhou/01/2013
AGQ88511 A/Jiangsu/1/2013
AGO51399 A/Jiangsu/1/2013
AGO51387 A/Jiangsu/2/2013
AGO28204 A/Nanchang/1/2013
AGJ73503 A/Nanjing/1/2013
AGR85026 A/Nanjing/2/2013
AGR85014 A/Nanjing/4/2013
AGR84954 A/Nanjing/6/2013
AGR84930 A/Nanjing/7/2013
AHK10800 A/Shanghai/01/2014
AGL44438 A/Shanghai/02/2013
AGI60292 A/Shanghai/4664T/2013
AHH30760 A/Shanghai/5190T/2013
AHF20568 A/Shanghai/CN02/2013
AKD00316 A/Shanghai/ION/2013
AGW82612 A/Shanghai/JS01/2013
AGW82624 A/Shanghai/MH01/2013
AID70634 A/Shanghai/Mix1/2014
AHJ57411 A/Shanghai/PD-01/2014
AJJ91921 A/Shenzhen/SP-W1/2014
AJJ91873 A/Shenzhen/SP-Z93/2014
AJJ95644 A/Shenzhen/SP113/2014
AJJ95656 A/Shenzhen/SP116/2014
AJJ95668 A/Shenzhen/SP118/2014
AJJ96829 A/Shenzhen/SP126/2014
AJJ96841 A/Shenzhen/SP139/2014
AJJ91933 A/Shenzhen/SP16/2014
AJJ91897 A/Shenzhen/SP17/2014
AJJ91909 A/Shenzhen/SP26/2014
AJJ91945 A/Shenzhen/SP38/2014
AJJ91885 A/Shenzhen/SP4/2014
AJJ91957 A/Shenzhen/SP44/2014
AJJ91969 A/Shenzhen/SP48/2014
AJJ91981 A/Shenzhen/SP49/2014
AJJ95596 A/Shenzhen/SP58/2014
AJJ95608 A/Shenzhen/SP60/2014
AJJ95632 A/Shenzhen/SP62/2014
AJJ95620 A/Shenzhen/SP75/2014
AGR84966 A/Suzhou/3/2013
AGR84942 A/Suzhou/5/2013
AGL43637 A/Taiwan/1/2013
AGN69474 A/Wuxi/1/2013
AGN69462 A/Wuxi/2/2013
AGR84990 A/Wuxi/3/2013
AGR84978 A/Wuxi/4/2013
AGO02477 A/Xuzhou/1/2013
ARG43206 A/Yunnan/0129/2017
AJS16350 A/Zhejiang/17/2014
AHW83651 A/Zhejiang/DTID-ZJU01/2013
AGJ51953 A/Zhejiang/DTID-ZJU01/2013
AGU02227 A/Zhejiang/DTID-ZJU02/2013
AGM16242 A/Zhejiang/HZ1/2013
AGO51374 A/Zhejiang/KLED32/2013
AIN76383 A/Zhejiang/LS01/2014
AGR85002 A/Zhenjiang/1/2013
EPI439507 A/Anhui/1/2013 H7N9 HA
EPI439486 A/Shanghai/1/2013 H7N9 HA
JQ906576 A/duck/Zhejiang/12/2011 H7N3 HA

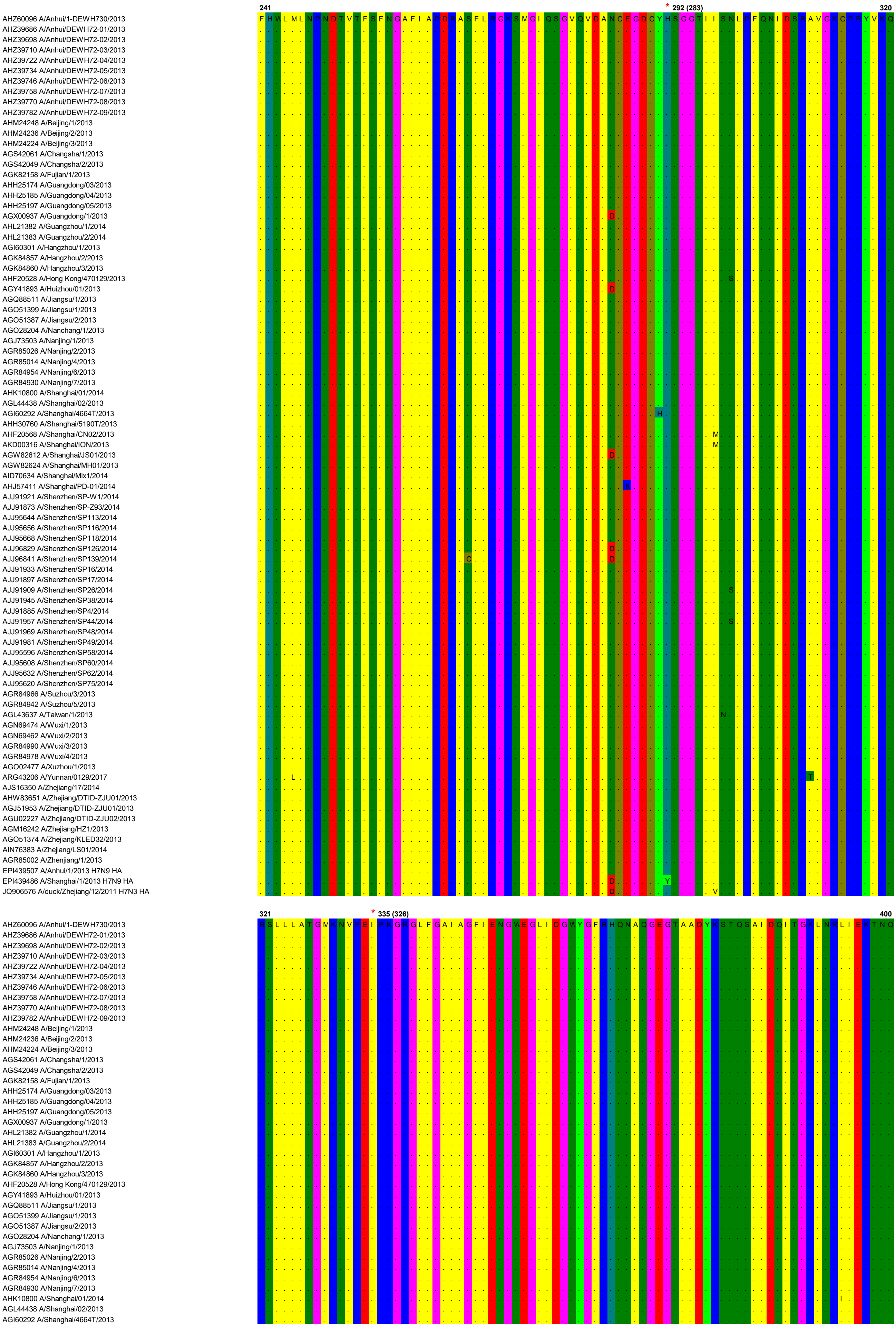

241
* 292 (283)
320
AHZ60096 A/Anhui/1-DEWH730/2013
AHZ39686 A/Anhui/DEWH72-01/2013
AHZ39698 A/Anhui/DEWH72-02/2013
AHZ39710 A/Anhui/DEWH72-03/2013
AHZ39722 A/Anhui/DEWH72-04/2013
AHZ39734 A/Anhui/DEWH72-05/2013
AHZ39746 A/Anhui/DEWH72-06/2013
AHZ39758 A/Anhui/DEWH72-07/2013
AHZ39770 A/Anhui/DEWH72-08/2013
AHZ39782 A/Anhui/DEWH72-09/2013
AHM24248 A/Beijing/1/2013
AHM24236 A/Beijing/2/2013
AHM24224 A/Beijing/3/2013
AGS42061 A/Changsha/1/2013
AGS42049 A/Changsha/2/2013
AGK82158 A/Fujian/1/2013
AHH25174 A/Guangdong/03/2013
AHH25185 A/Guangdong/04/2013
AHH25197 A/Guangdong/05/2013
AGX00937 A/Guangdong/1/2013
AHL21382 A/Guangzhou/1/2014
AHL21383 A/Guangzhou/2/2014
AGI60301 A/Hangzhou/1/2013
AGK84857 A/Hangzhou/2/2013
AGK84860 A/Hangzhou/3/2013
AHF20528 A/Hong Kong/470129/2013
AGY41893 A/Huizhou/01/2013
AGQ88511 A/Jiangsu/1/2013
AGO51399 A/Jiangsu/1/2013
AGO51387 A/Jiangsu/2/2013
AGO28204 A/Nanchang/1/2013
AGJ73503 A/Nanjing/1/2013
AGR85026 A/Nanjing/2/2013
AGR85014 A/Nanjing/4/2013
AGR84954 A/Nanjing/6/2013
AGR84930 A/Nanjing/7/2013
AHK10800 A/Shanghai/01/2014
AGL44438 A/Shanghai/02/2013
AGI60292 A/Shanghai/4664T/2013
AHH30760 A/Shanghai/5190T/2013
AHF20568 A/Shanghai/CN02/2013
AKD00316 A/Shanghai/ION/2013
AGW82612 A/Shanghai/JS01/2013
AGW82624 A/Shanghai/MH01/2013
AID70634 A/Shanghai/Mix1/2014
AHJ57411 A/Shanghai/PD-01/2014
AJJ91921 A/Shenzhen/SP-W1/2014
AJJ91873 A/Shenzhen/SP-Z93/2014
AJJ95644 A/Shenzhen/SP113/2014
AJJ95656 A/Shenzhen/SP116/2014
AJJ95668 A/Shenzhen/SP118/2014
AJJ96829 A/Shenzhen/SP126/2014
AJJ96841 A/Shenzhen/SP139/2014
AJJ91933 A/Shenzhen/SP16/2014
AJJ91897 A/Shenzhen/SP17/2014
AJJ91909 A/Shenzhen/SP26/2014
AJJ91945 A/Shenzhen/SP38/2014
AJJ91885 A/Shenzhen/SP4/2014
AJJ91957 A/Shenzhen/SP44/2014
AJJ91969 A/Shenzhen/SP48/2014
AJJ91981 A/Shenzhen/SP49/2014
AJJ95596 A/Shenzhen/SP58/2014
AJJ95608 A/Shenzhen/SP60/2014
AJJ95632 A/Shenzhen/SP62/2014
AJJ95620 A/Shenzhen/SP75/2014
AGR84966 A/Suzhou/3/2013
AGR84942 A/Suzhou/5/2013
AGL43637 A/Taiwan/1/2013
AGN69474 A/Wuxi/1/2013
AGN69462 A/Wuxi/2/2013
AGR84990 A/Wuxi/3/2013
AGR84978 A/Wuxi/4/2013
AGO02477 A/Xuzhou/1/2013
ARG43206 A/Yunnan/0129/2017
AJS16350 A/Zhejiang/17/2014
AHW83651 A/Zhejiang/DTID-ZJU01/2013
AGJ51953 A/Zhejiang/DTID-ZJU01/2013
AGU02227 A/Zhejiang/DTID-ZJU02/2013
AGM16242 A/Zhejiang/HZ1/2013
AGO51374 A/Zhejiang/KLED32/2013
AIN76383 A/Zhejiang/LS01/2014
AGR85002 A/Zhenjiang/1/2013
EPI439507 A/Anhui/1/2013 H7N9 HA
EPI439486 A/Shanghai/1/2013 H7N9 HA
JQ906576 A/duck/Zhejiang/12/2011 H7N3 HA
321
* 335 (326)
400
AHZ60096 A/Anhui/1-DEWH730/2013
AHZ39686 A/Anhui/DEWH72-01/2013
AHZ39698 A/Anhui/DEWH72-02/2013
AHZ39710 A/Anhui/DEWH72-03/2013
AHZ39722 A/Anhui/DEWH72-04/2013
AHZ39734 A/Anhui/DEWH72-05/2013
AHZ39746 A/Anhui/DEWH72-06/2013
AHZ39758 A/Anhui/DEWH72-07/2013
AHZ39770 A/Anhui/DEWH72-08/2013
AHZ39782 A/Anhui/DEWH72-09/2013
AHM24248 A/Beijing/1/2013
AHM24236 A/Beijing/2/2013
AHM24224 A/Beijing/3/2013
AGS42061 A/Changsha/1/2013
AGS42049 A/Changsha/2/2013
AGK82158 A/Fujian/1/2013
AHH25174 A/Guangdong/03/2013
AHH25185 A/Guangdong/04/2013
AHH25197 A/Guangdong/05/2013
AGX00937 A/Guangdong/1/2013
AHL21382 A/Guangzhou/1/2014
AHL21383 A/Guangzhou/2/2014
AGI60301 A/Hangzhou/1/2013
AGK84857 A/Hangzhou/2/2013
AGK84860 A/Hangzhou/3/2013
AHF20528 A/Hong Kong/470129/2013
AGY41893 A/Huizhou/01/2013
AGQ88511 A/Jiangsu/1/2013
AGO51399 A/Jiangsu/1/2013
AGO51387 A/Jiangsu/2/2013
AGO28204 A/Nanchang/1/2013
AGJ73503 A/Nanjing/1/2013
AGR85026 A/Nanjing/2/2013
AGR85014 A/Nanjing/4/2013
AGR84954 A/Nanjing/6/2013
AGR84930 A/Nanjing/7/2013
AHK10800 A/Shanghai/01/2014
AGL44438 A/Shanghai/02/2013
AGI60292 A/Shanghai/4664T/2013

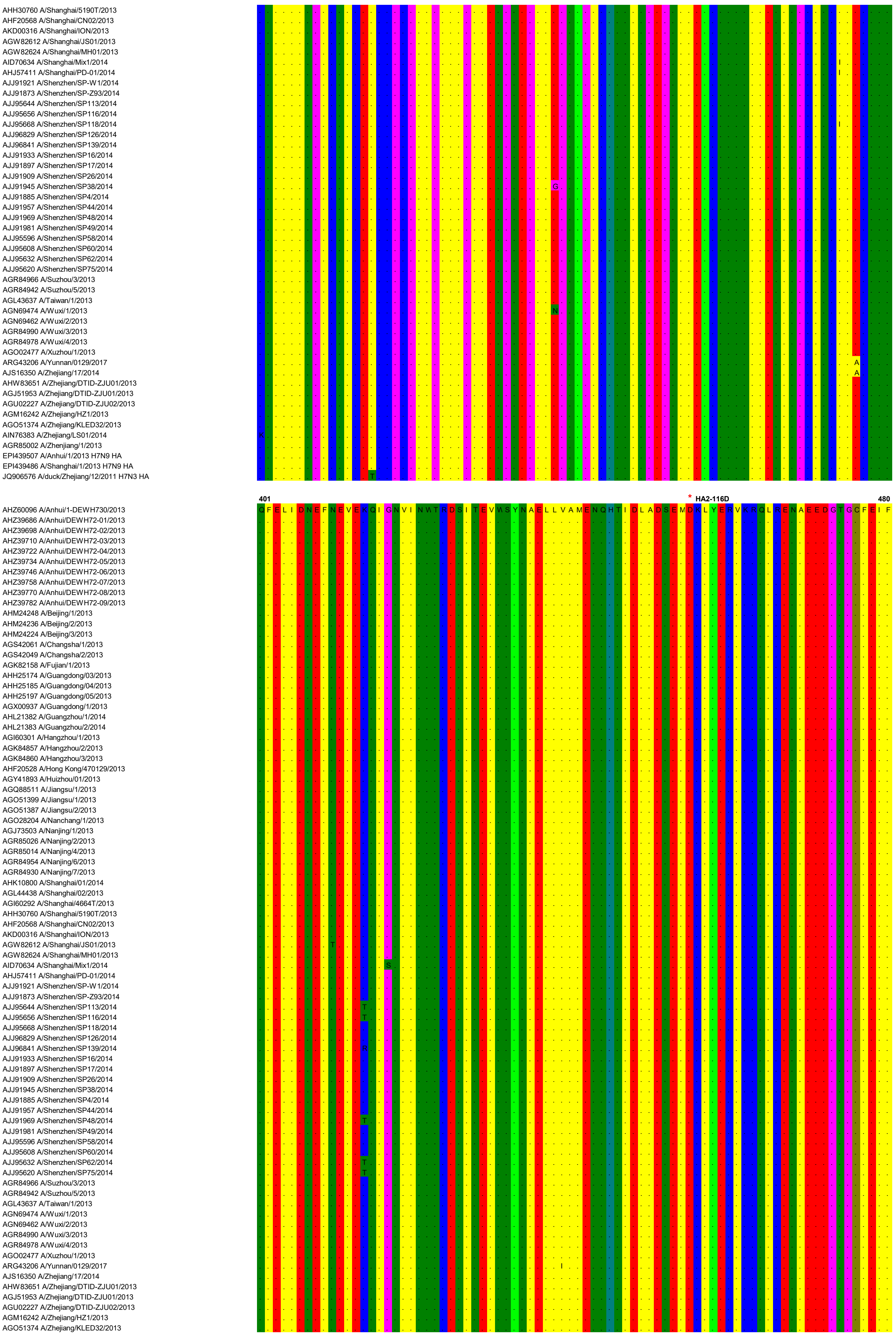
AHH30760 A/Shanghai/5190T/2013
AHF20568 A/Shanghai/CN02/2013
AKD00316 A/Shanghai/ION/2013
AGW82612 A/Shanghai/JS01/2013
AGW82624 A/Shanghai/MH01/2013
AID70634 A/Shanghai/Mix1/2014
AHJ57411 A/Shanghai/PD-01/2014
AJJ91921 A/Shenzhen/SP-W1/2014
AJJ91873 A/Shenzhen/SP-Z93/2014
AJJ95644 A/Shenzhen/SP113/2014
AJJ95656 A/Shenzhen/SP116/2014
AJJ95668 A/Shenzhen/SP118/2014
AJJ96829 A/Shenzhen/SP126/2014
AJJ96841 A/Shenzhen/SP139/2014
AJJ91933 A/Shenzhen/SP16/2014
AJJ91897 A/Shenzhen/SP17/2014
AJJ91909 A/Shenzhen/SP26/2014
AJJ91945 A/Shenzhen/SP38/2014
AJJ91885 A/Shenzhen/SP4/2014
AJJ91957 A/Shenzhen/SP44/2014
AJJ91969 A/Shenzhen/SP48/2014
AJJ91981 A/Shenzhen/SP49/2014
AJJ95596 A/Shenzhen/SP58/2014
AJJ95608 A/Shenzhen/SP60/2014
AJJ95632 A/Shenzhen/SP62/2014
AJJ95620 A/Shenzhen/SP75/2014
AGR84966 A/Suzhou/3/2013
AGR84942 A/Suzhou/5/2013
AGL43637 A/Taiwan/1/2013
AGN69474 A/Wuxi/1/2013
AGN69462 A/Wuxi/2/2013
AGR84990 A/Wuxi/3/2013
AGR84978 A/Wuxi/4/2013
AGO02477 A/Xuzhou/1/2013
ARG43206 A/Yunnan/0129/2017
AJS16350 A/Zhejiang/17/2014
AHW83651 A/Zhejiang/DTID-ZJU01/2013
AGJ51953 A/Zhejiang/DTID-ZJU01/2013
AGU02227 A/Zhejiang/DTID-ZJU02/2013
AGM16242 A/Zhejiang/HZ1/2013
AGO51374 A/Zhejiang/KLED32/2013
AIN76383 A/Zhejiang/LS01/2014
AGR85002 A/Zhenjiang/1/2013
EPI439507 A/Anhui/1/2013 H7N9 HA
EPI439486 A/Shanghai/1/2013 H7N9 HA
JQ906576 A/duck/Zhejiang/12/2011 H7N3 HA
401
* HA2-116D
480
QFELIDNEFNEVEKQIGNVINWTRDSITEVWSYNAELLVAMENQHTIDLADSEMDKLYERVKRQLRENAEEDGTGCFEIF
AHZ60096 A/Anhui/1-DEWH730/2013
AHZ39686 A/Anhui/DEWH72-01/2013
AHZ39698 A/Anhui/DEWH72-02/2013
AHZ39710 A/Anhui/DEWH72-03/2013
AHZ39722 A/Anhui/DEWH72-04/2013
AHZ39734 A/Anhui/DEWH72-05/2013
AHZ39746 A/Anhui/DEWH72-06/2013
AHZ39758 A/Anhui/DEWH72-07/2013
AHZ39770 A/Anhui/DEWH72-08/2013
AHZ39782 A/Anhui/DEWH72-09/2013
AHM24248 A/Beijing/1/2013
AHM24236 A/Beijing/2/2013
AHM24224 A/Beijing/3/2013
AGS42061 A/Changsha/1/2013
AGS42049 A/Changsha/2/2013
AGK82158 A/Fujian/1/2013
AHH25174 A/Guangdong/03/2013
AHH25185 A/Guangdong/04/2013
AHH25197 A/Guangdong/05/2013
AGX00937 A/Guangdong/1/2013
AHL21382 A/Guangzhou/1/2014
AHL21383 A/Guangzhou/2/2014
AGI60301 A/Hangzhou/1/2013
AGK84857 A/Hangzhou/2/2013
AGK84860 A/Hangzhou/3/2013
AHF20528 A/Hong Kong/470129/2013
AGY41893 A/Huizhou/01/2013
AGQ88511 A/Jiangsu/1/2013
AGO51399 A/Jiangsu/1/2013
AGO51387 A/Jiangsu/2/2013
AGO28204 A/Nanchang/1/2013
AGJ73503 A/Nanjing/1/2013
AGR85026 A/Nanjing/2/2013
AGR85014 A/Nanjing/4/2013
AGR84954 A/Nanjing/6/2013
AGR84930 A/Nanjing/7/2013
AHK10800 A/Shanghai/01/2014
AGL44438 A/Shanghai/02/2013
AGI60292 A/Shanghai/4664T/2013
AHH30760 A/Shanghai/5190T/2013
AHF20568 A/Shanghai/CN02/2013
AKD00316 A/Shanghai/ION/2013
AGW82612 A/Shanghai/JS01/2013
AGW82624 A/Shanghai/MH01/2013
AID70634 A/Shanghai/Mix1/2014
AHJ57411 A/Shanghai/PD-01/2014
AJJ91921 A/Shenzhen/SP-W1/2014
AJJ91873 A/Shenzhen/SP-Z93/2014
AJJ95644 A/Shenzhen/SP113/2014
AJJ95656 A/Shenzhen/SP116/2014
AJJ95668 A/Shenzhen/SP118/2014
AJJ96829 A/Shenzhen/SP126/2014
AJJ96841 A/Shenzhen/SP139/2014
AJJ91933 A/Shenzhen/SP16/2014
AJJ91897 A/Shenzhen/SP17/2014
AJJ91909 A/Shenzhen/SP26/2014
AJJ91945 A/Shenzhen/SP38/2014
AJJ91885 A/Shenzhen/SP4/2014
AJJ91957 A/Shenzhen/SP44/2014
AJJ91969 A/Shenzhen/SP48/2014
AJJ91981 A/Shenzhen/SP49/2014
AJJ95596 A/Shenzhen/SP58/2014
AJJ95608 A/Shenzhen/SP60/2014
AJJ95632 A/Shenzhen/SP62/2014
AJJ95620 A/Shenzhen/SP75/2014
AGR84966 A/Suzhou/3/2013
AGR84942 A/Suzhou/5/2013
AGL43637 A/Taiwan/1/2013
AGN69474 A/Wuxi/1/2013
AGN69462 A/Wuxi/2/2013
AGR84990 A/Wuxi/3/2013
AGR84978 A/Wuxi/4/2013
AGO02477 A/Xuzhou/1/2013
ARG43206 A/Yunnan/0129/2017
AJS16350 A/Zhejiang/17/2014
AHW83651 A/Zhejiang/DTID-ZJU01/2013
AGJ51953 A/Zhejiang/DTID-ZJU01/2013
AGU02227 A/Zhejiang/DTID-ZJU02/2013
AGM16242 A/Zhejiang/HZ1/2013
AGO51374 A/Zhejiang/KLED32/2013

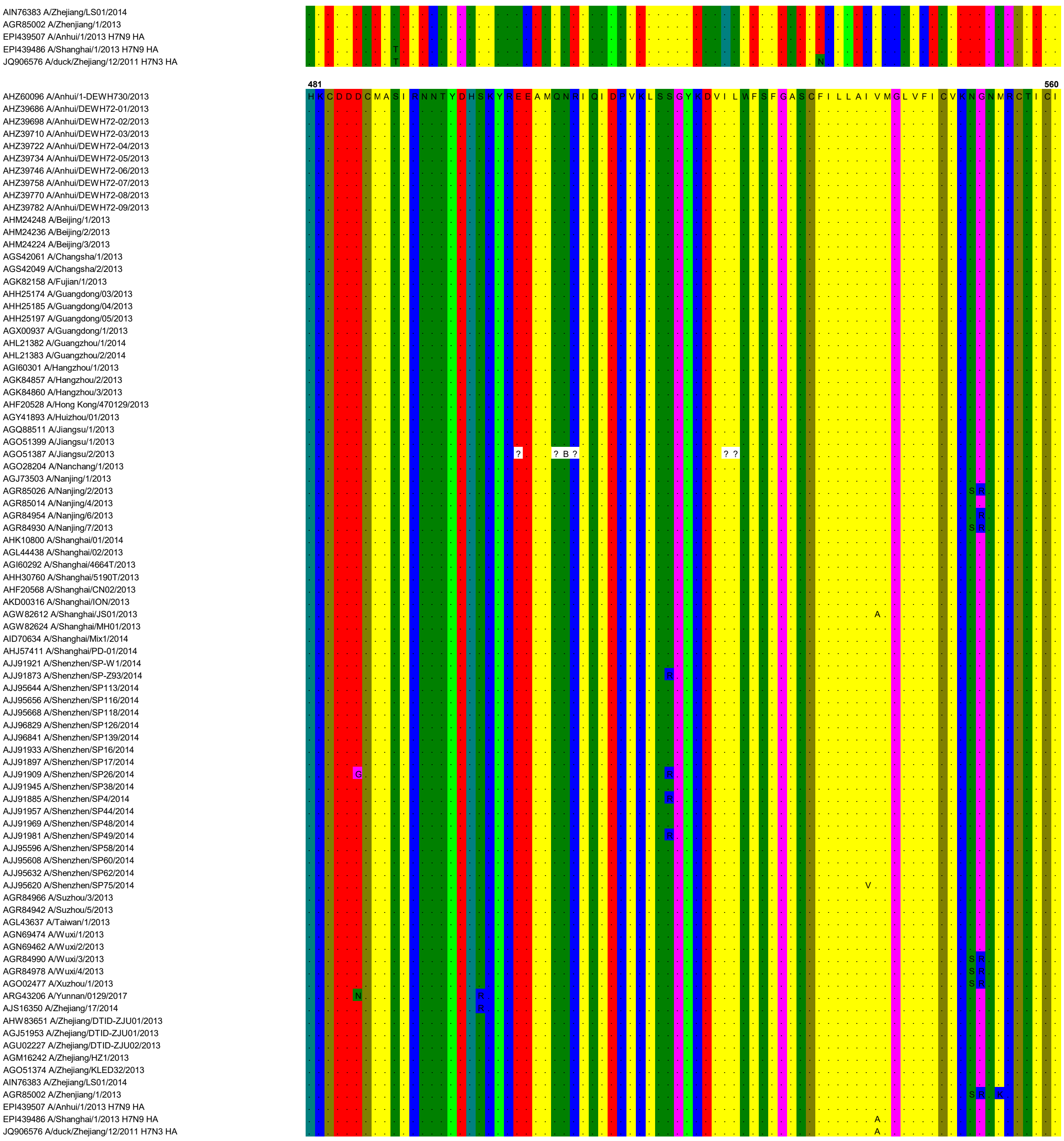
AIN76383 A/Zhejiang/LS01/2014
AGR85002 A/Zhenjiang/1/2013
EPI439507 A/Anhui/1/2013 H7N9 HA
EPI439486 A/Shanghai/1/2013 H7N9 HA
JQ906576 A/duck/Zhejiang/12/2011 H7N3 HA
481
560
AHZ60096 A/Anhui/1-DEWH730/2013
AHZ39686 A/Anhui/DEWH72-01/2013
AHZ39698 A/Anhui/DEWH72-02/2013
AHZ39710 A/Anhui/DEWH72-03/2013
AHZ39722 A/Anhui/DEWH72-04/2013
AHZ39734 A/Anhui/DEWH72-05/2013
AHZ39746 A/Anhui/DEWH72-06/2013
AHZ39758 A/Anhui/DEWH72-07/2013
AHZ39770 A/Anhui/DEWH72-08/2013
AHZ39782 A/Anhui/DEWH72-09/2013
AHM24248 A/Beijing/1/2013
AHM24236 A/Beijing/2/2013
AHM24224 A/Beijing/3/2013
AGS42061 A/Changsha/1/2013
AGS42049 A/Changsha/2/2013
AGK82158 A/Fujian/1/2013
AHH25174 A/Guangdong/03/2013
AHH25185 A/Guangdong/04/2013
AHH25197 A/Guangdong/05/2013
AGX00937 A/Guangdong/1/2013
AHL21382 A/Guangzhou/1/2014
AHL21383 A/Guangzhou/2/2014
AGI60301 A/Hangzhou/1/2013
AGK84857 A/Hangzhou/2/2013
AGK84860 A/Hangzhou/3/2013
AHF20528 A/Hong Kong/470129/2013
AGY41893 A/Huizhou/01/2013
AGQ88511 A/Jiangsu/1/2013
AGO51399 A/Jiangsu/1/2013
AGO51387 A/Jiangsu/2/2013
AGO28204 A/Nanchang/1/2013
AGJ73503 A/Nanjing/1/2013
AGR85026 A/Nanjing/2/2013
AGR85014 A/Nanjing/4/2013
AGR84954 A/Nanjing/6/2013
AGR84930 A/Nanjing/7/2013
AHK10800 A/Shanghai/01/2014
AGL44438 A/Shanghai/02/2013
AGI60292 A/Shanghai/4664T/2013
AHH30760 A/Shanghai/5190T/2013
AHF20568 A/Shanghai/CN02/2013
AKD00316 A/Shanghai/ION/2013
AGW82612 A/Shanghai/JS01/2013
AGW82624 A/Shanghai/MH01/2013
AID70634 A/Shanghai/Mix1/2014
AHJ57411 A/Shanghai/PD-01/2014
AJJ91921 A/Shenzhen/SP-W1/2014
AJJ91873 A/Shenzhen/SP-Z93/2014
AJJ95644 A/Shenzhen/SP113/2014
AJJ95656 A/Shenzhen/SP116/2014
AJJ95668 A/Shenzhen/SP118/2014
AJJ96829 A/Shenzhen/SP126/2014
AJJ96841 A/Shenzhen/SP139/2014
AJJ91933 A/Shenzhen/SP16/2014
AJJ91897 A/Shenzhen/SP17/2014
AJJ91909 A/Shenzhen/SP26/2014
AJJ91945 A/Shenzhen/SP38/2014
AJJ91885 A/Shenzhen/SP4/2014
AJJ91957 A/Shenzhen/SP44/2014
AJJ91969 A/Shenzhen/SP48/2014
AJJ91981 A/Shenzhen/SP49/2014
AJJ95596 A/Shenzhen/SP58/2014
AJJ95608 A/Shenzhen/SP60/2014
AJJ95632 A/Shenzhen/SP62/2014
AJJ95620 A/Shenzhen/SP75/2014
AGR84966 A/Suzhou/3/2013
AGR84942 A/Suzhou/5/2013
AGL43637 A/Taiwan/1/2013
AGN69474 A/Wuxi/1/2013
AGN69462 A/Wuxi/2/2013
AGR84990 A/Wuxi/3/2013
AGR84978 A/Wuxi/4/2013
AGO02477 A/Xuzhou/1/2013
ARG43206 A/Yunnan/0129/2017
AJS16350 A/Zhejiang/17/2014
AHW83651 A/Zhejiang/DTID-ZJU01/2013
AGJ51953 A/Zhejiang/DTID-ZJU01/2013
AGU02227 A/Zhejiang/DTID-ZJU02/2013
AGM16242 A/Zhejiang/HZ1/2013
AGO51374 A/Zhejiang/KLED32/2013
AIN76383 A/Zhejiang/LS01/2014
AGR85002 A/Zhenjiang/1/2013
EPI439507 A/Anhui/1/2013 H7N9 HA
EPI439486 A/Shanghai/1/2013 H7N9 HA
JQ906576 A/duck/Zhejiang/12/2011 H7N3 HA
