# Supplementary figures and images for: The host tropism of current zoonotic H7N9 viruses depends mainly on an acid-labile hemagglutinin with a single amino acid mutation in the stalk region
Source: PLoS Pathog. 2024 Oct 22;20(10):e1012427. doi: 10.1371/journal.ppat.1012427 (PMC11495601; doi:10.1371/journal.ppat.1012427)

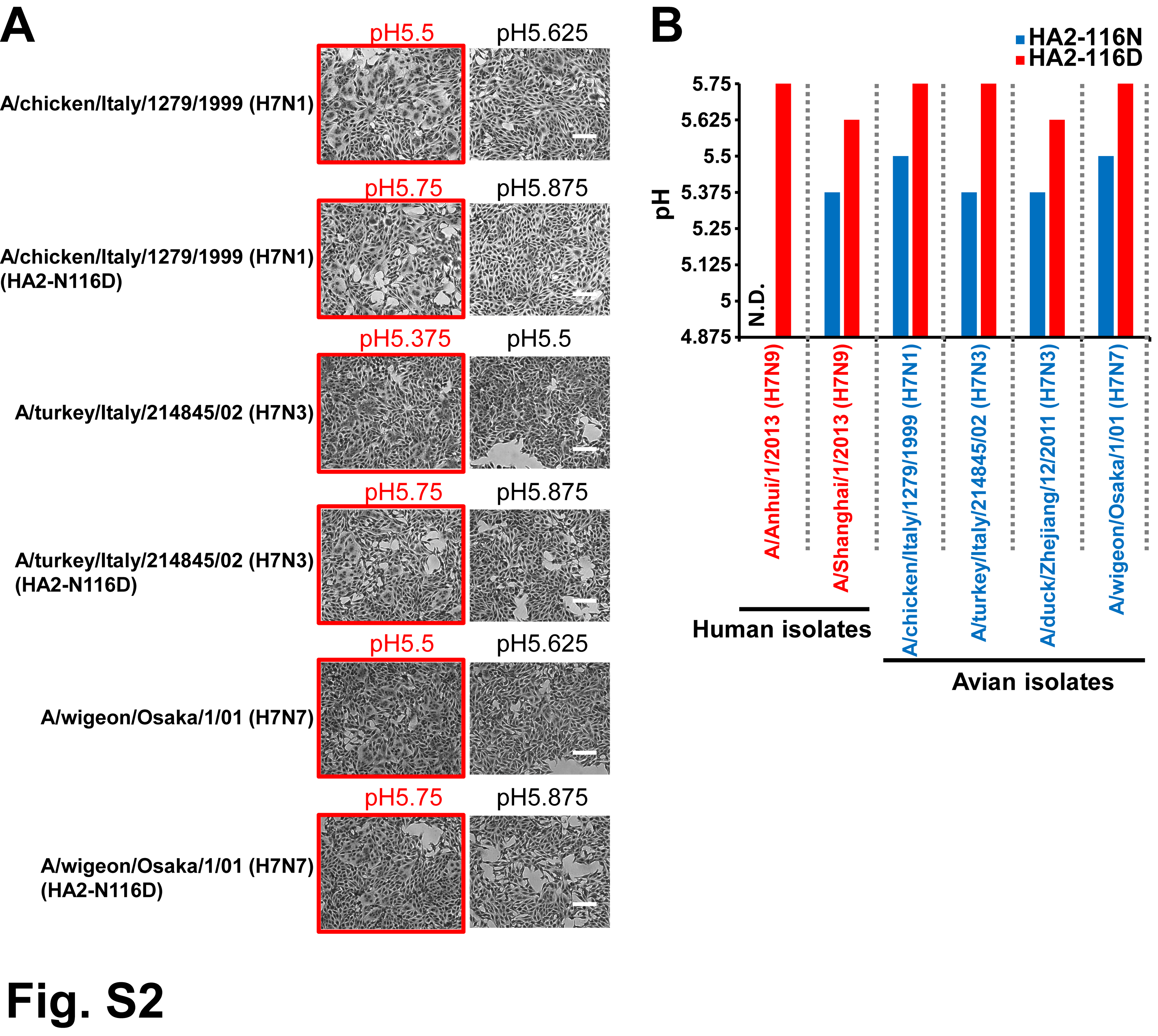

Supplement: S2 Fig — (A) MDCK cells were transfected with the influenza virus HA gene from Ck/Ita (H7N1), Tk/Ita/214845 (H7N3), or Wg/OS (H7N7), and their respective HA mutants (HA2 N116D). Fusion induction over a pH range of 5.375–5.875 was conducted as described in Fig 2. Red squares show polykaryon formation. Micrographs lacking a red square represent a pH above the fusion threshold. Values in red indicate the pH threshold for HA membrane fusion. The pH threshold was determined as described in “Materials and Methods.” Scale bars, 200 μm. (B) Summary of the pH thresholds for membrane fusion for HA mutant viruses in transfected cells. Representative results from the membrane fusion assay are shown. (TIF) [file ppat.1012427.s002.tif]

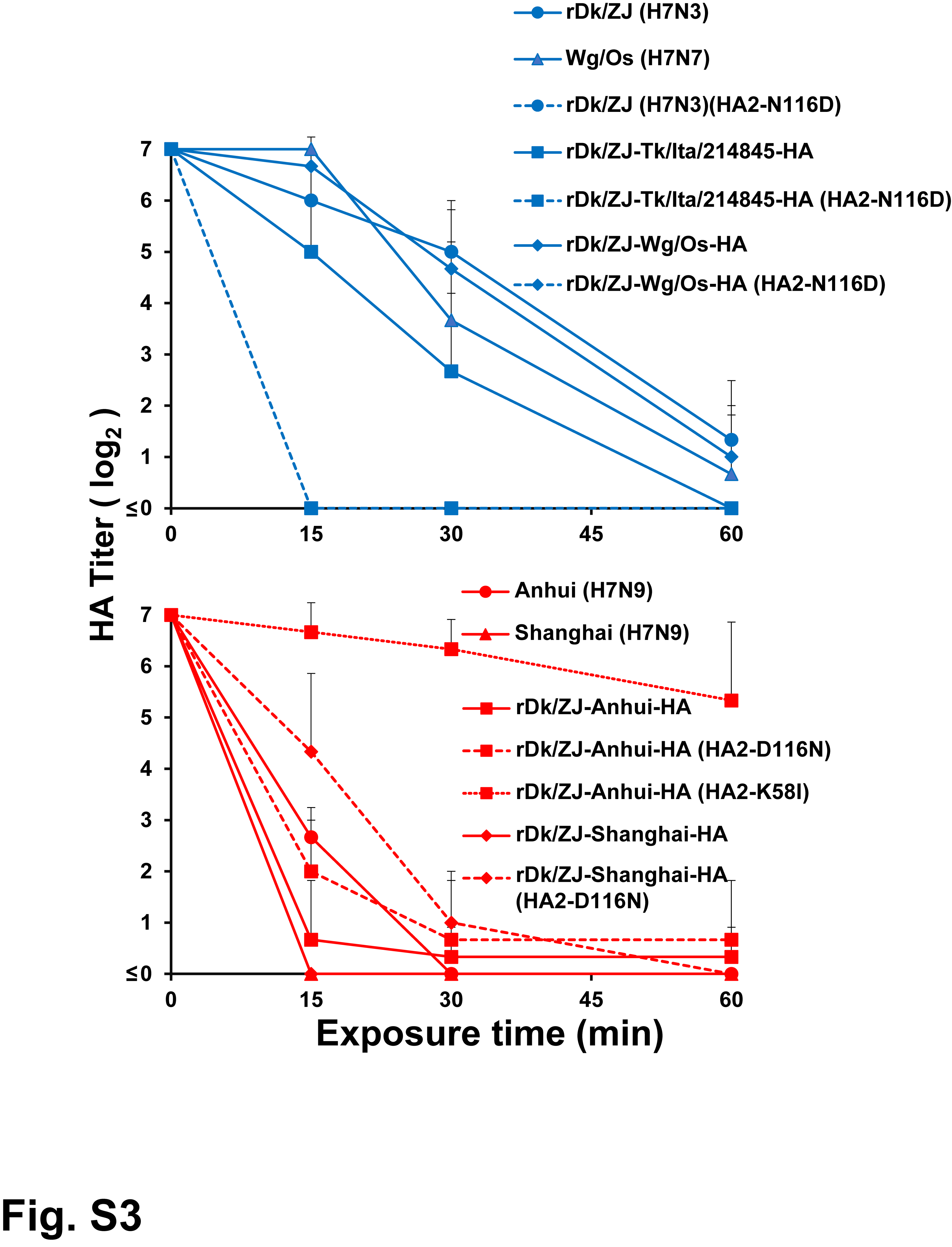

Supplement: S3 Fig — A sample of each virus (128 hemagglutination units) was incubated for the indicated times at 54°C. The hemagglutination titers of the heat-treated samples were determined in hemagglutination assays. Isolated avian viruses and recombinant Dk/ZJ (H7N3) harboring the HA gene from avian isolates are shown with a blue line; isolated human viruses and recombinant Dk/ZJ (H7N3) harboring the HA gene from human isolates are shown with a red line. Data are expressed as the mean ± S.D. of three independent results. (TIF) [file ppat.1012427.s003.tif]

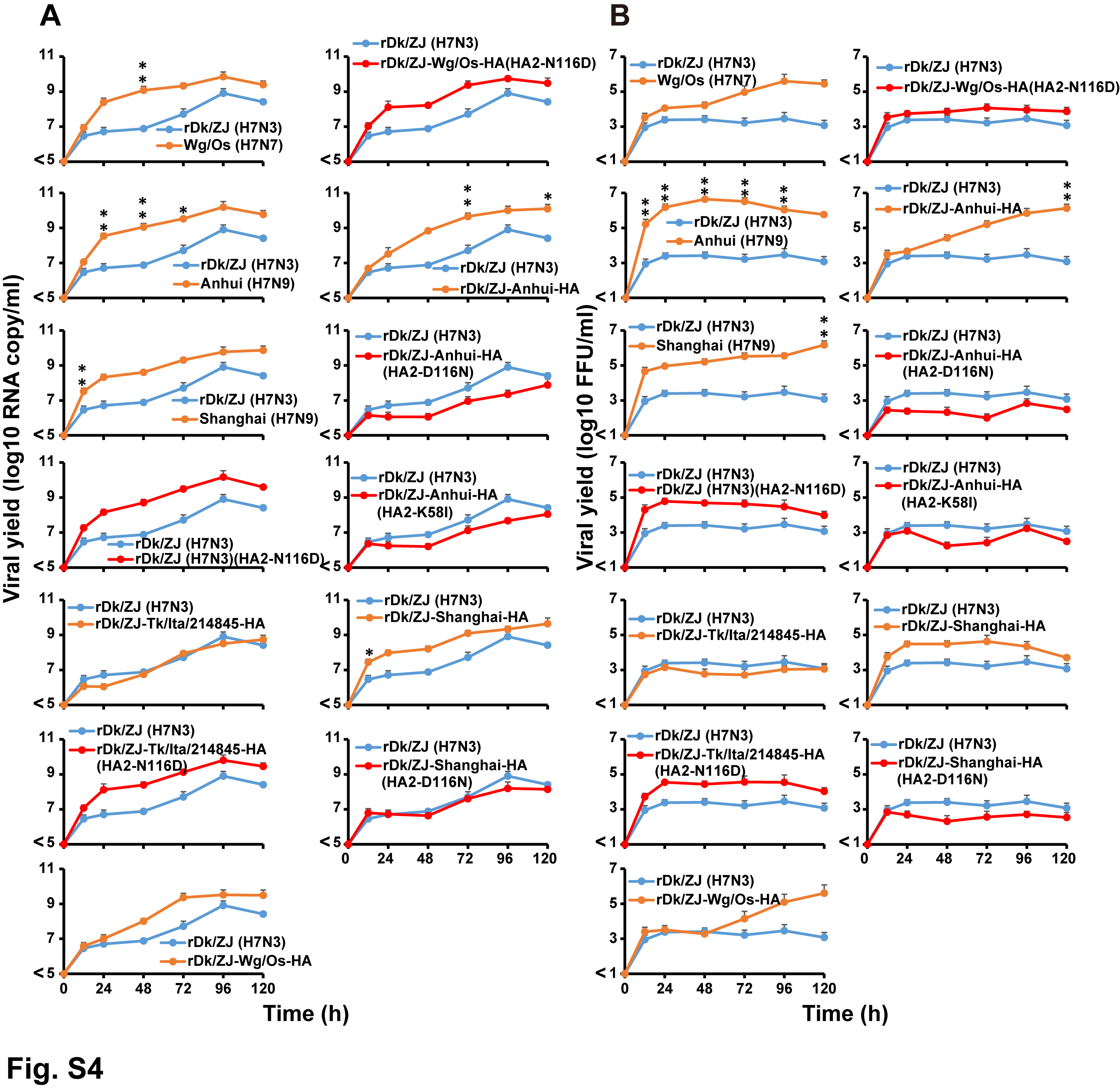

Supplement: S4 Fig — (A) 21E5 cells, derived from primary human bronchiolar epithelial cells [please see “Materials and Methods”] were infected as described in Fig 4. All cells were infected at an m.o.i. of 0.1. The amount of progeny vRNA within the culture supernatants at 12, 24, 48, 72, 96, and 120 h post-infection was determined by quantitative real-time PCR assays (the parameters of virions released at 48 and 72 h post-infection are shown in Fig 4C). (B) 21E5 cells were infected with the same virus used in (A) at an m.o.i. of 0.1. The infectious titer of released virions within the culture supernatants at 12, 24, 48, 72, 96, and 120 h post-infection was determined in a focus-forming assay (the growth curves based on the infectious virus titer of released virions at 48 and 72 h post-infection are shown in Fig 4D). Data are expressed as the mean ± S.D. of three (A) or four (B) independent results. Asterisks indicate that the value for a virus was significantly different from that of rDk/ZJ (H7N3) within the same graph. A p-value < 0.05 (single asterisk) or < 0.01 (double asterisk) was considered significant (one-way ANOVA followed by Tukey’s multiple comparisons post-hoc test). The growth kinetics of the parent strain [rDk/ZJ (H7N3)] and a recombinant Dk/ZJ (H7N3) virus harboring HA gene of other H7 viruses are shown in blue and orange, respectively. The growth kinetics of recombinant H7 viruses with a specific mutation (HA2-D116N, HA2-N116D, or HA2-K58I) are shown in red. (TIF) [file ppat.1012427.s004.tif]

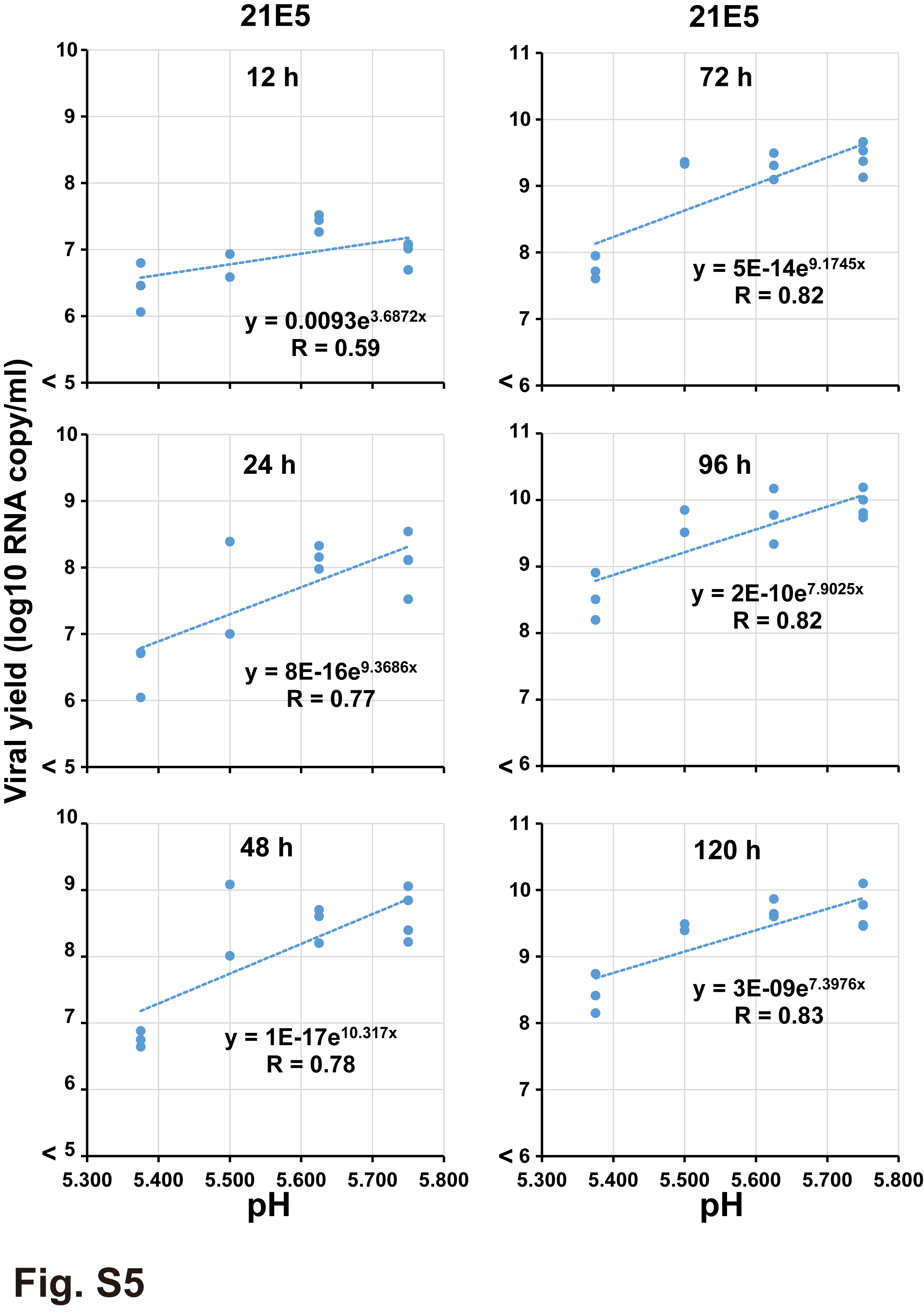

Supplement: S5 Fig — All parameters were taken from Figs 2, S2 and S4. (TIF) [file ppat.1012427.s005.tif]

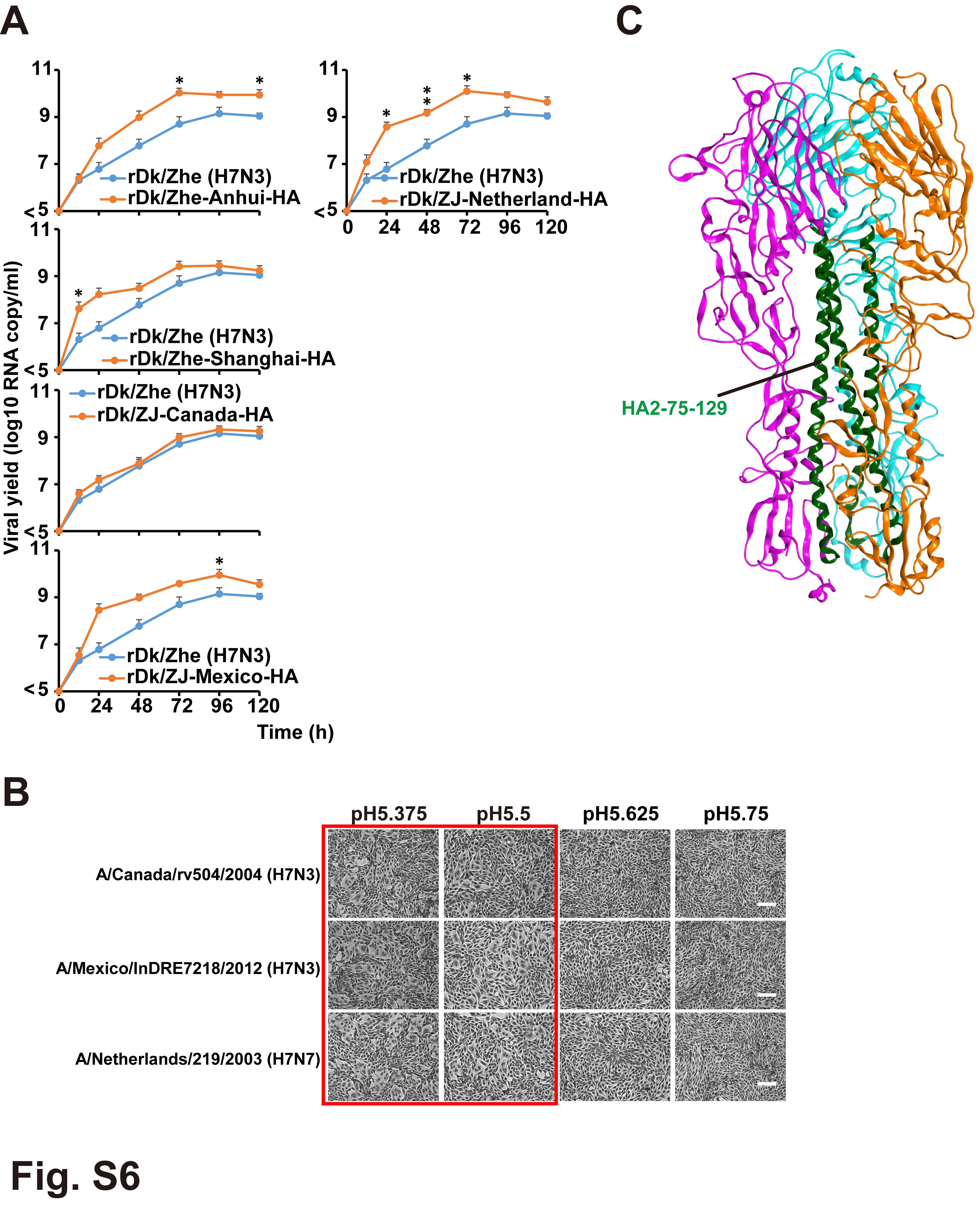

Supplement: S6 Fig — (A) 21E5 cells were infected with rDk/ZJ (H7N3) or with recombinant Dk/ZJ (H7N3) [rDk/ZJ-Anhui-HA, rDk/ZJ-Shanghai-HA, rDk/ZJ-Canada-HA, rDk/ZJ-Mexico-HA, or rDk/ZJ-Netherland-HA]. All cells were infected at an m.o.i. of 0.1. The amount of progeny viral RNA within the culture supernatants at 12, 24, 48, 72, 96, and 120 h post-infection was determined by measuring virus titers in quantitative real-time PCR assays. Data are expressed as the mean ± S.D. of four independent results. Asterisks indicate that the values for each virus were significantly different from that of rDk/ZJ (H7N3) within the same graph. A p-value < 0.05 (single asterisk or sharp) or < 0.01 (double asterisk or double sharp) was considered significant (one-way ANOVA followed by Tukey’s multiple comparisons post-hoc test). (B) MDCK cells were transfected with the influenza virus HA gene from Canada (H7N3), Mexico (H7N3), and Netherland (H7N7) and the acid stability of the HA protein was examined in a membrane fusion assay (conducted as described in Fig 2). Representative fields of cells transfected with each of the indicated viruses and exposed to low pH are shown. Red squares show polykaryon formation. Micrographs lacking a red square represent a pH above the fusion threshold. (C) The interaction energies between helix (HA2-75–129) on the stalk and adjacent HA molecules or the other helices (HA2-75–129) on the opposite site were analyzed to assess the structural stability of HA trimetric molecules (please see S1 and S2 Tables). The structural stability of the HA trimer of Canada (H7N3), Mexico (H7N3), and Netherland (H7N7) was compared with that of Dk/ZL (H7N3). Analyzed helix regions (HA2-75–129) are shown as green in the ribbon model of the HA trimer from Dk/ZJ (H7N3). (TIF) [file ppat.1012427.s006.tif]

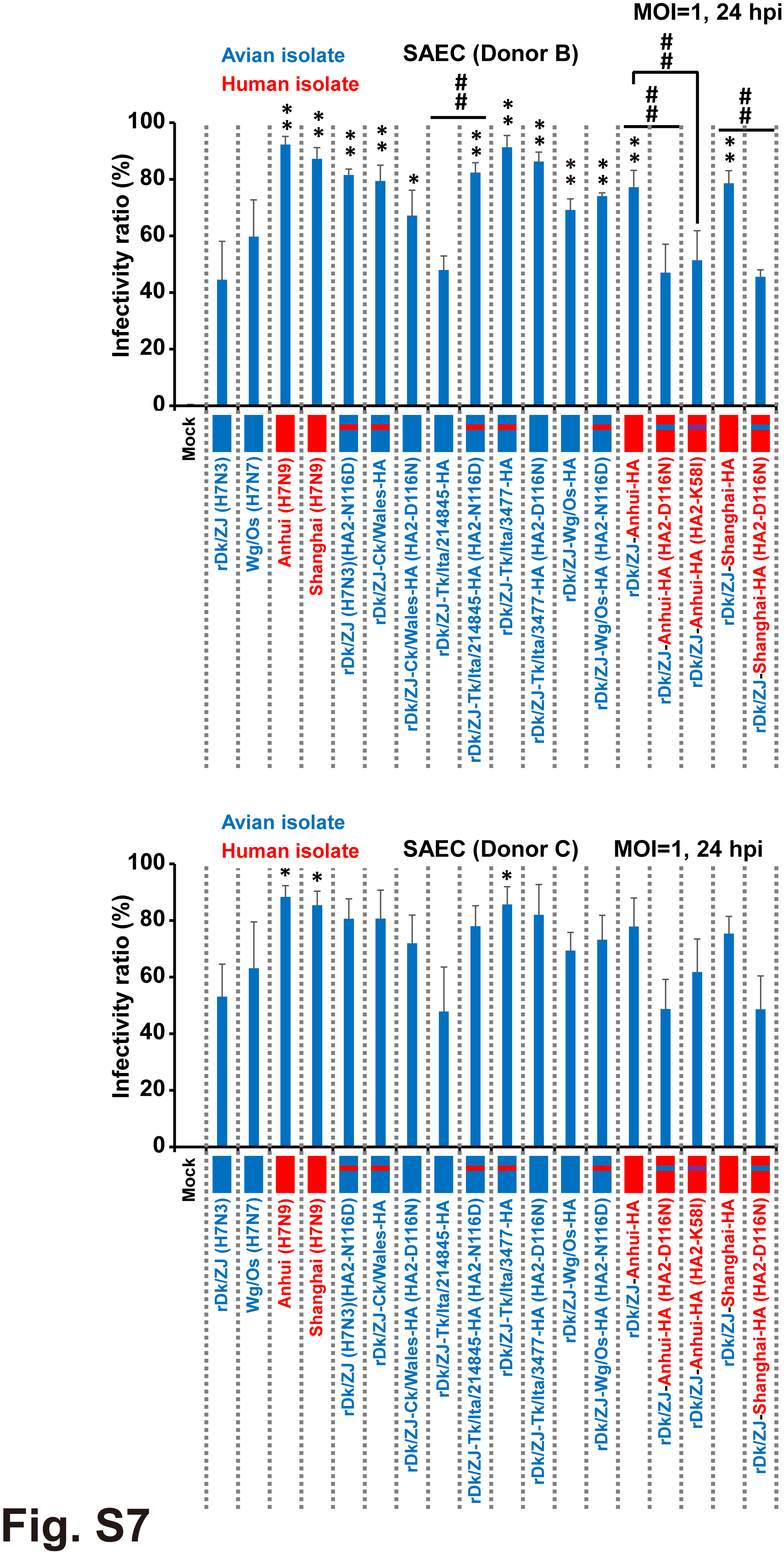

Supplement: S7 Fig — Primary human bronchiolar epithelial cells [SAECs, see “Materials and Methods”] from different donors (denoted as donor B and C; SAECs in Fig 6 are from donor A) were infected with wild-type and recombinant H7 viruses. An infectious assay was conducted as described in Fig 6. The experimental conditions and virus strains are same as in Fig 6. Viral infectivity was determined by calculating the percentage of antigen-positive cells after immunostaining at 24 h post-infection. Data are expressed as the mean ± S.D. of three independent results. Asterisks indicate that the value for each virus was significantly different from that of rDk/ZJ (H7N3) within the same graph; sharps indicate that the value for each virus harboring a mutation (HA2-N116D, HA2-D116N, or HA2-K58I) was significantly different from that of each virus without a mutation within the same graph. A p-value < 0.05 (single asterisk or sharps) or < 0.01 (double asterisk or sharp) was considered significant (one-way ANOVA followed by Tukey’s multiple comparisons post-hoc test). The mutated amino acids (HA2-N116D or HA2-D116N) are shown in red (HA2-N116D) and blue (HA2-D116N), respectively. The mutated amino acid HA2-K58I is shown in purple. The position of HA2-116D (native residue) in both rDk/ZJ-Ck/Wales-HA and rDk/ZJ-Tk/Ita/3477-HA is also shown in red. Please note that the mutated amino acid (HA2-116) in rDk/ZJ-Ck/Wales-HA (HA2-D116N) and rDk/ZJ-Tk/Ita/3477-HA (HA2-D116N) is shown in the same color as the background (blue). (TIF) [file ppat.1012427.s007.tif]

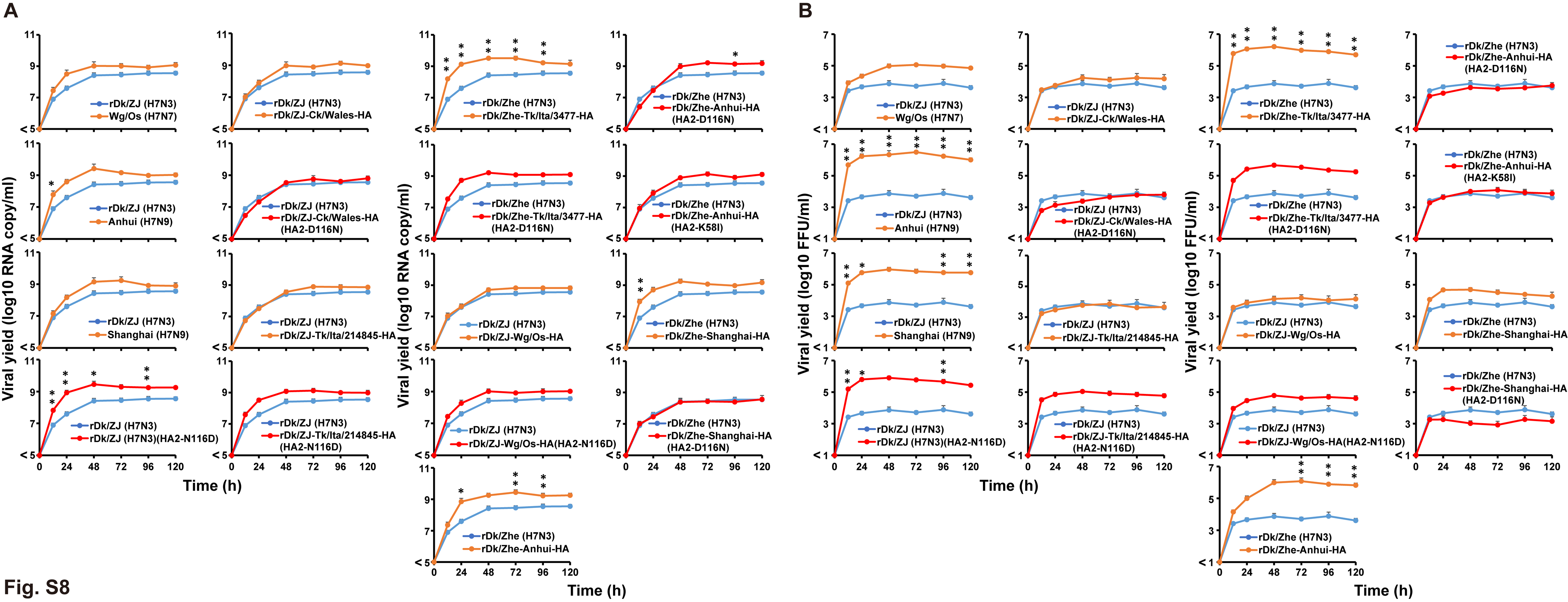

Supplement: S8 Fig — (A) Primary human bronchiolar epithelial cells [SAECs, see “Materials and Methods”] were infected as described in Fig 6. All cells were infected at an m.o.i. of 0.1. The amount of progeny vRNA within the culture supernatants at 12, 24, 48, 72, 96, and 120 h post-infection was determined by measuring virus titers in quantitative real-time PCR assays (the parameters of released virions at 24 and 48 h post-infection are shown in Fig 6B). (B) SAECs were infected with same virus used in (A) at an m.o.i. of 0.1. The infectious virus titer of the released virions within the culture supernatants at 12, 24, 48, 72, 96, and 120 h post-infection was determined in a focus-forming assay (the growth curves based on the infectious virus titer of released virions at 24 and 48 h post-infection are shown in Fig 6C). Data are expressed as the mean ± S.D. of three independent results. Asterisks indicate that the value for each virus was significantly different from that of rDk/ZJ (H7N3) within the same graph. A p-value < 0.05 (single asterisk) or < 0.01 (double asterisk) was considered significant (one-way ANOVA followed by Tukey’s multiple comparisons post-hoc test). The growth kinetics of parent strain [rDk/ZJ (H7N3)] and recombinant Dk/ZJ (H7N3) harboring the HA gene of other H7 viruses are shown as blue and orange lines, respectively. The growth kinetics of recombinant H7 viruses harboring a specific mutation (HA2-D116N, HA2-N116D, or HA2-K58I) are shown as a red line. (TIF) [file ppat.1012427.s008.tif]
