# Supplementary material for: The host tropism of current zoonotic H7N9 viruses depends mainly on an acid-labile hemagglutinin with a single amino acid mutation in the stalk region
Source: PLoS Pathog. 2024 Oct 22;20(10):e1012427. doi: 10.1371/journal.ppat.1012427 (PMC11495601; doi:10.1371/journal.ppat.1012427)
Supplement: S1 Table — The amino acid sequence of the analyzed helix within the HA2 domain corresponds to HA2-75–129 (please see S6C Fig). (DOCX) [file ppat.1012427.s009.docx]

**S1 TABLE. Interaction energies between the helix within the HA2 domain of one monomer and the other two HA molecules**

**Monomer A–B/C Monomer B–C/A Monomer C–A/B Average (kcal/mol)**

A/duck/Zhejiang/12/2011(H7N3) -1595.24 -1555.18 -1511.1 -1553.84

A/Canada/rv504/2004 (H7N3) -1354.16 -1313.79 -1398.92 -1355.62

A/Mexico/InDRE7218/2012 (H7N3) -1572.28 -1469.37 -1406.29 -1482.65

A/Netherlands/219/2003 (H7N7) -1560.49 -1560.43 -1471.67 -1530.86

The amino acid sequences of the analyzed helix within the HA2 domain correspond to HA2-75–129 (please see S6C Fig).
