# Supplementary material for: The host tropism of current zoonotic H7N9 viruses depends mainly on an acid-labile hemagglutinin with a single amino acid mutation in the stalk region
Source: PLoS Pathog. 2024 Oct 22;20(10):e1012427. doi: 10.1371/journal.ppat.1012427 (PMC11495601; doi:10.1371/journal.ppat.1012427)
Supplement: S2 Table — The amino acid sequence of the analyzed helix within the HA2 domain corresponds to HA2-75–129 (please see S6C Fig). (DOCX) [file ppat.1012427.s010.docx]

**S2 TABLE. Interaction energies between the helix within the HA2 domain of one monomer and the helices within the other two HA molecules**

**Monomer A–B/C Monomer B–C/A Monomer C–A/B Average (kcal/mol)**

A/duck/Zhejiang/12/2011(H7N3) -145.01 -155.07 -159.02 -153.03

A/Canada/rv504/2004 (H7N3) -125.08 -151.65 -141.41 -139.38

A/Mexico/InDRE7218/2012 (H7N3) -103.69 -129.62 -122.5 -118.60

A/Netherlands/219/2003 (H7N7) -131.26 -146.33 -164.9 -147.50

The amino acid sequences of the analyzed helix within the HA2 domain correspond to HA2-75–129 (please see S6C Fig).
